# Starch Wars—New Episodes of the Saga. Changes in Regulations on Hydroxyethyl Starch in the European Union

**DOI:** 10.3389/fvets.2018.00336

**Published:** 2019-01-18

**Authors:** Katja-Nicole Adamik, Ivayla D. Yozova

**Affiliations:** ^1^Department of Clinical Veterinary Medicine, Vetsuisse Faculty, University of Bern, Bern, Switzerland; ^2^School of Veterinary Science, Massey University, Palmerston North, New Zealand

**Keywords:** gelatin, dextran, plasma expanders, pharmacovigilance, synthetic colloids, fluid therapy, European Medicines Agency, Food and Drug Administration

## Abstract

After a safety review of hydroxyethyl starch (HES) solutions in 2013, restrictions on the use of HES were introduced in the European Union (EU) to reduce the risk of kidney injury and death in certain patient populations. Similar restrictions were introduced by the Food and Drug Administration in the United States and other countries. In October 2017, a second safety review of HES solutions was triggered by the European pharmacovigilance authorities based on a request by the Swedish Medical Products Agency to completely suspend HES. After several meetings and repeated evaluations, the recommendation to ban HES was ultimately not endorsed by the responsible committee; however, there was a vote for more restricted access to the drug and rigorous monitoring of policy adherence. This review delineates developments in the European pharmacovigilance risk assessment of HES solutions between 2013 and 2018. In addition, the divergent experts' opinions and the controversy surrounding this official assessment are described. As the new decisions might influence the availability of HES products for veterinary patients, potential alternatives to HES solutions, such as albumin solutions and gelatin, are briefly discussed.

First introduced in the 1960s, hydroxyethyl starch (HES) rapidly became the most commonly used synthetic colloid in human intensive care units (ICUs) throughout the world, with over 60 products registered in Europe and four in the United States (US) by the year 2010 (1–3). The first veterinary reviews advocating the use of HES date from the 1980s and, until recently, veterinarians have been widely using these products to treat anesthesia-induced hypotension and hypovolemia non-responsive to crystalloids (4, 5). Additionally, HES has been used to increase intravascular colloid osmotic pressure in hypoalbuminemic animals by administering it as a low-dose constant rate infusion over several days (6–8). However, an increasing number of potential side effects of HES administration in both humans and animals have since come to light. These include tissue accumulation, acute kidney injury (AKI), coagulopathies and bleeding tendencies, anaphylactoid reactions, and pruritus (the latter only described in humans) (9–11). The nephrotoxic effects of HES have been proposed to be secondary to renal tissue uptake and intracellular storage based on studies in small populations of patients (12, 13). As HES molecules cannot be degraded once they leave the blood, HES leads to vacuolization, swelling, and subsequent cellular dysfunction in the kidney (12, 13). HES-induced coagulopathies and bleeding are suggested to be the result of direct effects on hemostasis through impaired fibrinogen/fibrin polymerization and platelet dysfunction leading to a weaker clot (14, 15).

Awareness of these side effects has resulted in a succession of changes to the warnings, restrictions, and contraindications on HES product packaging (**Table 2**) and prompted lively debates in the intensive care community about the potential risks and theoretical benefits of HES administration. This review traces back the history and summarizes the current state of regulations on HES use in human medicine. Its purpose is to inform the reader on the origins of the controversy surrounding HES use. Furthermore, a brief overview on HES-related veterinary literature as well as alternatives to HES is presented to help the reader understand the impact (or lack thereof) of the potential loss of access to HES for veterinary patients.

## The History of HES Restrictions—First Episode 2013

In 2008, the *Efficacy of Volume Substitution and Insulin Therapy in Severe Sepsis* (VISEP) trial, a multicenter human randomized controlled trial (RCT) conducted in 537 septic patients in Germany, found higher rates of AKI and need for renal-replacement therapy (RRT) associated with the use of 10% HES 200/0.5 than with Ringer's lactate (16). In this study, a number of patients received higher than recommended dosages of the hyperoncotic HES 200/0.5 (>22 ml/kg per day); however, the increased risk to need RRT was also seen in patients treated with HES 200/0.5 at the recommended daily doses (16). This trial was followed by three other large human multicenter RCTs in critically ill or septic patients in 2012 (17–19). The *Scandinavian Starch for Severe Sepsis/Septic Shock* (6S trial) involving 798 septic patients, found an increased requirement for RRT and higher mortality at 90 days in patients receiving 6% HES 130/0.42 compared to Ringer's acetate (17). The *Crystalloid vs. Hydroxyethyl Starch Trial* (CHEST trial) involving 7,000 critically ill patients in Australia and New Zealand, also found a higher requirement for RRT but no difference in 90-day mortality in patients receiving 6% HES 130/0.4 compared to saline (18). Finally, the *Assessment of Hemodynamic Efficacy and Safety of 6% Hydroxyethylstarch 130/0.4 vs. 0.9% NaCl Fluid Replacement in Patients with Severe Sepsis* (Crystalloids Morbidity Associated with severe Sepsis, CRYSTMAS) trial involving 196 septic patients in France and Germany, found no difference in adverse events, including AKI and mortality, between patients receiving 6% HES 130/0.4 compared to saline (19).

Simultaneously with the aforementioned trials, between 2009 and 2013, one of the largest scandals of scientific misconduct in history was unraveled (20, 21). This involved retractions of over 90 HES-related scientific articles of the (back then) prominent German anesthetist and prolific defender of HES, Joachim Boldt, for data fabrication and lack of ethics approval (22, 23). Data from studies by Boldt and coworkers were included in systematic reviews and used to form clinical guidelines worldwide (24, 25). In particular, re-evaluation of one meta-analysis that originally reported no association between HES administration and all-cause mortality, revealed a significantly increased risk of mortality and AKI when 7 studies from Boldt were subsequently excluded from the analysis (26). This elucidates the fact that the fraudulent data from Boldt's many years of research, which mostly favored HES, might have previously tipped the “meta-analytical scales” and masked the potential harm of HES products for many years (21).

Following, VISEP (16), 6S (17), CHEST (18), and CRYSTMAS (19), both the US Food and Drug Administration (FDA) and the European Medicines Agency (EMA) initiated a safety review of HES products. Both agencies reviewed data from RCTs, meta-analyses and observational studies, with a particular emphasis on 6S (17) (FDA and EMA), CRYSTMAS (19) (FDA only), and CHEST (18) and VISEP (16) (EMA only) (3). The EMA review was started in November 2012 by the Pharmacovigilance Risk Assessment Committee (PRAC) as a referral procedure (Article 31 of Directive 2001/83/EC) and was initiated at the request of the German medicines agency (Data Sheet 1) (27, 28) (Table 1). The PRAC is a section of the EMA composed of experts from each of the member states of the EU as well as members representing patient organizations responsible for all aspects of risk management of medicines (43). In June 2013, the PRAC issued a recommendation to suspend marketing authorization for all HES products as it concluded that the risks of HES outweighed its benefits (29) (Table 1). In the interim, HES was banned by the British Medicines and Healthcare Products Regulatory Agency, and a drug alert was issued in the United Kingdom calling for the return of all HES products to the manufacturers (44). Simultaneously, the United Kingdom triggered an urgent Union procedure (Article 107i of Directive 2001/83/EC) which was performed by PRAC as well (31). Such procedure is automatically triggered when a member state considers suspending marketing authorization or prohibiting supply of a medical product (45) (Table 1).

**Table 1:** Timeline for regulatory key events in 2013 and 2018 for hydroxyethyl starch (HES) restrictions by the European Medicines Agency's (EMA) Pharmacovigilance Risk Assessment Committee (PRAC).

**Date**	**Event**
30th November 2012	Review of HES solutions started under Article 31 of Directive 2001/83/EC (28).
14th June 2013	PRAC recommends suspending marketing authorizations for infusion solutions containing HES (29).
12th July 2013	Recommendation to suspend marketing authorizations for HES solutions to be re-examined under Article 31 of Directive 2001/83/EC (30).
12th July 2013	New review of HES-containing solutions for infusion started under Article 107i of Directive 2001/83/EC (urgent Union procedure triggered by the United Kingdom) (31).
11th October 2013	PRAC confirms that HES should no longer be used in patients with sepsis or burn injuries or in critically ill patients. HES will be available in restricted patient populations (32).
25th October 2013	CMDh endorses PRAC recommendations: HES solutions should no longer be used in patients with sepsis or burn injuries or in critically ill patients (33)
6th March 2014	European Commission final decision: HES solutions no longer to be used in patients with sepsis or burn injuries or in critically ill patients (34)
27th October 2017	EMA starts new review of HES containing medicines at the request of the Swedish Medical Products Agency, under Article 107i of Directive 2001/83/EC (35).
12th January 2018	PRAC recommends suspending HES solutions for infusion from the market (36).
26th January 2018	HES solutions for infusion to be suspended—CMDh endorses PRAC recommendation (37).
9th April 2018	European Commission/ Meeting of the Standing Committee On Medical Products For Human Use refers back the CMDh position/PRAC recommendation to the European Medicines Agency for further consideration (38).
17th May 2018	PRAC confirms its recommendation to suspend HES solutions for infusion in the EU (39, 40).
29th June 2018	HES solutions: CMDh introduces new measures to protect patients (41).
27th July 2018	European Commission final decision: Scientific conclusions and CMDh's detailed explanation on the scientific grounds for differences with the PRAC recommendation (42).

*CMDh, Coordination Group for Mutual Recognition and Decentralized Procedures—Human*.

Numerous commentaries in scientific journals were exchanged between experts with different opinions regarding the risks and benefits of HES, and the quality of the aforementioned landmark trials. Concerns raised by HES proponents (and Marketing Authorization Holders [MAHs]) with regard to the VISEP (16), 6S (17), and CHEST (18) trials included that patients were entered into studies several hours after admission to ICU (up to 24 h after) and were possibly hemodynamically stable at randomization, wherefore HES was no longer indicated. Also, a significant number of patients who were randomized to receive HES were in renal failure. Namely, renal failure without RRT was not an absolute contraindication for HES according to the steering committee and scientific advisors of the 6S (17) trial, despite that renal failure was already a contraindication for HES [stated e.g., by the American FDA (46)] before 6S was initiated (47). Some patients randomized to the crystalloid group had received initial treatment with colloids, and there was a lack of specific criteria for starting RRT. Finally, there was no justification for extrapolating findings in critically ill patients to the entire patient population (47–50). HES opponents disagreed with these critiques against VISEP (16), 6S (17), and CHEST (18) and argued that data were misread and misinterpreted by the critics (51).

In March 2013, another large human RCT was published: The *Colloids vs. Crystalloids for the Resuscitation of the Critically Ill* (CRISTAL) trial randomized 2,857 critically ill patients in 57 ICUs in France, Belgium, North Africa, and Canada, requiring fluid resuscitation for acute hypovolemia with either colloids (gelatins, dextrans, HES, or albumin) or crystalloids (isotonic saline, hypertonic saline, or any other buffered solution) (52). Even though the trial was open-label, the outcome assessment was blinded, and patients received HES within the maximum dose limit. The study found no significant difference in the 28-day mortality between subjects receiving colloids and those receiving crystalloids, but a significant reduction in mortality at 90 days in the colloid group, as well as more vasopressor-free and more ventilator-free days by day 28 (52). Subgroup analysis confirmed a significantly reduced 90-day mortality in patients treated with HES when compared with patients treated with 0.9% saline. In contrast to VISEP (16), 6S (17), and CHEST (18) the authors of CRISTAL (52) recruited patients newly admitted to the ICU as soon as resuscitation was required. Nevertheless, the trial had limitations, such as long duration of the trial (9 years), open-label design, only 70% of patients in the colloid group received HES, overlap between treatments with the different colloids, some patients received more than one type of colloid, and a high proportion of patients in both groups received colloids prior to ICU colloid administration (52).

Following requests for re-examination by MAHs, including Fresenius Kabi and B. Braun Melsungen AG (located in Germany), a second PRAC committee, composed of a different expert group, re-evaluated the evidence (30) (Table 1). The re-examination focused on the benefit-risk ratio of HES in the treatment of hypovolemic shock in surgery and trauma patients (53). Consequently, two reviews were running in parallel by different expert committees of the PRAC: The re-examination under Article 31 of Directive 2001/83/EC [requested by the MAHs) (30)] and the additional review under Article 107i of Directive 2001/83/EC [triggered by the United Kingdom in June 2013 (31)]. Both reviews were finalized in October 2013 but came to different conclusions. The PRAC review under Article 31 maintained its recommendation (from June 2013) for suspension of marketing authorization for HES (53). However, a re-examination only looks at the evidence provided for the original procedure and therefore, this committee did not include new data (53). In contrast, the PRAC review under Article 107i included new data, which were not available or not considered in the referral under Article 31 and did not recommend an absolute HES suspension (54). Factors that led to a different assessment of that review were short-term hemodynamic improvement (55), volume-sparing effect (56), prevention and limitation of edema formation (57), significantly lower estimated blood loss (58), and reduction in red blood cell transfusions (59) in surgical and trauma patients receiving HES (54). Further, studies provided some evidence that the risks of AKI (60, 61) and death (52, 62) in these patients may be lower than in critically ill and septic patients (54). The PRAC's final recommendation on the use of HES solutions was based on the new evidence considered in the Article 107i procedure and the decision was published in October 2013 on the EMA website (32) (Table 1). Notably, in the online regulatory texts provided by the EMA, no difference is made regarding different HES preparations (e.g., second generation HES 200/0.5 vs. third generation HES 130/0.4 or 0.42, respectively). The EMA recommended that HES should no longer be used in critically ill patients or those with sepsis or burn injuries (Table 2), but it could still be administered to patients with hypovolemia due to acute blood loss if treatment with crystalloids was inadequate. In these patients, HES should be only given for initial volume resuscitation with a maximum dose of 30 ml/kg and kidney function should be monitored for 90 days (although the type of monitoring was not specified) (32). Only Annex III “Amendments to relevant sections of the summary of product characteristics and package leaflet” specifically mentioned the HES preparations (HES 130/0.4 and HES 130/0.42, respectively) and stated that for other HES products (e.g., HES 200/0.5) the maximum daily dose should be recalculated accordingly (63).

**Table 2:** Contraindications for hydroxyethyl starch products before and after 2013 in the European Union (EMA) and United States (FDA).

**EMA**	**FDA**
**Contraindications before 2013** Renal failure (with oliguria or anuria) Patients on dialysis Hypersensitivity Congestive heart failure Hyperhydration states (including pulmonary edema) Intracranial bleeding Severely impaired hepatic function Hyperkalemia Severe hypernatremia or hyperchloremia Clinical conditions with volume overload	**Contraindications before 2013** Renal failure (with oliguria or anuria) Hypersensitivity Congestive heart failure Treatment of lactic acidosis Patients on dialysis Clinical conditions with volume overload
**Additional contraindications in 2013** Critically ill patients Sepsis Burn injuries Renal impairment Renal replacement therapy Severe coagulopathy and bleeding Organ transplant patients	**Additional contraindications in 2013** Critically ill adult patients Sepsis Renal dysfunction Severe liver disease Pre-existing coagulation/bleeding disorders Patients undergoing open heart surgery in association with cardiopulmonary bypass
**Additional contraindications in 2018** Fluid maintenance therapy Dehydrated patients Cerebral hemorrhage	**No update**

*EMA, European Medicines Agency; FDA, United States Food and Drug Administration*.

This decision was not unanimously supported, as 14 of the 33 PRAC members voted against the revision (54). Nevertheless, the decision was endorsed by the Coordination Group for Mutual Recognition and Decentralized Procedures—Human (CMDh, responsible for examining questions relating to marketing authorization of human medicines in the EU, composed of one representative per EU Member State) (33), and then by the European Commission, the governing body of the EU, for legal binding in the EU (34) (Table 1). As a condition from the PRAC, the MAHs were asked to conduct drug utilization studies in several member states to evaluate the effectiveness of the risk minimization measures taken. The goal of these drug utilization studies was to characterize prescribing practices during typical clinical use in representative groups of prescribers to verify adherence to the updated product information. As further conditions, changes to the product information, information to the healthcare professionals and patients, and RCTs conducted by the MAHs in order to demonstrate the efficacy and safety of HES in the perioperative and trauma populations were requested (54).

For the FDA review, an expert workshop was set up to review HES safety (64). This led to new safety information added to HES product labeling in November 2013 in the form of a black box warning. It stated not to use HES in critically ill adult patients, and patients with sepsis, severe liver disease, pre-existing coagulopathy, and in patients undergoing open heart surgery in association with cardiopulmonary bypass (Data Sheet 2) (65) (Table 2).

The revised EMA decision was criticized, as it was (for the most part) based on the same studies as the ban had been based on, but with different conclusions (66). HES opponents argued that the risks of HES outweighed the benefits, that there was insufficient evidence that colloid resuscitation improved outcome in surgical and trauma patients, and that a number of other, safer alternative intravenous fluids existed (67, 68). In a 2014 open letter to the Executive Director of the EMA, the authors (overall 70 intensive care researchers) asked, “what assumptions or clinical data would indicate that the same pathological mechanisms [tissue storage with subsequent organ injury, and coagulopathy] do not apply in patients with hypovolemia from blood loss” and argued that the known side effects should be considered to be potential risks in all patient groups. Further, the clinical trials recommended by the PRAC to prove the safety of HES would expose surgical and trauma patients to known risks of harm [e.g., risk of AKI and bleeding] without a proven benefit (67). At the same time, CHEST (18) investigators were heavily criticized by HES defenders due to changes in their methods, statistical analysis, and data after publication in 2012 as well as refusal to share their raw data for independent reanalysis (69, 70). A reanalysis of the CHEST (18) trial was ultimately published in 2016 (confirming the conclusion of the original article), but only two of the eight authors of this reanalysis were from an independent institution. All other listed authors came from the institution which conducted the original study, including three authors who were part of the original 2012 publication, one of which was the prior study's principal investigator (71).

Based on the 2013 PRAC recommendation for the MAHs to conduct human RCTs, Fresenius Kabi and B. Braun Melsungen AG, together with the European Society of Anesthesiology, launched two RCTs in 2017 (72, 73). The *Safety and Efficacy of a 6% Hydroxyethyl Starch Solution vs. an Electrolyte Solution in Trauma Patients* (TETHYS) trial will include up to 350 patients with blunt or penetrating trauma suffering from an estimated blood loss of ≥ 500 ml and subsequent hypovolemia, who are undergoing surgery within 24 h. The primary endpoint is 90-day mortality and 90-day renal failure (defined as biomarker increase as defined by AKIN stage 2 or RIFLE injury stage or need for RRT at any time during the first 3 months) (73). The *Safety and Efficacy of 6% Hydroxyethyl Starch Solution vs. an Electrolyte Solution in Patients Undergoing Elective Abdominal Surgery* (PHOENICS) trial will include up to 2,280 patients undergoing elective abdominal surgery with an expected blood loss of ≥ 500 ml and subsequent hypovolemia. Primary outcome is the difference in glomerular filtration rate between the two treatment groups (72).

The regulatory changes and controversy regarding HES led to a worldwide decrease in synthetic colloid use in human ICUs between 2007 and 2014, with considerable geographic variations and an increase in the use of human albumin instead (74).

## The History of HES Restrictions—Second Episode 2018

In February 2017, the US-based Public Citizen Foundation sent a petition to the FDA requesting the immediate removal of all HES products from the market in the US (75). The FDA has yet to undertake official action. The Public Citizen Foundation also joined other experts in signing an open letter to the EMA urging the executive director to reconsider the 2013 PRAC decision and ban the use of HES products for all patients in Europe (Data Sheet 4) (76). In addition, in October 2017, the Swedish Medical Products Agency requested a review of HES products and considered its suspension due to concerns regarding non-compliance to restrictions in their use (Data Sheet 3) (77). Two survey-based drug utilization studies conducted in 2016 and 2017, involving 11 European countries, showed non-adherence to the 2013 PRAC restrictions of up to 77% (77). They showed that HES solutions have continued to be used in high-risk populations (up to 34% of patients) (Data Sheet 5) (78, 79).

In October 2017, the EMA started a new review of HES under Article 107i of Directive 2001/83/EC (35) (Table 1). Upon request from the PRAC, the EMA convened an *ad-hoc* expert group meeting in December 2017 [minutes of this meeting are not publicly available (80)]. In January 2018, the PRAC recommended removing all HES products from the European market (36) (Table 1). The recommendation was endorsed by the CMDh (37) and forwarded to the European Commission (Table 1). Several experts worldwide supported this decision by publishing open letters and comments in scientific journals (81, 82). A group of British, Australian, Danish, and German scientists [some of them 6S (17) or CHEST (18) trial investigators] even appealed to the World Health Organization (WHO), demanding a worldwide ban of HES (82). In contrast, a group of European scientists [some of them CRISTAL (52) trial investigators] responded that the recommendation to suspend HES was “not scientifically grounded and is potentially hazardous to patients” (83). In addition, experts who were part of the EMA *ad-hoc* expert group from December 2017 complained that part of their recommendation not to suspend HES was left out in the official PRAC recommendation on the EMA website (80, 84). Criticism regarding the PRAC recommendation also came from a group of 19 European anesthesia societies in the form of an open letter to the European Commission (85). In this letter, they urged the European Commission to denounce the suspension of HES products. They criticized the validity of the survey-based drug utilization studies that showed non-compliance of PRAC recommendations, because respondents could only select dehydration or overhydration as a reason for administering HES (no check box for hypovolemia was provided). Moreover, it was unclear as to whether or not some septic patients had received HES before the development of sepsis. It was also criticized that suspension of HES would lead to unmet clinical needs for colloids in specific situations, such as plasmapheresis, pediatric cardiac surgery, and prevention of hypotension in patients undergoing cesarean section with spinal anesthesia. They claimed that if HES were to be suspended, there would be no alternative colloid, as dextran, gelatin, or albumin are not superior to HES. Finally, the experts also strongly recommended that HES should not be suspended before the results of the PHOENICS (72) and TETHYS (73) trials become available (84). Not surprisingly, European HES solution manufacturers also claimed that HES should remain available on the market, stating that the off-label use of a product by clinicians is not a sufficient argument to withdraw it from the market (86, 87). Accordingly, the new PRAC recommendation and CMDh position were referred back to the EMA by the European Commission in April 2018, as the concern regarding unmet medical needs (e.g., no safe alternative to HES solutions) and the feasibility and effectiveness of risk minimization measures (e.g., changes to the product information, direct health care professional communication) had not been adequately addressed (38) (Table 1).

In May 2018, after re-assessing data on these specific aspects, the PRAC confirmed its previous recommendation that HES solutions should be suspended and sent it again to the CMDh for consideration (39) (Table 1). For the re-assessment, the PRAC reviewed “all newly available data since the previous referral procedures, including results from drug utilization studies, clinical studies, meta-analyses of clinical studies, post-marketing experience, EudraVigilance data (adverse reactions reporting system), literature reviews, responses submitted by MAHs as well as stakeholders' submissions (e.g., different European anesthesia societies), and views expressed by experts during an *ad-hoc* experts meeting” (40). In regard to the efficacy of HES, the PRAC concluded that although a volume-sparing effect in patients undergoing surgery was demonstrated in some studies, it remained uncertain to what extent this leads to improved postoperative outcomes (88–90). In regard to safety in septic, critically ill, and surgical patients, the quality of new studies were deemed to be insufficient to change the existing restriction (40). In fact, since 2013, some *post-hoc* analyses of the 6S (17) trial have been published, evaluating AKI in the first 5 days (results in favor of crystalloids) (91), the risk of bleeding and death (results in favor of crystalloids) (92), cytokine concentrations (results reveal no difference between crystalloids and HES) (93), and endothelial damage (results in favor of HES) (94). However, there have been no large RCTs performed, comparable with the previous landmark trials VISEP (16), 6S (17), and CHEST (18). In summary, the PRAC concluded that the volume-sparing effect of HES in patients with hypovolemia is only modest, the key results of the two drug utilization studies were reliable, and that non-adherence to the revised product information was high. Accordingly, the PRAC stated that the benefits of HES solutions did not outweigh the risks, its continued use raised important public health concerns, and current risk minimization measures (e.g., information on the package insert) were inadequate (40).

This time, the CMDh disagreed with the conclusions of the PRAC and decided that HES products should not be withdrawn from the market in June 2018 (41) (Table 1). The rationale for this dissenting decision was the potential unmet medical needs in some of the EU member states, in which HES alternatives may be of limited availability or very expensive. In addition, measures to minimize risk were to be implemented, including contraindications (Table 2), limitations on supply to accredited hospitals, training of healthcare professionals, and additional packaging warnings (42). Like the 2013 sanctions, the PRAC recommended that HES use should be limited to initial volume resuscitation with a dose not exceeding 30 ml/kg over a period of administration not exceeding 24 h, and that kidney function should be monitored for at least 90 days thereafter (Data sheet 3) (42).

At the time of writing this review and to the authors' knowledge, no new regulations beyond the 2013/2014 restrictions have been published from non-EU countries, such as Canada, US, Australia, New Zealand, and Switzerland.

## Hydroxyethyl Starch in Veterinary Medicine

Synthetic colloids are widely used in veterinary medicine with HES being the most frequently used according to a recent international internet-based survey in small animals from 2016 (4) and a previous Veterinary Information Network-based survey from 2013 (95). According to the survey from 2016 (4), HES was selected as the most frequently used synthetic colloid by 84%, gelatin by 4.3%, and dextran by 2.7% of the survey participants. Several review articles critically highlighting the expected effects (e.g., volume effect, increase in colloid-osmotic pressure, plugging endothelial leaks), limitations, and side effects of synthetic colloids, and HES in particular, were recently published (10, 96–99). However, no official guidelines from veterinary experts on the use of HES have thus far been provided. The gaps in species-specific HES-related evidence in veterinary medicine has led to extrapolation of previous indications from human medicine with some exceptions. Notably, many veterinarians seem to use HES as a constant rate infusion for colloid osmotic support, which is uncommon in human medicine (4, 8). Not surprisingly, the changes in human recommendations impacted veterinary medicine. Reduction of frequency, dose, and length of administration of HES products was noted in the 2016 survey (4), with 71% of participants having changed their use of HES due to safety concerns. Of these, AKI and coagulopathy were most often considered contraindications for HES (4). Participants who completely stopped using HES, replaced it with isotonic crystalloids in 85%, plasma in 63%, hypertonic saline in 57%, albumin in 28%, and other/unspecified in 3% of respondents. About half of these participants reported using vasopressors more frequently (4).

The evidence regarding the risk of HES-induced AKI in dogs and cats remains controversial. Four retrospective studies found no increased risk of AKI after 6% HES 130/0.4 (tetrastarch) administration in dogs and cats (100–103), and one retrospective study found evidence for an increased risk of AKI and mortality in dogs receiving 10% HES 200/0.5 (pentastarch) (104). Reasons for these discrepant findings may lie in different types of HES products used (older-generation pentastarch in the study that found increased incidence of AKI vs. newer generation tetrastarch in the other four studies), definitions of AKI, co-morbidities, severity of illness scores and dose regimens. Prospective RCTs evaluating HES-induced AKI in critically ill and/or anesthetized dogs or cats are still lacking. Only one recent study in a small population of dogs undergoing emergency abdominal surgery reported increased levels of urinary neutrophil gelatinase-associated lipocalin (NGAL) after 6% tetrastarch administration compared to the use of an isotonic crystalloid after surgery (105). Notably, this study was not designed to compare AKI incidence after HES vs. crystalloid use, fluid administration was not randomized, and therefore results should be interpreted with caution. In a prospective crossover study in healthy, anesthetized dogs with acute controlled hemorrhage, resuscitation with 20 ml/kg 6% tetrastarch did not reveal evidence of AKI (assessed by urine and plasma NGAL and creatinine) for up to 72 h after its administration (106). Likewise, a recent abstract presented a controlled hemorrhagic shock model in anesthetized dogs treated with a bolus of either 6% tetrastarch, 4% succinylated gelatine, fresh whole blood, or an isotonic crystalloid, HES did not lead to a greater incidence of AKI (assessed by different renal biomarkers, e.g., urinary NGAL, and renal histopathology) compared to the other resuscitations fluids (107). Furthermore, a 72-h infusion of 6% tetrastarch at 50 ml/kg per 24 h did not significantly impact renal function (assessed by urinary NGAL and renal histopathology) in healthy dogs as shown in a recent study presented in an abstract (108). The small study populations and the studies' experimental nature, not representative of the typical cohort of critically ill patients, needs to be taken in consideration before clinical decisions can be based on these results. Furthermore, scientific abstracts often present only partial information and are not submitted to the full scrutiny of a peer-reviewed process reviewing the entire dataset, methods, and limitations of the respective study.

A fair amount of *in vitro* and *in vivo* studies evaluating the effects of 6% tetrastarch on hemostasis exists in dogs (8, 109–116), but only one *in vitro* study in cats (117). In dogs administered 6% tetrastarch, a dose-dependent impairment of platelet function and changes in viscoelastic coagulation testing (rotational thromboelastometry [ROTEM] or thromboelastography) in healthy dogs (109, 110, 113, 114), dogs with controlled hemorrhagic shock (112, 116), and dogs with sepsis was found (111). However, no difference in platelet function was found between 20 ml/kg of 6% tetrastarch and a 3- to 4-fold volume of 0.9% NaCl in healthy dogs (*in vitro*) (114) or in dogs with controlled hemorrhagic shock (*in vivo*) (112). These findings suggest that 6% tetrastarch does not cause platelet dysfunction beyond the effects of hemodilution alone. In a similar experimental model in dogs with controlled hemorrhagic shock, dogs received 20 ml/kg of 6% tetrastarch, 4% succinylated gelatine, fresh whole blood, or 80 ml/kg of an isotonic crystalloid solution. Plasmatic coagulation testing and ROTEM showed evidence of mild hypocoagulability beyond hemodilution after HES and gelatin administration, with gelatin administration leading to impaired platelet function and HES administration causing hypocoagulable ROTEM and plasma coagulation assays (116). In dogs with naturally-occurring hemorrhagic shock due to spontaneous hemoperitoneum receiving boluses of either 10 ml/kg of 6% tetrastarch or 30 ml/kg of isotonic crystalloid, an exacerbation of the pre-existing coagulopathy was found after both solutions in plasma coagulation and ROTEM assays, with more pronounced effects on ROTEM after HES (118). Therefore, HES should be avoided or used with caution in dogs at risk for hemorrhage or with pre-existing coagulopathy.

## Alternatives to HES Solutions

The impact HES restrictions will have on veterinary practice in Europe and elsewhere is uncertain and alternative products will have to be considered. Potential available replacements for HES in people [that have been said to be safer but just as effective, as stated e.g., by a regulatory citizen petition (75)], are gelatin solutions (Europe), dextran (US), albumin solutions, and isotonic crystalloids. A detailed discussion of the benefits and risks of each of these products is beyond the scope of this review. However, significantly less evidence exists in the small animal literature about the efficacy and safety of gelatin and dextran compared to HES.

Gelatin solutions are derived from the degradation of bovine collagen with subsequent chemical modifications to increase solubility (119). Due to their smaller molecular weight compared to HES, gelatins are rapidly excreted by the kidneys and provide a shorter and less pronounced intravascular volume effect (10). Similar to HES, gelatin solutions were introduced into clinical practice in the 1960s before current information requirements for licensing and extensive safety studies were mandatory (119). A meta-analysis in humans concluded that the safety and efficacy of gelatins cannot be assessed based on available evidence despite 60 years of use (120). Gelatin solutions increase the risk of anaphylaxis and may further be harmful by increasing mortality through renal failure and bleeding due to extravascular uptake and coagulation impairment, respectively (121). In a recent prospective study in people with severe sepsis, AKI occurred in 70% of patients receiving HES and in 68% of patients receiving gelatin vs. 47% patients receiving crystalloids. Moreover, in the same study, fluid resuscitation with only crystalloids was equally effective (122). Gelatin has been investigated much less in dogs or cats than HES. Only a few and mostly experimental studies (not related to the risk of AKI) on the use of gelatins in dogs and cats have been published (123–129). In a recent abstract, a greater incidence of kidney injury after the administration of a 4% gelatin solution compared to the administration of 6% tetrastarch, fresh whole blood, or an isotonic crystalloid in a controlled hemorrhagic shock model in anesthetized dogs was reported (107). In this study, a variety of urinary biomarkers as well as renal histopathology were evaluated, and dogs given gelatin had significantly increased NGAL concentrations (with up to a 23-fold increase) within 3 h and increased cystatin C levels, compared to other treatments. Tubular injury scores assessed by histopathology were comparable across treatments, while microvesiculation (intracellular storage of colloid molecules) was significantly higher in the gelatin group (107). Notably, gelatin was withdrawn from the US market in 1978 due to safety concerns over increased blood viscosity and blood coagulation (130). In other countries (e.g., Germany), it is licensed and despite evidence of AKI after intravenous gelatin administration in people (131), it is not labeled to be contraindicated in kidney disease. Indeed, there is no clear dose or maximum daily limit, and the recommendation on the package insert states that the “maximum daily dose is determined by the degree of hemodilution” (Data Sheet 6) (132, 133).

Dextran, a macromolecular polysaccharide, has been withdrawn from the market in a number of European countries (e.g., Germany) due to its adverse effects, such as anaphylactic reactions, osmotic kidney failure with hypertonic dextran preparations, and impaired coagulation (2). By contrast, dextran is licensed in the US, Russia, China, some eastern European countries, and Scandinavia (e.g., Sweden, Norway) (2). In dogs, dextran and hypertonic dextran have been studied in septic shock secondary to pyometra (134), hemorrhagic shock (135, 136), and gastric dilatation volvulus (137, 138). In addition, investigations of its effects on hemostasis are available (139, 140). Evaluation of the effects of oxypolygelatin and dextran 70 on hemostatic variables in healthy dogs showed that both dextran and oxypolygelatin interfered with hemostatic variables (e.g., plasma coagulation assays, platelet numbers, factor VIII coagulant activity, von Willebrand factor antigen concentration, and platelet function and buccal mucosal bleeding time), but dextran's effect was more profound and prolonged when compared to oxypolygelatin (140).

Canine albumin (e.g., lyophilized canine albumin) manufactured in the US, is currently shipped only to Hong Kong, Taiwan, Singapore, and Canada. Consequently, other countries are forced to use human serum albumin products if they wish to administer albumin to their canine or feline patients. No feline albumin products are currently commercially available. Clear evidence of anaphylactic reactions and life-threatening complications after administration of human serum albumin were reported in dogs (141, 142), although large retrospective studies have not demonstrated a high complication rate with human serum albumin solutions use in critically ill dogs and cats (143, 144).

## Conclusion

Despite two PRAC expert committees (in 2013 and 2018) recommending suspension of the use of HES and after several reviews and intense discussions, the European Commission decided against a total suspension of HES in July of 2018. Instead of a suspension, additional restrictions have been implemented, allowing its continued use as long as access is controlled and warnings on the packaging inserts are clearly stated. Most likely, this is not the final episode of the HES “saga” as large human RCTs are currently ongoing. In spite of, or maybe because of the HES-controversy, HES is currently the most studied synthetic colloid in human and veterinary medicine. The complicated process of these safety reviews illustrates the difficulties in decision making regarding such a wide-spread drug, particularly when multiple countries with different claims are involved. In spite of the seeming transparency of regulatory authorities in publicly disclosing data and regulatory documents, the exact methods used in their risk assessment procedures remain convoluted and unclear. This was probably one of the main reasons for a remarkable “flood” of commentaries and editorial letters from different experts with strongly held opinions, further fueling the controversy. This situation also reflects the challenges in designing and implementing clinical trials and appropriately interpreting large amounts of data. In such instances, where substantial controversy exists in a field, it is critical that original data from pivotal trials (such as colloid 6S and CHEST) be made available for independent (re-) analysis (145). Therefore, the reluctance of the authors to share original data further exacerbated the debate (69). Examining the available literature leaves the authors of this review with the impression that HES safety and efficacy has become a matter of opinion rather than evidence. Despite efforts to apply evidence-based medicine, grounded guidelines for HES-related clinical decision making are lacking. If and how the 2018 changes may impact the availability and use of HES in veterinary medicine remains unclear. Moreover, if clinicians seek alternatives to HES, the paucity of evidence for their safety and efficacy in veterinary medicine should also be carefully considered.

## Author Contributions

K-NA and IY performed the literature search and wrote the manuscript.

### Conflict of Interest Statement

The authors declare that the research was conducted in the absence of any commercial or financial relationships that could be construed as a potential conflict of interest.

## Abbreviations

6S: Scandinavian Starch for Severe Sepsis/Septic Shock
AKI: acute kidney injury
CHEST: Crystalloid vs. Hydroxyethyl Starch Trial
CHRYSTMAS: Crystalloids Morbidity Associated with severe Sepsis
CMDh: Coordination Group for Mutual Recognition and Decentralized Procedures—Human
EMA: European Medicines Agency
EU: European Union
FDA: Food and Drug Administration
HES: hydroxyethyl starch
ICU: intensive care unit
MAH: marketing authorization holders
NGAL: neutrophil gelatinase-associated lipocalin
PRAC: Pharmacovigilance Risk Assessment Committee
RCT: randomized clinical trial
RRT: renal-replacement therapy
US: United States
VISEP: Volume Substitution and Insulin Therapy in Severe Sepsis.
