# Supplementary material for: Starch Wars—New Episodes of the Saga. Changes in Regulations on Hydroxyethyl Starch in the European Union
Source: Front Vet Sci. 2019 Jan 18;5:336. doi: 10.3389/fvets.2018.00336 (PMC6345713; doi:10.3389/fvets.2018.00336)
Supplement: Supplementary file 1 [file Data_Sheet_1.PDF]

Appendix 1 – Available online sources for the HES restrictions 2013 used in the article

Index

1. 11/29/2012: Review of hydroxyethyl-starch-containing solutions for infusion started
2. 06/14/2013: PRAC recommends suspending marketing authorizations for infusion solutions containing hydroxyethyl-starch
3. Drug alert UK_27.June 2013
4. 07/12/2013: New review of hydroxyethyl starch-containing solutions for infusion started under Article 107i
5. 07/12/2013: Recommendation to suspend marketing authorizations for HES to be re-examined under Article 31
6. 11/11/2013: Assessment report for solutions for infusion containing hydroxyethyl starch. Procedure under Article 31
7. 11/11/2013: Assessment report for solutions for infusion containing hydroxyethyl starch. Procedure under Article 31
8. 2013: Annex III CMDh position 2013 hydroxyethyl-starch-article-107i-procedure. Amendments to relevant sections of the summary of product characteristics and package leaflet
9. 10/11/2013: PRAC confirms that HES should no longer be used in patients with sepsis or burn injuries or in critically ill
10. 10/25/2013: HES should no longer be used in sepsis, burn injuries, in critically ill patients – CMDh endorse
11. 12/19/2013: HES no longer to be used in patients with sepsis or burn injuries or in critically ill patients.

29 November 2012
EMA/757392/2012
EMEA/H/A-31/1348

Review of hydroxyethyl-starch-containing solutions for infusion started

The European Medicines Agency has started a review of solutions containing hydroxyethyl starch (HES) used for the management of hypovolaemia (low blood volume caused by dehydration or blood loss) and hypovolaemic shock (a steep fall in blood pressure caused by drop in blood volume) in critically ill patients and, in particular, patients with sepsis (damage to organs caused by bacteria and their toxins in the blood following an infection).

HES-containing solutions are given by infusion (drip) into a vein and are used as volume expanders to replace lost fluids in patients with hypovolaemia to prevent shock.

Safety concerns have been raised following the publication of recent studies comparing HES with other volume expanders in critically ill patients. A study[1] comparing HES with Ringer's acetate (another volume expander) in patients with severe sepsis showed that patients treated with HES had a higher risk of death and were more likely to receive renal replacement therapy (treatment for kidney failure such as dialysis). These results were similar to those of an earlier study[2] in patients with severe sepsis. In addition, a more recent study[3] carried out in 7,000 intensive care patients comparing HES with saline solution also showed a higher need for renal replacement therapy but no increased risk of death in patients treated with HES.

The European Medicines Agency will evaluate the benefit-risk balance of HES-containing solutions for infusion and issue an opinion on whether their marketing authorisations should be maintained, varied, suspended or withdrawn across the EU.

[1] Perner, A. *et al.* Hydroxyethyl Starch 130/0.42 versus Ringer's acetate in severe sepsis. N Engl J Med 2012; 367(2):124-134.
[2] Brunkhorst, F.M. *et al.* Intensive insulin therapy and pentastarch resuscitation in severe sepsis. N Engl J Med, 2008; 358(2):125-39.
[3] Myburgh, J.A. *et al.* Hydroxyethyl starch or saline for fluid resuscitation in intensive care; N Engl J Med 2012; 367(20):1901-11.

7 Westferry Circus ● Canary Wharf ● London E14 4HB ● United Kingdom
Telephone +44 (0)20 7418 8400 **Facsimile** +44 (0)20 7523 7129
E-mail info@ema.europa.eu **Website** www.ema.europa.eu
An agency of the European Union

© European Medicines Agency, 2012. Reproduction is authorised provided the source is acknowledged.

More about the medicine

Infusion solutions containing HES are frequently used volume expanders and belong to the class of colloids. There are two main types of volume expanders: crystalloids and colloids. Colloids contain large molecules such as starch whereas crystalloids such as saline solutions contain smaller molecules. In the EU, HES-containing solutions for infusion have been approved via national procedures.

More about the procedure

The review of HES solutions for infusion has been initiated at the request of the German medicines agency under Article 31 of Directive 2001/83/EC.

The review is being carried out by the Pharmacovigilance Risk Assessment Committee (PRAC), the Committee responsible for the evaluation of safety issues for human medicines, which will make a set of recommendations. As HES-containing medicines are all authorised nationally, the PRAC recommendation will be forwarded to the Co-ordination Group for Mutual Recognition and Decentralised Procedures – Human (CMDh), which is a regulatory body that represents national medicines regulatory authorities of the EU Member States. This will results in harmonised measures to be implemented in all Member States.

14 June 2013
EMA/349341/2013

PRAC recommends suspending marketing authorisations for infusion solutions containing hydroxyethyl-starch

The European Medicines Agency's Pharmacovigilance Risk Assessment Committee (PRAC) has concluded following a review of the available evidence that the benefits of infusion solutions containing hydroxyethyl-starch (HES) no longer outweigh their risks and therefore recommended that the marketing authorisations for these medicines be suspended.

Infusion solutions containing HES are medicines mainly used to replace lost blood volume in hypovolaemia (low blood volume caused by dehydration or blood loss) and hypovolaemic shock (a steep fall in blood pressure caused by drop in blood volume). They are used in critically ill patients including patients with sepsis (bacterial infection of the blood) or burn or trauma injuries, or patients who are undergoing surgery.

The review of infusion solutions containing HES was triggered by the German medicines agency, the Federal Institute for Drugs and Medical Devices (BfArM), following three recent studies[1,2,3] that compared HES with other products used for volume replacement called crystalloids in critically ill patients. The studies showed that patients with severe sepsis treated with HES were at a greater risk of kidney injury requiring dialysis. Two of the studies[1,2] also showed that in patients treated with HES there was a greater risk of mortality. The PRAC was therefore requested to assess the available evidence and how it impacts on the risk-benefit balance of HES infusion solutions in the management of hypovolaemia and hypovolaemic shock.

The PRAC assessed data from the scientific literature and the data submitted by the companies, and took advice from a group of external experts. The PRAC was of the opinion that, when compared with crystalloids, patients treated with HES were at a greater risk of kidney injury requiring dialysis and had a greater risk of mortality. The PRAC also considered that the available data only showed a limited benefit of HES in hypovolaemia, which did not justify its use considering the known risks. The PRAC therefore concluded that the marketing authorisations for these medicines be suspended.

[1] Perner, A. *et al.* Hydroxyethyl Starch 130/0.42 versus Ringer's acetate in severe sepsis. N Engl J Med 2012; 367(2):124-134.
[2] Brunkhorst, F.M. *et al.* Intensive insulin therapy and pentastarch resuscitation in severe sepsis. N Engl J Med, 2008; 358(2):125-39.
[3] Myburgh, J.A. *et al.* Hydroxyethyl starch or saline for fluid resuscitation in intensive care; N Engl J Med 2012; 367(20):1901-11.

7 Westferry Circus ● Canary Wharf ● London E14 4HB ● United Kingdom
Telephone +44 (0)20 7418 8400 **Facsimile** +44 (0)20 7523 7129
E-mail info@ema.europa.eu **Website** www.ema.europa.eu

© European Medicines Agency, 2013. Reproduction is authorised provided the source is acknowledged.

The suspension should remain in place unless the marketing authorisation holder can provide convincing data to identify a group of patients in whom the benefits of the medicines outweigh their risks.

The PRAC recommendation will be considered by the Coordination Group for Mutual Recognition and Decentralised Procedures – Human (CMDh).

More about the medicine

Infusion solutions containing HES are frequently used for volume replacement and belong to the class known as colloids. There are two main types of medicines used for volume replacement: crystalloids and colloids. Colloids contain large molecules such as starch, whereas crystalloids, such as saline (salt) solutions or Ringer acetate, contain smaller molecules. In the European Union (EU), HES-containing solutions for infusion have been approved via national procedures and are available in all Member States under various trade names.

More about the procedure

The review of HES solutions for infusion was initiated on 29 November 2012 at the request of the German medicines agency, under Article 31 of Directive 2001/83/EC.

The review has been carried out by the Pharmacovigilance Risk Assessment Committee (PRAC), the Committee responsible for the evaluation of safety issues for human medicines, which has made a set of recommendations. The marketing authorisation holders may request a re-examination within 15 days of being notified of the PRAC recommendation.

As these medicines are all authorised nationally, the PRAC recommendation will now be forwarded to the Co-ordination Group for Mutual Recognition and Decentralised Procedures – Human (CMDh), which will adopt a final position. The CMDh is a medicines regulatory body representing the EU Member States.

If the CMDh position is agreed by consensus, the agreement will be directly implemented by the Member States where the medicines are authorised. Should the CMDh position be adopted by majority vote, the CMDh position will be sent to the European Commission, for the adoption of an EU-wide legally binding decision.

Contact our press officers

Monika Benstetter or Martin Harvey

Tel. +44 (0)20 7418 8427

E-mail: press@ema.europa.eu

DRUG ALERT

CLASS 2 MEDICINES RECALL

Action Within 48 Hours
Pharmacy, Clinic and Wholesaler Level Recall

Date: 27 June 2013   EL (13)A/18   Our Ref: MDR 22-06/13

Dear Healthcare Professional,

B Braun Melsungen AG

Hydroxyethyl Starch (HES) products

**Tetraspan 10% solution for infusion (500ml)**	**PL 03551/0107**
**Tetraspan 6% solution for infusion (500ml)**	**PL 03551/0106**
**Venofundin 60mg/ml solution for infusion (500ml)**	**PL 03551/0097**

Fresenius Kabi Limited

Hydroxyethyl Starch (HES) products

**Voluven 10% solution for infusion (500ml)**	**PL 08828/0207**
**Voluven 6% solution for infusion (500ml)**	**PL 08828/0145**
**Volulyte 6% solution for infusion (500ml)**	**PL 08828/0174**

All unexpired stock of these products is being recalled to pharmacy, clinic and wholesaler level irrespective of batch number and expiry date.

These products are being withdrawn after results from large randomised clinical trials have reported an increased risk of renal dysfunction and mortality in critically ill or septic patients who received hydroxyethyl starch (HES) compared with crystalloids (simple salt solutions). The UK Commission on Human Medicines (CHM) has concluded that the benefits no longer outweigh the risks.

No further hydroxyethyl starch (HES) products should be dispensed. An alternative resuscitation fluid should be selected according to clinical guidelines.

Remaining stocks of the affected batches should be quarantined and returned to the original supplier for credit. For enquiries relating to stock returns please contact:

B Braun Medical Limited (UK) on 01142 259 155
Fresenius Kabi Limited Customer Services on 01928 533 697

Contd/…

For medical information enquiries please contact:

B Braun Medical Limited (UK) Medical Information on 01142 259 159
Fresenius Kabi Limited Medical Information on 01928 533 612 or email pharmacovigilance.GB@fresenius-kabi.com.

Recipients of this Drug Alert should bring it to the attention of relevant contacts by copy of this letter. Local area teams are asked to forward this to relevant clinics, general practitioners and community pharmacists for information.

Yours faithfully

Adam Burgess
Defective Medicines Report Centre
151 Buckingham Palace Road
London
SW1W 9SZ
Telephone +44 (0)20 3080 6574

12 July 2013
EMA/403074/2013

New review of hydroxyethyl starch-containing solutions for infusion started

The European Medicines Agency has started a new review of hydroxyethyl starch (HES)-containing solutions for infusion, following the suspension of the use of these medicines in the UK on 27 June 2013.

The EMA's Pharmacovigilance Risk Assessment Committee (PRAC) had recommended in June 2013 that these medicines be suspended in the EU, following an assessment of available data which concluded that their benefits do not outweigh the risks of kidney injury and mortality. However, the process to implement the PRAC's recommendation across the EU has not yet begun since a number of marketing authorisation holders exercised their legal right to request a re-examination of the recommendation.

In the meantime, some Member States have taken action to suspend or limit the marketing or use of these medicines in their territories. In accordance with EU legislation this type of action currently requires that a review procedure be carried out. Consequently, the United Kingdom has requested the PRAC to start this review procedure, which will run in parallel with the re-examination of the PRAC's June 2013 recommendation.

The Agency invites all stakeholders (e.g. healthcare professionals, patients' organisations, the general public) to submit data relevant to this procedure. Full details are available under the 'data submission' tab.

More about the medicine

HES solutions are volume expanders used to replace lost blood volume in hypovolaemia (low blood volume caused by dehydration or blood loss) and hypovolaemic shock (a steep fall in blood pressure caused by drop in blood volume). They are used in critically ill patients including patients with sepsis (bacterial infection of the blood) or burn or trauma injuries, or patients who are undergoing surgery. HES solutions are given by infusion (drip) into a vein.

7 Westferry Circus ● Canary Wharf ● London E14 4HB ● United Kingdom
Telephone +44 (0)20 7418 8400 **Facsimile** +44 (0)20 7523 7129
E-mail info@ema.europa.eu **Website** www.ema.europa.eu
An agency of the European Union

© European Medicines Agency, 2013. Reproduction is authorised provided the source is acknowledged.

Infusion solutions containing HES belong to the class of colloids. There are two main types of volume expanders: crystalloids and colloids. Colloids contain large molecules such as starch, whereas crystalloids such as saline solutions contain smaller molecules. In the EU, HES-containing solutions for infusion have been approved via national procedures.

More about the procedure

This review of HES solutions for infusion has been initiated at the request of the UK medicines agency, MHRA, under Article 107i of Directive 2001/83/EC, also known as the urgent Union procedure.

The review is being carried out by the Pharmacovigilance Risk Assessment Committee (PRAC), the Committee responsible for the evaluation of safety issues for human medicines, which will make a set of recommendations. As these medicines are all authorised nationally, the PRAC recommendation will be forwarded to the Co-ordination Group for Mutual Recognition and Decentralised Procedures – Human (CMDh), which will adopt a final position. The CMDh is a regulatory body that represents the EU Member States, responsible for ensuring harmonised safety standards for medicines authorised via national procedures across the EU.

In June 2013, the PRAC had adopted recommendations on HES solutions under Article 31 of Directive 2001/83/EC. A number of marketing authorisation holders have requested a re-examination of the recommendations. More information about this can be found here.

EUROPEAN MEDICINES AGENCY
SCIENCE MEDICINES HEALTH

12 July 2013
EMA/349341/2013

Recommendation to suspend marketing authorisations for hydroxyethyl-starch solutions to be re-examined

On 13 June 2013, the EMA's Pharmacovigilance Risk Assessment Committee (PRAC) concluded following a review of the available evidence that the benefits of infusion solutions containing hydroxyethyl-starch (HES) no longer outweigh their risks and therefore recommended that the marketing authorisations for these medicines be suspended.

Following the PRAC recommendation, some of the marketing authorisation holders requested a re-examination. Upon receipt and validation of the grounds, the PRAC will re-examine its recommendation and issue a final recommendation.

Infusion solutions containing HES are medicines mainly used to replace lost blood volume in hypovolaemia (low blood volume caused by dehydration or blood loss) and hypovolaemic shock (a steep fall in blood pressure caused by drop in blood volume). They are used in critically ill patients including patients with sepsis (bacterial infection of the blood) or burn or trauma injuries, or patients who are undergoing surgery.

The review of infusion solutions containing HES was triggered by the German medicines agency, the Federal Institute for Drugs and Medical Devices (BfArM), following three recent studies[1,2,3] that compared HES with other products used for volume replacement called crystalloids in critically ill patients. The studies showed that patients with severe sepsis treated with HES were at a greater risk of kidney injury requiring dialysis. Two of the studies[1,2] also showed that in patients treated with HES there was a greater risk of mortality. The PRAC was therefore requested to assess the available evidence and how it impacts on the risk-benefit balance of HES infusion solutions in the management of hypovolaemia and hypovolaemic shock.

The PRAC assessed data from the scientific literature and the data submitted by the companies, and took advice from a group of external experts. The PRAC was of the opinion that, when compared with crystalloids, patients treated with HES were at a greater risk of kidney injury requiring dialysis and had a greater risk of mortality. The PRAC also considered that the available data only showed a limited

[1] Perner, A. *et al.* Hydroxyethyl Starch 130/0.42 versus Ringer's acetate in severe sepsis. N Engl J Med 2012; 367(2):124-134.
[2] Brunkhorst, F.M. *et al.* Intensive insulin therapy and pentastarch resuscitation in severe sepsis. N Engl J Med, 2008; 358(2):125-39.
[3] Myburgh, J.A. *et al.* Hydroxyethyl starch or saline for fluid resuscitation in intensive care; N Engl J Med 2012; 367(20):1901-11.

7 Westferry Circus ● Canary Wharf ● London E14 4HB ● United Kingdom
Telephone +44 (0)20 7418 8400 **Facsimile** +44 (0)20 7523 7129
E-mail info@ema.europa.eu **Website** www.ema.europa.eu

An agency of the European Union

© European Medicines Agency, 2013. Reproduction is authorised provided the source is acknowledged.

benefit of HES in hypovolaemia, which did not justify its use considering the known risks. The PRAC therefore concluded that the marketing authorisations for these medicines be suspended. The Committee considered that the suspension should remain in place unless the marketing authorisation holder can provide convincing data to identify a group of patients in whom the benefits of the medicines outweigh their risks.

The PRAC recommendation will now be re-examined and the outcome will be made public on the EMA website.

More about the medicine

Infusion solutions containing HES are frequently used for volume replacement and belong to the class known as colloids. There are two main types of medicines used for volume replacement: crystalloids and colloids. Colloids contain large molecules such as starch, whereas crystalloids, such as saline (salt) solutions or Ringer acetate, contain smaller molecules. In the European Union (EU), HES-containing solutions for infusion have been approved via national procedures and are available in all Member States under various trade names.

More about the procedure

The review of HES solutions for infusion was initiated on 29 November 2012 at the request of the German medicines agency, under Article 31 of Directive 2001/83/EC.

The review has been carried out by the Pharmacovigilance Risk Assessment Committee (PRAC), the Committee responsible for the evaluation of safety issues for human medicines, which has made a set of recommendations.

As these medicines are all authorised nationally, the final PRAC recommendation following the re-examination will be forwarded to the Co-ordination Group for Mutual Recognition and Decentralised Procedures – Human (CMDh), which will adopt a final position. The CMDh is a medicines regulatory body representing the EU Member States.

If the CMDh position is agreed by consensus, the agreement will be directly implemented by the Member States where the medicines are authorised. Should the CMDh position be adopted by majority vote, the CMDh position will be sent to the European Commission, for the adoption of an EU-wide legally binding decision.

Contact our press officers

Monika Benstetter or Martin Harvey

Tel. +44 (0)20 7418 8427

E-mail: press@ema.europa.eu

11 November 2013
EMA/667674/2013

Assessment report for solutions for infusion containing hydroxyethyl starch

Procedure under Article 31 of Directive 2001/83/EC resulting from pharmacovigilance data

Procedure number: EMEA/H/A-31/1348

Final Assessment Report as adopted by the PRAC with all information of a confidential nature deleted.

7 Westferry Circus ● Canary Wharf ● London E14 4HB ● United Kingdom
Telephone +44 (0)20 7418 8400 **Facsimile** +44 (0)20 7523 7051
E-mail info@ema.europa.eu **Website** www.ema.europa.eu

An agency of the European Union

© European Medicines Agency, 2013. Reproduction is authorised provided the source is acknowledged.

Table of contents

1. Background information on the procedure 3

2. Scientific discussion 3

2.1. Clinical aspects 3

2.1.1. Safety 4

2.1.2. Efficacy 10

3. Overall discussion and risk / benefit assessment.................................... 18

4. Re-examination procedure 18

5. Communication plan 21

6. Conclusion and grounds for the recommendation................................. 21

1. Background information on the procedure

On 20 November 2012, Germany informed the European Medicines Agency, pursuant to Article 31 of Directive 2001/83/EC, of their consideration to review the benefit risk balance of HES solutions for infusion in the treatment and prophylaxis of hypovolemia and hypovolemic shock resulting from the evaluation of data relating to pharmacovigilance showing an increased risk of mortality and renal replacement therapy (RRT) in patients treated with HES.

2. Scientific discussion

Hydroxyethyl starch (HES) solutions for infusion include products with starch derived from potato or corn (waxy maize), with different molecular weights (mainly 130kD; 200kD) and substitution ratios (the number of hydroxyethyl groups per glucose molecule). More than 80% of patients treated with HES receive HES 130kD. HES containing solutions for infusion are authorised worldwide including all EU and EEA countries with the main indication for the treatment and prophylaxis of hypovolaemia and hypovolemic shock.

In the therapy of patients with hypovolemia due to severe sepsis the mainstay of treatment is the application of intravenous fluids. Colloid solutions are used for volume substitution in these patients but there are limited data to support this.

Concerns with regards to HES were previously considered by the Pharmacovigilance Working Party in September 2008 on the basis of results of several studies, some of which showing an increased risk for renal RRT or acute renal failure. Published studies (6S[1], VISEP[2]) including a recent one (6S) provided further data supporting an increased risk of mortality at day 90 and RRT in patients with sepsis. Furthermore, the higher risk for RRT was shown in another recently published large clinical trial for all intensive care unit (ICU) patients (CHEST[3]) supporting the results of the 6S study. Mortality difference was not confirmed, however, the study enrolled a broad mixture of patients with on average lower baseline mortality risk admitted to intensive care units. Although some limitations of the studies were raised, the data which were collected from these large randomised clinical trials were considered solid enough to indicate a potential harm associated with HES.

In view of the above, the PRAC was requested to assess the benefit risk balance of HES containing medicinal products for solutions for infusion in the treatment and prophylaxis of hypovolaemia and hypovolemic shock.

2.1. Clinical aspects

Fluid resuscitation and blood volume substitution are fundamental interventions in many different clinical situations. The need to substitute fluid or blood volume may result from an actual loss of plasma or blood volume, but also from the relative hypovolemia induced by pathological vasodilation, such as in sepsis. A direct loss of blood volume may occur as a consequence of direct injury, such as bleeding during surgery or in trauma, but it may also be a result of an altered capillary permeability with consequent extravasation of plasma volume. This latter situation is encountered in situations with massive inflammatory activation, such as in sepsis and burn injury.

[1] Perner A, Haase N, Guttormsen AB *et al*. Hydroxyethyl starch 130/0.42 versus ringer's acetate in severe sepsis. *N Engl J* 2012 Jul 12;367(2):124-34
[2] Brunkhorst FM, Engel C, Bloos F *et al*. Intensive Insulin Therapy and Pentastarch Resuscitation in Severe Sepsis. *N Engl J Med* 2008; 358(2):125-39
[3] Myburgh J, Finder S, Bellomo R *et al*. Hydroxyethyl starch or saline for fluid resuscitation in intensive care. *N Engl J Med* 2012; 367:1901-11

When crystalloids (saline or balanced salt solutions) are used for blood volume replacement they are distributed across the entire extracellular space, as governed by capillary permeability and the Starling equilibrium. Consequently, in theory, approximately ¾ of a given crystalloid volume is redistributed to the extracellular space, leaving only ¼ as effective blood volume replacement. The expansion of the extracellular space may be seen as an unwanted side effect.

This provides the rationale for blood volume replacement with colloid solutions. Colloids are molecules large enough to mainly remain within the vascular space and through colloid osmotic influence keep the administered fluid volume largely in the intravascular space. A colloid solution can therefore, at least in theory, provide a more rapid blood volume expansion with less volume given and consequently also less unwanted oedema formation. These basic physiological principles for the effects of colloids may, however, be compromised in situations such as sepsis and burn injury, where extensive capillary leakage may lead to increased extravasation of colloids. This makes the actual distribution of colloids difficult to predict, especially in the critically ill patients (Ernest, Belzberg *et al.* 1999[4]; van der Heijden, Verheij *et al.* 2009[5]).

There are three main types of synthetic colloid currently available: gelatins, dextrans and hydroxyethyl starch solutions. HES solutions which are derived of the partial hydrolysis of maize or potato starch, amylopectin, with replacement of the hydroxyl (OH) radicals (present in position C2, C3 and C6) by hydroxyethyl radicals are characterised by four elements: the molecular weight ranging from 70 to 670kDa, the degree of substitution which is usually 0.4 (tetrastarch) to 0.7 (hetastarch), the C2/C6 ratio characterizing the type of substitution (substitution is only possible at level 2, 3 or 6) and the concentration (generally 6% or 10%). Use of HES solutions has been associated with several problems. First, they alter hemostasis in a dose-dependent fashion. These alterations are primarily similar to a von Willebrand type of disease, as for dextrans. Second, HES solutions can persist in the organism sometimes for very prolonged periods of time, especially in the reticuloendothelial system. Third, these solutions may alter renal function, possibly as the result of the development of osmotic-nephrosis-like damage.

The PRAC considered all available data which includes recently published large clinical trials in critically ill patients and patients with sepsis, several meta-analyses as well as results of two unpublished clinical trials which compared HES with crystalloids and other colloids. The PRAC also consulted the views of experts through an Ad-Hoc Expert meeting.

2.1.1. Safety

Based on data from clinical studies, HES when compared to crystalloids was shown to be associated with an increased risk of mortality in patients with severe sepsis and adverse renal effects in particular in critically ill patients. A higher risk for other adverse reactions was also reported. These safety concerns which were reported in several clinical studies as well as meta-analyses are presented below.

Risk of mortality

Treatment with HES in critically ill patients has been associated with increased risk of mortality at day 90 in two large randomised clinical trials (including a recent published one) in patients with sepsis and septic shock (6S, VISEP).

The 6S study which was a randomised, multicentre, parallel-group, blinded trial was conducted in 804 patients (798 included in the modified intention-to-treat ITT population). The two intervention groups

[4] Ernest D, Belzberg AS *et al*. Distribution of normal saline and 5% albumin infusions in septic patients. Crit Care Med 1999; 27(1): 46-50
[5] van der Heijden M, Verheij J *et al*. Crystalloid or colloid fluid loading and pulmonary permeability, edema, and injury in septic and nonseptic critically ill patients with hypovolemia. Crit Care Med 2009; 37(4): 1275-81

had similar baseline characteristics. At day 90 after randomisation, 201 of 398 patients (51%) assigned to HES 130/0.42 had died, as compared with 172 of 400 patients (43%) assigned to Ringer's acetate (relative risk(RR), 1.17; 95% confidence interval [CI], 1.01 to 1.36; P = 0.03); 1 patient in each group had end-stage kidney failure.

In the randomised, multicentre, two-by-two factorial trial including 600 patients (VISEP Study), the rate of death at 28 days did not differ significantly between the HES group and the Ringer's lactate group (26.7% and 24.1%, respectively; P=0.48). However, mortality at day 90 was significantly increased in patients who received higher dose of HES 205/0.5 (>22 ml/kg bodyweight per day) compared to the Ringer's lactate group (41.0% vs 33.9%, P=0.09).

These findings were confirmed by recent meta-analyses (Zarychanski et al. 2013[6]; Cochrane review 2013[7]).

The meta-analysis (Zarychanski et al. 2013) included 38 trials with 10,880 critically ill patients and compared HES with crystalloids, albumins or gelatine. When 7 trials were excluded from an investigator whose subsequent research had been retracted because of scientific misconduct, HES was found to be associated with increased risk of mortality among 10290 patients (RR: 1.09; 95% CI): 1.02-1.17; (heterogeneity) I^2 0%). A subgroup analysis of 12 randomised clinical studies that used 6% HES 130/0.4 formulations only, confirmed the increased risk of mortality in patients treated with HES.

In the Cochrane review, a 10 % higher mortality rate was shown for patients who received HES (RR: 1.10; 95% CI 1.02 - 1.19). It should be noted that two studies contributed to 80% of the weight in the meta-analyses (CHEST study Myburgh *et al*. 2012; 6S Study Perner *et al*. 2012) which were adequately powered and blinded, multicentre studies.

Adverse renal effects

The potential mechanism behind adverse renal effects associated with HES includes an increased uptake of starch into the renal epithelial cells inducing osmotic nephrosis, tubular obstructions by hyperviscous urine, and renal inflammation (Claus RA *et al*. 2010[8]). However, the potential mechanism is not fully elucidated. Adverse renal effects of HES, independent of the molecular weight or other differences in the product composition were reported in several clinical studies.

<u>Safety data from clinical trials</u>

- VISEP study (Brunkhorst FM *et al*. 2008)

The VISEP study was conducted as a multicentre, two-by-two factorial trial, in 600 patients (537 included for ITT analysis) with severe sepsis randomised to receive either intensive insulin therapy to maintain euglycemia or conventional insulin therapy and either 10% pentastarch, a high-molecular-weight 10% HES (HES200/0.5; hypertonic), or modified Ringer's lactate for fluid resuscitation. The rate of death at 28 days and the mean score for organ failure were co-primary end points.

The study results showed an increased rate of renal failure in patients with severe sepsis treated with HES (200/0.5) compared to patients treated with Ringer's lactate. At day 90, patients who had received HES, even when they received lower HES doses, were more likely to have renal failure than those who had received Ringer's lactate (30.9% vs. 21.7%, P = 0.04) and were more likely to need renal-replacement therapy (25.9% vs. 17.3%, P = 0.03). The PRAC acknowledged that a number of

[6] Zarychanski, R., A. M. Abou-Setta, et al. (2013). Association of hydroxyethyl starch administration with mortality and acute kidney injury in critically ill patients requiring volume resuscitation: a systematic review and meta-analysis. JAMA 2013;309(7): 678-88
[7] Perel P, Roberts I, Ker K. Colloids versus crystalloids for fluid resuscitation in critically ill patients (Cochrane review).Cochrane Database Syst Rev 2013; CD000567(2):1-73
[8] Claus RA, Sossdorf M, Hartog C *et al*. The effects of hydroxyethyl starch on cultured renal epithelial cells. Anesth Analg. 2010 Feb 1;110(2):300-1

patients received higher dose of HES (200/0.5) (>22 ml/kg/d), however, the risk of RRT was also seen in patients treated with HES (200/0.5) at the recommended daily doses.

- 6S trial (Perner A *et al.* 2012)

The 6S trial is a randomised, multicentre, parallel-group, blinded trial which was conducted in 798 patients with severe sepsis receiving fluid resuscitation in ICU with either 6% HES (130/0.42) (n=398) or Ringer's acetate (n=400) at a dose of up to 33 ml per kilogram of ideal body weight per day. Septic shock was present in 84% of patients of both groups. The composite primary outcome was death or dependence on dialysis 90 days after randomisation.

In this 6S study, there was a significantly higher risk for RRT in patients in ICU treated with HES (130/0.42) (22% (87/398) compared to patients treated with Ringers' acetate (16% (65/400)) (RR: 1.35; 95% CI: 1.01-1.80; P=0.04). The results were supported by multivariate analyses, with adjustment for known risk factors for death or acute kidney injury at baseline.

The MAHs claimed that some patients in the trial appear to have received HES outside the indication hypovolemia. The MAHs claimed that since the median central venous pressure, S_vO_2 and lactate in each arm of the study were within the normal range, the patients were not hypovolemic when enrolled in the trial. The PRAC cannot endorse the MAHs interpretation and considered that central venous pressure, S_vO_2 and lactate can very well be in the normal range and still be compatible with clinical hypovolemia.

Furthermore, the PRAC acknowledged that a significant number of patients had acute kidney injury at randomisation, although renal failure with oliguria/anuria is a contraindication for HES. The patients with acute kidney injury at baseline were evenly randomised to the different treatment groups (142 in HES, 140 in Ringer's Acetate). Acute kidney injury occurred with equal frequency in the two intervention groups and the effect of HES 130/0.42 did not differ significantly between patients with and those without acute kidney injury at the time of randomisation. Therefore, the inclusion of the patients with acute kidney injury is unlikely to have affected the results of the study.

In conclusion, the suggested limitations of the 6S trial cannot be endorsed by the PRAC. The PRAC considered that the 6S study was well-designed and adequately powered. Due to the double blinding and the multi-center design of the study there is a low risk of bias.

The 6S study showed a significant higher risk for mortality at day 90 (see section on Risk of Mortality above) and need for RRT during the course of treatment in patients with severe sepsis and septic shock treated with HES (130/0.42) compared to Ringer's acetate.

- CHEST study (Myburgh *et al*. 2012)

The CHEST study is a randomised, multicentre, blinded, controlled study which was conducted in 7000 patients who had been admitted to an ICU in a 1:1 ratio to receive either 6% HES (130/0.4) in 0.9% sodium chloride or 0.9% sodium chloride (saline) for all fluid resuscitation until ICU discharge, death, or 90 days after randomisation. The main subgroups were: surgical (approximately 42%), sepsis (approximately 29%) and trauma (approximately 8%) patients.

Adult patients admitted to the ICU and whom the treating physician judged to require fluid resuscitation (bolus of intravenous fluid over and above that required for maintenance or replacement fluids) were included. It should be noted that some of the patients have been treated before randomisation. Fluid was administered to correct hypovolaemia at any time during the patients ICU admission. Patients who had received more than 1000ml of HES before screening were excluded.

In this study, RRT was administered to 7.0% (235/3352 patients) of patients treated with HES and in 5.8% (196/ 3375 patients) of patients treated with saline (RR: 1.21; 95% CI: 1.00-1.45; P = 0.04).

Indication for RRT was non-standardised and subjective. The decision when to start and stop RRT was purely dependent on the opinion of the physician (who were unaware of study group assignments) and may have included reasons other than reduced kidney function, such as over-hydration. This made it unlikely that the difference was caused by variations in the thresholds for initiating therapy.

This study also evaluated RIFLE criteria, which are composite of effects on serum creatinine levels and urine output. The results showed that renal risk (RIFLE-R) occurred significantly more often in the saline group (57.3%) as compared to the HES 130/0.4 group (54%; p=0.007). Likewise, renal injury (RIFLE-I) occurred more often in the saline group (38%) as compared to the HES 130/0.4 group (34.6%; p=0.005).

In view of these results, a post hoc analysis was conducted. The results showed that serum creatinine levels were significantly increased in the HES group suggesting a progressive reduction in creatinine clearance, and urine output was significantly decreased in the HES group, as compared with the saline group, during the first 7 days (P = 0.004 and 0.003, respectively).

The PRAC noted that the number of patients who had chronic kidney disease at baseline has not been published, and the status of chronic kidney disease was also not specified. However, the following baseline data have been presented in the study publication. Serum creatinine in HES group was 101.5±57.1 µmol/l and 100.1±58 µmol/l in the saline group. Urine output 6 hours before randomisation was 453.5±418.3 ml in the HES group and 426.6±422.9 ml in the saline group. Therefore, there was no significant difference between both groups at baseline.

In conclusion, the CHEST study has shown an increased risk of RRT in patients treated with HES compared to the patients treated with 0.9% NaCl solution.

6S, CHEST and VISEP studies:

The PRAC has acknowledged the potential limitations of the studies presented by MAHs and noted the request from some MAHs for Good Clinical Practice (GCP) inspections to be conducted for the 6S, CHEST and VISEP studies. According to the MAHs the validity of these studies could be questioned due to potential flaws in their conduct. The PRAC has carefully considered the arguments presented by the MAHs. The PRAC also noted that the 6S study publication made clear reference to compliance with GCP. Notwithstanding the above, the PRAC considered that there was not sufficient evidence provided by the MAHs which would put into question the reliability of the study's results in relation to the identified risks.

- FIRST (Fluids in Resuscitation of Severe Trauma) trial (James MF *et al*. 2011)[9]

The FIRST trial was a randomized, controlled, double-blind study of severely injured patients requiring 3 litres of fluid resuscitation. Blunt and penetrating trauma were randomised separately. Patients were followed up for 30 days. A total of 115 patients were randomized; of which, 109 were studied.

When applying the RIFLE criteria, there was no difference between the groups in renal injury over 30 days between HES and saline groups. In the HES group significantly better lactate clearance and less renal injury than in the saline group was seen for patients with penetrating trauma. For the separately randomised group of patients with blunt trauma no advantage could be seen.

[9] James MF, Michell WL, Joubert IA *et al*. Resuscitation with hydroxyethyl starch improves renal function and lactate clearance in penetrating trauma in a randomized controlled study: the FIRST trial (Fluids in Resuscitation of Severe Trauma). Br J Anaesth 2011;107(5): 693-702

- van der Linden *et al.*(2013)[10]

The review by van der Linden *et al.* assessed the safety of tetrastarches (HES 130, mostly HES 130/0.4) during surgery in 4529 patients. The control group (N=2390) was another colloid, a crystalloid, a blood product, a vasoactive drug or no other treatment.

No differences were seen for adverse renal effects or need for RRT, increased blood loss, allogeneic erythrocyte transfusion or mortality (odds ratio (OR) for HES mortality=0.51(0.24-1.05) P= 0.079; 11/956 deaths reported with tetrastarches and 22/928 in the comparator group).

However, it should be noted that most of the included studies had low numbers of patients between 20 and 90 patients (such as the study by Feldheiser *et al*. (2013)[11] (n=50) or Godet *et al*. (2008)[12] (n=65)), and the larger studies were conducted with between 184 and 203 patients.

Furthermore, the comparator groups were very heterogeneous, some of the studies compared tetrastarches (HES 130) to other HES products (pentastarches (substitution degree 0.5), hexastarch (substitution degree 0.6), hetastarch (substitution degree 0.7), albumin, gelatine, crystalloids, a blood product, a vasoactive drug or no other treatment. The volumes of 130/0.4 used in the studies were rather small compared to those in 6S and CHEST.

The surgery patient group included patients from major abdominal surgery as well as from orthopaedic surgery, patients with trauma or burns some of whom might not be severely ill with regard to their need of therapy of hypovolemia or hypovolemic shock. There is no information on the number of patients in need of intensive care. In addition, the review does not allow conclusions about sepsis patients.

In conclusion, due to different comparators, small sample sizes, rather small doses of HES 130, and very short follow-up periods, this study does not allow any conclusion on renal safety or mortality differences between the use of HES 130/0.4 and crystalloids. The review would only be able to detect extensive differences of HES to all comparators (which include also other HES products). In addition, data from the post-operative follow up period are lacking.

<u>Safety data from meta-analyses</u>

Recently conducted meta-analyses and systematic reviews confirm the increased rate of renal dysfunction in HES treated patients.

- Zarychanski R *et al*. (2013)

In the meta-analysis (Zarychanski *et al*. 2013) targeting critically ill patients, when 7 trials (involving 590 patients) by an investigator were excluded as previously mentioned, HES was found to be associated with increased risk for renal failure among 8725 patients (RR 1.27; 95% CI, 1.09 to 1.47; I2 26%) and an increased risk for RRT among 9258 patients (RR 1.32; 95% CI 1.15 to 1.50; I^2 0%). The conclusion of the authors is that the use of hydroxyethyl starch for acute volume resuscitation is not warranted due to serious safety concerns.

[10] Van der Linden P, James M, Mythen M, Weiskopf RB. Safety of modern starches used during surgery. Anesth Analg 2013;116:35-48

[11] Feldheiser A, Pavlova V, Bonomo T *et al*. Balanced crystalloid compared with balanced colloid solution using a goal-directed haemodynamic algorithm. Br J Anaesth. 2013;110(2):231-40.

[12] Godet G, Lehot JJ, Janvier G *et al*. Safety of HES 130/0.4 (Voluven(R)) in patients with preoperative renal dysfunction undergoing abdominal aortic surgery: a prospective, randomized, controlled, parallel-group multicentre trial. European journal of anaesthesiology 2008; 25: 986-994.

- Haase N et *al*.[13]

The meta-analysis by Haase *et al*. compared HES 130/0.38-0.45 versus crystalloid or albumin in patients with sepsis. This systematic review with meta-analysis and trial sequential analysis includes some small studies (Dolecek M *et al*. and Palumbo D *et al*.) as well as the BaSES[14], CHEST and 6S trials. Altogether nine trials have been included. The meta-analysis showed a higher risk of RRT for HES users (RR 1.36, CI 1.08 to 1.72, Trial sequential analysis (TSA) adjusted 1.03 to 1.80, 1311 patients, five trials), and a higher risk for acute kidney injury (RR 1.18, CI 0.99 to 1.40, TSA adjusted 0.90 to 1.54, 994 patients, four trials).

- Meta-analysis on HES in cardiovascular surgery

A meta-analysis initiated by one of the MAHs was performed to compare HES as volume expanders in cardiovascular surgery with other conventional volume expanders, namely, albumin, crystalloids and gelatin. The meta-analysis focuses on the harm outcomes total blood loss, frequency of blood transfusions, frequency of reoperations, frequency of acute kidney injury, and mortality.

No clear difference was seen comparing different types of HES to crystalloids. The overall methodological problems with this meta-analysis, however, substantially limit the conclusions that can be drawn. Some results support differences in favour of lower substitution ratio HES products but results are not consistent. No conclusions can be drawn from data on reoperation, acute kidney injury or mortality.

The main other adverse reactions reported in published clinical trials are presented below.

Coagulation system

Several studies have shown that HES was associated with platelet dysfunction, decrease of factor VIII and von Willebrand factor levels and interaction with the coagulation system. An increased bleeding tendency was not only detected with high molecular weight HES but also with low molecular weight HES.

In the 6S study more patients in the HES group received blood product transfusions with higher volumes than patients in the Ringer's lactate group (RR: 1.20; 95% CI: 1.07-1.36; p: 0.002). Patients in the HES group were at higher risk for severe bleeding events (P=0.09).

Results of the CHEST study also showed that the first 4 days, the HES group received significantly more blood products than the saline group (78±250 ml vs. 60±190 ml, P<0.001).

In the VISEP study, a significant lower platelet count in septic patients treated with 10% HES 200/0.5 (179,600 per cubic millimeter; interquartile range, 122,000 to 260,000) compared to Ringer's lactate group (224,000 per cubic millimeter; interquartile range, 149,800 to 314,800; P<0.001) was reported.

In the FIRST trial, the HES 130/0.4 group required significantly more blood products [packed red blood cell volumes 2943 (SD:1628) vs 1473 (SD:1071) ml, P=0.005] than the saline group and a significantly greater deterioration in coagulation measures was seen, although this could be due to greater injury severity.

[13] Haase N, Perner A, Hennings LI, et al. Hydroxyethyl starch 130/0.38-0.45 versus crystalloid or albumin in patients with sepsis: systematic review with meta-analysis and trial sequential analysis. Brit Med J2013; [Epub ahead of print]
[14] BaSES Trial: http://clinicaltrials.gov/show/NCT00273728

In the CRYSTMAS trial[15], 29% of patients in the HES group required red blood cell transfusion, compared to 21% in the control group (NaCl 0.9%).

Hepatic organ failure

The CHEST study reported a significantly higher incidence of new hepatic organ failure (in terms of S-Bilirubin increase) in the HES group than in the saline group (1.9% versus 1.2%; RR: 1.56; p=0.03). In addition, deposition of HES into hepatocytes has been reported in patients with worsening hepatic dysfunction (Christidis C *et al.* 2001[16]).

Anaphylactic reactions and pruritus

Anaphylactic reactions and pruritus are rather well characterised unfavourable effects of treatment with HES solutions for infusion. HES associated pruritus seems to be related to dosage.

In the CHEST study, the use of HES was associated with a significant increase in the rate of adverse events (5.3 vs. 2.8%, P<0.001). Of these events, pruritus and rash were the most common.

Conclusions on safety

The PRAC considered the data from recent published clinical trials as well as meta-analyses in the review of the safety of HES solutions for infusion in different patient populations. The three important studies that provided evidence for harm in septic patients or ICU patients including septic patients used three different HES products: 10% HES 200/0.45-0.55 in the VISEP study, HES 130/0.42 in the 6S trial and HES 130/0.4 in the CHEST trial. All three trials showed an increased risk for RRT or renal failure in patients treated with low and high molecular weight HES. The PRAC also considered that the use of HES solutions for infusion has been shown to be associated with an increased risk of mortality at day 90 (6S, VISEP studies). The PRAC acknowledged limitations of the above mentioned studies, but did not consider that they have an impact on the overall safety results. Furthermore, the PRAC considered that these data could be extrapolated to other populations such as trauma, burn injury, and elective surgery patients since they all experienced a systemic inflammatory response which is comparable in nature to the general population of critically ill or septic patients.

In view of the available data, the PRAC considered that the use of HES solutions for infusion has been shown to be associated with an increased risk of mortality and renal replacement therapy or renal failure. In addition, the PRAC took note of the available data showing an increased risk of adverse reactions in patients treated with HES such as increasing bleeding, hepatic organ failure, anaphylactic reactions and pruritus.

2.1.2. Efficacy

HES solutions for infusion are used in the setting of hypovolaemia to expand plasma volume. Colloidal solutions are used to sustain intravascular oncotic pressure and to shorten circulatory stabilisation time. Less amount of fluid for resuscitation is needed when compared to crystalloids.

The PRAC considered the dataset submitted by the MAHs. A critical assessment of studies as well as meta-analyses where HES was used has been performed.

[15] Guidet B, Martinet O, Boulain T *et al*. Assessment of hemodynamic efficacy and safety of 6% hydroxyethylstarch 130/0.4 vs. 0.9% NaCl fluid replacement in patients with severe sepsis: The CRYSTMAS study. *Crit Care*. 2012 May 24;16(3):R94
[16] Christidis C, Mal F, Ramos J *et al*. Worsening of hepatic dysfunction as a consequence of repeated hydroxyethylstarch infusions. J Hepatol 2001; 35:726-32

Critically ill patients / ICU patients

The assessment of the CHEST study in critically ill patients, as well as the results of two recent meta-analyses (Perel P *et al.* 2012; Zarychanski R *et al.* 2013), are discussed in the following section. In addition, the PRAC took note of the results of an unpublished clinical trial (CRYSTAL study[17]).

- The CHEST study (Myburgh JA *et al.* 2012)

The results showed that a total of 597 of 3315 patients (18.0%) in the HES group and 566 of 3336 (17.0%) in the saline group died (RR in the HES group: 1.06; 95% CI: 0.96-1.18). There was no significant difference between HES group and the saline group.

The use of HES was associated with the administration of lower volumes of resuscitation fluid, although the ratio between HES and saline was similar to that observed in other blinded trials, suggesting that the use of HES was not associated with a substantive volume-sparing effect (6S study, CRYSTMAS trial, James *et al*). There was no clinically meaningful volume-sparing effect of HES.

Therefore, the CHEST study does not provide evidence that resuscitation with 6% HES (130/4), as compared with saline, in the ICU provided any clinical benefit to the patient. Moreover as previously discussed, the results showed that treatment with HES in critically ill patients has been associated with an increased risk of RRT.

A limitation of the study is that the observed rate of death was lower than predicted. In addition, patients were recruited after admission to the ICU, when the requirements for fluid resuscitation are often less than those for patients in the emergency department or the operating room. Despite these limitations, the trial had sufficient statistical power to detect an absolute mortality difference of 3 percentage points.

These findings have been strengthened by two recent meta-analyses (Cochrane Review: Perel P et al. 2013; Zarychanski R et al. 2013) (see section Risk of mortality).

In conclusion, there was no clinically meaningful volume-sparing effect of HES in this patient population. Therefore, no benefit with HES has been demonstrated in this population of patients.

- CRYSTAL study

The Efficacy and Safety of Colloids Versus Crystalloids for Fluid Resuscitation in Critically Ill Patients (CRYSTAL) by Annane *et al*. trial compared any type of colloid versus any type of crystalloid in ICU patients who needed fluid resuscitation with the primary endpoint day 28 mortality. Secondary outcome measures were ICU and hospital mortality rates, number of days free of mechanical ventilation, vasopressors, renal replacement therapy, and organ system failure (90 days), difference in the area under the curve of MAP from inclusion to hour 24, weight gain, PaO2/FiO2 ratio, chest x ray score (day 2), frequency of adverse events (90 days) and length of stay. The time between the decision to resuscitate a patient with fluids and randomisation was kept as short as possible (15 minutes or less). The amount of HES was not allowed to exceed 30 ml/kg/24 hours. According to the protocol, the starch cumulative dose was planned to be limited to 35/40 ml/kg for all ICU stay. In case additional volume replacement was necessary, gelatines or albumin could be used. Groups were stratified by site and diagnosis: 1. Trauma or haemorrhage, 2. Sepsis, 3. other diagnoses. Blinding was considered unfeasible, except primary endpoint was assessed by a blinded assessor. In the colloid group, 774 (54.7%) of patients were included in the sepsis stratum and compared to 779 (54.0 %) in the crystalloid group. More than 40 % of the colloid group received HES (30% received gelatine).

[17] Annane, D. The CRYSTAL trial. 2013. CRISTAL Trial: http://clinicaltrials.gov/ct2/show/NCT00318942
SLRF 2013, CRYSTAL, Annane D http://www.srlf.org/congres/ancien- congres/2013/index.phtml.

First data were presented in January 2013 in Paris (41 Congrès International de la Société de Réanimation de Langue Française). On day 28, the mortality in the colloid arm was 25.4 % versus 27 % in the crystalloid arm (p=0.30, primary endpoint). On day 90, 30.7 % of patients died in the colloid arm versus 34.2 % in the crystalloid arm (p=0.04). The odds ratio was 0.90 (95 % CI: 0.745-1.082) which is a non-significant trend in favour of colloids as regards the primary endpoint 28- day mortality. For the 90 day mortality the odds ratio was 0.83 (95 % CI: 0.693-0.948).

Although, the MAHs claimed that the preliminary results of the study show a clinical benefit of HES in critically ill patients, the data of this study have not been published at the time of this discussion nor was a full report available and therefore further assessment is required before any conclusion can be drawn.

Sepsis

A number of results from several large, overall well-conducted randomised clinical trials have been identified as a basis for a benefit-risk assessment of HES in patients with sepsis. The critical assessment of three recent published clinical trials (6S, CRYSTMAS, VISEP) and an observational cohort study (Bayer O *et al.* 2012[18]) is presented below. In addition, the PRAC considered the review of the results of unpublished clinical trial (BaSES study[19]) which were presented by one of the investigators of this study during an oral explanation in June 2013 PRAC meeting.

- 6S trial (Perner A *et al.* 2012)

The results of the study showed a significantly higher mortality at day 90. There were 51% (201/398) of the patients in the HES 130/0.42 group versus 43% (172/400) in the Ringer's acetate group who died (RR: 1.17; 95% CI 1.01-1.36; P= 0.03). The results were supported by multivariate analyses, with adjustment for known risk factors for death or acute kidney injury at baseline. Doses were given up to 33 mL/ideal bodyweight/day. As in the CHEST study, there does not appear to be any volume sparing effect of HES, which would be expected to be the main benefit from a colloid.

Therefore, the study 6S did not show any benefit of HES for patients with severe sepsis.

Limitations of this study are addressed below.

The MAHs claimed that many patients in the 6S study were already haemodynamically stable at baseline, and therefore a volume replacement solution was not indicated in these patients. The PRAC considered that baseline characteristics were comparable in both groups and that there was no significant difference in blood products given before randomisation between the groups (packed red blood cells, fresh frozen plasma, platelets). One of the MAH stated that patients in the HES group might have been more ill than those in the Ringer's acetate group. Patients in the HES group had more emergency admissions, but only 27% versus 24%, so that it seems unlikely that the unfavourable results could be explained by this small difference. Therefore, the PRAC considered that there is no evidence in the baseline characteristics indicating that HES treated patients were more severely ill than patients in the Ringer's acetate group.

Even though most of the patients in the 6S trial had fluid volume therapy 24 hours before randomisation the criteria for hypovolemia for the patients were fulfilled. 336/398 (84%) of the patients in the HES group and 337/400 (84%) in the Ringer's acetate group had shock at randomisation which was defined as a mean arterial pressure of less than 70 mm Hg, the need for on-

[18] Bayer O, Reinhart K *et al*. Effects of fluid resuscitation with synthetic colloids or crystalloids alone on shock reversal, fluid balance, and patient outcomes in patients with severe sepsis: A prospective sequential analysis. Critical Care Medicine 2012;40(9):September

[19] BaSES Trial: http://clinicaltrials.gov/show/NCT00273728

going treatment with vasopressor or inotropic agents, or a plasma lactate level of more than 4.0 mmol/L in the hour before randomisation. Patients with severe sepsis were randomly assigned to receive fluid resuscitation in the ICU. The trial definition of fluid resuscitation was a bolus of intravenous fluid which was given to increase intravascular volume, the fluid should be given in addition to that required to replace on-going insensible losses, urinary losses etc. or for nutrition. However, inclusion criteria were the same for each fluid group and thus both groups are comparable.

No substantial differences were found with regard to the study fluid amounts of HES compared to Ringer's acetate in patients with severe sepsis.

The 6S study was well-designed and adequately powered. Due to the double blinding and the multi-center design of the study there is a low risk of bias. The vehicle of HES used in the intervention group was Ringer's acetate. Since Ringer's acetate was also used as control, the causal effect of HES on outcomes can be assessed.

- VISEP study

In the randomised, multicentre, two-by-two factorial trial including 600 patients (VISEP Study), the rate of death at 28 days did not differ significantly between the HES group and the Ringer's lactate group (26.7% and 24.1%, respectively; P=0.48). However, mortality at day 90 was significantly increased in patients who received higher dose of HES 205/0.5 (>22 ml/kg bodyweight per day) compared to the Ringer's lactate group (41.0% vs 33.9%, P=0.09).

- CRYSTMAS trial (Guidet B *et al*. 2012)

The CRYSTMAS trial is a prospective, multicentre, controlled, double blinded randomised trial which was conducted in 196 patients with severe sepsis, where 100 patients received HES and 96 patients received sodium chloride 0.9%. The primary endpoint of the study was the amount of study drug required to achieve initial hemodynamic stabilisation (HDS).

The results of the study showed that an about 20% less amount of HES 130/0.4 was needed to achieve circulatory stabilisation of septic patients in the initial phase of resuscitation compared to saline. However, the reduction in volume requirement was not clinically relevant (while statistically significant) and the total amount of study fluid over 4 days was similar for HES and saline. Therefore, the PRAC was of the opinion that the volume sparing effect observed in this study did not translate in clinical benefit for patients.

The study was not powered to show differences in mortality or kidney function.

- Cohort study (Bayer O *et al.* 2012)

The study was a non-randomised observational cohort study which compared HES (predominantly 6% HES 130/0.4), 4% gelatine, and crystalloids in 1046 patients with severe sepsis (HES n=360; gelatine n=352 and crystalloids n=334).The primary outcome was time to shock reversal and the secondary endpoint was required fluid volumes in severe sepsis.

The results showed that all groups had similar time to shock reversal (P=0.68). More fluid was needed over the first 4 days in the crystalloid group (fluid ratios 1.4:1 [crystalloids to HES] and 1.1:1 [crystalloids to gelatine]). After day 5, fluid balance was more negative in the crystalloid group. HES and gelatine were independent risk factors for acute kidney injury (OR: 95% CI: 2.55, 1.76-3.69 and 1.85, 1.31-2.62, respectively).

Therefore, according to the data shock reversal was achieved equally fast with synthetic colloids or crystalloids. Use of colloids resulted in only marginally lower required volumes of resuscitation fluid.

As this is a non-randomised observational cohort study with a sequential design, the possibility of systematic differences between the two groups reflecting non-measured alterations in other aspects of therapy, or period effects as a result of general improvements in the care of septic patients cannot be ruled out. Furthermore, it cannot be excluded that uncontrolled changes in treatment patterns such as changes in end of life decisions may have contributed to the decreased length of stay on the ICU and reduced time on the ventilator over the three sequential study periods.

- BaSES study

The PRAC further considered the results of an unpublished clinical study, the BaSES study. The results of this study were presented by one of the investigator during an oral explanation at PRAC in June 2013.

The BaSES (Basel Starch Evaluation in Sepsis) trial was a single centre investigator-initiated study performed in Basel, Switzerland and first presented at the European Society of Anaesthesiology (ESA) conference in June 2012. This double-blind, randomised study included 241 patients with severe sepsis and septic shock treated with 6% HES (117/241) or crystalloid (124/241). Both groups received additional infusion of Ringer's lactate solution. Mortality among ICU patients (6% HES: 28% vs. crystalloid: 29%) and hospital mortality (6% HES: 30% vs. crystalloid: 31%), as well as renal function parameters, did not differ between groups. However, there was a significantly reduced hospital length of stay in favour of 6% HES (6% HES : 20 days, vs. crystalloid: 28.5 days).

However, the data of the study have not yet been published and therefore further assessments are necessary before any conclusion can be drawn.

The two studies (6S, VISEP) have shown a significant higher mortality at day 90 in patients with severe sepsis and septic shock. The observational cohort study (Bayer *et al.*) did not show any difference between HES and crystalloids with regard the time to shock reversal. The PRAC took note of the results of the BaSES study suggesting a benefit of HES over crystalloids in critically ill patients and patients with sepsis. However, the PRAC considered that the available data coming from this study are limited as the study is not published nor a full report is available, and therefore require further assessment before any conclusion can be drawn.

In addition to the above PRAC noted that recent clinical guidelines do not recommend use of HES in this patient population. The Surviving Sepsis campaign guideline[20] which was updated in 2012 does not recommend the use of HES for fluid resuscitation in patients with severe sepsis or septic shock because of the absence of any clear benefit following the administration of colloid solutions compared to crystalloid solutions. A high grade recommendation for the use of crystalloid solutions in the initial resuscitation of patients with severe sepsis and septic shock is supported.

Since 2008, the German S-2k guideline[21] (Deutsche Sepsis-Gesellschaft) also recommends against the use of HES 200/0.5 and HES products with lower molecular weight because of lack of data.

The present available data do not show any benefit for HES when compared to crystalloids for the patients with severe sepsis and septic shock. Overall PRAC considered that for subjects with severe sepsis or septic shock the risks of increased mortality (and more frequent use of RRT) are considered to outweigh the limited benefits of more rapid attainment of hemodynamic stability compared to crystalloids.

---

[20] Dellinger RP, Levy MM, Rhodes A *et al.* Surviving Sepsis Campaign: International guidelines for Management of severe Sepsis and Septic Shock. Intensive Care Med. 2013;39(2):165-228

[21] Reinhart K, Brunkhorst FM, Bone HG et al. Prevention, diagnosis, therapy and follow-up care of sepsis: 1st revision of S-2k guidelines of the German Sepsis Society (Deutsche Sepsis-Gesellschaft e.V. (DSG)) and the German Interdisciplinary Association of Intensive Care and Emergency Medicine (Deutsche Interdisziplinäre Vereinigung für Intensiv- und Notfallmedizin (DIVI)). Ger Med Sci. 2010; 8: Doc14.

Burn injury

Few data are available supporting the use of HES in burns.

The pilot open-label randomised study from Sudhakar *et al.* (2008)[22] investigated the safe use of HES for initial resuscitation and if early treatment can reduce crystalloid overload in burned patients. HES doses were given up to 20 mL/kg in 48 hours, post burn parameters for oedema were more favourable for HES, significantly more patients in the crystalloid group died (9/10 (90%) versus 11 /22(50%)). Patients in the crystalloid group had 7% more total burn surface at admission.

Although the results suggested a more rapid response to HES compared to crystalloids, severe methodological issues have been identified meaning that no efficacy or safety (both short time and long-time) conclusion can be drawn from this study.

With the negative effect seen on mortality in sepsis, the well demonstrated deposit of HES in tissues, and the pathophysiological similarities between sepsis and early burn injury physiology with a massive inflammatory response and capillary leakage, the benefit-risk of HES in burns cannot presently be considered favourable.

Trauma patients

Trauma patients constitute an important subgroup of patients with particular considerations needed. Trauma was included as a pre specified subgroup analysis in a recent meta-analysis (Zarychanski *et al.* 2013) which included five studies (Nagy K *et al*. 1993[23]; Younes RN *et al*. 1998[24]; Carli P *et al*. 2000[25]; James MF *et al*. 2011; Myburgh JA *et al*. 2012; Myburgh JA *et al*. 2013[26]). There was no signal of benefit, if anything there appeared to be an increase in mortality associated with HES (RR 1.24 (0.81, 1.90).

Figure 1: Outcome in trauma patients (Zarychanski *et al*. 2013)

Outcome or Subgroup	Studies	Participants HES (n/N) Control(n/N)		Effect estimate (CI)	I^2 (uCI)
Trauma[4,23,48,51,54]	5	45/ 434	35/ 430	RR 1.24 (0.81, 1.90)	0% (0%, 75%)

The FIRST trial (James MF *et al.*) in trauma patients provides information on the comparison to the use of HES 130/0.4 versus salines (NaCl 0.9%), but not on the effect on mortality or renal dysfunction. In this randomised, controlled, double-blind study of severely injured patients requiring 3 litres of fluid resuscitation, patients were followed up for 30 days. Blunt and penetrating trauma were randomised separately. A total of 115 patients were randomised; of which, 109 were studied. For patients with penetrating trauma (n=67), the mean (SD) fluid requirements were 5.1 (2.7) litres in the HES group and 7.4 (4.3) litres in the saline group (P, 0.001). In blunt trauma (n=42), there was no difference in study fluid requirements, but the HES group required significantly more blood products as previously mentioned. Although the study showed a positive effect of HES in penetrating trauma patients, the results need further confirmation due to the relatively low number of events.

[22] Sudhakar GV *et al.* Role of HES 130/0.4 in resuscitation of patients with major burn injury. *Transfus Altern Transfus Med* 2008; 10(2):43/50
[23] Nagy K. *et al.* A comparison of pentastarch and lactated Ringer's solution in the resuscitation of patients with hemorrhagic shock. *Circ Shock* 1993;40(4): 289-94.
[24] Younes RN *et al*. Use of pentastarch solution in the treatment of patients with hemorrhagic hypovolemia: randomized phase II study in the emergency room. World J Surg 1998;22(1): 2-5
[25] Carli P *et al*. (2000). Remplissage vasculaire prehospitalier en taumatologie: hesteril 6% versus plasmion. Jeur 2000;13(1-2): 101-105.
[26] Myburgh JA *et al*. Hydroxyethyl starch or saline in intensive care. N Engl J Med 2013; 368(8): 775

In this study, the volume of clear fluids (crystalloids) administered was half of that given in other trials where an association with compartment syndrome and gastrointestinal dysfunction with high volumes of crystalloids could be seen. In addition, severe abdominal compartment syndrome was only seen in patients receiving very large crystalloid loads. The absence of any difference in recovery of gut function and the low incidence of abdominal compartment syndrome suggest that in trauma patients, reasonable fluid resuscitation is not a major risk factor. However, this study only gives sparse information on whether patients with penetrating trauma and a need for high fluid volume have a benefit when treated with HES.

In view of the limited benefit of HES in this setting, and the potential harm as referred above the benefit risk ratio is not considered favourable.

Elective surgery

Several studies in surgery patients with HES have been presented by the MAHs. The vast majority of the studies presented could only provide limited information since only comparisons with other HES solutions were made. The critical assessment of clinical trials published by Standl T *et al.* 2008[27] and Mercier FJ *et al.* 2011[28] as well as the study published by Madi-Jebara et al. 2004[29] and Feldheiser *et al*[30] are presented below.

- Standl T *et al.* 2008

A prospective, randomised, open-label pilot study conducted in paediatric patients less than 2 years of age compared HES 130/0.4 to albumin 5% in cardiac and non-cardiac surgery. The results of the study showed similar efficacy profiles between both treatments (observation time until hospital discharge). Therefore, in this study, short term efficacy for HES 130/0.4 seems equal with albumin when used as volume expander in small children. However, the limited observation time and number of studied children prevents any conclusions of the overall efficacy profile of HES 130/0.4 in this population.

- Mercier FJ *et al.* 2011

The phase IV, randomised, controlled, blinded clinical trial compared 500 mL HES 130/0.4, 6% + 500 mL Ringer's lactate vs 1000 mL Ringer's lactate in the context of spinal anaesthesia for caesarean section in 167 patients. The results of the study showed hypotension periods in 37% in the HES group and in 55% in the Ringer's lactate group. HES could be detected in umbilical cord. The results suggested that short term efficacy seems better for HES compared with Ringer's lactate when equal amounts of volume are administered. It should be noted that this comparison is of limited relevance. A larger volume of Ringer's lactate is needed to generate a comparable plasma volume expansion as HES. Any crystalloid/colloid comparison needs to take this fact into account. Consequently, the addition of Ringer's lactate in the HES group, made in order to facilitate blinding, distorts the comparison. Lower incidence of hypotension is therefore expected and the results are of limited clinical relevance. A volume sparing effect has not been demonstrated and relevance of the short-term hemodynamic differences is unclear since potential long-term effects are not studied.

---

[27] Standl T, Lochbuehler H, Galli C *et al.* HES 130/0.4 (Voluven®) or human albumin in children younger than 2 yr undergoing non-cardiac surgery. A prospective, randomized, open label, multicentre trial. *Eur J Anaesthesiol* 2008; 25(6): 437-45

[28] Mercier FJ, Diemunsch P, Ducloy-Bouthors A *et al.* 6% HES (130/0.4) vs. Ringer's Lactate to Prevent Hypotension during Spinal Anesthesia for C-section. American Society of Anesthesiologists (ASA), Annual Meeting 2011; A973

[29] Madi-Jebara S, Goshn A, Cherfane A, *et al*. Prevention of hypotension after spinal anesthesia for caesarean section: Voluven (6% hydroxyethyl starch 130/0.4) versus lactated Ringer's solution. Anesthesiology 2004; 101(suppl): A1197

[30] Feldheiser A, Pavlova V, Bonomo T, Jones A, Fotopoulou C, Sehouli J, Wernecke KD, Spies C. Balanced crystalloid compared with balanced colloid solution using a goal-directed haemodynamic algorithm. British journal of anaesthesia 2013 ;110(2):231-40

- Madi-Jebara *et al*. 2004

The study was a randomised, open label study comparing HES 130/0.4, 6% (500 mL) with Ringer's lactate (1000 mL) in 120 patients with elective caesarean section. The results of the study showed that hypotension occurred in 64% in the HES group and 81% in the Ringer's lactate group. The study suggested that HES 130/0.4 6% seems to have benefits over twice the volume of Ringer's lactate in preventing spinal anaesthesia induced hypotension. The advantages for preloading with HES 130/0.4 6% are small but measurable when only spinal anaesthesia is used.

- Feldheiser *et al.* 2013

The Feldheiser study targets major elective surgery studying 50 patients undergoing major cancer surgery. It is however a small study (by the authors characterised as a pilot study where tests should be considered exploratory) and not powered to study long-term mortality. In the short term, the study indicated a benefit for the HES group in maintaining a better cardiac output with less volume given (Time to reach 50 mL/kg: HES = 2:26 h, crystalloids = 3:33 h).

Overall, it appears to be a difference in hemodynamic variables when HES is used for preload during spinal anaesthesia, as compared to Ringer's solution. Benefit for elective surgical patients has been shown in short-term surrogate hemodynamic outcomes along with a modest volume sparing effect (mean crystalloid-colloid ratio 1.8, SD 0.1) (Hartog et al. 2011[31]). Studies in elective surgery have not been performed in sufficiently large populations and had a sufficiently long follow-up time to allow any conclusions about risks for mortality and use of RRT. In view of the limited benefit and the potential harm as referred above the benefit risk ratio is not considered favourable.

In addition, the PRAC took note of a meta-analysis initiated by one of the MAHs which compared HES as volume expanders in cardiovascular surgery with other conventional volume expanders, namely, albumin, crystalloids and gelatin. The meta-analysis focuses on the harm outcomes total blood loss, frequency of blood transfusions, frequency of reoperations, frequency of acute kidney injury, and mortality. The PRAC noted that no assessment of bias in the underlying studies is made. In addition, the PRAC highlighted that the design issues are not in line with the PRISM guidelines for meta-analyses. The PRAC was of the opinion that the report does not provide a conclusion for HES degree of substitution 0.45 (tetrastarch) compared to albumin but the result appears to favour albumin as does the results for HES degree of substitution 0.7 (hetastarch). The results for HES 0.4 appear to favour HES but results are sensitive to the definition of blood loss. The overall methodological problems with this meta-analysis substantially limit the conclusions that can be drawn. No clear difference was seen comparing different types of HES to crystalloids.

Conclusion on efficacy

In view of the available data, the PRAC is of the opinion that HES solutions for infusion do not provide any clinical benefit in critically ill patients over crystalloids. There was no clinically meaningful volume-sparing effect of HES in this patient population. For subjects with severe sepsis or septic shock the risks of increased mortality (and more frequent use of RRT) are considered to outweigh the limited benefits of more rapid attainment of hemodynamic stability compared to crystalloids. The PRAC also considered that studies on trauma and elective surgery only showed limited clinical benefit of HES. Furthermore, evidence is not found that resuscitation with colloids reduces the risk of death in patients with trauma, burns, or after surgery when compared to crystalloids.

[31] Hartog C, Kohl M, *et al*. "A systematic review of third-generation hydroxyethyl starch (HES 130/0.4) in resuscitation: safety not adequately addressed. Anesth Analg 2011; 112(3): 635-45

Ad-hoc Expert group

The PRAC also consulted the views of experts through an Ad-Hoc Expert meeting.

The expert group agreed that the now consistent hydroxyethyl starch (HES) data for harm in patients with sepsis could be extrapolated to patients with burn injury, mixed conditions needing critical care units' resources, trauma, elective surgery patients and in any other clinical setting.

The expert group considered that these data could be extrapolated to these patient groups, since they all experience a systemic inflammatory response which is comparable in nature to the general population of critically ill or septic patients. Moreover, as these patients are at risk of developing critical illness, they are therefore also at risk of developing harm from the prior administration of starch-based intravenous fluids.

The expert group considered that there is no patient relevant benefit (like hospital length of stay or survival) for the patients in taking HES above and beyond surrogate parameters and they could not identify any subgroup of patient who should or should not be included. This view is supported by several peer-reviewed research studies. The group were therefore of the view that patients in these clinical groups were at increased risk of harm from starch-based intravenous fluids.

3. Overall discussion and risk/benefit assessment

The PRAC considered all available data which includes recently published large clinical trials in critically ill patients and patients with sepsis, several meta-analyses as well as results of two unpublished clinical trials which compared HES with crystalloids and other colloids. The PRAC also consulted the views of experts through an Ad-Hoc Expert meeting.

The PRAC is of the opinion that HES do not provide any clinical benefit in critically ill patients over crystalloids. There was no clinically meaningful volume-sparing effect of HES in this patient population. For subjects with severe sepsis or septic shock the risks of increased mortality (and more frequent use of RRT) are considered to outweigh the limited benefits of more rapid attainment of hemodynamic stability compared to crystalloids. Furthermore, evidence is not found that resuscitation with colloids reduces the risk of death in patients with trauma, burns, or after surgery when compared to crystalloids.

PRAC also considered that the use of HES for solutions for infusion has been shown to be associated with an increased risk of mortality and renal replacement therapy or renal failure. HES is also associated with other serious adverse reactions such as increased bleeding, hepatic organ failure, anaphylactic reactions and pruritus.

In view of the available data, the PRAC concluded that the benefit risk balance of hydroxyethyl starch solutions for infusion is not favourable in the approved indications and in any patient population.

4. Re-examination procedure

Following the adoption of the PRAC recommendation during the June 2013 PRAC meeting, re-examination requests were received from several MAHs involved in the procedure, including Fresenius Kabi Deutschland GmbH, B. Braun Melsungen AG and Serumwerk Bernburg AG on 16th of August 2013. The scope of the re-examination focused on the re-evaluation of the benefit-risk of HES solutions for infusion in the treatment of hypovolaemia and hypovolemic shock in patient populations under specific settings (e.g. surgery and trauma).

It is noted that the PRAC is a scientific committee and that while it operates within the legal framework, it cannot discuss the specific merits of procedural and legal aspects of administrative

procedures laid down in the legislation. As a result, procedural and legal considerations are outside the remit of the PRAC, and therefore the re-examination of the referral procedure under Article 31 of Directive 2001/83/EC focused only on the scientific grounds for re-examination.

Detailed grounds for re-examination submitted by the MAHs

The MAHs disagreed with the negative benefit risk assessment of the PRAC of HES solutions for infusion:

- The MAHs considered that the beneficial effects for HES with regard to volume-stabilising effect and volume resuscitation as well as hemodynamic endpoints have been shown in several patient populations (e.g. surgery, trauma) and these have not been adequately assessed by the PRAC.
- The MAHs considered the extrapolation of study results derived from septic and critically ill patients to all other patients groups and indications not justified.
- The MAHs considered that the main studies (VISEP, 6S, CHEST) which were the main basis of the PRAC recommendation have strong limitations concerning study design and conduct. The MAHs considered that in these studies, many patients received HES solutions, although they were already haemodynamically stabilised at baseline meaning that many of those patients had no need for volume therapy and/or were partly overdosed.
- The MAHs considered that two clinical trials (BASES and CRYSTAL) have not been adequately assessed by the PRAC and that the results of these trials could potentially have a major impact on the conclusions of the procedure.
- The MAHs considered that spontaneous report on HES are rare compared to the number of patients under treatment with HES containing medicinal products
- The MAHs also considered that alternative synthetic colloids (e.g. dextran, gelatin) are less studied than HES and do not represent better alternatives (e.g. risk of anaphylactoid reactions). The MAHs further highlighted that safety data evaluated since registration of the HES products have not shown higher risk of mortality or higher risk of negative effects on renal functions.

The MAHs therefore considered that the benefit risk balance of HES solutions for infusion in the treatment of hypovolaemia and hypovolemic shock is positive in some patient populations (e.g. surgery, trauma).

PRAC conclusion on grounds for re-examination

The PRAC confirmed that it considered the totality of the data available in the context of the initial recommendation of the article 31 referral procedure. The PRAC reiterated that three important studies have shown evidence of harm in septic patients or ICU patients when HES products were administered (VISEP, 6S and CHEST). The studies' results reflect the clinical practice in the participating centres with regards to fluid resuscitation. In clinical practice patient history and clinical situation together with trends in various clinical variables are weighted together and treatment for hypovolemia is attempted. The results also showed an increase risk for RRT or renal failure in those treated HES; limitations have been previously discussed and were acknowledged.

Data available from studies in other patient populations, looking into volume-sparing effect and haemodynamic endpoints (e.g. Hanart C *et al.* 2009[32], Feldheiser A *et al.* 2013[33], James MF *et al.*

[32] Hanart C, Khalife M, De Villé A, et al. Perioperative volume replacement in children undergoing cardiac surgery: Albumin versus hydroxyethyl starch 130/0.4. Crit Care Med 2009;37(2):696-701

2011, Neff *et al.* 2003[34]; Myburgh *et al.* 2012; Ogilvie *et al*. 2010[35]) have also been considered. However, the studies' results were of limited value, as sample sizes were either too small, imbalances were noted, or there was short follow up and inappropriate control. Other meta-analysis (e.g. Van der Linden *et al.* 2013) do not allow thorough conclusions as studies with small sample sizes, low doses of HES, short follow up and different comparators including an experimental hemoglobin solution and comparison with other HES solutions were included. Gattas DJ *et al*. 2013[36]; Wiedermann CJ *et al.* 2012[37] have also shown safety concerns, namely an increased risk for death (Gattas DJ *et al.* and Wiedermann CJ *et al.*) and renal replacement therapy (Gattas DJ *et al*.). The MAH's meta-analysis on HES in cardiovascular surgery had also been considered before. No clear difference was seen between HES and crystalloids, and although some results did support differences in favour of lower substitution ratio HES products, some limitations were noted. The MAHs further referred to a number of small studies or meta-analysis in support of the use of HES 130/0.4 in surgical patients (e.g. Kasper *et al.* 2003; Gallandat-Huet *et al.* 2000; Ickx *et al.* 2003; Magder *et al.* 2010; Martin *et al.* 2013). The evidence for improved volume sparing effect observed in these studies, which is considered a surrogate endpoint, must be balanced against the lack of evidence to demonstrate benefits in relation to longer-term, clinically meaningful endpoints.

With regards to alternatives, the PRAC considered that there is no evidence from well-designed head to head comparisons that other colloids are associated with more or less serious risks compared to HES solutions.

The MAHs argued that the spontaneous adverse drug reactions (ADR) reports on HES have to be taken into consideration for the safety evaluation of HES. The PRAC noted that due to limited number of cases reported this data is of limited added value. It is well known that frequencies of certain ADRs as well as causality cannot be determined through passive adverse reaction reporting.

With regards to the evidence from CRYSTAL and BaSES studies, only preliminary results were available for the initial assessment in the referral under Article 31 of Directive 2001/83/EC. The PRAC acknowledged that new results became available after finalisation of the initial assessment of the referral under Article 31 of Directive 2001/83/EC and the MAHs claimed new evidence of a clinical benefit. The PRAC recognised that new evidence could be of relevance but these new data do not fall within the scope of this referral under Article 31 and therefore cannot be considered. These new data were nevertheless assessed in the referral on HES on the basis of Article 107i of Directive 2001/83/EC that was conducted separately but in parallel to the re-examination of the referral under Article 31 of Directive 2001/83/EC.

In addition, the PRAC consulted another *ad hoc* expert group that was convened on 13 September 2013. The *ad hoc* expert group was requested to clarify whether from a clinical perspective, given the available data and taking into account the increased risk of renal events and the increased mortality, there are subpopulations of critically ill patients (defined as patients admitted to the ICU) for whom HES treatment remains beneficial. Some experts considered that subpopulations of critically ill patients can be identified for whom treatment with HES remains beneficial such as all critically ill patients in emergency, all patients from ICU including limited trauma patients before surgery. However, the

[33] Feldheiser A, Pavlova V, Bonomo T, et al. Balanced crystalloid compared with balanced colloid solution using goal-directed haemodynamic algorithm. Br J Anaesth 2013; 110(2):231-40
[34] Neff TA, Doelberg M, Jungheinrich C, et al. Repetitive large-dose infusion of the novel hydroxyethyl starch 130/0.4 in patients with severe head injury. AnesthAnalg 2003;96(5):1453-9
[35] Ogilvie MP, Pereira BM, McKenney MG, McMahon PJ, Manning RJ, Namias N, Livingstone AS, Schulman CI, Proctor KG. First report on safety and efficacy of hetastarch solution for initial fluid resuscitation at a level 1 trauma center. Journal of the American College of Surgeons 2010; 210: 870-80, 880
[36] Gattas DJ, Dan A, Myburgh J, Billot L, Lo S, Finfer S; CHEST Management Committee. Fluid resuscitation with 6% hydroxyethyl starch (130/0.4 and 130/0.42) in acutely ill patients: systematic review of effects on mortality and treatment with renal replacement therapy. Intensive Care Med 2013 Apr;39(4):558-68
[37] Wiedermann CJ, Joannidis M. Mortality after hydroxyethyl starch 130/0.4 infusion: an updated meta-analysis of randomized trials. Swiss Med Wkly 2012 Jul 30;142:w13656

majority of experts considered that based on the small and limited available studies it is not possible to identify any subpopulation in which the benefit would outweigh the risks. Overall the expert group agreed that the benefit may exist in severe hypovolaemia in short duration only at the beginning i.e. perioperative setting and disappearing faster with patient's stabilisation. The experts suggested that benefits of HES may be seen in perioperative bleeding. The expert group unanimously agreed that the increased risk of renal events and the increased mortality observed in patients with sepsis and the critically ill could not be directly extrapolated to perioperative setting or trauma or to any other clinical setting. The experts agreed that the data available is not convincing however may suggest that the risks are lower in other settings than sepsis and critically ill patients. The experts highlighted that the administration of HES to normovolaemic patients in certain trials was potentially an important issue. The experts further highlighted that additional research on HES must be undertaken.

Finally the PRAC considered the data presented by the MAHs during the oral explanation held on 7 October 2013.

Overall risk-benefit conclusion

In view of the grounds for re-examination submitted by the MAHs, the PRAC took into consideration only the data available at the time of the previous recommendation in June 2013. This included recently published large clinical trials in critically ill patients and patients with sepsis, several meta-analyses as well as results of two unpublished clinical trials which compared HES with crystalloids and other colloids. The PRAC also consulted the views of experts through Ad-Hoc Expert meetings.

The PRAC considered that the use of HES for solutions for infusion has been shown to be associated with an increased risk of mortality and renal replacement therapy or renal failure. HES is also associated with other serious adverse reactions such as increased bleeding, hepatic organ failure, anaphylactic reactions and pruritus. For subjects with severe sepsis or septic shock the risks of increased mortality (and more frequent use of RRT) are considered to outweigh the limited benefits of more rapid attainment of haemodynamic stability compared to crystalloids. Furthermore, sufficient evidence is not available for this procedure to indicate that use in other indications outweighs the risks.

In view of the available data, the PRAC concluded that the benefit risk balance of hydroxyethyl starch solutions for infusion is not favourable in the approved indications and in any patient population. These conclusions are without prejudice to the conclusions of the referral under Article 107i of Directive 2001/83/EC that was conducted separately but in parallel. In the procedure under Article 107i of Directive 2001/83/EC additional data has been included that was not available when the PRAC issued its recommendation on the referral in accordance with Article 31 of Directive 2001/83/EC in June 2013 and therefore could not be considered in the re-examination of the latter in October 2013.

5. Communication plan

The PRAC considered that a Direct Healthcare Professional Communication (DHPC) was needed to communicate on the review of hydroxyethyl starch and the recommended regulatory measures. The communication should take due account of the conclusions of the procedure under article 107i, where additional data was considered.

6. Conclusion and grounds for the recommendation

Whereas,

- The Pharmacovigilance Risk Assessment Committee considered the procedure under Article 31 of Directive 2001/83/EC resulting from pharmacovigilance data, for hydroxyethyl starch containing products for solutions for infusion (see Annex I).

- The Pharmacovigilance Risk Assessment Committee considered the data available for hydroxyethyl starch containing products for solutions for infusion in relation to the risk of mortality and renal failure. This included data from clinical studies and meta-analyses and Marketing Authorisation Holder's responses.
- The Pharmacovigilance Risk Assessment Committee considered that the use of hydroxyethyl starch is associated with an increased risk of mortality and renal replacement therapy or renal failure as well as other serious adverse reactions.
- The Pharmacovigilance Risk Assessment Committee therefore concluded, in view of the available data, that the increased risk of mortality and renal replacement therapy or renal failure associated with the use of hydroxyethyl starch containing medicinal products for solutions for infusion outweighs its limited clinical benefits in the approved indications and in any patient population.

The Pharmacovigilance Risk Assessment Committee, as a consequence, concluded that pursuant to Article 116 of Directive 2001/83/EC the benefit-risk balance for hydroxyethyl starch containing products for solutions for infusion is not favourable.

Therefore, in accordance with Articles 31 and 32 of Directive 2001/83/EC, the Pharmacovigilance Risk Assessment Committee maintains, by majority, its recommendation and consequently the marketing authorisations for all medicinal products referred to in Annex I should be suspended. These conclusions are without prejudice to the conclusions of the procedure under Article 107i of Directive 2001/83/EC. In the procedure under Article 107i of Directive 2001/83/EC additional data has been included that was not available when the PRAC issued its recommendation on the referral in accordance with Article 31 of Directive 2001/83/EC in June 2013 and therefore could not be considered in the re-examination of the latter in October 2013.

Appendix 1

Divergent positions to PRAC recommendation

Article 31 of Directive 2001/83/EC resulting from pharmacovigilance data

Procedure No: EMEA/H/A-31/1348

Solutions for infusion containing hydroxyethyl starch

Divergent statement

The following members of PRAC did not agree with the PRAC's Recommendation on the Article 31 referral re-examination resulting from pharmacovigilance data for solutions for infusion containing hydroxyethyl starch based on the following reasons:

1. The results indicating harm in patients with severe sepsis may not be extrapolated to other patient groups and indications:
    a. The expert group unanimously agreed that the increased risks of renal events and the increased mortality observed in patients with sepsis and the critically ill cannot be directly extrapolated to perioperative setting or trauma setting or to any other clinical setting
    b. In sepsis the most important pathophysiological mechanism behind hypovolemia is a severe generalised inflammatory activation with vasodilatation and capillary leakage. In elective surgery and trauma the main mechanism is mostly a direct loss of blood volume, with an inflammatory activation of substantially lower magnitude and consequently substantially less capillary leakage.
    c. In sepsis no clinical meaningful volume-sparing effect is seen while in elective surgery there is a clinically meaningful volume-sparing effect.
    d. The relative increase in mortality risk is substantially lower in critically ill patients in general (CHEST study) compared to the studies on severe sepsis, suggesting potential effect-modification by patient subset.
2. Treatment of hypovolemia is symptomatic, aiming to resolve the immediate threat to life and vital organ function. The patients underlying condition (such as severe infection, trauma, reason for surgery) is, however, the main determinant for long-term survival. Improvement in short-term hemodynamic end-points is therefore clinically relevant and should contribute to the benefit-risk assessment.
3. Benefit in elective surgery in terms of volume-sparing effect and hemodynamic endpoints has been shown. Harm as identified in septic patients cannot be extrapolated to elective surgical patients. The benefit-risk relation in elective surgery is therefore considered favourable based on currently available data.
4. In trauma patients there is a rationale for colloids in specific settings, but limited data on clinical benefit. HES is still present in recently updated European guidelines on management of bleeding following major trauma. The benefit-risk relation in trauma is therefore considered favourable based on currently available data despite the uncertainty.
5. Alternative synthetic colloids (albumin and gelatin) are notably less well studied and there are data suggesting that they may not be considered as better alternatives. Albumin has possibly unfavourable outcome for some patient categories. Therefore, switching to alternative synthetic colloid therapy is a concern.
6. Additional measures should be proposed to further minimize the identified and potential risks.

Due to the above mentioned arguments the below mentioned PRAC delegates consider the benefit/risk balance of Hydroxyethyl starch (HES) positive for specified subgroups justifying the maintenance of the marketing authorisations of all HES-containing medicinal products subject to variation and conditions to the marketing authorisations.

PRAC members expressing a divergent position:

Jane Ahlqvist Rastad	10 October 2013	Signature: ……………………………..
Margarida Guimarães (PT)	10 October 2013	Signature: ……………………………..
Jolanta Gulbinovic (LT)	10 October 2013	Signature: ……………………………..
Herve Le Louet	10 October 2013	Signature: ……………………………..
Eva Jirsovà (CZ)	10 October 2013	Signature: ……………………………..
Tatiana Magálová (SK)	10 October 2013	Signature: ……………………………..
Isabelle Robine (FR)	10 October 2013	Signature: ……………………………..
Maia Uusküla (ET)	10 October 2013	Signature: ……………………………..
Qun-Ying Yue (SE)	10 October 2013	Signature: ……………………………..
Andis Lacis (LV)	10 October 2013	Signature: ……………………………..

Article 31 of Directive 2001/83/EC resulting from pharmacovigilance data

Procedure No: EMEA/H/A-31/1348

Solutions for infusion containing hydroxyethyl starch

Divergent statement

The following member of PRAC did not agree with the PRAC's Recommendation on the Article 31 referral resulting from pharmacovigilance data for solutions for infusion containing hydroxyethyl starch based on the following reasons:

1. Treatment of hypovolemia is symptomatic, aiming to resolve the immediate threat to life and vital organ function. The patients underlying condition (such as severe infection, trauma, reason for surgery) is, however, the main determinant for long-term survival. Improvement in short-term hemodynamic end-points is clinically relevant and should contribute to the benefit-risk assessment. The benefit-risk in elective surgery can be considered favourable based on available data. In this indication HES can be acceptable for short term use.

2. Additional measures should be proposed to further minimize the identified and potential risks.

Due to the above mentioned arguments the below mentioned PRAC delegates consider the benefit/risk balance of Hydroxyethyl starch (HES) positive for specified subgroups justifying the maintenance of the marketing authorisations of all HES-containing medicinal products subject to variation and conditions to the marketing authorisations.

PRAC members expressing a divergent position:

Sabine Straus (NL)	10 October 2013	Signature: ……………………………...

11 November 2013
EMA/667553/2013

Assessment report for solutions for infusion containing hydroxyethyl starch

Procedure under Article 107i of Directive 2001/83/EC

Procedure number: EMEA/H/A-107i/1376

Assessment Report as adopted by the PRAC with all information of a confidential nature deleted.

7 Westferry Circus ● Canary Wharf ● London E14 4HB ● United Kingdom
Telephone +44 (0)20 7418 8400 **Facsimile** +44 (0)20 7523 7051
E-mail info@ema.europa.eu **Website** www.ema.europa.eu

An agency of the European Union

© European Medicines Agency, 2013. Reproduction is authorised provided the source is acknowledged.

Table of contents

1. Background information on the procedure 3

2. Scientific discussion 3

2.1. Clinical aspects 4

2.1.1. Clinical safety.. 4

2.1.2. Clinical efficacy 16

2.2. Risk minimisation activities, including communication 22

2.3. Product information 23

3. Overall discussion and benefit-risk assessment 26

4. Action plan and communication plan 28

5. Conclusion and grounds for the recommendation.................................. 28

1. Background information on the procedure

On 27 June 2013, the United Kingdom's Medicines and Healthcare Products Regulatory Agency (MHRA) notified the Member States, European Medicines Agency and the European Commission in accordance with Article 107i of Directive 2001/83/EC, of its consideration of the need to suspend the marketing authorisations for Hydroxyethyl starch (HES) solutions for infusion in the UK.

The decision of the MHRA was based on evidence from randomised controlled clinical trials where HES solutions for infusion, when compared to crystalloids, were associated with an increased risk of mortality and renal replacement therapy (RRT) or renal failure as well as other serious adverse reactions in patients with sepsis and in the critically ill. The MHRA considered there is a lack of evidence to provide reassurance that these risks are not present in other clinical settings and there is little evidence that HES provides clinical benefit over crystalloids in any setting. Therefore, given the evidence for harm associated with HES products and the continued significant used of these products, the MHRA considered the need of urgent national action.

The PRAC was requested to assess the matter and to make a recommendation under the provisions of Article 107i of Directive 2001/83/EC to the Human Co-ordination Group for Mutual Recognition and Decentralised Procedures (CMDh) on any measures necessary to ensure the safe and effective use, and on whether the marketing authorisation for these products should be maintained, varied, suspended or withdrawn.

After reviewing all the available data submitted by the Marketing Authorisation Holders (MAHs) and by others Stakeholders, the PRAC adopted a recommendation on 10 October 2013.

2. Scientific discussion

Hydroxyethyl starch (HES) solutions for infusion include products with starch derived from potato or corn (waxy maize), with different molecular weights (mainly 130kD; 200kD) and substitution ratios (the number of hydroxyethyl groups per glucose molecule). HES containing solutions for infusion are authorised worldwide including all EU and EEA countries with the main indication for the treatment and prophylaxis of hypovolaemia and hypovolemic shock.

Concerns with regards to HES were previously considered by the Pharmacovigilance Working Party in September 2008 and by the PRAC in November 2012 on the basis of results of several studies, some of which showed an increased risk for RRT or acute renal failure. Published studies (6S[1], VISEP[2]) including a recent one (6S) provided further data supporting an increased risk of mortality at day 90 and RRT in patients with sepsis. Furthermore, the higher risk for RRT was shown in another recently published large clinical trial in intensive care unit (ICU) patients (CHEST[3]). Mortality difference was not confirmed, however, the study enrolled a broad mixture of patients with on average lower baseline mortality risk admitted to intensive care units. Although some limitations of the studies were raised, the data which were collected from these large randomised clinical trials were considered solid enough to indicate a potential harm associated with HES.

The EMA's Pharmacovigilance Risk Assessment Committee (PRAC) had recommended in June 2013 that HES solutions for infusion be suspended in the European Union (EU), following an assessment of

[1] Perner A, Haase N, Guttormsen AB *et al*. Hydroxyethyl starch 130/0.42 versus ringer's acetate in severe sepsis. *N Engl J* 2012 Jul 12;367(2):124-34
[2] Brunkhorst FM, Engel C, Bloos F *et al*. Intensive Insulin Therapy and Pentastarch Resuscitation in Severe Sepsis. *N Engl J Med* 2008; 358(2):125-39
[3] Myburgh J, Finder S, Bellomo R *et al*. Hydroxyethyl starch or saline for fluid resuscitation in intensive care. *N Engl J Med* 2012; 367:1901-11

available data which concluded that their benefits do not outweigh the risks of RRT or acute renal failure and mortality. A number of marketing-authorisation holders exercised their legal right to request a re-examination of the recommendation, and this procedure was considered by the PRAC separately.

In the meantime, on 27 June 2013 the UK's MHRA initiated an urgent union procedure under Article 107i of Directive 2001/83/EC, following its consideration of the need to suspend the marketing authorisations for HES solutions for infusion in the UK. This review procedure ran separately but in parallel with the re-examination of the PRAC's June 2013 Article 31 of Directive 2001/83/EC recommendation. In the scope of the Article 107i of Directive 2001/83/EC, new data which were not available in the referral under Article 31 of Directive 2001/83/EC were considered.

2.1. Clinical aspects

2.1.1. Clinical safety

In support of the clinical safety of HES solutions for infusion, published data on the risks of mortality and renal injury associated with HES solutions for infusion, in critically ill and ICU patients (including sepsis, trauma, surgical and non-surgical ICU patients) and surgical patients were provided. These data included those previously considered by the PRAC as part of the assessment of the Article 31 referral. It also included new available data including randomised clinical studies, meta-analysis of clinical studies, post-marketing experience, responses submitted by the marketing authorisation holders (MAHs) in writing and at oral explanations on the safety and efficacy of hydroxyethyl starch containing products for solutions for infusion, as well as stakeholder submissions in particular with regards to the risk of mortality and renal failure. Only a summary of relevant data is presented hereafter[4].

VISEP, 6S and CHEST trials limitations

The PRAC previously considered the VISEP, 6S and CHEST trials and acknowledged the limitations of the studies in the context of the referral under Article 31 of Directive 2001/83/EC. Although some limitations were identified, the PRAC concluded that the studies were well-designed and adequately powered to show an increased risk of mortality (6S, VISEP) in patients with sepsis and a risk of renal replacement therapy or renal failure in patients with sepsis and those who were critically ill (6S, VISEP, CHEST).However, the MAHs further expressed concerns over the design and execution of the VISEP, 6S, and CHEST trials, and the possibility that this may have influenced the results and cast doubt on the strength of the evidence. The main points raised by the MAHs were patients starting study treatment several hours after admission to ICU and possibly being haemodynamically stable at randomisation; patients subsequently randomised to the crystalloid arm had received initial treatment with colloids; and that there was the lack of defined criteria for starting RRT in the protocols.

The PRAC has again carefully considered the arguments presented by the MAHs. As published in a letter to the editor of the Scandinavian Journal of Trauma, Resuscitation and Emergency Medicine (Chappell and Jacob), the authors of the 6S study state that the "*24 hour window from diagnosis of severe sepsis resembles clinical practice, where fluid resuscitation in septic patients is initiated without waiting for the results of new blood samples to confirm the diagnosis of severe sepsis*". Forty-nine percent (49%) of patients in 6S received colloids in the 24 hours prior to randomisation, "*but the*

[4] This report details the assessment of the Article 107i of Directive 2001/83/EC procedure. Details on the assessment report for the Article 31 procedure that was conducted separately but in parallel to this procedure can be found in the respective report.

clinician judged that fluid resuscitation was still needed as this was an inclusion criterion". Therefore, the MAHs' suggestion that patients were already haemodynamically stable and no longer hypovolaemic at randomisation was not supported by the PRAC.

Regarding the observation that in some of the studies patients in the crystalloid group had already received colloids prior to randomisation, the PRAC highlighted that early exposure to HES would logically be expected to reduce the observed differences between the treatment arms, if it had any effect at all.

Although there was no trigger for starting and terminating RRT specified in the CHEST study protocol the suggestion that this could bias the results in favour of one intervention or another is not accepted, as the physicians were blinded to the treatment in CHEST and similarly in 6S.

According to the authors of the 6S study, kidney failure without RRT was not a 'clear' or 'absolute' contraindication for HES. It is important to note that the increased risk of death with HES was independent of kidney failure at inclusion in the pre-planned subgroup analysis.

Overall the concerns over the study design and execution of VISEP, 6S, and CHEST raised by the MAHs do not constitute major limitations of these studies, and do not alter the assessment of these data as robust evidence of increased renal dysfunction and mortality associated with the use of HES from large randomised clinical trials in septic and critically ill patients. Furthermore, the PRAC has previously considered and assessed these data as part of the referral procedure under Article 31 of Directive 2001/83/EC and these arguments did not alter the conclusion reached on the risks of renal injury and mortality associated with HES in sepsis and critically ill patients.

Risk of mortality

<u>Safety data from clinical trials</u>

- CRYSTAL trial[5]

The CRYSTAL trial results were already considered by the PRAC in the context of the referral procedure under Article 31 of Directive 2001/83/EC. However, the draft manuscript intended for publication and the protocol of this study was made available by the principal investigator to the PRAC in the context of the referral under Article 107i of Directive 2001/83/EC. These were not taken into consideration when the PRAC issued its recommendation on the referral in accordance with Article 31 of Directive 2001/83/EC in June 2013 and therefore could not also be considered in the re-examination of the latter in October 2013.

The results of the study are presented and discussed hereafter.

Protocol

The CRYSTAL study was an investigator-initiated study comparing colloid and crystalloid therapies for fluid resuscitation. It was a multinational, randomised, controlled parallel group trial. Patients included were those hospitalised in an ICU who needed fluid resuscitation according to the physician in charge of the patient, and were randomised to receive either crystalloids (0.9% saline, hypertonic saline or Ringer Lactate) or colloids (gelatins, starch solutions (including HES) and albumin).

Treatment was then chosen from whatever was available at local hospitals within this framework. According to the protocol, "*those patients whose physician believes they should receive colloids like albumin will receive them and not be part of the study*."

[5] Annane D. et al. CRISTAL: Colloids Compared to Crystalloids in Fluid Resuscitation of Critically Ill Patients: A Multinational Randomised Controlled Trial. NCT00318942. Available on: http://clinicaltrials.gov/ct2/show/NCT00318942

The study was not blinded as according to the protocol "*double blind seems unfeasible as the time window for inclusion is extremely short (treatment should be available promptly at bedside) and the amounts of volume replacement for all ICU stay could not be predicted a priori.*"

The primary endpoint was 28-day mortality rate. Eleven secondary endpoints were listed, first of which was 90-day mortality rate. All the secondary endpoints were subjective.

Block randomisation stratified by site and diagnosis, (1) trauma or haemorrhage, (2) sepsis, (3) other diagnoses was used.

A total of 2857 patients, 1414 in the colloids arm and 1443 in the crystalloids arm, were recruited over the nine years period of the study.

Prior to ICU admission, 585 and 685 patients in the colloids and crystalloids arms, respectively, received a median volume of 1000 ml of colloids [Interquartile range (IQR): 500-2000]; and 526 and 402 patients in the colloids and crystalloids arms, respectively, received a median volume of 950 ml of crystalloids [IQR: 500-1000].

Severe sepsis was the main diagnosis upon admission in both arms.

Results – Interim analyses

The study was stopped on the basis of 706 observed deaths from 2,612 enrolled patients before the end of the study. The boundaries of the sequential plan were drawn to demonstrate an absolute difference of 5% in 28-day mortality rate between the two treatment arms, assuming a 20% mortality rate in the crystalloids group, and with alpha and beta of 5% and 10% respectively. When a boundary is crossed, the enrolments in the study may be stopped, and the conclusion depends on which boundary has been crossed. The conclusion was that there was no statistical difference in 28-day mortality between those groups.

At Day 28, they were 359/1414 (25.4%) deaths in the colloids arm and 390/1443 (27.0%) in the crystalloids arm (RR =0.96; 95%CI: 0.88-1.04; P=0.26).

At Day 90, they were 434/1414 (30.7%) deaths in the colloids arm and 493/1443 (34.2%) in the crystalloids arm (RR =0.92; 95%CI: 0.86-0.99; P=0.03).

There was significant heterogeneity in mortality rates and treatment effect across centres.

Conclusion

The authors of the study concluded that "*among ICU patients with hypovolemia, the use of colloids vs crystalloids did not result in a significant difference in 28-day mortality. Although 90-day mortality was lower among patients receiving colloids, this finding should be considered exploratory and requires further study before reaching conclusions about efficacy.*"

The PRAC acknowledged a number of limitations of the study such as the fact that HES is only part of one of the arms and therefore the results are to be taken with caution. Other limitations included the possibility of excluding patients if their physicians considered them to need colloid therapy, which has an impact on the ability to generalise the results and the likelihood that there was a shift in clinical practice during the long duration of this pragmatic trial. The PRAC was of the opinion that the rationale for not blinding the study was clear and understandable.

The PRAC noted that protocol deviations occurred in both arms of the study. In the colloids arm, these included administration of normal saline 252/1414 (17.8%), Ringers lactate 88/1414 (6.2%), and hypertonic saline 19/1414 (1.3%). In the crystalloid arm, these included administration of gelatins 24/1443 (1.7%) and hydroxyethyl starch 69/1443 (4.8%). Although some limitations were highlighted

as described above, the PRAC noted that this randomised controlled trial favours colloids (including HES) for volume resuscitation due to lower volume needed, more ventilator- and vasopressors- free days. In addition, the PRAC noted that colloids (including HES) did not increase the risk of mortality at day-28 and day-90. The PRAC therefore acknowledged the results of this study which shows no risk of mortality associated with the use of HES but considered that given the limitations of this study its findings could not negate the findings from 6S and VISEP studies that had shown an increased risk of mortality in critically ill patients.

- BaSES[6]

The BaSES (Basel Starch Evaluation in Sepsis) trial results are further discussed hereafter.

Protocol

The purpose of this study was to determine whether initial infusion therapy with HES and Ringer's lactate in septic patients reduces ICU and hospital length of stay without impairment of renal function.

This trial was double-blind, randomised and included 241 patients with sepsis, severe sepsis and septic shock. Patients received 1000 ml study infusion (i.e. either HES 130/0.4 or saline) followed by 1000 ml Ringer's lactate, alternating these treatments up to a total volume of 50 ml/kg per day of study infusion in the first five days of intensive care treatment.

Primary outcome measures were ICU length of stay, hospital length of stay and mortality (ICU, hospital and one year). Secondary outcome measures were kidney function at ICU discharge and after one year, and lung function during ICU stay (see section "*risk of renal injury"*).

In total, 241 patients (2 ICUs in one hospital) with sepsis were resuscitated with 6% HES 130/0.4 in saline or isotonic saline for 5 days.

Results and conclusion

The difference in ICU length of stay was nearly 24 hours in favour of HES, although this difference was not statistically significant. Total length of hospital stay was statistically significantly reduced with HES.

The RR (95% CI) for mortality did not differ between HES and saline (RR 0.97, 95% CI 0.65-1.45). No differences were found in mortality between the two groups.

The authors of the study concluded that with strict alternating intravenous application following a hemodynamic protocol, administration of 6%HES (130/0.4) results in no significant reduction of the amount of resuscitation fluid, and no increase in the risk of acute kidney injury (see section "risk of renal injury") or mortality, but did decrease ICU and hospital length of stay compared with Ringer's lactate.

The PRAC noted the small sample size of this study to detect an increased risk of mortality. A meta-analysis by Haase et al, 2013 which included the results from the BaSES found an increased risk of renal replacement therapy for HES compared with crystalloids.

[6] Siegemund M. Firstly presented at European Society of Anaesthesiology conference 2012. Basel Study for Evaluation of Starch (130;0.4) Infusion in Septic Patients:BaSES (130;0.4) Trial, listed at http://clinicaltrials.gov/show/NCT00273728

Safety data from meta-analysis or analysis

- Wiedermann CJ and Joannidis M 2012[7]

Wiedermann CJ and Joannidis M 2012 published an updated version of a previous meta-analysis, with the FIRST[8] and CRYSTMAS[9] studies included as new additions. These two studies provide more than 50% of the weight in the analysis. Overall, 13 studies reporting 1,131 participants met the inclusion criteria.

The results showed a pooled RR for mortality of 1.14 (CI 0.89 to 1.46). However, publication bias favouring HES was detected (p=0.038), and after adjustment the RR for mortality was 1.25 (CI 0.98 to 1.58; p=0.069). No heterogeneity was found (I2, 0%; CI, 0% to 32%; p = 0.81).

The review of Wiedermann and Joannidis 2012 have shown that HES does not negatively affect mortality and renal function in surgical patients, although the observation periods were usually too short to provide longterm data on mortality.

- M.A.R.C.O meta-analysis

The meta-analysis of trials in surgical settings initiated by one of the MAHs and conducted by the clinical research organisation M.A.R.C.O, that had already been considered in the context of the referral under Article 31 of Directive 2001/83/EC, was provided.

The endpoints evaluated were total blood loss, frequency of transfusions, reoperation, kidney injury and mortality. Thirty-six (36) peer reviewed articles were considered in a period of 28 years. Studies examined lower and higher molecular weight (130 – 450) HES products with molecular substitution ratios between 0.4 – 0.7.

No statistically significant difference in mortality was identified for HES products relative to comparators (crystalloid, albumin, gelatin) in this analysis.

Table 1 - Mortality – Combined HES (0.4-0.7) vs. Combined Comparators

	Combined HES	Combined Comparators
Mortality	0.5% (6/1235)	0.7% (9/1262)
Common Risk Ratio (HES over comparators)	0.80; p=0.65	

The low numbers of events mortality observed in direct comparisons of HES with crystalloid may reflect the short length of follow up and/or small trial size. The PRAC noted that very few events of mortality were observed in the studies included and that the studies included in this meta-analysis had not been designed nor powered to investigate effects on mortality.

Safety data from an analysis: ARISCAT by Canet J *et al*. 2013[10]

The prospectively compiled database of the ARISCAT study of a large, representative cohort of general surgical patients was reanalysed to compare outcomes according to whether intraoperative colloids

[7] Wiedermann CJ, Joannidis M. Mortality after hydroxyethyl starch 130/0.4 infusion: an updated meta-analysis of randomized trials. Swiss Med Wkly 2012 Jul 30;142:w13656

[8] James MF, Michell WL, Joubert IA, et al. Resuscitation with hydroxyethyl starch improves renal function and lactate clearance in penetrating trauma in a randomized controlled study: the FIRST trial (Fluids in Resuscitation of Severe Trauma). Br J Anaesth 2011; 107(5):693-702

[9] Guidet B, Martinet O, Boulain T, et al: Assessment of hemodynamic efficacy and safety of 6% hydroxyethylstarch 130/0.4 vs. 0.9% NaCl fluid replacement in patients with severe sepsis: The CRYSTMAS study. Crit Care 2012; 16:R94

[10] Canet J, Sabaté S, Mazo V. Effects of intraoperative colloid administration on outcome in a population-based general surgical cohort: a propensity score analysis. Minerva Anestesiol. 2013 Aug;79(8):891-905. Epub 2013 May 6

were administered or not; a propensity score was used to adjust for potential confounders. The primary outcomes were major postoperative complications. Secondary outcomes were postoperative hospital-free days within 90 days and mortality at 30 and 90 days. In a retrospective survey each centre's data collectors were asked to estimate the proportions of the different colloids administered during the study period.

Of 2462 patients analysed, 556 (22.6%) received some type of colloid intraoperatively. The median (25th-75th percentile) of total fluids administered was significantly higher in patients receiving colloids (10.0 [6.9-14.1] mL·kg-1·h-1 vs. 8.8 [6.0-12.8] mL·kg-1·h-1 for patients not receiving colloids; P<0.01). The median volume of colloids administered was 7.5 (6.3-10.4) mL·kg-1. An estimated 75.7% of the patients received third-generation hydroxyethyl starches (130/0.4). Patients receiving colloids had 1.9 fewer postoperative hospital-free days (P<0.006). There were no significant differences in 30- and 90-day mortality.

The PRAC acknowledged potential methodological limitations of this observational non-randomized study and that the results should be interpreted with caution.

- Zampieri FG *et al*. 2013[11]

Zampieri FG *et al.* 2013 performed a retrospective observational analysis including 894 patients submitted to oncologic surgery. A total of 385 propensity-matched patients remained in the analysis: 97 in the no-hydroxyethyl starch group and 288 in the hydroxyethyl starch group. There were no differences between the groups in the need for other blood products, intensive care unit length of stay or mortality. The PRAC noted that this was a retrospective observational study and so heterogeneity and selection bias are limitations for this type of study.

<u>Safety data from the Rational Fluid Therapy in Germany (RaFTinG) clinical registry[12]</u>

One of the MAHs submitted the RaFTinG clinical registry summary report.

The RaFTinG clinical registry was a prospective non-interventional ICU registry including 65 German ICUs. In total, 4545 patients were documented in the presented data. In the analysis presented, the primary aim was to evaluate the impact of different colloids (HES 200, HES 130/0.4, HES 130/0.42, and gelatin) and crystalloids on 90-day mortality (defined as ICU stay plus 90 days after discharge) by Cox regression analysis. The impact of resuscitation fluid type on secondary endpoints of ICU mortality, renal replacement therapy, and acute kidney injury was evaluated by multiple logistic regression.

Conditions present at admission which might have had an influence on endpoint incidence were included in the regression model to adjust for imbalances of the cohorts. The following parameters were used for adjustment:

- Age
- Sex
- Mortality risk based on SAPS II and Apache II on admission
- Chronic renal failure on admission
- Sepsis on admission

---

[11] Zampieri FG, Ranzani OT, Morato PF, Campos PP, Caruso P. Effect of intraoperative HES 6% 130/0.4 on the need for blood transfusion after major oncologic surgery: a propensity-matched analysis. Clinics (Sao Paulo). 2013 Apr;68(4). 501-509

[12] Rational Fluid Therapy in Germany (RaFTinG). Available on ClinicalTrials.gov (NCT01122277) last updated on 07 July 2011: http://clinicaltrials.gov/ct2/show/NCT01122277?term=NCT01122277&rank=1

- Cumulative units packed red blood cells*
- Cumulative fluid balance
- Average colloid/crystalloid dose/day*
- Standardised AUC of SOFA score*
- Vasopressor use > 0.6 mg/h

*until onset of event investigated

During ICU stay 54.6% of the study patients received only crystalloids and 45.4% received colloids. Patients in the colloid group had more severe illness on admission (as measured by SAPS II and APACHE II scores) and were more likely to have severe sepsis (69.1% for colloids group, 64.6% for crystalloids only). The cumulative fluid balance was comparable for patients treated with or without colloids. Packed red blood cell (PRBC) and non-PRBC transfusion were similar among patients treated with or without colloids.

Overall mortality for all patients was 9.6% on ICU and 16.0% for 90-day-mortality. Unadjusted ICU mortality was higher for patients treated with colloids compared with those treated with crystalloids only. Compared with crystalloids only the mortality risk was significantly higher for gelatin or HES 130/0.42 and HES 200 infusion. HES 130/0.4 infusion had no independent effect on ICU or 90-day mortality.

In sub-group analyses, the adjusted risks of ICU and 90-day mortality were analysed separately for surgical vs. medical patients and patients with or without severe sepsis on admission, respectively. Since HES 130/0.4 was the most commonly administered colloid (n=1142), subcohort analyses were only performed for patients who received HES 130/0.4 compared to those who solely received crystalloids. The adjusted risk of ICU mortality was similar for patients treated with HES 130/0.4 or solely crystalloids in the subcohort of patients with severe sepsis on admission. In the subcohort of patients admitted without severe sepsis, the adjusted risk of ICU mortality was lower for patients treated with HES 130/0.4 as compared to those receiving only crystalloids. For surgical patients, the adjusted risk of ICU mortality tended to be lower for patients treated with HES 130/0.4 as compared to solely crystalloids, whereas there was no difference in medical patients. This effect could not be observed for medical patients.

Therefore, the results presented from RaFTinG showed no statistically significant differences between patients treated with crystalloids only (n=2482) and those treated with colloids (all HES preparations and gelatin, n=2063) for the endpoints of 90-day mortality.

Non-significant trends favouring HES 130/0.4 were reported for ICU mortality (OR 0.858, 95% CI 0. 560 – 1.315), and 90-day mortality (HR 0.873, 95 % CI 0.695 – 1.097). The numbers of patients included in this subgroup analysis for crystalloids only (n=1885) and HES 130/0.4 (n=1127) differ from the numbers of patients assessed for baseline characteristics; crystalloids only (n=2482), HES 130/0.4 (n=1142). The reason for exclusion of patients, including a large number of patients from the crystalloid only group, from the subgroup analysis is not apparent.

Differences favouring HES 130/0.4 are reported for ICU mortality when only surgical patients or only patients without severe sepsis are considered and renal failure according to RIFLE. Risk estimates and details of the numbers of patients included when surgical admission patients only or patients without severe sepsis on admission only are considered are not provided.

Overall the results from RaFTinG do not show and increased risk for the mortality in patients admitted to ICU receiving colloids compared with crystalloids only, or in a subgroup of ICU patients receiving

HES 130/0.4 compared with crystalloids only. The PRAC noted, however, that this was an observational study and only limited conclusions can be drawn with respective to relative benefits and risks of treatment as it is not possible to exclude that treatment bias or differences between baseline characteristics between the treated groups could have had an impact on the results.

Risk of renal injury

Safety data from clinical trials

- BaSES

The results of the BaSES trial showed no differences in incidences of acute kidney injury (AKI) and RRT between the two groups. No patients required RRT after one year (cited according to Haase *et al.* 2013). However, given the limited number of patients included in this study these results need further confirmation.

Safety data from prospective and retrospective observational studies

- Sümpelmann R *et al*. 2011 (PASS study)[13]

PASS is a European multicentre (11 centres in 5 countries) open prospective observational postauthorisation safety study to evaluate the use of HES 130/0.42/6:1 in normal saline (ns-HES, 629 children, 2006-2009) or in a balanced electrolyte solution (bal-HES, n=475) in 1130 children up to 12 years undergoing surgery. Data were collected prospectively using a standardised case report form. In roughly one third of patients, biochemical changes were also assessed.

Mean infused volume was approximately 10 (0.8-50) ml/kg. Mean duration of observation was short, 6 ± 14 (0.1– 216) hours. Sixty (60) adverse events (3.5%) were reported in 40 patients out of 1130. All cases resolved until the end of the study. Mild to moderate adverse drug reactions (hemodilution, abnormal acid-base balance, low blood pressure) were reported in 14 (1.2%) patients. No anaphylactoid reactions, clotting disorders or renal failure was observed. There were significantly fewer complications (adverse events (AE) and drug reactions (ADR)) with HES in balanced electrolyte solution. For the AE/ADR rates, dose-response but no age relationships could be demonstrated.

The authors concluded that PASS is an audit of international intraoperative anaesthesiological practice in children. They consider that this study where moderate doses (< 20 ml/kg) were used with adherence to the contraindications (hypervolemia, renal failure, intracranial bleeding, severe hypernatremia or hyperchloremia, hypersensitivity to HES, severely impaired hepatic function, and congestive cardiac failure) supports the safety of these solutions.

The PRAC acknowledged that HES was used in both study arms and therefore no conclusions on beneficial or harmful effects of HES can be drawn. However, the PRAC noted that no serious adverse events related to HES occurred during the surgery, which may provide some reassurance that serious adverse effects do not occur very commonly because if this were the case a higher number of these events could be expected to have been reported.

- Boussekey N *et al.* 2010[14]

This observational retrospective study included 363 patients hospitalised for more than 72 hours in an ICU. A hundred and sixty eight patients received HES during their stay and 195 did not. Patients'

[13] Sümpelmann R, Kretz FJ, Luntzer R, de Leeuw TG, Mixa V, Gäbler R, Eich C, Hollmann MW, Osthaus WA. Hydroxyethyl starch 130/0.42/6:1 for perioperative plasma volume replacement in 1130 children: results of an European prospective multicenter observational postauthorization safety study (PASS). Paediatr Anaesth. 2012 Apr;22(4):371-8. Epub 2011 Dec 23

[14] Boussekey N, Darmon R, Langlois J et al. Resuscitation with low volume hydroxyethylstarch 130 kDa/0.4 is not associated with acute kidney injury. Crit Care. 2010;14(2):R40. Epub 2010 Mar 18

baseline characteristics were recorded on admission and type and volume of fluid resuscitation during the first 3 weeks of ICU stay. Urine output, the risk of renal dysfunction, injury to the kidney, failure of kidney function, loss of kidney function and end-stage kidney disease (RIFLE) classification and sepsis related organ failure assessment (SOFA) score were documented over 3 weeks.

Patients in the HES group were more severely ill on admission but AKI incidence was similar (as well as ICU mortality). Urine output (P = 0.74), RIFLE classification (P = 0.44) and SOFA score (P = 0.23) was not different. However, HES volumes administered were low (763+/-593 ml during the first 48 hours).

As this is an observational retrospective study, the study has numerous biases. The HES group was more severely ill, the HES volumes administered were low, and attending physicians could have generated a systematic bias with no HES use when patients had renal failure or were at risk of AKI. In addition, the observational follow-up is short (21 days) and the number of patients included in the study is low. Moreover, the selection of these patients is unclear, but relevant selection bias cannot be ruled out as this constitutes only 45% of the initial population. Nonetheless, it is demonstrated that (contrary to similar kidney function), for example the frequency of hemofiltration (7% vs 0% and 8% vs 6% comparing HES to no-HES in patients with initially normal kidney function and RIFLE at risk) were numerically larger. However, this is not significantly different, given the relatively small numbers with consecutively low statistical power. The same applies to mortality and other clinically relevant outcomes. Overall, the data provided cannot exclude a risk of acute kidney injury.

The PRAC acknowledged the limitations of the study and considered that a new well-designed study should be performed.

<u>Safety data from meta-analysis and retrospective chart review</u>

- Martin *et al.* 2013[15]

The aim of this meta-analysis was to evaluate renal safety with the active substance of the latest generation of waxy maize-derived HES in surgical patients. The authors focused on prospective, randomised, controlled studies that documented clinically relevant variables with regard to renal effects of waxy maize-derived HES 130/0.40. Seventeen (17) studies that analysed patients (n = 1,230) undergoing a variety of surgical procedures were included.

For maximum serum creatinine values, the effect size estimate was 0.068 (95% CI: -0.227 to 0.362; P = 0.65). For calculated creatinine clearance values, pooled risk difference was 0.302 (95% CI: -0.098 to 0.703; P = 0.14). For incidence of acute renal failure, pooled risk difference was 0.0003 (95% CI : -0.018 to 0.019; P = 0.98). For incidence of renal replacement therapy, pooled risk difference was -0.003 (95% CI: -0.028 to 0.022; P = 0.85).

Therefore, the results showed no evidence for renal dysfunction caused by waxy-maize derived HES 130/0.4 in surgical patients. No significant difference for the effect of HES 130/0.4 on serum creatinine and no significant risk difference of acute renal failure as compared with respective controls, which included higher molecular weight HES 200, gelatin, human albumin, and crystalloid solutions were found. The PRAC noted that a limitation of this meta-analysis was that only 6 of the studies directly compared HES with crystalloids.

- Endo *et al.* 2012[16]

Endo *et al.* conducted an uncontrolled retrospective chart review to identify adult surgical patients with intraoperative blood loss of ≥1000 mL at a university hospital. AKI was defined as >50% increase in

---

[15] Martin C, Jacob M, Vicaut E, et al. Effect of waxy maize-derived hydroxyethyl starch 130/0.4 on renal function in surgical patients. Anesthesiology 2013;118(2):387-94

[16] Endo A, Uchino S, Iwai K, Saito K, Sanui M, Takinami M, Uezono S. Intraoperative hydroxyethyl starch 70/0.5 is not related to acute kidney injury in surgical patients: retrospective cohort study. Anesth Analg. 2012 Dec;115(6):1309-14

serum creatinine from the preoperative value within 7 days after the operation according to the RIFLE (Risk, Injury, Failure, Loss, or End-stage kidney disease) criteria. The study compared the incidence of AKI between patients with and without intraoperative HES administration. Multivariate logistic regression analysis and propensity score matching were also conducted to elucidate the impact of HES on postoperative AKI.

Among 14,332 surgical cases, 846 patients met the inclusion criteria. In patients given HES (a median dose of 1000 mL, n = 635), 12.9% developed AKI, compared with 16.6% (odd ratio: -3.7%, 95% CI: -1.7% to 9.1%) in patients without HES (n = 211). Multivariate logistic regression analysis showed that HES was not an independent risk factor for postoperative AKI (odds ratio: 0.76, 95% CI 0.48-1.21). Using the propensity score, 179 pairs were matched. In patients with HES, 12.3% developed AKI, compared with 14.5% in patients without HES (odd ratio: -2.2%, 95% CI: -4.9% to 9.3%).

The authors concluded that intraoperative 6% HES 70/0.5 in a low dose was not related to postoperative AKI in patients with major intraoperative blood loss. They however highlighted that randomised controlled trials are warranted to further evaluate the safety and efficacy of low-molecular-weight HES. The PRAC noted that this study had similar shortcomings to those of the study by Zampieri *et al.* 2013.

- Mutter TC *et al.* (2013)[17]

The latest Cochrane review by Mutter *et al*. 2013 focused on effects on kidney function of HES versus other fluid therapies in different patient populations.

Randomised controlled trials and quasi-RCTs in which HES was compared to an alternate fluid therapy for the prevention or treatment of effective intravascular volume depletion were included. Primary outcomes were renal replacement therapy (RRT), author-defined kidney failure and AKI as defined by the RIFLE criteria.

This review included 42 studies (11,399 patients) including 19 studies from the original review (2010), as well as 23 new studies. Fifteen studies were excluded from the original review (nine retracted from publication due to concerns about integrity of data and six lacking individual patient creatinine data for the calculation of RIFLE criteria). Overall, there was a significant increase in the need for RRT in the HES treated individuals compared to individuals treated with other fluid therapies (RR 1.31, 95% CI 1.16 to 1.49; 19 studies, 9857 patients) and the number with author-defined kidney failure (RR 1.59, 95% CI 1.26 to 2.00; 15 studies, 1361 patients). The RR of AKI based on RIFLE-F (failure) criteria also showed an increased risk of AKI in individuals treated with HES products (RR 1.14, 95% CI 1.01 to 1.30; 15 studies, 8402 participants). The risk of meeting urine output and creatinine based RIFLE-R (risk) criteria for AKI was in contrast in favour of HES therapies (RR 0.95, 95% CI 0.91 to 0.99; 20 studies, 8769 patients). However, when RIFLE-R urine output based outcomes were excluded as per study protocol, the direction of AKI risk again favoured the other fluid type, with a non-significant RR of AKI in HES treated patients (RR 1.05, 95% CI 0.97 to 1.14; 8445 patients). A more robust effect was seen for the RIFLE-I (injury) outcome, with a RR of AKI of 1.22 (95% CI 1.08 to 1.37; 8338 patients). No differences between subgroups for the RRT and RIFLE-F based outcomes were seen between sepsis versus non-sepsis patients, high molecular weight (MW) and degree of substitution (DS) versus low MW and DS (≥ 200 kDa and > 0.4 DS versus 130 kDa and 0.4 DS) HES solutions, or high versus low dose treatments (i.e. ≥ 2 L versus < 2 L). There were differences identified between sepsis versus non-sepsis subgroups for the RIFLE-R and RIFLE-I based outcomes only, which may reflect the differing renal response to fluid resuscitation in pre-renal versus sepsis-associated AKI.

[17] Mutter TC, Ruth CA, Dart AB. Hydroxyethyl starch (HES) versus other fluid therapies: effects on kidney function. Cochrane Database Syst Rev. 2013 Jul23;7

- M.A.R.C.O. meta-analysis

No statistically significant difference in incidence of acute kidney injury was identified for HES products relative to comparators (crystalloid, albumin, gelatin) in this analysis.

Only three studies comparing HES and crystalloid reported any events of acute kidney injury, and the criteria used for reporting these events were different in all three studies. The low numbers of events of kidney injury observed in direct comparisons of HES with crystalloid may reflect the short length of follow up and/or small trial size. Very few events of re-operations were observed, and therefore no conclusions can be drawn regarding these endpoints.

Table 2 - Acute Kidney Injury – Combined HES (0.4 – 0.7) vs. combined comparators

	Combined HES	Combined Comparators
AKI	1.3% (16/1235)	1.0% (13/1262)
Common Risk Ratio (HES over comparators)	1.21; p=0.59	

Most of the additional studies submitted by the MAHs as evidence for the quantification of risk of renal injury have involved very small samples and therefore it is not possible to draw any conclusion from these data (e.g. Hokema *et al*. 2011; Fenger-EriksenC *et al*. 2005[18]; Akkucuk FG *et al*. 2013[19] Alavi SM *et al*. 2012[20]).

Safety data from the Rational Fluid Therapy in Germany (RaFTinG) clinical registry

The results of this study showed that the crude incidence of acute kidney injury (AKI) as judged by RIFLE-F criterion was 12.3% for all patients. Compared to crystalloid infusion only, colloids did not alter the adjusted risk of AKI significantly. In addition, the results showed that during ICU stay 7.9% of all patients received renal replacement therapy. The crude incidence of RRT was considerably higher for patients treated with colloids (13.7%) as compared with those treated with crystalloids only (3.1%). Compared with solely treatment with crystalloids only, colloids per se did not modify the adjusted risk of renal replacement therapy

In sub-group analyses, receiving HES 130/0.4 significantly reduced this risk for renal failure according to RIFLE in the subcohort of surgical patients. This effect could not be observed for medical patients.

The results presented from RaFTinG show no statistically significant differences between patients treated with crystalloids only (n=2482) and those treated with colloids (all HES preparations and gelatin, n=2063) for the endpoints AKI and RRT. For the sub-group analysis comparing crystalloid only with HES 130/0.4 a significantly lower rate of AKI is reported for HES 130/0.4 compared with crystalloids only (OR 0.582, 95 % CI 0.386 – 0.877).

Non-significant trends favouring HES 130/0.4 were reported for RRT (OR 0.980, 95 % CI 0.599 – 1.606), ICU mortality (OR 0.858, 95% CI 0. 560 – 1.315). The numbers of patients included in this

[18] Fenger-Eriksen C, Hartig Rasmussen C, Kappel Jensen T, Anker-Moller E, Heslop J, Frokaer J, Tonnesen E. Renal effects of hypotensive anaesthesia in combination with acute normovolaemic haemodilution with hydroxyethyl starch 130/0.4 or isotonic saline. Acta Anaesthesiol Scand 2005 Aug;49(7):969-74
[19] Akkucuk FG, Kanbak M, Ayhan B, et al. The Effect of HES (130/0.4) Usage as the Priming Solution on Renal Function in Children Undergoing Cardiac Surgery. Ren Fail. 2013; 35(2):210-15
[20] Alavi SM, Ahmadi BB, Baharestani B, Babaei T. Comparison of the effects of gelatin, Ringer's solution and a modern hydroxyl ethyl starch solution after coronary artery bypass graft surgery. Cardiovasc J Afr 2012; 23: 428-431

subgroup analysis for crystalloids only (n=1885) and HES 130/0.4 (n=1127) differ from the numbers of patients assessed for baseline characteristics; crystalloids only (n=2482), HES 130/0.4 (n=1142). The reason for exclusion of patients, including a large number of patients from the crystalloid only group, from the subgroup analysis is not apparent.

Overall the results from RaFTinG do not show an increased risk for renal endpoints considered in patients admitted to ICU receiving colloids compared with crystalloids only, or for a subgroup of ICU patients receiving HES 130/0.4 compared with crystalloids only. As outlined previously, the observational nature of this study mean only limited conclusions can be drawn on relative benefits and risks of HES compared with crystalloids and other colloids. Furthermore, only limited data from the RaFTinG study were provided.

Spontaneous adverse drug reaction

EudraVigilance data

Data were extracted from EudraVigilance database for HES solutions for infusion.

A total of 408 case reports were retrieved, of which 31 case reports described a fatal outcome. The most frequent adverse reactions (ADR) reported were skin and subcutaneous disorders, respiratory, thoracic and mediastinal disorders, and general disorders and administration site conditions.

Because of the limitations of spontaneous ADR reports, little additional information is provided by these data on the benefit-risk balance and the renal and mortality risks of HES.

Stakeholders' submissions

The PRAC noted and assessed the stakeholders' submissions which comprised data from randomised trials, observational studies, database, retrospective cohort studies, meta-analyses and systematic review. There were 97 submissions received in total. These included 78 submissions where the benefit/risk balance of HES was claimed to be favourable in some groups of patients and the most frequent reason for that was related to the good clinical experience with the use of HES in intensive care.

Overall data provided as stakeholders' responses are consistent with the evidence that HES increases the risk of renal dysfunction in critically ill and septic patients. These are on-going randomised trial and a retrospective cohort study that do not appear to show statistically significant effects of renal injury or mortality for HES in surgical patients (e.g. Cochrane review by Mutter *et al*. 2013). No additional details on the characteristics of the database used in the retrospective cohort study are available at present. There are limitations of the data that were acknowledged. The submissions also included small studies where the use of HES in elective surgery was associated with no harm in this specific population. However, the PRAC noted that these studies were conducted in a limited number of patients.

Ad hoc expert meeting

The PRAC consulted an *ad hoc* expert group that was convened on 13 September 2013. The *ad hoc* expert group was requested to clarify whether from a clinical perspective, given the available data and taking into account the increased risk of renal events and the increased mortality, there are subpopulations of critically ill patients (defined as patients admitted to the ICU) for whom HES treatment remains beneficial.

Some experts considered that subpopulations of critically ill patients can be identified for whom treatment with HES remains beneficial such as all critically ill patients in emergency, all patients from ICU including limited trauma patients before surgery. However, the majority of experts considered that

based on the small and limited available studies it is not possible to identify any subpopulation in which the benefit would outweigh the risks.

Overall the expert group agreed that the benefit may exist in early in the course of treatment of severe hypovolaemia due to bleeding i.e. in the perioperative setting and disappearing fast as the patient becomes more stable.

The expert group unanimously agreed that the increased risk of renal events and the increased mortality observed in patients with sepsis and the critically ill could not be directly extrapolated to perioperative setting or trauma or to any other clinical setting. The experts agreed that the data available is not convincing however may suggest that the risks are lower in other settings than sepsis and critically ill patients.

The experts highlighted that the administration of HES to normovolaemic patients in certain trials was potentially an important issue.

The experts further highlighted that additional research on HES must be undertaken.

Conclusion on safety

The PRAC reviewed all safety data of hydroxyethyl starch, with a particular focus on the risk of mortality and renal injury. These included data that from clinical studies, meta-analyses of clinical studies, post-marketing experience, responses submitted by the marketing authorisation holders (MAHs) in writing and at oral explanations and stakeholders' submissions.

On the basis of the available data, in particular results from VISEP, 6S and CHEST studies, the PRAC concluded that HES is associated with an increased risk of mortality and renal failure in patients with sepsis, in critically ill and burn patients and that the benefits of HES do not outweigh the risks in these patient populations.

The PRAC noted the available data from studies in surgical and trauma patients and considered that although these studies were limited in size and duration of follow-up they did provide some reassurance that the risks of mortality and renal injury in surgical and trauma patients may be lower than those in the critically ill and patients. Although the mechanisms by which increased renal injury and mortality occur is not well established, it is possible that the degree of inflammatory processes seen in sepsis and critically ill patients is greater and associated with significant capillary leakage compared with other patient populations such as the perioperative setting after elective surgery or un-complicated trauma where the systematic inflammatory process and the extent of capillary leak may be lower.

The PRAC concluded that studies are needed to investigate the safety of HES in elective surgery and trauma patients.

2.1.2. Clinical efficacy

Clinical data in support of the clinical efficacy of HES solutions for infusion in surgical patients (including cardiac surgery and elective caesarean section under spinal anaesthesia), trauma patients including patients with severe haemorrhage were submitted. The data from the most relevant studies or analyses are summarised hereafter. The overview also includes data previously considered by the PRAC as part of the assessment of the referral under Article 31 of Directive 2001/83/EC.

Surgical patients

- M.A.R.C.O meta-analysis

In this meta-analysis, total blood loss referred to intraoperative blood loss and blood loss up to 24 hours after the end of the operation. The results showed the estimated difference in blood loss between HES and crystalloid from the seven trials analysed was – 0.09, in favour of HES (CI 95% -0.25 – 0.07). This was not statistically significant, and this is not of any clinical relevance. However, in this meta-analysis HES (130/0.4) was reported to be comparable in terms of total blood loss. The results are shown in the table 3.

Table 3 - Total Blood Loss – Tetrastarch 0.4 vs crystalloid

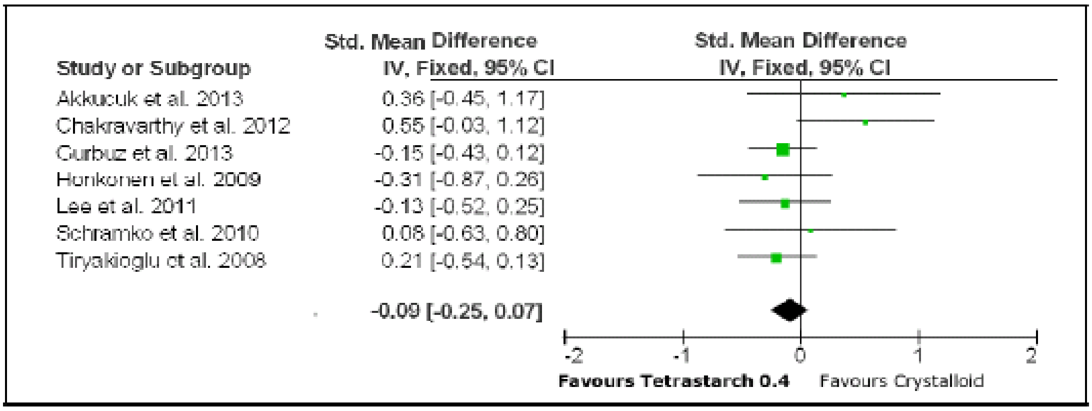

- Martin G *et al.* 2002[21]

Martin *et al.* (2002) studied the effects of different fluids on the coagulation profile in a prospective, randomised, double-blind trial of patients undergoing major elective surgery (non cardiac surgery) with an anticipated blood loss >500 mL. The effect of lactated Ringer's solution, 6% hetastarch (HES 550) in a balanced electrolyte solution, and 6% hetastarch in normal saline on coagulation as determined by thromboelastography was compared. A total of 90 patients undergoing elective non-cardiac surgery were enrolled with 30 patients in each group, study fluids were administered intraoperatively based on a fluid administration algorithm.

Ringer's lactate-treated patients developed a hypercoagulable state until post-operative day 1, while in patients treated with HES in normal saline a hypocoagulant effect was seen post-surgery, which was reversible within 24 hours. HES in the balanced electrolyte solution did not disturb coagulation after surgery and showed some, minor degree of a hypercoagulant state at 24 h after operation. HES treatment resulted in a significantly lower estimated blood loss. There was no difference in red blood cells, or blood product utilisation among the groups. HES administration resulted in a better coagulation profile as determined by thromboelastography in comparison to lactated Ringer's solution.

- Hamaji A *et al.* 2013[22]

Hamaji *et al.* (2013) reported a small randomised, controlled trial in 48 patients scheduled for hip arthroplasty with spinal anaesthesia. Patients received either a preload of 15 mL/kg HES 130/0.4 (n=24) or a preload of 30 mL/kg lactated Ringer's solution (n=24) before surgery.

[21] Martin G, nett-Guerrero E, Wakeling H, Mythen MG, el-Moalem H, Robertson K, Kucmeroski D, Gan TJ. A prospective, randomized comparison of thromboelastographic coagulation profile in patients receiving lactated Ringer's solution, 6% hetastarch in a balanced-saline vehicle, or 6% hetastarch in saline during major surgery. J Cardiothorac Vasc Anesth 2002; 16: 441-446

[22] Hamaji A, Hajjar L, Caiero M, Almeida J, Nakamura RE, Osawa EA, Fukushima J, Galas FR, Auler Junior JO. Volume replacement therapy during hip arthroplasty using hydroxyethyl starch (130/0.4) compared to lactated Ringer decreases allogeneic blood transfusion and postoperative infection. Rev Bras Anestesiol. 2013 Jan-Feb;63(1):27-35

Significantly fewer red blood cell transfusions were required in the HES group (17% HES vs. 46% Ringer's solution). In the Ringer's solution group 11 patients (46%) needed red blood cell transfusion compared with four patients (17%) in the HES group (p = 0.029). Postoperative infections were less frequent in the HES group (0) compared with the Ringer's group (4/27, 17%) (p=0.03). There were no significant differences between groups in mortality, hospital length of stay and clinical complications other than infection.

- Moretti EW *et al.* 2003[23]

Moretti *et al.* (2003) performed a prospective, blinded study in 90 patients (30 patients per group) undergoing major elective general, gynaecological, orthopaedic or urologic surgery with an anticipated blood loss of >500 mL and under maintenance of predefined haemodynamic targets. Patients were enrolled to one of 3 different resuscitation therapies requiring the administration of the following: 1301±1079 mL of 6% hetastarch in saline, 1448±759mL 6% hetastarch in balanced salt, or 5946±1909 mL lactated Ringer's solution. The colloid groups had significant smaller odds of nausea, nausea severity, emesis, antiemetic use, severe pain, periorbital oedema and double vision. The MAH claimed that the use of colloid led to improvement in the quality of postoperative outcome.

- Clinical trial (NCT01117649)[24]

Details of a randomised, controlled, double-blind, multicentre phase IV clinical trial (NCT01117649) was provided by one of the MAH. The aim of the study was to investigate the efficacy of target controlled fluid therapy in patients undergoing elective surgery of the pancreatic head comparing 6% HES 130/0.42 with 10% HES 130/0.42. A third group, serving as a control for descriptive analysis only, received balanced electrolyte solution.

The study was designed with an internal pilot phase to evaluate in a blinded manner the pooled variances of the primary variables (first endpoint: intra-operatively required amount of HES and second endpoint: time until fully on oral (solid) diet (days) of the HES-groups in order to re-evaluate sample size calculation. This blinded assessment of the pooled variances after recruitment of 63 patients showed that the variances were much larger than initially assumed, leading to much higher sample sizes than initially estimated. The study was terminated due to futility in accordance with the study protocol.

Evaluation of the intra-operatively required amount of HES was performed as total amount (ml) of investigational product as well as relative to body weight (ml/kg) and relative to duration of surgery (ml/h). Furthermore, total amount of fluid (i.e. including open label treatment during on-going surgery after maximal daily dose of HES had already been administered) was analysed again in ml, ml/kg and ml/h.

No substantial differences were observed between the evaluations in the full analysis set and in the intention to treat analysis.

The three groups showed significant differences based on higher doses (ml, ml/kg bodyweight and ml/h surgery) of balanced electrolyte solution compared with 6% HES and 10% HES (table 4). Using doses in ml, the analysis showed no relevant differences between 6% HES and 10% HES. When the 6% HES and 10% HES groups were combined, the comparison with balanced electrolyte showed significantly lower doses (ml, ml/kg, ml/h) compared with HES.

---

[23] Moretti EW, Robertson KM, el-Moalem H, Gan TJ. Intraoperative colloid administration reduces postoperative nausea and vomiting and improves postoperative outcomes compared with crystalloid administration. Anesth Analg 2003; 96: 611-7, table.
[24] Plasma Volume Replacement (PVR) Therapy With Colloid and Crystalloid Solutions. http://clinicaltrials.gov/show/NCT01117649

Table 4 - Amount of double-blind trial medication administered during surgery (Full Analysis Set)

Dosage [mL]	Tetraspan 10%	Tetraspan 6%	Sterofundin ISO
Number of patients	18	22	21
minimum	500	750	250
maximum	4000	4000	4000
median	2000.0	2000.0	2500.0
mean	2041.7	2215.9	2726.2
standard dev.	862.8	787.9	1024.4

The inclusion of the open label infusion after consumption of the blinded investigational product (i. e. after having administered the allowed maximal dose with respect to HES) did not alter the results as for the overall group comparison as described before for the full analysis set. In the *per* protocol set analysis the difference between the groups was less pronounced. The analysis was not altered for the combination of the two HES groups in comparison with balanced electrolyte.

For the second primary variable of time until fully on oral (solid) diet, no statistically significant group differences were detected. No statistically significant differences were observed with regard to intraoperative initial haemodynamic stabilisation. The intraoperative stroke volume (oesophageal Doppler Cardio Q, Pulse Contour Continuous Cardiac Output) and central venous pressure (CVP) were statistically significantly higher in the HES groups compared with balanced electrolyte.

- Yang J *et al.* 2011[25]

A randomised, open-label study examined the effects of different volume replacement regimens on inflammatory response and liver function in patients with hepatocellular carcinoma undergoing hepatectomy. Patients received 20% human albumin (group 1, HA group; n=30), 6% HES 130/0.4 (group 2, HES group; n=30), or crystalloids (lactated Ringer's (LR); group 3, LR group; n=30). Additional crystalloid solutions were administered to maintain central venous pressure (CVP) between 5 and 9 mmHg throughout the period in the ICU and a mean arterial pressure (MAP) of 60–80 mmHg throughout the remainder of the study period. Total bilirubin, alanine aminotransferase, and aspartate aminotransferase increased from baseline in all groups, and did not differ significantly between groups. Morbidity including postoperative complications and mortality during the study period in the HA group and the HES group were significantly better than in the LR group (no death were reported during the study and there were no cases of renal failure in any treatment group). The length of ICU stay was similar in all groups, but the duration of postoperative hospitalisation was significantly shorter in the HA and HES groups (HA: 7.6±0.9 days, HES: 7.6±0.6 days, and LR: 8.6±1.3 days, P <0.001). C-reactive protein levels were significantly lower in the HES group (P <0.001) indicating more favourable effects on the acute phase response.

The study by Yang *et al*. (2011) suggests benefit for HES in hepatectomy.

- Other studies in cardiac surgery, elective caesarean section under spinal anaesthesia, thermal injury and neurosurgery patients

A number of other studies in the cardiac surgery setting were provided (Alavi *et al*; Gondos *et al*[26]; Magder *et al*[27]; Verheij *et al*[28]; Sirvinskas *et al*; Kvalheim *et al*; Ali and Saleh; Gurbuz *et al*[29]; Lee *et*

[25] Yang J, Wang WT, Yan LN, Xu MQ, Yang JY. Alternatives to albumin administration in hepatocellular carcinoma patients undergoing hepatectomy: an open, randomized clinical trial of efficacy and safety. Chinese medical journal 2011; 124: 1458-1464

[26] Gondos T, Marjanek Z, Ulakcsai Z, Szabo Z, Bogar L, Karolyi M, Gartner B, Kiss K, Havas A, Futo J. Short-term effectiveness of different volume replacement therapies in postoperative hypovolaemic patients. European journal of anaesthesiology 2010; 27: 794-800

al[30]). The studies consistently show better haemodynamic outcomes, reduced time in ICU and reduced hospital stay for HES compared with crystalloid. The qualitative impression is that short term survival and renal dysfunction was similar for crystalloid and colloid in the studies presented. However the PRAC noted that long term survival data are not available and that the studies are of small size.

A number of studies from the literature in which volume preloading prior to elective caesarean section under spinal anaesthesia with either HES or crystalloid were compared for the prevention of hypotension were also submitted (French *et al.* 1999; Hasan *et al*. 2012; Madi-Jebara *et al*. 2008; Siddik *et al*. 2000; Riley *et al*. 1995; Ueyama *et al*. 1999). The studies demonstrated that there was less hypotension and a lesser requirement for sympathomimetic vasoconstrictor drugs in the groups treated with HES. The outcome for the neonate was no different regardless of whether the mother received colloid or crystalloid (as measured by APGAR score and foetal acidosis). The PRAC noted that the observation periods were short in all of the studies described and that long-term safety and mortality in mother and child were not assessed in any of the studies.

Studies in thermal injury and neurosurgery patients were submitted by the MAHs (Mokline *et al* 2012; Vlachou *et al.* 2010; Schiller *et al* 1997; Lindroos *et al.* 2013). The studies were limited by their small size, and by having designs not intended to provide information on long-term safety outcomes or the overall benefit-risk balance of HES.

Trauma patients

The MAHs made reference to studies which were already discussed by the PRAC in the context of the referral under Article 31 of Directive 2001/83/EC (i.e. James MF *et al.* 2011[31], Neff TA *et al.* 2003[32], Myburgh J *et al*. 2012[33], Perel P *et al.* 2011[34]).

- Ogilvie *et al.* (2010)[35]

Ogilvie *et al.* describes an observational study which examined death rates in trauma patients at a single centre in the USA and compared the rate for those who received standard of care with that for patients who received 6% hetastarch. The results showed that the overall mortality is significantly reduced after HES treatment compared to standard of care (5.2% vs 8.9% p= 0.0035).

The study is not a randomised trial so there is clear potential for bias and a disproportionate number of death occurred in the standard of care treatment group within 30 minutes after arrival at the trauma centre which might indicate a selection bias. The data are not sufficient robust data to allow for

[27] Magder S, Potter BJ, Varennes BD, Doucette S, Fergusson D; Canadian Critical Care Trials Group. Fluids after cardiac surgery: a pilot study of the use of colloids versus crystalloids. Crit Care Med 2010 Nov;38(11):2117-24
[28] Verheij J, Van LA, Beishuizen A, Christiaans HM, de J, Girbes AR, Wisselink W, Rauwerda JA, Huybregts MA, Groeneveld AB. Cardiac Response Is Greater for Colloid Than Saline Fluid Loading After Cardiac or Vascular Surgery. Intensive care medicine 2006; 32: 1030-1038
[29] Gurbuz HA, Durukan AB, Salman N, Tavlasoglu M, Durukan E, Ucar HI, Yorgancioglu C. Hydroxyethyl starch 6%, 130/0.4 vs. a balanced crystalloid solution in cardiopulmonary bypass priming: a randomized, prospective study. J Cardiothorac Surg 2013; 8:71
[30] Lee JS, Ahn SW, Song JW, Shim JK, Yoo KJ, Kwak YL. Effect of hydroxyethyl starch 130/0.4 on blood loss and coagulation in patients with recent exposure to dual antiplatelet therapy undergoing off-pump coronary artery bypass graft surgery. Circ J 2011; 75: 2397-2402

[31] James MF, Michell WL, Joubert IA, et al. Resuscitation with hydroxyethyl starch improves renal function and lactate clearance in penetrating trauma in a randomized controlled study: the FIRST trial (Fluids in Resuscitation of Severe Trauma). Br J Anaesth 2011; 107(5):693-702
[32] Neff TA, Doelberg M, Jungheinrich C, et al. Repetitive large-dose infusion of the novel hydroxyethyl starch 130/0.4 in patients with severe head injury. AnesthAnalg 2003; 96(5):1453-9
[33] Myburgh J, Finfer S, Bellomo R et al for the CHEST Investigators and the Australian and New Zealand Intensive Care Society Clinical Trials Group. Hydroxyethyl Starch or Saline for Fluid Resuscitation in Intensive Care. Published on 17 October 2012 at NEJM.org
[34] Perel P, Roberts I. Colloids versus crystalloids for fluid resuscitation in critically ill patients. Cochrane Database Syst Rev. 2011 Mar 16;(3):CD000567
[35] Ogilvie MP, Pereira BM, McKenney MG, McMahon PJ, Manning RJ, Namias N, Livingstone AS, Schulman CI, Proctor KG. First report on safety and efficacy of hetastarch solution for initial fluid resuscitation at a level 1 trauma center. Journal of the American College of Surgeons 2010; 210: 870-80, 880

conclusion on the benefits in this setting, indeed the authors conclude that a randomised blinded trial is necessary before their results can be accepted with confidence.

- Guidry *et al.* (2013)[36]

Guidry *et al.* report a retrospective analysis in trauma patients receiving high ratios of fresh frozen plasma/packed red blood cells in damage control resuscitation (DCR). In total, 56 patients were included, 28 each in the crystalloid and colloid groups (Hextend = 6% hetastarch in an electrolyte solution). Ten-day mortality in the colloid group (7.1 %) was significantly lower in comparison to the crystalloid group (39.9 %, p=0.004). In addition, significantly greater volumes of crystalloid were infused.

The results of the study implied that there may be a lower risk of death in patients receiving HES compared with those receiving crystalloid. The authors concluded "A multi-institutional analysis is needed in order to validate these results."

Volume sparing effect of colloids

The MAHs claimed that the volume efficacy of iso-oncotic colloids (including HES) is higher compared to crystalloid solutions, and less colloid volume is needed to stabilise the patient haemodynamically (e.g. Jacob *et al.* 2013[37]; Feldheiser *et al.* 2013[38]). Furthermore, some studies showed that colloids exert lower extravascular extravasation, and improved tissue perfusion, especially in initial phase of resuscitation (e.g. Rackow *et al.* 1983[39], Trof *et al*. 2010[40]). They also claimed that in the hypovolaemic patient with normal pulmonary function, the use of colloids to maintain colloid-osmotic pressure may limit the development of peripheral as well as pulmonary oedema (Vincent JL 2000[41]). It is suggested that colloids might help preventing positive fluid balance and/or over-infusion of fluids (Wills 1995, Naing CM and Win DK 2010[42]). The MAHs also considered that a positive net fluid balance is associated with a decrease in organ perfusion and an increased mortality (e.g. Sadaka F *et al*. 2013[43], Payen D *et al*. 2008[44]). Meybohm P *et al*. 2013[45] suggest that use of HES should be restricted to the initial phase of volume resuscitation with a maximum time interval of 24 h.

Conclusion on efficacy

The PRAC was of the opinion that short-term haemodynamic improvements have been observed in other patient populations, including surgical and trauma patients. Whilst recognising the limitations of these studies which included limited size and short-term follow-up in many studies, the PRAC noted that some volume sparing effect was reported in Madi-Jebara *et al.* 2004, that suggested that HES

[36] Guidry C, Gleeson E, Simms ER, Stuke L, Meade P, McSwain NE Jr, Duchesne JC. Initial assessment on the impact of crystalloids versus colloids during damage control resuscitation. J Surg Res. 2013 Jun 15. pii: S0022-4804(13)00568-4
[37] Jacob M. Chapter 10. The pharmacology of colloid solutions.pp.72-80. In: Jacob M, Nohé B. Rational Fluid and Volume Therapy in Anaesthesia and Intensive Care Medicine. 2013 UNI-MED Verlag AG, Bremen, International Medicinal Publishers (London, Boston)
[38] Feldheiser A, Pavlova V, Bonomo T, et al. Balanced crystalloid compared with balanced colloid solution using goal-directed haemodynamic algorithm. Br J Anaesth 2013; 110(2):231-40
[39] Rackow EC, Falk JL, Fein A, et al. Fluid resuscitation in circulatory shock: a comparison of the cardiorespiratory effccts of albumin, hetastarch, and saline solutions in patients with hypovolemic and septic shock. Crit Care Med 1983; 11(11):839-50
[40] Trof JR, Sukul SP, Twisk JWR et al. Greater cardiac response of colloid than saline fluid loading in septic and non-septic critically ill patients with clinical hypovolaemia. Intensive Care Med 2010; 36(4):697-701
[41] Vincent JL. Issues in contemporary fluid management. Crit Care 2000; 4(Suppl 2):S1-2
[42] Naing CM, Win DK. Do colloids in comparison to crystalloids for fluid resuscitation improve mortality? Transactions of the Royal Society of Tropical Medicine and Hygiene 2010; 104(5):311-2
[43] Sadaka F, Juarez M, Naydenov S, et al. Fluid Resuscitation in Septic Shock: The Effect of Increasing Fluid Balance on Mortality. J Intensive Care Med. 2013 Feb 27. [Epub ahead of print]
[44] Payen D, De Pont AC, Sakr Y, et al. A positive fluid balance is associated with a worse outcome in patients with acute renal failure. Crit Care 2008; 12(3):R74
[45] Meybohm P, Van Aken H, De Gasperi A, et al. Re-evaluating currently available data and suggestions for planning randomised controlled studies regarding the use of hydroxyethyl-starch in critically ill patients - a multidisciplinary statement. Crit Care 2013; 17:R166[Epub ahead of print]

130/0.4 6% seems to have benefits over twice the volume of Ringer's lactate in preventing spinal anaesthesia induced hypotension. Some benefit for elective surgical patients has also been shown in short-term surrogate hemodynamic outcomes along with a modest volume sparing effect (Hartog *et al*. 2011[46]). In hypovolaemic patients with normal pulmonary function, the use of colloids to maintain colloid-osmotic pressure may limit the development of peripheral as well as pulmonary oedema (Vincent JL 2000). Some publications also suggest that colloids might help to prevent positive fluid balance and/or over-infusion of fluids (Wills 1995, Naing CM and Win DK 2010). Some of authors argue that a positive net fluid balance is associated with a decrease in organ perfusion and an increased mortality (e.g. Sadaka F *et al*. 2013, Payen D *et al*. 2008). Meybohm P *et al*. 2013 suggest that use of HES should be restricted to the initial phase of volume resuscitation with a maximum time interval of 24 h. Martin *et al* 2002 showed that HES treatment resulted in a significantly lower estimated blood loss and that there was no difference in red blood cells, or blood product utilisation among the groups. Hamaji et al 2013 also showed significantly fewer red blood cell transfusions were required in the HES group.

Given the lack of robust evidence to demonstrate that the short-term hemodynamic benefit shown translates into efficacy in terms of long-term patient relevant outcomes, PRAC agreed that further studies are needed to evaluate the efficacy of HES in elective surgery and trauma patients.

2.2. Risk minimisation activities, including communication

The PRAC recommended the following activities to minimise the risks.

Amendments to the product information

Based on the above assessment, the PRAC recommended amendments to the product information for hydroxyethyl starch containing products for solutions for infusion.

The amendments aim to reflect a restricted indication.

The amendments also aim to minimise the risk of mortality and renal failure associated with HES and require close monitoring of the renal function during treatment to be performed and that use of HES is contraindicated in patients with sepsis, critically patients and burns patients.

Furthermore, the PRAC decided that hydroxyethyl starch-containing products should be included in the additional monitoring list. Therefore, further amendments have been included in the product information.

More details on the proposed changes to the product information can be found in the relevant section.

Information and awareness of the Healthcare professionals and Patients

Educational measures are necessary in order to clearly inform prescribers and patients on the risk of mortality and renal failure associated with HES and on the measures necessary to minimise the risk.

Direct healthcare professional communication (DHPC)

Core elements of a direct healthcare professional communication (DHPC) were agreed during the assessment of these medicinal products to inform the healthcare professionals on the outcome of the procedure and the changes to the use of HES-containing medicinal products.

[46] Hartog CS, et al. A systematic review of third-generation hydroxyethyl starch (HES 130/0.4) in resuscitation: Safety not adequately addressed. Anesth Analg. 2011;112:635-45

Future Monitoring

i. Randomised clinical trials (RCT)

As a condition to the marketing authorisations, the PRAC requested the MAHs to perform large randomised clinical trials in order to demonstrate the efficacy and safety of hydroxyethyl starch containing products in the perioperative and trauma populations. The following meaningful endpoints should be considered:

Composite primary endpoints

90-day mortality and 90-day renal failure

Secondary endpoints

- major peri-operative complications (e.g. infections, bleedings, anastomosis insufficiency, reoperation rate, diagnosis of pulmonary oedema).

- haemodynamic stabilisation in relation to dose (e.g. Heart rate, mean arterial pressure, central venous pressure, central venous oxygen saturation, serum lactate level, base excess and urine output)

- length of stay, morbidity, coagulation, inflammation, hospital mortality

- measurement of creatinine (GFR)

The synopsis of the studies should be submitted to the NCAs within 2-month of the CMDh agreement/ European Commission (EC) decision. The final protocol of the studies should be submitted to the NCAs within 6-month of the CMDh agreement/ EC decision. The results studies should be made available by end of 2016.

ii. Drug utilisation study (DUS)

As a condition to the marketing authorisations, the MAHs should conduct a drug utilisation study (DUS) (or survey) in several member states to evaluate the effectiveness of the risk minimisation measures taken. This drug utilisation study should aim to characterise prescribing practices during typical clinical use in representative groups of prescribers.

iii. Risk management plan (RMP)

The MAHs are encouraged to submit RMPs to NCAs.

2.3. Product information

Summary of product characteristics

The PRAC concluded that hydroxyethyl should be subject to additional monitoring. Therefore the following sentence is to be added into the SmPC : "▼*This medicinal product is subject to additional monitoring. This will allow quick identification of new safety information. Healthcare professionals are asked to report any suspected adverse reactions. See section 4.8 for how to report adverse reactions."*

Section 4.1 Therapeutic indication

The wording of this section should be read as below:

Treatment of hypovolaemia due to acute blood loss when crystalloids alone are not considered sufficient. (see sections 4.2, 4.3 and 4.4)

Section 4.2 Posology and method of administration

The PRAC considered that use of HES should be restricted to the initial phase of volume resuscitation with a maximum time interval of 24.
The PRAC also recommended that the first 10-20 ml should be infused slowly and under careful monitoring of the patient so that any anaphylactoid reaction can be detected as early as possible.
The section should reflect the maximum daily dose is 30ml/kg for 6% HES (130/0.40) and 6% HES (130/0.42). The maximum daily dose may be different between HES products and therefore this dose should be recalculated accordingly for other HES products.
The PRAC considered that the lowest possible effective dose should be applied. The PRAC also recommended that the treatment should be guided by continuous haemodynamic monitoring so that the infusion is stopped as soon as appropriate haemodynamic goals have been achieved. The maximum recommended daily dose must not be exceeded.

The PRAC considered that since data are limited in children, it is recommended not to use HES products in this population.

Section 4.3 Contraindications

The PRAC considered that the following contraindications should be added to this section:

- hypersensitivity to the active substances or to any of the other excipients listed in section 6.1
- sepsis
- burns
- renal impairment or renal replacement therapy
- intracranial or cerebral haemorrhage
- critically ill patients (typically admitted to the intensive care unit)
- hyperhydration
- pulmonary oedema
- dehydration
- hyperkalaemia *[only applicable to products containing potassium]*
- severe hypernatraemia or severe hyperchloraemia
- severely impaired hepatic function
- congestive heart failure
- severe coagulopathy
- organ transplant patients

Section 4.4 Special warnings and precautions for use

The PRAC recommended for warnings to be included in this section.
The section should reflect the risk of allergic (anaphylactoid) reactions, the patient should be monitored closely and the infusion instituted at a low rate.
In surgery and trauma, the PRAC considered that there is a lack of robust long term safety data in patients undergoing surgical procedures and in patients with trauma. The expected benefit of treatment should be carefully weighed against uncertainty with regard to this long term safety. Other available treatment options should be considered.

The PRAC indicated that volume replacement with HES has to be considered carefully, and haemodynamic monitoring is required for volume and dose control

The PRAC recommended that volume overload due to overdose or too rapid infusion must always be avoided. The dosage must be adjusted carefully, particularly in patients with pulmonary and cardiocirculatory problems. Serum electrolytes, fluid balance and renal function should be monitored closely.

This section was also amended to reflect that HES products are contraindicated in patients with renal impairment or renal replacement therapy and that the use of HES must be discontinued at the first sign of renal injury.
It is also reflected that an increased need for renal replacement therapy has been reported up to 90 days after HES administration and that monitoring of renal function in patients is recommended for at least 90 days.

The PRAC recommended that particular caution should be exercised when treating patients with impaired hepatic function or in patients with blood coagulation disorders.
Severe haemodilution resulting from high doses of HES solutions must also be avoided in the treatment of hypovolaemic patients.
In the case of repeated administration, blood coagulation parameters should be monitored carefully.
The section also reflects that use of HES should be discontinued at the first sign of coagulopathy.
The PRAC recommended that in patients undergoing open heart surgery in association with cardiopulmonary bypass the use of HES products is not recommended due to the risk of excess bleeding.

This section also reflects that data are limited in children therefore it is recommended not to use HES products in this population.

Section 4.8 Undesirable effects

This section was amended to include the following adverse events: hepatic injury and renal injury. It is to be noted that the frequency of these adverse events is not known (cannot be estimated from the available data).

Package Leaflet

The package leaflet was aligned to the SmPC proposals.

3. Overall discussion and benefit-risk assessment

Hydroxyethyl starch (HES) solutions for infusion include products with starch derived from potato or corn with different molecular weights and substitution ratios. HES containing solutions for infusion were indicated mainly for the treatment and prophylaxis of hypovolaemia and hypovolemic shock.

HES solutions have been the object of two reviews. The first review was initially started under the framework of Article 31 of Directive 2001/83/EC. The PRAC issued a recommendation on available data for this review in June 2013, concluding that HES solutions should be suspended in all patient populations. Following requests for re-examination by marketing authorisation holders (MAHs), the PRAC confirmed its previous position under the Article 31 in October 2013. While the re-examination was ongoing some Member States decided to suspend or limit the marketing or use of these medicines in their territories. In accordance with the EU legislation, this type of action required that an EU review procedure be carried out. Consequently, a second review of HES solutions under Article 107i of Directive 2001/83/EC was initiated, and it ran separately but in parallel with the re-examination of the Article 31, also finalising in October 2013. However, it must be noted that new evidence was considered in the procedure under Article 107i of Directive 2001/83/EC. This new evidence was not available when the PRAC recommendation on the procedure under Article 31 of Directive 2001/83/EC was issued in June 2013 and could therefore not be considered in the re-examination of the latter in October 2013. It is on the basis of the totality of the data available, including the new evidence, that the PRAC issued conclusion on the procedure provided for in Article 107i of Directive 2001/83/EC in October 2013. Therefore the conclusions on the Article 107i of Directive 2001/83/EC reflect the most complete and up-to-date evaluation of the available data relating to the HES containing medicinal products.

Details of this recommendation are presented hereafter.

Under the framework of the Article 107i of Directive 2001/83/EC, the PRAC considered recommendations on HES rendered in the referral under Article 31 of Directive 2001/83/EC and also reviewed available data including clinical studies, meta-analyses of clinical studies, post-marketing experience, responses submitted by the marketing authorisation holders (MAHs) in writing and at oral explanations, spontaneous reports on the safety and efficacy of hydroxyethyl starch containing products for solutions for infusion, as well as stakeholders' submissions in particular with regards to the risk of mortality and renal failure.

On the basis of the available data, in particular results from VISEP, 6S and CHEST studies, the PRAC concluded that HES is associated with an increased risk of mortality and renal failure in patients with sepsis, in critically ill and burn patients and that the benefits of HES do not outweigh the risks in these patient populations.

However, it was noted that short-term haemodynamic improvements have been observed in other patient populations, including surgical and trauma patients. Whilst recognising the limitations of these studies which included limited size and short duration of follow-up, the PRAC noted that some volume sparing effect was reported in Madi-Jebara *et al.* 2004, that suggested that HES 130/0.4 6% seems to have benefits over twice the volume of Ringer's lactate in preventing spinal anaesthesia induced hypotension. Some benefit for elective surgical patients has also been shown in short-term surrogate hemodynamic outcomes along with a modest volume sparing effect (Hartog *et al*. 2011). In hypovolaemic patients with normal pulmonary function, the use of colloids to maintain colloid-osmotic pressure may limit the development of peripheral as well as pulmonary oedema (Vincent JL 2000). Some publications also suggest that colloids might help to prevent positive fluid balance and/or over-infusion of fluids (Wills 1995, Naing CM and Win DK 2010). Some of authors argue that a positive net

fluid balance is associated with a decrease in organ perfusion and an increased mortality (e.g. Sadaka F *et al*. 2013, Payen D *et al*. 2008). Meybohm P *et al*. 2013 suggest that use of HES should be restricted to the initial phase of volume resuscitation with a maximum time interval of 24 h. Martin *et al* 2002 showed that HES treatment resulted in a significantly lower estimated blood loss and that there was no difference in red blood cells, or blood product utilisation among the groups. Hamaji et al 2013 also showed that significantly fewer red blood cell transfusions were required in the HES group.

Therefore, the PRAC noted the available data from studies in surgical and trauma patients and considered that although these studies were limited in size and duration of follow-up they did provide some reassurance that the risks of mortality and renal injury in surgical and trauma patients may be lower than those in the critically ill and patients. Although the mechanisms by which increased renal injury and mortality occur is not well established, it is possible that the degree of inflammatory processes seen in sepsis and critically ill patients is greater and associated with significant capillary leakage compared with other patient populations such as the perioperative setting after elective surgery or un-complicated trauma where the systematic inflammatory process and the extent of capillary leak may be lower.

New results from CRYSTAL have also become available. Despite the studies' limitations which were noted, the results from the CRYSTAL study comparing colloids to crystalloids showed that in patients with hypovolemia, the use of colloids vs crystalloids did not result in a significant difference in 28-day mortality. Although 90-day mortality was lower among patients receiving colloids, this requires further investigations. In addition, in the BaSES study, the hospitalisation time was significantly reduced in patients treated with 6% HES 130/0.4 compared to 0.9% NaCl. Results from the RaFTinG registry in intensive care units, an observational, non-randomised study aiming to gather more information in 'real-life' clinical practice showed no statistically significant differences between patients treated with crystalloids only (n=2482) and those treated with colloids (all HES preparations and gelatin, n=2063) for the endpoints of 90-day mortality. The PRAC therefore acknowledged the results of this studies which shows no risk of mortality associated with the use of HES but considered that given the limitations of this study its findings could not negate the findings from 6S and VISEP studies that had shown an increased risk of mortality in critically ill patients.

Additional expert advice was sought from an ad-hoc expert group. The experts agreed that the benefits may be observed in severe hypovolaemia in short duration only at the beginning i.e. perioperative setting and disappearing faster with patient's stabilisation. The experts suggested that benefit of HES may be seen in particular in perioperative bleeding.

Therefore, the PRAC agreed that the therapeutic indication of HES containing products should be restricted to treatment of hypovolaemia due to acute blood loss when crystalloids alone are not considered sufficient. However additional measures must be implemented to minimise potential risks in these patients. HES solutions should be restricted to the initial phase of volume resuscitation with a maximum time interval of 24 h. The posology section should identify the maximum daily dose and should recommend that the lowest possible effective dose should be employed. HES products are contraindicated in patients with renal impairment or renal replacement therapy but the contraindications should also be extended to include other patient populations including patients with sepsis, critically ill patients and burns patients. The PRAC considered that the use of HES must be discontinued at the first sign of renal injury. Monitoring of renal function in patients is recommended for at least 90 days. Particular caution should be exercised when treating patients with impaired hepatic function or in patients with blood coagulation disorders. The product information will be updated to reflect these restrictions and warnings.

In addition, two phase IV randomised clinical trials with an appropriate control and clinically meaningful endpoints will need to be conducted to provide more evidence on the efficacy and safety,

including the risk of 90-day mortality and renal failure, in perioperative and trauma populations. An European drug utilisation study will also be conducted to evaluate the effectiveness of the recommended risk minimisation measures. Protocols and results of these studies will be submitted to national competent authorities according to agreed timelines. The MAHs are also encouraged to submit risk management plans to national competent authorities.

Benefit risk balance

In view of the totality of the evidence available in the procedure under Article 107i of Directive 2001/83/EC, the PRAC considered that Hydroxyethyl starch should be restricted to the treatment of hypovolaemia due to acute blood loss when crystalloids alone are not considered sufficient subject to agreed restrictions, contraindications, warnings, other changes to the product information and additional risk minimisation measures.

The PRAC conclusion in the context of the referral procedure under Article 107i of Directive 2001/83/EC included additional data that was not available when the PRAC issued its recommendation on the referral in accordance with Article 31 of Directive 2001/83/EC in June 2013 and therefore could not be considered in the re-examination of the latter in October 2013. Therefore the conclusions on the Article 107i of Directive 2001/83/EC reflect the most complete and up-to-date evaluation of the available data relating to the HES containing medicinal products.

4. Action plan and communication plan

The PRAC considered that a Direct Healthcare Professional Communication (DHPC) was needed to communicate on the measures taken for the safe use of these medicinal products. The core elements of this DHPC were agreed by the PRAC, together with the communication plan (see attachments to this report).

The MAHs should agree the translations and local specificities of the DHPC with national competent authorities. The DHPC should be sent to specialists depending on country (anaesthesiologists, specialists in intensive care medicine, specialists in infectious diseases, specialists in renal diseases, specialists in burn care, specialists in trauma care).

5. Conclusion and grounds for the recommendation

Whereas,

- The Pharmacovigilance Risk Assessment Committee (PRAC) considered the procedure under Article 107i of Directive 2001/83/EC, for hydroxyethyl starch containing products for solutions for infusion.
- The PRAC noted the conclusions of a review under Article 31 of Directive 2001/83/EC. However, for the current procedure under Article 107i of Directive 2001/83/EC the PRAC reviewed new available data, with a focus on risk of mortality and renal failure, including clinical studies, meta-analyses of clinical studies, post-marketing experience, responses submitted by the marketing authorisation holders (MAHs) in writing and at oral explanations and stakeholders' submissions.
- The PRAC considered that the use of hydroxyethyl starch is associated with an increased risk of mortality and renal replacement therapy or renal impairment in patients with sepsis, critically ill and burn patients.

- The PRAC considered, in view of the new evidence which includes data from clinical trials, further expert advice, new proposals for additional risk minimisation measures, including restrictions on use and a commitment from the MAHs to perform additional studies in patients with trauma and in elective surgery, that the benefit of hydroxyethyl starch containing products outweighs the risk in the treatment of hypovolaemia due to acute blood loss when crystalloids alone are not considered sufficient. This is subject to restrictions, warnings and other changes to the product information.
- The PRAC concluded that hydroxyethyl starch containing products should be contraindicated in patients with sepsis, in critically ill and burn patients. In addition, special warnings in surgery and trauma patients have been included.
- The PRAC also concluded that there was need for further risk minimisation measures such as information to patients and healthcare professionals. Core elements of a direct healthcare professional communication were agreed, together with the timelines for distribution, and that studies should be conducted. The PRAC also considered that studies should be conducted to provide more evidence on the efficacy and safety of hydroxyethyl starch in the perioperative setting and trauma.

The PRAC concluded that the benefit-risk balance for hydroxyethyl starch containing medicinal products remains favourable in treatment of hypovolaemia due to acute blood loss when crystalloids alone are not considered sufficient subject to the agreed restrictions, contraindications, warnings, other changes to the product information and additional risk minimisation measures.

The PRAC conclusion in the context of the referral procedure under Article 107i of Directive 2001/83/EC included additional data that was not available when the PRAC issued its recommendation on the referral in accordance with Article 31 of Directive 2001/83/EC in June 2013 and therefore could not be considered in the re-examination of the latter in October 2013. Therefore the conclusions on the Article 107i reflect the most complete and up-to-date evaluation of the available data relating to HES containing medicinal products.

Appendix 1

Listing of submissions of all data received by the Agency

Listing of submissions of data received by the Agency (i.e. from MAHs and other stakeholders) for hydroxyethyl starch containing medicinal products

Submission
**MAHs**
Serumwerk Bernburg AG
B.Braun Melsungen AG
Fresenius Kabi
Baxter
**Stakeholders**
Healthcare professional – Anaesthesiology, Greece
Healthcare professional - Anaesthesiology and Intensive Care Finland
Healthcare professional – Anaesthesia, India
Healthcare professional - Intensive care, India
Research – Department of Outcomes Research, Anaesthesiology Institute, USA
Healthcare professional - Intensive Care, Denmark
Healthcare professional –Department Anaesthesiology and Critical Care and Department of Anaesthesia, South Africa
Healthcare professional - Department of Anaesthesia, Philippines
Healthcare professional - Critical Care, India
Healthcare professional -Cardiac Anaesthesiology, India
Healthcare professional - Critical Care, India
Healthcare professional – Internist-intensivist, Greece
Healthcare professional -French Society of Anaesthesia and Intensive Care Medicine (SFAR)
Healthcare professional –Anaesthetist, India
Patient - Mexico
Healthcare professional - Spain
Healthcare professional - Anaesthesiology, Malaysia
Healthcare professional –Specialist anaesthetist and researcher, South Africa
Healthcare professional - Internist and Intensivist; Clinical Pharmacologist and Metaanalyst, Italy
Healthcare professional- Internist and Intensivist; Clinical Pharmacologist and Metaanalyst, Italy

Submission
Healthcare professional – Internist and Intensivist; Clinical Pharmacologist and Metaanalyst, Italy
Healthcare professional - Department of Anaesthesiology and Intensive Care, Center for Sepsis Control and Care, Germany
Healthcare professional - Transfusions, Spain
Healthcare professional - Anaesthesiologists, Germany
Healthcare professional –Critical Care Medicine, India
Industry – Germany
Healthcare professional – Intensivist, Greece
Healthcare professional –Emergency Dept., India
Healthcare professional –Department of Anaesthesiology, South Africa
Healthcare professional –Anaesthesiologist, Mexico
Healthcare professional –Mexico
Healthcare professional – Intensive Care specialist , Internal medicine/nephrology, Belgium
Healthcare professional –Intensive Care specialist, Belgium
Healthcare professional - Sepsis and multiple organ failure, Anaesthesiology, Germany
Healthcare professional - Medical specialist in Cardiovascular Anaesthesia, Mexico
Healthcare professional - Anaesthesiology and ICU, Argentina
Healthcare professional – Anesthesiologist, USA
Healthcare professional - German Society of Anaesthesiology and Intensive Care Medicine (DGAI), Germany
Healthcare professional - G.I.C.R. (gruppo interdisciplinare chimica dei radiofarmaci), Italy
Healthcare professional - Anaesthesiologist, France
Healthcare professional –Anaesthesia and Surgical ICU Department, Egypt
Healthcare professional - Anaesthesiology and intensive care medicine, Germany
Healthcare professional –Anaesthesia, India
Healthcare professional - Emergency medicine and critical care, México
Healthcare professional – Anaesthesiology, Japan
Healthcare professional –Anaesthesiologist and Intensivist, The Netherlands
Healthcare professional - Intensive Care, Belgium
Healthcare professional –Anaesthesiologist and Critical Care, Critical Care and Pain, India
Academia/student - Critical Care & Trauma Division, Australia
Healthcare professional - Anaesthesiology, Argentina

**Submission**
Healthcare professional - . Anaesthesiology and Intensive Care Medicine, Czech Republic
Research institute - Department of Surgery, Greece
Healthcare professional – Perfusionist, USA
Healthcare professional - Austrian Society of Anaesthesiology, Resuscitation and Intensive Care, Austria
Healthcare professional –Anaesthesia and Intensive Care in Cardiac Surgery, Italy
Healthcare professional –Anaesthesiologist, The Netherlands
Healthcare professional - Cardiothoracic and Vascular Anaesthesiologist, Mexico
Healthcare professional - Critical Care, Spain
Healthcare professional –Anaesthetist and researcher, Department of Anaesthesia , South Africa
Healthcare professional - Asian heart institute, India
Healthcare professional - Critical Care Medicine
Healthcare professional - Cardiac anaesthesiologist , USA
Healthcare professional - Anaesthesia and Intensive Care, France
Healthcare professional –Intensivist, India
Healthcare professional – Anaesthesia, India
Healthcare professional – India
Healthcare professional - Cardiac Anaesthesiologist and intensivist, India
Healthcare professional – Respiratory Medicine, Critical Care and Sleep Medicine, India
Healthcare professional –Intensivist,Critical Care & Emergency Medicine , India
Healthcare professional - Anaesthesiology and Perioperative Care, USA
Healthcare professional – Anaesthesia, India
Healthcare professional – Anaesthesiologists, Mexico
Healthcare professional - Anaesthesia and Intensive Care, Italy
Healthcare professional –Institute of Physiology and Pathophysiology, Germany
Healthcare professional - Education Research Foundation, India
Healthcare professional –Intensivist, India
Healthcare professional –Consultant Anaesthetist, Singapore
Healthcare professional –Anaesthesia, India
Healthcare professional –Consultant anaesthesiologist, India
Healthcare professional

Submission
Healthcare professional - Anaesthesiology, Spain
Healthcare professional - consultant anaesthetist, India
Healthcare professional - Paediatric Surgeon, India
Healthcare professional - Indian Society of Critical Care Medicine, India
Healthcare professional - Consultant in critical care, India
Healthcare professional - Anaesthesiologist , Korea Republic
Healthcare professional - Cardiac anaesthesiologist, India.
Healthcare professional - Anaesthesiology and Reanimation., Turkey
Healthcare professional –Anaesthesiologist, Belgium
Healthcare professional - Intensivist, India
Healthcare professional - Anaesthesiologist, India
Healthcare professional - Critical care, Mexico
Healthcare professional - Liver Transplant, Anaesthesia and Intensive Care, India
Healthcare professional -, National Cancer Institute, Anaesthesiology , Egypt,
Healthcare professional –Anaesthesia and Critical Care, India
Healthcare professional - India
Healthcare professional - Department of Anaesthesiology and Intensive Therapy, Hungary
Research Institute - Queen Marys University of London

Appendix 2

Divergent positions to PRAC recommendation

Article 107i of Directive 2001/83/EC

Procedure No: EMEA/H/A-107i/1376

Solutions for infusion containing hydroxyethyl starch

Divergent statement

The following members of PRAC did not agree with the PRAC's Recommendation on the Article 107i referral resulting from pharmacovigilance data for solutions for infusion containing hydroxyethyl starch (HES) based on the following reasons:

1. Harm from use of HES compared with crystalloids in terms of increased mortality and increased renal injury, as well as other serious adverse reactions, has been shown in large well-designed randomised controlled trials in septic and critically ill patients. The available studies in elective surgery and trauma cannot provide reassurance of a lower risk than in septic and critically ill patients, or indeed exclude such a risk. The ad hoc Expert Advice Group held in September 2013 agreed that the evidence to demonstrate a lower risk of renal injury and mortality in any settings other than sepsis and the critically ill is weak.

2. There is very limited evidence on the benefits and risks of hydroxethyl starch solutions for use in elective surgery and trauma. The magnitude of the volume sparing effect of HES relative to crystalloid solutions has often been cited as 3-4 fold, however there is some evidence that this ratio is lower in surgical settings, around 1.8 fold in some types of surgery (Hartog 2011). It is unclear how the surrogate endpoints from these studies translate to clinically relevant endpoints. Both ad hoc Expert Advice Groups (meetings 19 April 2013 and 13 September) 2013) commented that the data evaluating benefit in the perioperative setting and trauma setting do not adequately support a clinically relevant beneficial effect for HES.

3. There is an overlap between those patient groups where the benefit-risk balance of HES is judged to be negative (septic and critically ill patients), and the patients who will receive HES under the revised indication allowing use in patients with hypovolaemia due to acute bleeding (e.g. including the trauma and perioperative settings). In traumatic injury the patients most likely to receive HES are also those likely to have the most severe injury, and therefore have a greater degree of systemic inflammatory processes and increased risk from exposure to HES. It should also be noted that elective surgery and trauma patients can develop sepsis or complications requiring critical care and these patients cannot be identified in advance. Approximately 20% of the critically ill patients in the CHEST study entered the ICU following elective surgery (Myburgh et al, 2012).

4. The mechanism underlying the risks of renal injury and mortality observed with HES is not well understood, and therefore these should be considered to be potential risks in all patient groups. Systemic inflammatory processes may contribute to the observed increased risk in sepsis and burn injury. There is a continuum in the extent of systemic inflammation between healthy individuals and patients with sepsis or burn injury. Trauma and surgery patients are located on an intermediate position on this continuum. There is also evidence that tissue deposition of hydroxyethyl starch occurs in healthy patients without inflammatory processes (Sirtl et al, 1999).

5. Alternative treatments are available in the form of crystalloids, and high quality care is possible without the use of HES: a survey of 391 ICUs worldwide conducted in 2010 (Finfer et al, 2010) showed no use of HES in the United States or Australia.

6. Without evidence to provide reassurance that patients will not be exposed to increased risk of mortality and renal injury by use of HES, and given the lack of data supporting a clinically relevant benefit, suspension of marketing authorisations for HES products in all patient populations remains appropriate to protect public health. This would avoid the situation where patients are unnecessarily exposed to risk from treatment with HES with no convincing evidence that they are receiving any additional benefit.

7. The ability of the proposed risk minimisation measures to sufficiently minimise the risks of HES is a concern. Data are lacking to identify an appropriate maximum dose, and expert advice is that there is no absolute 'safe' lower dose below which there is no risk associated with HES

administration. The recommendation to monitor renal function in patients for at least 90 days may not be an effective measure to minimize the risk of renal injury in all patients as detection of worsening of renal function by monitoring may not be practical in patients who are discharged shortly after receiving HES. Furthermore, in emergency settings, it may be particularly difficult to evaluate patients for contraindications.

Due to the above mentioned arguments the below mentioned PRAC delegate considers the benefit-risk balance of hydroxyethyl starch (HES) to be negative in all patient populations, justifying the suspension of the marketing authorisations of all HES-containing medicinal products.

PRAC members expressing a divergent position:

Kamila Czajkowska (PL)	10 October 2013	Signature: …………………………....
Marie Louise De Bruin	10 October 2013	Signature: …………………………....
Jacqueline Genoux-Hames (LU)	10 October 2013	Signature: …………………………....
Martin Huber (DE)	10 October 2013	Signature: …………………………....
Brigitte Keller-Stanislawski	10 October 2013	Signature: …………………………....
Maria Popova-Kiradjieva (BG)	10 October 2013	Signature: …………………………....
Carmela Macchiarulo (IT)	10 October 2013	Signature: …………………………....
Almath Spooner (IE)	10 October 2013	Signature: …………………………....
Doris Stenver (DK)	10 October 2013	Signature: …………………………....
Amy Tanti (MT)	10 October 2013	Signature: …………………………....
Kirsti Villikka (FI)	10 October 2013	Signature: …………………………....
Julie Williams (UK)	10 October 2013	Signature: …………………………....

Stephen Evans	10 October 2013	Signature: ……………………………...

Article 107i of Directive 2001/83/EC resulting from pharmacovigilance data

Procedure No: EMA/H/A-107i/1376

Solutions for infusion containing hydroxyethyl starch (HES)

Divergent statement

The following member of PRAC did not agree with the PRAC's Recommendation on the Article 107i referral resulting from pharmacovigilance data for solutions for infusion containing hydroxyethyl starch based on the following reasons:

1. Treatment of hypovolemia is symptomatic, aiming to resolve the immediate threat to life and vital organ function.

   Harm from use of HES compared with crystalloids in terms of increased mortality and increased renal injury, as well as other serious adverse reactions, has been shown in large well-designed randomised controlled trials in septic and critically ill patients. The available studies in trauma cannot provide reassurance of a lower risk than in septic and critically ill patients, or indeed exclude such a risk. The ad hoc Expert Advice Group held in September 2013 agreed that the evidence to demonstrate a lower risk of renal injury and mortality in any settings other than sepsis and the critically ill is weak. Overall the group agreed the benefits may exist in severe hypovolaemia in short duration only at the beginning i.e. peri-operative setting and disappearing faster with patient's stabilisation.

   The benefit-risk may therefore be considered favourable only in this specific population (elective surgery) based on available data. In this indication HES can be acceptable for short term use.

2. Additional measures should be proposed to further minimize the identified and potential risks.

Due to the above mentioned arguments the below mentioned PRAC delegates consider the benefit/risk balance of Hydroxyethyl starch (HES) to be negative in populations other than elective surgery, justifying the suspension of the marketing authorisations of all HES-containing medicinal products for these indications.

PRAC members expressing a divergent position:

Sabine Straus (NL)	10-October 2013	Signature: ……………………………...

Article 107i of Directive 2001/83/EC

Procedure No: EMEA/H/A-107i/1376

Solutions for infusion containing hydroxyethyl starch

Divergent statement

The following members of PRAC did not agree with the PRAC's Recommendation on the Article 107i referral resulting from pharmacovigilance data for solutions for infusion containing hydroxyethyl starch (HES) based on the following reasons:

1. Harm from use of HES compared with crystalloids in terms of increased mortality and increased renal injury, as well as other serious adverse reactions, has been shown in large well-designed randomised controlled trials in septic and critically ill patients. The available studies in elective surgery and trauma cannot provide reassurance of a lower risk than in septic and critically ill patients, or indeed exclude such a risk. The ad hoc Expert Advice Group held in September 2013 agreed that the evidence to demonstrate a lower risk of renal injury and mortality in any settings other than sepsis and the critically ill is weak.

2. There is very limited evidence on the benefits and risks of hydroxethyl starch solutions for use in elective surgery and trauma. The magnitude of the volume sparing effect of HES relative to crystalloid solutions has often been cited as 3-4 fold, however there is some evidence that this ratio is lower in surgical settings, around 1.8 fold in some types of surgery (Hartog 2011). It is unclear how the surrogate endpoints from these studies translate to clinically relevant endpoints. Both ad hoc Expert Advice Groups (meetings 19 April 2013 and 13 September) 2013) commented that the data evaluating benefit in the perioperative setting and trauma setting do not adequately support a clinically relevant beneficial effect for HES.

3. There is an overlap between those patient groups where the benefit-risk balance of HES is judged to be negative (septic and critically ill patients), and the patients who will receive HES under the revised indication allowing use in patients with hypovolaemia due to acute bleeding (e.g. including the trauma and perioperative settings). In traumatic injury the patients most likely to receive HES are also those likely to have the most severe injury, and therefore have a greater degree of systemic inflammatory processes and increased risk from exposure to HES. It should also be noted that elective surgery and trauma patients can develop sepsis or complications requiring critical care and these patients cannot be identified in advance. Approximately 20% of the critically ill patients in the CHEST study entered the ICU following elective surgery (Myburgh et al, 2012).

4. The mechanism underlying the risks of renal injury and mortality observed with HES is not well understood, and therefore these should be considered to be potential risks in all patient groups. Systemic inflammatory processes may contribute to the observed increased risk in sepsis and burn injury. There is a continuum in the extent of systemic inflammation between healthy individuals and patients with sepsis or burn injury. Trauma and surgery patients are located on an intermediate position on this continuum. There is also evidence that tissue deposition of hydroxyethyl starch occurs in healthy patients without inflammatory processes (Sirtl et al, 1999).

5. Alternative treatments are available in the form of crystalloids, and high quality care is possible without the use of HES: a survey of 391 ICUs worldwide conducted in 2010 (Finfer et al, 2010) showed no use of HES in the United States or Australia.

6. Without evidence to provide reassurance that patients will not be exposed to increased risk of mortality and renal injury by use of HES, and given the lack of data supporting a clinically relevant benefit, suspension of marketing authorisations for HES products in all patient populations remains appropriate to protect public health. This would avoid the situation where patients are unnecessarily exposed to risk from treatment with HES with no convincing evidence that they are receiving any additional benefit.

7. The ability of the proposed risk minimisation measures to sufficiently minimise the risks of HES is a concern. Data are lacking to identify an appropriate maximum dose, and expert advice is that there is no absolute 'safe' lower dose below which there is no risk associated with HES

administration. The recommendation to monitor renal function in patients for at least 90 days may not be an effective measure to minimize the risk of renal injury in all patients as detection of worsening of renal function by monitoring may not be practical in patients who are discharged shortly after receiving HES. Furthermore, in emergency settings, it may be particularly difficult to evaluate patients for contraindications.

Due to the above mentioned arguments the below mentioned PRAC delegate considers the benefit-risk balance of hydroxyethyl starch (HES) to be negative in all patient populations, justifying the suspension of the marketing authorisations of all HES-containing medicinal products.

PRAC member expressing a divergent position:

Ingebjørg Buajordet (NO)	10 October 2013	Signature: …………………………....

Annex III

Amendments to relevant sections of the summary of product characteristics and package leaflet

Note:

The relevant sections of the Summary of Product Characteristics and package leaflet are the outcome of the referral procedure.

The product information shall be subsequently updated by the Member State competent authorities, in liaison with the Reference Member State, as appropriate, in accordance with the procedures laid down in Chapter 4 of Title III of Directive 2001/83/EC.

I. Summary of Product Characteristics

<▼This medicinal product is subject to additional monitoring. This will allow quick identification of new safety information. Healthcare professionals are asked to report any suspected adverse reactions. See section 4.8 for how to report adverse reactions.>

[...]

Section 4.1 Therapeutic indications

[The wording of this section should be read as below]

Treatment of hypovolaemia due to acute blood loss when crystalloids alone are not considered sufficient. (see sections 4.2, 4.3 and 4.4)

Section 4.2 Posology and method of administration

[This section should be amended to reflect the following wording]

Use of HES should be restricted to the initial phase of volume resuscitation with a maximum time interval of 24 h.

The first 10-20 ml should be infused slowly and under careful monitoring of the patient so that any anaphylactoid reaction can be detected as early as possible.

The maximum daily dose is *<30ml/kg for 6% HES (130/0.40) and 6% HES (130/0.42); for other HES products the maximum daily dose should be recalculated accordingly>*.

The lowest possible effective dose should be applied. Treatment should be guided by continuous haemodynamic monitoring so that the infusion is stopped as soon as appropriate haemodynamic goals have been achieved. The maximum recommended daily dose must not be exceeded.

Paediatric population:
Data are limited in children therefore it is recommended not to use HES products in this population.

[...]

Section 4.3 Contraindications

[This section should be amended to include the following contraindications]

- hypersensitivity to the active substances or to any of the other excipients listed in section 6.1
- sepsis
- burns
- renal impairment or renal replacement therapy
- intracranial or cerebral haemorrhage
- critically ill patients (typically admitted to the intensive care unit)
- hyperhydration
- pulmonary oedema
- dehydration
- hyperkalaemia *[only applicable to products containing potassium]*
- severe hypernatraemia or severe hyperchloraemia
- severely impaired hepatic function
- congestive heart failure
- severe coagulopathy
- organ transplant patients

[...]

Section 4.4 Special warnings and precautions for use

[This section should be amended to reflect the following wording]

Because of the risk of allergic (anaphylactoid) reactions, the patient should be monitored closely and the infusion instituted at a low rate (see section 4.8).

Surgery and trauma:
There is a lack of robust long term safety data in patients undergoing surgical procedures and in patients with trauma. The expected benefit of treatment should be carefully weighed against uncertainty with regard to this long term safety. Other available treatment options should be considered.

The indication for volume replacement with HES has to be considered carefully, and haemodynamic monitoring is required for volume and dose control. (See also section 4.2.)

Volume overload due to overdose or too rapid infusion must always be avoided. The dosage must be adjusted carefully, particularly in patients with pulmonary and cardiocirculatory problems. Serum electrolytes, fluid balance and renal function should be monitored closely.

HES products are contraindicated in patients with renal impairment or renal replacement therapy (see section 4.3). The use of HES must be discontinued at the first sign of renal injury.
An increased need for renal replacement therapy has been reported up to 90 days after HES administration. Monitoring of renal function in patients is recommended for at least 90 days.

Particular caution should be exercised when treating patients with impaired hepatic function or in patients with blood coagulation disorders.
Severe haemodilution resulting from high doses of HES solutions must also be avoided in the treatment of hypovolaemic patients.
In the case of repeated administration, blood coagulation parameters should be monitored carefully. Discontinue the use of HES at the first sign of coagulopathy.
In patients undergoing open heart surgery in association with cardiopulmonary bypass the use of HES products is not recommended due to the risk of excess bleeding.

Paediatric population:
Data are limited in children therefore it is recommended not to use HES products in this population. (see section 4.2)

[...]

Section 4.8 Undesirable effects

[This following wording should be reflected in this section]

[...]

Hepatic injury < frequency not known (cannot be estimated from the available data)>
Renal injury < frequency not known (cannot be estimated from the available data)>

[...]

II. Package Leaflet

< ▼This medicine is subject to additional monitoring. This will allow quick identification of new safety information. You can help by reporting any side effects you may get. See the end of section 4 for how to report side effects. >

[...]

1. What *<Product name>* is and what it is used for

[This section should be amended to include the below wording]

<Product name> is a plasma volume substitute that is used to restore the blood volume when you have lost blood when other products called crystalloids are not considered sufficient alone.

[...]

2. What you need to know before you use *<Product name>*

Do not use *<Product name>* if you:

[This section should be amended to include the below wording]

- are allergic to any of the active substances or any of the other ingredients of this medicine
- suffer from serious generalised infection (sepsis)
- suffer from burn injury
- have kidney impairment or receive dialysis
- have severe liver disease
- suffer from bleeding in the brain (intracranial *or cerebral* bleeding)
- are critically ill (e.g. you need to stay in an intensive care unit)
- have too much fluid in your body and you have been told that you have a condition known as hyperhydration
- have fluid in the lungs (pulmonary oedema)
- are dehydrated
- have been told that you have a severe increase of potassium [Note: only for products which contain potassium], sodium or chloride in your blood
- have severely impaired liver function
- have severe heart failure
- have severe problems with your blood clotting
- have received an organ transplant

[...]

Warnings and precautions

[This section should be amended to include the below wording]

It is important to tell your doctor if you have:
- impairment of your liver function
- problems with your heart or circulation
- blood clotting (coagulation) disorders
- problems with your kidneys

Because of the *risk of allergic* (anaphylactic/anaphylactoid*) reactions*, you will be monitored closely to detect early signs of an allergic reaction when you receive this medicine.

Surgery and trauma:
Your doctor will consider carefully if this medicine is suitable for you.

Your doctor will adjust the dose of *<Product name>* carefully in order to prevent fluid overload. This will be done especially if you have problems with your lungs or with your heart or circulation.

The nursing staff will also take measures to observe your body's fluid balance, blood salt level, and kidney function. If necessary you may receive additional salts.

In addition it will be ensured that you receive enough fluids.

<Product name> is contraindicated if you have kidney impairment or of kidney injury requiring dialysis.
If impaired kidney function occurs during therapy:
If the doctor detects first signs of kidney impairment he/she will stop giving you this medicine. In addition your doctor may need to monitor your kidney function for up to 90 days.

If you are given *<Product name>* repeatedly your doctor will monitor the ability of your blood to clot, bleeding time and other functions. In case of an impairment of the ability of your blood to clot, your doctor will stop giving you this medicine.

If you are undergoing open heart surgery and you are on a heart-lung machine to assist in pumping your blood during the surgery, the administration of this solution is not recommended.

[...]

3. How to use <Product name>

[This section should be amended to include the below wording]

Dosage
Your doctor will decide on the correct dose for you to receive.
Your doctor will use the lowest possible effective dose and will not infuse *<Product name>* for more than 24 hours.
The maximum daily dose is *<30ml/kg for 6% HES (130/0.40) and 6% HES (130/0.42); for other HES products the maximum daily dose should be recalculated accordingly>*.

Use in children
There is only limited experience of the use of this medicine in children. Therefore it is not recommended to use this medicine in children.

[...]

4. Possible side effects

[This section should be amended to include the below wording]

[...]

Frequency not known (cannot be estimated from the available data)
- Kidney injury
- Liver injury

Reporting of side effects
If you get any side effects, talk to your <doctor> <or> <,> <pharmacist> <or nurse>. This includes any possible side effects not listed in this leaflet. You can also report side effects directly via the national reporting system listed in Appendix V*. By reporting side effects you can help provide more information on the safety of this medicine.

[...]

11 October 2013
EMA/606303/2013

PRAC confirms that hydroxyethyl-starch solutions (HES) should no longer be used in patients with sepsis or burn injuries or in critically ill patients

HES will be available in restricted patient populations

The EMA's Pharmacovigilance Risk Assessment Committee (PRAC) has completed its review of HES solutions following an assessment of new information and commitments from companies for additional studies and risk minimisation activities. The Committee confirmed that HES solutions must no longer be used to treat patients with sepsis (bacterial infection in the blood) or burn injuries or critically ill patients, because of an increased risk of kidney injury and mortality. HES solutions may, however, continue to be used in patients to treat hypovolaemia (low blood volume) caused by acute blood loss, provided that appropriate measures are taken to reduce potential risks and that additional studies are carried out.

The review of HES solutions was initially triggered by the German medicines agency, the Federal Institute for Drugs and Medical Devices (BfArM), following studies showing an increased risk of mortality in patients with sepsis and an increased risk of kidney injury requiring dialysis in critically ill patients following treatment with HES solutions.

The PRAC had initially concluded on 13 June 2013 that HES solutions should be suspended in all patient populations. Since then, the PRAC has analysed and considered new evidence that was not available at the time of the initial recommendation, including new studies. The Committee has also looked at new proposals for additional risk minimisation measures, including restrictions on use and a commitment from the companies to conduct additional studies.

The PRAC, on the basis of all data available to date, considered whether a group of patients could be identified for whom HES treatment remains beneficial. The Committee concluded that there was clear evidence for an increased risk of kidney injury and mortality in critically ill and septic patients, and that therefore HES should no longer be used in these patients. However the PRAC agreed that HES could continue to be used in patients with hypovolaemia caused by acute blood loss where treatment with alternative infusions solutions known as 'crystalloids' alone are not considered to be sufficient. The PRAC acknowledged the need for measures to minimise potential risks in these patients and recommended that HES solutions should not be used for more than 24 hours and that patients' kidney

7 Westferry Circus ● Canary Wharf ● London E14 4HB ● United Kingdom
Telephone +44 (0)20 7418 8400 **Facsimile** +44 (0)20 7523 7129
E-mail info@ema.europa.eu **Website** www.ema.europa.eu
An agency of the European Union

© European Medicines Agency, 2013. Reproduction is authorised provided the source is acknowledged.

function should be monitored for at least 90 days. In addition, the PRAC requested that further studies be carried out on the use of these medicines in elective surgery and trauma patients.

The PRAC recommendation will now be sent to the Coordination Group for Mutual Recognition and Decentralised Procedures – Human (CMDh), for consideration at its meeting on 21-23 October 2013.

More about the medicine

Infusion solutions containing HES are frequently used for volume replacement and belong to the classknown as colloids. There are two main types of medicines used for volume replacement: crystalloids and colloids. Colloids contain large molecules such as starch, whereas crystalloids, such as saline (salt) solutions or Ringer's acetate, contain smaller molecules. In the European Union (EU), HES-containing solutions for infusion have been approved via national procedures and are available in all Member States under various trade names.

More about the procedures

A review of HES solutions for infusion was initiated on 29 November 2012 at the request of the German medicines agency, under Article 31 of Directive 2001/83/EC. This review, which had been carried out by the Pharmacovigilance Risk Assessment Committee (PRAC), concluded on 13 June 2013, but some of the marketing authorisation holders requested a re-examination.

While the re-examination was ongoing, some Member States decided to suspend or limit the marketing or use of these medicines in their territories. In accordance with EU legislation, this type of action required that an EU review procedure be carried out. Consequently, on 27 June 2013, the UK triggered an EU review of HES solutions under Article 107i of Directive 2001/83/EC. This review procedure ran in parallel with the re-examination of the PRAC's June 2013 recommendation and both procedures were finalised on 10 October 2013. For the re-examination procedure the PRAC confirmed its previous position. However, new evidence was considered in the parallel Article 107i procedure and this was the basis for the PRAC's final recommendation on the use of HES solutions.

As HES-containing solutions for infusion are all authorised nationally, the PRAC recommendation will now be forwarded to the Co-ordination Group for Mutual Recognition and Decentralised Procedures – Human (CMDh), which will adopt a final position. The CMDh, a body representing EU Member States, is responsible for ensuring harmonised safety standards for medicines authorised via national procedures across the EU.

If the CMDh position is agreed by consensus, the agreement will be directly implemented by the Member States where the medicines are authorised. Should the CMDh position be adopted by majority vote, the CMDh position will be sent to the European Commission, for the adoption of an EU-wide legally binding decision.

Contact our press officers

Monika Benstetter or Martin Harvey

Tel. +44 (0)20 7418 8427

E-mail: press@ema.europa.eu

25 October 2013
EMA/640658/2013

Hydroxyethyl-starch solutions (HES) should no longer be used in patients with sepsis or burn injuries or in critically ill patients – CMDh endorses PRAC recommendations

HES will be available in restricted patient populations

The Coordination Group for Mutual Recognition and Decentralised Procedures – Human (CMDh)*,has endorsed by majority the recommendations of the European Medicines Agency's Pharmacovigilance Risk Assessment Committee (PRAC), which concluded that HES solutions must no longer be used to treat patients with sepsis (bacterial infection in the blood) or burn injuries or critically ill patients because of an increased risk of kidney injury and mortality.

The CMDh also agreed with the PRAC recommendation that HES solutions may continue to be used in patients to treat hypovolaemia (low blood volume) caused by acute (sudden) blood loss, where treatment with alternative infusions solutions known as 'crystalloids' alone are not considered to be sufficient. In order to minimise potential risks in these patients, HES solutions should not be used for more than 24 hours and patients' kidney function should be monitored after HES administration. In addition to updating the product information, further studies should be carried out on the use of these medicines in elective surgery and trauma patients.

The review of HES solutions was carried out by the PRAC following the publication of studies showing an increased risk of mortality in patients with sepsis[1,2] and an increased risk of kidney injury requiring dialysis in critically ill patients[1,2,3] following treatment with HES solutions.

As the CMDh position has been adopted by majority vote, it will now be sent to the European Commission, which will take a final legally binding decision that will be valid throughout the European Union (EU).

Information for patients

- Because of the risk of kidney injury and mortality, HES solutions must no longer be used in patients with sepsis (bacterial infection in the blood) or burn injuries or critically ill patients.
- HES solutions may continue to be used to treat hypovolaemia (low blood volume) caused by acute (sudden) blood loss. However, the doctor should monitor the patient's kidney function after HES administration.

* The CMDh is a medicines regulatory body representing the European Union (EU) Member States

7 Westferry Circus ● Canary Wharf ● London E14 4HB ● United Kingdom
Telephone +44 (0)20 7418 8400 **Facsimile** +44 (0)20 7523 7129
E-mail info@ema.europa.eu **Website** www.ema.europa.eu

An agency of the European Union

© European Medicines Agency, 2013. Reproduction is authorised the source is acknowledged.

- Patients who have any questions or concerns should speak to the treating doctor, pharmacist or nurse.

Information for healthcare professionals

- Because of the risk of kidney injury and mortality, HES solutions must no longer be used in patients with sepsis, burn injuries or critically ill patients.
- HES solutions should only be used for the treatment of hypovolaemia due to acute blood loss when crystalloids alone are not considered sufficient.
- There is a lack of robust long-term safety data in patients undergoing surgical procedures and in patients with trauma. The expected benefit of treatment should be carefully weighed against the uncertainties with regard to long-term safety, and other available treatment options should be considered. Additional studies will be performed with HES solutions in patients with trauma and in elective surgery.
- HES solutions should be used at the lowest effective dose for the shortest period of time. Treatment should be guided by continuous haemodynamic monitoring so that the infusion is stopped as soon as appropriate haemodynamic goals have been achieved.
- HES solutions are now contraindicated in patients with renal impairment or renal replacement therapy. The use of HES must be discontinued at the first sign of renal injury. An increased need for renal replacement therapy has been reported up to 90 days after HES administration. Patients' kidney function should be monitored after HES administration.
- HES solutions are contraindicated in severe coagulopathy. HES solutions should be discontinued at the first sign of coagulopathy. Blood coagulation parameters should be monitored carefully in case of repeated administration.

These recommendations are based on a review of all available safety and efficacy data, including recent data[4,5,6], from clinical studies, meta-analyses and post-marketing experience.

Healthcare professionals will be informed in writing of the outcome of the review and the changes to the use of HES solutions.

References:

1. Perner A, Haase N, Guttormsen AB et al. Hydroxyethyl starch 130/0.42 versus ringer's acetate in severe sepsis. N Engl J Med 2012;367(2):124-34
2. Brunkhorst FM, Engel C, Bloos F et al. Intensive Insulin Therapy and Pentastarch Resuscitation in Severe Sepsis. N Engl J Med 2008; 358(2):125-39
3. Myburgh J, Finder S, Bellomo R et al. Hydroxyethyl starch or saline for fluid resuscitation in intensive care. N Engl J Med 2012; 367:1901-11
4. Annane D. et al. CRISTAL: Colloids Compared to Crystalloids in Fluid Resuscitation of Critically Ill Patients: A Multinational Randomised Controlled Trial. NCT00318942. Available on: http://clinicaltrials.gov/ct2/show/NCT00318942
5. Siegemund M. Firstly presented at European Society of Anaesthesiology conference 2012. Basel Study for Evaluation of Starch (130;0.4) Infusion in Septic Patients:BaSES (130;0.4) Trial, listed at http://clinicaltrials.gov/show/NCT00273728

6. Rational Fluid Therapy in Germany (RaFTinG). Available on ClinicalTrials.gov (NCT01122277) last updated on 07 July 2011: http://clinicaltrials.gov/ct2/show/NCT01122277?term=NCT01122277&rank=1

More about the medicine

Infusion solutions containing HES are frequently used for volume replacement and belong to the class known as colloids. There are two main types of medicines used for volume replacement: crystalloids and colloids. Colloids contain large molecules such as starch, whereas crystalloids, such as saline (salt) solutions or Ringer's acetate, contain smaller molecules.

In the EU, HES-containing solutions for infusion have been approved via national procedures and are available in all Member States under various trade names.

More about the procedures

A review of HES solutions for infusion was initiated on 29 November 2012 at the request of the German medicines agency, under Article 31 of Directive 2001/83/EC. This review, which had been carried out by the Pharmacovigilance Risk Assessment Committee (PRAC), concluded on 13 June 2013, but some of the marketing authorisation holders requested a re-examination.

While the re-examination was ongoing, some Member States decided to suspend or limit the marketing or use of these medicines in their territories. In accordance with EU legislation, this type of action required that an EU review procedure be carried out. Consequently, on 27 June 2013, the UK triggered an EU review of HES solutions under Article 107i of Directive 2001/83/EC. This review procedure ran in parallel with the re-examination of the PRAC's June 2013 recommendation and both procedures were finalised on 10 October 2013. For the re-examination procedure the PRAC confirmed its previous position. However, new evidence was considered in the parallel Article 107i procedure and this was the basis for the PRAC's final recommendation on the use of HES solutions.

As HES-containing solutions for infusion are all authorised nationally, the PRAC recommendations were forwarded to the Co-ordination Group for Mutual Recognition and Decentralised Procedures – Human (CMDh), which adopted a final position. The CMDh, a body representing EU Member States, is responsible for ensuring harmonised safety standards for medicines authorised via national procedures across the EU.

As the CMDh position was adopted by majority vote, the CMDh position will now be sent to the European Commission, for the adoption of an EU-wide legally binding decision.

Contact our press officers

Monika Benstetter or Martin Harvey

Tel. +44 (0)20 7418 8427

E-mail: press@ema.europa.eu

EUROPEAN MEDICINES AGENCY
SCIENCE MEDICINES HEALTH

19 December 2013
EMA/809470/2013

Hydroxyethyl-starch solutions (HES) no longer to be used in patients with sepsis or burn injuries or in critically ill patients

HES will be available in restricted patient populations

On 23 October 2013, the Coordination Group for Mutual Recognition and Decentralised Procedures – Human (CMDh)*, endorsed by majority the recommendations of the European Medicines Agency's Pharmacovigilance Risk Assessment Committee (PRAC), which concluded that HES solutions must no longer be used to treat patients with sepsis (bacterial infection in the blood) or burn injuries or critically ill patients because of an increased risk of kidney injury and mortality.

HES solutions may continue to be used in patients to treat hypovolaemia (low blood volume) caused by acute (sudden) blood loss, where treatment with alternative infusions solutions known as 'crystalloids' alone are not considered to be sufficient. In order to minimise potential risks in these patients, HES solutions should not be used for more than 24 hours and patients' kidney function should be monitored after HES administration. In addition to updating the product information, further studies should be carried out on the use of these medicines in elective surgery and trauma patients.

The review of HES solutions was carried out by the PRAC following the publication of studies showing an increased risk of mortality in patients with sepsis[1,2] and an increased risk of kidney injury requiring dialysis in critically ill patients[1,2,3] following treatment with HES solutions.

As the CMDh position was adopted by majority vote, it was sent to the European Commission, which endorsed it and, on 19 December 2013, adopted a final legally binding decision valid throughout the European Union (EU).

Information for patients

- Because of the risk of kidney injury and mortality, HES solutions must no longer be used in patients with sepsis (bacterial infection in the blood) or burn injuries or critically ill patients.
- HES solutions may continue to be used to treat hypovolaemia (low blood volume) caused by acute (sudden) blood loss. However, the doctor should monitor the patient's kidney function after HES administration.

* The CMDh is a medicines regulatory body representing the European Union (EU) Member States

7 Westferry Circus ● Canary Wharf ● London E14 4HB ● United Kingdom
Telephone +44 (0)20 7418 8400 **Facsimile** +44 (0)20 7523 7129
E-mail info@ema.europa.eu **Website** www.ema.europa.eu

An agency of the European Union

© European Medicines Agency, 2014. Reproduction is authorised provided the source is acknowledged.

- Patients who have any questions or concerns should speak to the treating doctor, pharmacist or nurse.

Information for healthcare professionals

- Because of the risk of kidney injury and mortality, HES solutions must no longer be used in patients with sepsis, burn injuries or critically ill patients.
- HES solutions should only be used for the treatment of hypovolaemia due to acute blood loss when crystalloids alone are not considered sufficient.
- There is a lack of robust long-term safety data in patients undergoing surgical procedures and in patients with trauma. The expected benefit of treatment should be carefully weighed against the uncertainties with regard to long-term safety, and other available treatment options should be considered. Additional studies will be performed with HES solutions in patients with trauma and in elective surgery.
- HES solutions should be used at the lowest effective dose for the shortest period of time. Treatment should be guided by continuous haemodynamic monitoring so that the infusion is stopped as soon as appropriate haemodynamic goals have been achieved.
- HES solutions are now contraindicated in patients with renal impairment or renal replacement therapy. The use of HES must be discontinued at the first sign of renal injury. An increased need for renal replacement therapy has been reported up to 90 days after HES administration. Patients' kidney function should be monitored after HES administration.
- HES solutions are contraindicated in severe coagulopathy. HES solutions should be discontinued at the first sign of coagulopathy. Blood coagulation parameters should be monitored carefully in case of repeated administration.

These recommendations are based on a review of all available safety and efficacy data, including recent data[4,5,6], from clinical studies, meta-analyses and post-marketing experience.

Healthcare professionals have been informed in writing of the outcome of the review and the changes to the use of HES solutions.

References:

1. Perner A, Haase N, Guttormsen AB et al. Hydroxyethyl starch 130/0.42 versus ringer's acetate in severe sepsis. N Engl J Med 2012;367(2):124-34
2. Brunkhorst FM, Engel C, Bloos F et al. Intensive Insulin Therapy and Pentastarch Resuscitation in Severe Sepsis. N Engl J Med 2008; 358(2):125-39
3. Myburgh J, Finder S, Bellomo R et al. Hydroxyethyl starch or saline for fluid resuscitation in intensive care. N Engl J Med 2012; 367:1901-11
4. Annane D. et al. CRISTAL: Colloids Compared to Crystalloids in Fluid Resuscitation of Critically Ill Patients: A Multinational Randomised Controlled Trial. NCT00318942. Available on: http://clinicaltrials.gov/ct2/show/NCT00318942
5. Siegemund M. Firstly presented at European Society of Anaesthesiology conference 2012. Basel Study for Evaluation of Starch (130;0.4) Infusion in Septic Patients:BaSES (130;0.4) Trial, listed at http://clinicaltrials.gov/show/NCT00273728

6. Rational Fluid Therapy in Germany (RaFTinG). Available on ClinicalTrials.gov (NCT01122277) last updated on 07 July 2011: http://clinicaltrials.gov/ct2/show/NCT01122277?term=NCT01122277&rank=1

More about the medicine

Infusion solutions containing HES are frequently used for volume replacement and belong to the class known as colloids. There are two main types of medicines used for volume replacement: crystalloids and colloids. Colloids contain large molecules such as starch, whereas crystalloids, such as saline (salt) solutions or Ringer's acetate, contain smaller molecules.

In the EU, HES-containing solutions for infusion have been approved via national procedures and are available in all Member States under various trade names.

More about the procedures

A review of HES solutions for infusion was initiated on 29 November 2012 at the request of the German medicines agency, under Article 31 of Directive 2001/83/EC. This review, which had been carried out by the Pharmacovigilance Risk Assessment Committee (PRAC), concluded on 13 June 2013, but some of the marketing authorisation holders requested a re-examination.

While the re-examination was ongoing, some Member States decided to suspend or limit the marketing or use of these medicines in their territories. In accordance with EU legislation, this type of action required that an EU review procedure be carried out. Consequently, on 27 June 2013, the UK triggered an EU review of HES solutions under Article 107i of Directive 2001/83/EC. This review procedure ran in parallel with the re-examination of the PRAC's June 2013 recommendation and both procedures were finalised on 10 October 2013. For the re-examination procedure the PRAC confirmed its previous position. However, new evidence was considered in the parallel Article 107i procedure and this was the basis for the PRAC's final recommendation on the use of HES solutions.

As HES-containing solutions for infusion are all authorised nationally, the PRAC recommendations were forwarded to the Co-ordination Group for Mutual Recognition and Decentralised Procedures – Human (CMDh), which adopted a final position. The CMDh, a body representing EU Member States, is responsible for ensuring harmonised safety standards for medicines authorised via national procedures across the EU.

As the CMDh position was adopted by majority vote, it was sent sent to the European Commission, which endorsed it and adopted an EU-wide legally binding decision on 19 December 2013.

Contact our press officers

Monika Benstetter or Martin Harvey

Tel. +44 (0)20 7418 8427

E-mail: press@ema.europa.eu
