# Supplementary material for: Starch Wars—New Episodes of the Saga. Changes in Regulations on Hydroxyethyl Starch in the European Union
Source: Front Vet Sci. 2019 Jan 18;5:336. doi: 10.3389/fvets.2018.00336 (PMC6345713; doi:10.3389/fvets.2018.00336)
Supplement: Supplementary file 2 [file Data_Sheet_2.PDF]

Appendix 2 – Available online sources from US FDA used in this article

Index

1. 27th December 2007: FDA Approves Voluven to Treat Serious Blood Volume Loss following Surgery
2. FDA 2012 Workshop announcement
3. Black Box warning FDA 2013
4. FDA List of drugs withdrawn

FDA

Archived Content

The content on this page is provided for reference purposes only. This content has not been altered or updated since it was archived.

Search Archive

News & Events

FDA NEWS RELEASE

FOR IMMEDIATE RELEASE
December 27, 2007

Media Inquiries:
Peper Long, 301-827-6242
Consumer Inquiries:
888-INFO-FDA

FDA Approves Voluven to Treat Serious Blood Volume Loss following Surgery

This press release contains revisions posted Jan. 25, 2008 and Mar. 7, 2008.

The U.S. Food and Drug Administration today approved Voluven, an intravenous solution that prevents and treats a dangerous loss of blood volume, a condition that sometimes occurs during and after surgery.

Significant blood losses can cause a rapid drop in the volume of red blood cells and plasma circulating through the body. This car lead to shock, which is potentially fatal. Blood volume expanders are commonly administered to quickly restore some of the lost volume so that remaining red blood cells can continue to deliver needed oxygen to the body's tissues.

Voluven is manufactured from a water-insoluble starch modified to form hydroxyethyl starch. Hydroxyethyl starch-linked units (polymers) increase and maintain blood volume more effectively when used in combination with salt-and-water solutions.

"Massive blood loss is a life-threatening problem. Approval of Voluven provides clinicians with an alternative blood volume product that is safe and effective in a wide range of age groups," said Jesse L. Goodman, M.D., M.P.H., director of FDA's Center for Biologics Evaluation and Research.

In clinical trials, Voluven was compared to other approved blood volume expanders. During orthopedic surgery, Voluven was as safe and effective in expanding blood volume as Hetastarch, an approved starch solution.

In newborns and infants undergoing major surgery, Voluven was as safe and effective as an equivalent volume of another expander containing albumin, a protein found in the blood. In other trials conducted overseas, Voluven was as safe as other bloo volume expanders used in those countries in patients ranging in age from less than two years to 75 years who were undergoing a variety of surgical procedures.

The most common side effect from Voluven was itching.

Voluven is not recommended for the following:

- patients with known abnormal sensitivity to the synthetic starch used in the product
- patients experiencing fluid overload
- patients with kidney failure not related to low blood volume
- patients on dialysis
- patients with severe increases in blood levels of sodium or chloride
- patients with bleeding inside the head

Voluven was not studied in patients with sepsis, an infection of the blood. A post-market clinical trial involving patients with sepsis is planned.

Voluven (6% hydroxyethyl starch 130/0.4 in 0.9% sodium chloride injection) is manufactured by Fresenius Kabi, Bad Homburg, Germany.

#

RSS Feed for FDA News Releases[10] [what is RSS?[11]]

Page Last Updated: 04/09/2013
Note: If you need help accessing information in different file formats, see Instructions for Downloading Viewers and Players.
Language Assistance Available: Español | 繁體中文 | Tiếng Việt | 한국어 | Tagalog | Русский | العربية | Kreyòl Ayisyen | Français | Polski | Português | Italiano | Deutsch | 日本語 | فارسی | English

Accessibility Contact FDA Careers FDA Basics FOIA No FEAR Act Site Map Nondiscrimination Website Policies

FDA

U.S. Food and Drug Administration
10903 New Hampshire Avenue
Silver Spring, MD 20993
Ph. 1-888-INFO-FDA (1-888-463-6332)
Contact FDA

For GovernmentFor Press

U.S. Department of Health & Human Services

Links on this page:

1. https://wayback.archive-it.org/7993/20170112002645/http://www.addthis.com/bookmark.php?u508=true&v=152&username=fdamain
2. https://wayback.archive-it.org/7993/20170112002645/http://www.addthis.com/bookmark.php
3. /7993/20170112002645/http://www.fda.gov/default.htm
4. /7993/20170112002645/http://www.fda.gov/NewsEvents/default.htm
5. /7993/20170112002645/http://www.fda.gov/NewsEvents/Newsroom/default.htm
6. /7993/20170112002645/http://www.fda.gov/NewsEvents/Newsroom/PressAnnouncements/default.htm
7. /7993/20170112002645/http://www.fda.gov/NewsEvents/Newsroom/PressAnnouncements/2007/default.htm
8. /7993/20170112002645/http://www.fda.gov/NewsEvents/Newsroom/default.htm
9. /7993/20170112002645/http://www.fda.gov/NewsEvents/Newsroom/PressAnnouncements/default.htm
10. /7993/20170112002645/http://www.fda.gov/AboutFDA/ContactFDA/StayInformed/RSSFeeds/PressReleases/rss.xml
11. ssLINK/ucm144575.htm

the number of registrants requesting to speak is greater than can be reasonably accommodated during the scheduled open public hearing session, FDA may conduct a lottery to determine the speakers for the scheduled open public hearing session. The contact person will notify interested persons regarding their request to speak by August 22, 2012.

Persons attending FDA's advisory committee meetings are advised that the Agency is not responsible for providing access to electrical outlets.

FDA welcomes the attendance of the public at its advisory committee meetings and will make every effort to accommodate persons with physical disabilities or special needs. If you require special accommodations due to a disability, please contact Kalyani Bhatt at least 7 days in advance of the meeting.

FDA is committed to the orderly conduct of its advisory committee meetings. Please visit our Web site at *http://www.fda.gov/AdvisoryCommittees/AboutAdvisoryCommittees/ucm111462.htm* for procedures on public conduct during advisory committee meetings.

Notice of this meeting is given under the Federal Advisory Committee Act (5 U.S.C. app. 2).

Dated: July 19, 2012.

Jill Hartzler Warner,

Acting Associate Commissioner for Special Medical Programs.

[FR Doc. 2012–18095 Filed 7–24–12; 8:45 am]

BILLING CODE 4160–01–P

DEPARTMENT OF HEALTH AND HUMAN SERVICES

Food and Drug Administration

[Docket No. FDA–2012–N–0001]

Risks and Benefits of Hydroxyethyl Starch Solutions; Public Workshop

AGENCY: Food and Drug Administration, HHS.

ACTION: Notice of public workshop.

The Food and Drug Administration (FDA) is announcing a public workshop entitled: ''Risks and Benefits of Hydroxyethyl Starch Solutions.'' The purpose of this public workshop is to discuss new information on the risks and benefits of FDA-approved hydroxyethyl starch (HES) solutions.

The public workshop has been planned in partnership with the Department of Defense and the National Heart, Lung and Blood Institute, National Institutes of Health, and will include presentations and panel discussions with experts from academia, regulated industry, government, and other stakeholders.

Date and Time: The public workshop will be held on September 6, 2012, from 8:00 a.m. to 5:30 p.m., and September 7, 2012, from 8:30 a.m. to 1:00 p.m.

Location: The public workshop will be held at the Masur Auditorium, National Institutes of Health, 10 Center Dr., Bldg. 10, Clinical Center, Bethesda, MD 20892.

Contact Person: Jennifer Scharpf, Center for Biologics Evaluation and Research (HFM–300), Food and Drug Administration, 1401 Rockville Pike, suite 200N, Rockville, MD 20852–1448, Phone: 301–827–6128, FAX: 301–827–2843, email: *CBEROBRRWorkshops@fda.hhs.gov.*

Registration: Mail, fax, or email your registration information (including name, title, firm or organization name, address, telephone and fax numbers, and email address) to Jennifer Scharpf (see *Contact Person*) by August 15, 2012. There is no registration fee for the public workshop. Early registration is recommended because seating is limited. Registration on the day of the public workshop will be provided on a space available basis beginning at 7:00 a.m. If you need special accommodations due to a disability, please contact Jennifer Scharpf (see *Contact Person*) at least 7 days in advance.

SUPPLEMENTARY INFORMATION: HES solutions are synthetic colloids administered intravenously to patients to maintain or expand plasma volume when clinically indicated. Currently, three such products are approved by FDA. HES solutions are indicated for the treatment of hypovolemia (low blood volume) that may result from trauma, sepsis, burns, or anaphylaxis. These products are used in the prehospital and hospital environment in both military and civilian settings. This public workshop will serve as a forum for discussing new information on the potential effects of HES solutions on hemostasis and on the renal system.

The first day of the public workshop will include presentations and panel discussions on the following topics: (1) The risks and benefits associated with HES solutions in different clinical settings and (2) the findings of two recent major clinical studies conducted on HES solutions.

The second day of the public workshop will include a summary discussion and presentations concerning the overall safety profile of HES solutions and a discussion of future clinical research for the evaluation of HES solutions.

Transcripts: Please be advised that as soon as possible after a transcript of the public workshop is available, it will be accessible at: *http://www.fda.gov/BiologicsBloodVaccines/NewsEvents/WorkshopsMeetingsConferences/TranscriptsMinutes/default.htm.* Transcripts of the public workshop may also be requested in writing from the Division of Freedom of Information (ELEM–1029), Food and Drug Administration, 12420 Parklawn Dr., Rockville, MD 20857.

Dated: July 17, 2012.

Leslie Kux,

Assistant Commissioner for Policy.

[FR Doc. 2012–18110 Filed 7–24–12; 8:45 am]

BILLING CODE 4160–01–P

DEPARTMENT OF HEALTH AND HUMAN SERVICES

National Institutes of Health

Government-Owned Inventions; Availability for Licensing

AGENCY: National Institutes of Health, Public Health Service, HHS.

ACTION: Notice.

SUMMARY: The inventions listed below are owned by an agency of the U.S. Government and are available for licensing in the U.S. in accordance with 35 U.S.C. 207 to achieve expeditious commercialization of results of federally-funded research and development. Foreign patent applications are filed on selected inventions to extend market coverage for companies and may also be available for licensing.

FOR FURTHER INFORMATION CONTACT: Licensing information and copies of the U.S. patent applications listed below may be obtained by writing to the indicated licensing contact at the Office of Technology Transfer, National Institutes of Health, 6011 Executive Boulevard, Suite 325, Rockville, Maryland 20852–3804; telephone: 301–496–7057; fax: 301–402–0220. A signed Confidential Disclosure Agreement will be required to receive copies of the patent applications.

Novel Analogues of the Asthma Drug Fenoterol as Liver and Brain Cancer Therapeutic Agents

Description of Technology: Available for licensing are specific fenoterol analogues, such as MNF, that inhibit the growth of various types of cancers, including brain, liver, colon, and lung tumors. MNF acts as an agonist of the

FDA Safety Communication: Boxed Warning on increased mortality and severe renal injury, and additional warning on risk of bleeding, for use of hydroxyethyl starch solutions in some settings

Date: November 25, 2013 (Revised)

Safety Announcement Update-On November 25, 2013, the U.S. Food and Drug Administration (FDA) approved changes to the prescribing information for the class of hydroxyethyl starch products to add a new *Boxed Warning* about the risk of mortality and renal replacement therapy. The revised labeling also includes updates to the Contraindications, Warnings and Precautions as well as the Adverse Reactions and Clinical Studies section.

Summary of Safety Issue

Recommendations for Patients

Recommendations for Health Professionals

Data Summary

Summary of Safety Issues

Hydroxyethyl starch (HES) solutions are used for the treatment of hypovolemia (low blood volume) when plasma volume expansion is desired. Recent data have associated administration of these products with an increased risk of severe adverse events when used in certain patient populations.

On September 6-7, 2012, FDA convened a Public Workshop[1] in collaboration with the National Heart, Lung, and Blood Institute at the National Institutes of Health, the U.S. Army Materiel Command, Department of Defense, and the Office of the Assistant Secretary of Health, Health and Human Services, to discuss the risks and benefits of HES solutions. Panelists presented data from randomized controlled trials (RCTs), meta-analyses and observational studies (described below in the data summary) that showed increased mortality and/or renal replacement therapy (RRT), i.e., severe renal injury, when HES was used in critically ill adult patients, including patients with sepsis.

FDA has completed its review of the data from the above studies, and an additional RCT study. FDA finds that these studies indicate increased mortality and RRT in critically ill adult patients, including patients with sepsis who are treated with HES solutions. FDA has concluded that HES solutions should not be used in these patient populations, and that a Boxed Warning to highlight the risk of mortality and RRT is warranted. In addition, FDA has reviewed a meta-analysis of studies conducted in patients undergoing open heart surgery in association with cardiopulmonary bypass, and has determined that an additional warning about excessive bleeding is needed in the Warnings and Precautions Section of the package insert.

top

Recommendations for Patients

Patients should be aware of the risks associated with the use of HES solutions and discuss these risks with their healthcare provider.

- Be aware that severe kidney damage has been associated with the use of HES solutions.
- Be sure to follow up with your healthcare provider as requested and follow all instructions. Report any unusual symptoms immediately.
- Symptoms of kidney damage can include:
  - change in the frequency, amount, or color of urine
  - blood in the urine
  - difficulty urinating
  - swelling of the legs, ankles, feet, face, or hands
  - unusual weakness or fatigue
  - nausea and vomiting
  - shortness of breath

top

Recommendations for Health Professionals

- Do not use HES solutions in critically ill adult patients, including those with sepsis.
- Avoid use in patients with pre-existing renal dysfunction.
- Discontinue use of HES at the first sign of renal injury.
- Need for renal replacement therapy has been reported up to 90 days after HES administration. Continue to monitor renal function for at least 90 days in all hospitalized patients.
- Monitor the coagulation status of patients undergoing open heart surgery in association with cardiopulmonary bypass as excess bleeding has been reported with HES solutions in this population.[a] Discontinue use of HES at the first sign of coagulopathy.
- Do not use HES products in patients with severe liver disease.
- Monitor liver function in patients receiving HES products.

[a]Hespan (6% HES 450/0.7 in 0.9% Sodium Chloride Injection) is not recommended for use as a cardiac bypass pump prime, while the patient is on cardiopulmonary bypass, or in the immediate period after the pump has been discontinued because of the risk of increasing coagulation abnormalities and bleeding in patients whose coagulation status is already impaired.

top

Data Summary

Mortality and renal injury requiring renal replacement therapy (RRT)

Four HES products are currently FDA approved for the treatment and prophylaxis of hypovolemia: HESPAN (6% HES 450/0.7[b] in 0.9% Sodium Chloride Injection; B. Braun Medical Inc), Hetastarch (6% in 0.9% Sodium Chloride Injection, generic equivalent to HESPAN; Teva Pharmaceuticals USA), HEXTEND (6% HES 450/0.7 in physiological solution; BioTime Inc), and Voluven (6% HES 130/0.40 in normal saline; Fresenius Kabi USA, LLC).

Data from randomized controlled trials (RCTs), meta-analyses and observational studies show increased mortality and renal injury requiring RRT in critically ill patients, including patients with sepsis, who are treated with HES. The safety of higher molecular weight (molecular weight: 450 kDa) HES products, (HESPAN, Hetastarch (6%) in 0.9% Sodium Chloride Injection and HEXTEND) was assessed in retrospective studies and meta-analyses. In its analysis, FDA extrapolated safety data from the lower molecular weight HES product to higher molecular weight HES products. This extrapolation was justified because of similarities in chemical structure and mechanism of action between the higher and lower molecular weight HES products. In addition, both higher and lower molecular weight formulations are metabolized by α-amylase into similar smaller fragments until the renal threshold of excretion (45-60 kDa) is reached.[2] This fact implies that exposure to smaller molecular weight fragments occurs when higher molecular weight HES products are administered, so that renal toxicity may be anticipated.

Renal injury was not evident in a review of 59 RCTs in which HES products were administered in the operating room to adult and pediatric patients who were undergoing surgery and were followed for a short period of time, i.e., < 7days.[3] Possible explanations for the lack of observed toxicity in these surgical populations include low exposure levels; administration of HES to a medically-optimized, comparatively healthy surgery population; short follow-up monitoring; and/or other unknown factor(s).

Based on the totality of the evidence, FDA considers increased mortality and RRT in critically ill adult patients, including patients with sepsis.

Randomized controlled trials, meta-analyses, and observational studies

Increased mortality and/or renal injury requiring RRT in critically ill adult patients, including those with sepsis have been reported in three double-blind, multicenter RCTs published in 2012 comparing HES with crystalloid solution in which treated patients were monitored for 90 days.

- The *6S* study compared 6% HES 130/0.42 with Ringer's acetate (not licensed in US) for treatment of hypovolemia in a large population (N=804) of patients with severe sepsis. Death or dialysis-dependence at 90 days were co-primary endpoints; incidence of RRT was a secondary endpoint. Total volume of trial fluid administered (median) was 1500 mL on Day 1, 1500 mL on Day 2, and 1000 mL on Day 3. Mortality (201/398 vs. 172/400; p=0.03) increased independently of increased RRT (87/398 vs. 65/400; p=0.04) in the HES treatment arm. This study demonstrated both increased mortality and serious renal injury at labeled doses of HES, confirming its toxicity.[4]
- The *CRYSTMAS* study compared 6% HES 130/0.4 with normal saline in a smaller population (N=196) of severe sepsis patients, as compared to the 6S study. Volume of trial fluid needed to achieve hemodynamic stabilization was the primary endpoint; RRT was a secondary endpoint. Total volume of trial fluid administered (median) was 1000 mL on Day 1, and 500 mL/day on Days 2, 3 and 4, respectively. The difference in mortality was in the direction of an increase with Voluven (40/100 vs. 32/96), but did not reach statistical significance (p=0.33). A trend to increased RRT (p=0.06) was reported in the HES treatment arm (21/100 vs. 11/96).[5]
- The *CHEST* study (published after the FDA Public Workshop in 2012) compared 6% HES 130/0.40 with normal saline in a heterogeneous population of adult patients treated in the ICU (N=7000) that included patients with sepsis (N=1937) as well as elective surgery patients and patients with APACHE II score ≥ 25. The primary endpoint was death or dialysis dependency at Day 90. Total volume of trial fluid administered (median) was 1000 mL on Day 0, and 500 mL/day on Day 1, Day 2, and Day 3. The difference in mortality (597/3315 for HES vs. 566/3336 for saline) did not reach statistical significance. HES subjects experienced significantly greater need for RRT (235/3315 vs. 196/3336, p=0.04), but the incidence of RRT in the sepsis subgroup was not reported.[6]

Meta-analyses and observational studies lend additional support to these findings.

- A Cochrane Collaboration meta-analysis of 34 RCTs using different HES products (130/0.4, 200/0.5, 200/0.6, 70/0.5, 200/0.62, and 450/0.7) to treat hypovolemia found that in a subgroup of studies that captured RRT (9 studies, N=1333) or author-defined kidney failure (12 studies, N=1260) as secondary renal outcomes, a significant increase was observed in HES-treated sepsis patients; this was not observed in HES-treated trauma/surgery patients. The HES used in these studies included 6% HES 130/0.4 (Voluven), 6% HES 130/0.42, 6% HES 200/0.6, and 10% HES 200/0.5.[7]
- Increased mortality and renal injury requiring RRT were reported in four meta-analyses of RCTs in which different HES formulations were used for fluid resuscitation in critically ill adult patients (N=3156 to 10,391), including patients with sepsis. Most of these studies used 6% HES 130/0.4-0.042. [8,9,10,11]
- A single-arm, prospective, observational analysis of adults with severe sepsis (N=1046) who received only one type of colloid for hypovolemia over a 6-year study period reported increased RRT in those receiving Voluven (relative risk, 2.01; 95% CI, 1.34 to 3.02; p<0.001) from 2004 to 2006 compared to those receiving crystalloid from 2008 to 2010.[12]
- A retrospective evaluation of cardiac surgery patients (N=563) found that pentastarch (10% HES 200/0.45) was independently associated with acute kidney injury (AKI, prespecified as a 50% rise in serum creatinine within 4 days): relative risk 1.08 (1.04 to 1.12; p=0.001). Risk of AKI was dose-dependent, with doses ≥14 mL/kg predicting AKI.[13]
- A retrospective study of trauma patients (N=2225), 22% (N=497) of whom received HES 450/0.7 as part of their fluid resuscitation regimen, reported increased risk of acute kidney injury: relative risk 1.73 (1.30 to 2.28); increased mortality: relative risk 1.84 (1.48 to 2.29); and increased risk of death or acute kidney injury: relative risk 1.90 (1.59 to 2.27) in HES patients.[14]

FDA considers increased mortality and renal injury requiring RRT in critically ill adult patients, including patients with sepsis, to be a class effect warranting addition of this new safety information in a Boxed Warning.

top

Excess bleeding

In a meta-analysis of 18 RCTs in patients undergoing open heart surgery in association with cardiopulmonary bypass,[15] use of different HES products, irrespective of molecular weight or degree of molar substitution, was associated with increased bleeding. FDA considers excess bleeding a class effect warranting addition of this new safety information to the Warning and Precautions Section of the PI.>

top

References

1. Public Workshop – Risks and Benefits of Hydroxyethyl Starch Solutions **http://www.fda.gov/BiologicsBloodVaccines/NewsEvents/WorkshopsMeetingsConferences/ucm313370.htm (http://wayback.archive-it.org/7993/20170112095648/http://www.fda.gov/BiologicsBloodVaccines/NewsEvents/WorkshopsMeetingsConferences/ucm313370.htm)**
2. Westphal M, James MFM, Kozek-Langenecker S, et al. Hydroxyethyl starches: different products – different effects. Anesthesiology 2009;111:187-202
3. Van der Linden P, James M, Mythen M, et al. Safety of modern starches used during surgery. Anesth Analg 2013;116:35-48
4. Perner A, Haase N, Guttormsen AB, et al. Hydroxyethyl starch 130/0.4 versus Ringer's acetate in severe sepsis. N Engl J Med 2012;367:124-34. [Erratum, N Engl J Med 2012;367:481]
5. Guidet B, Martinet O, Boulain T, et al. Assessment of hemodynamic efficacy and safety of 6% hydroxyethyl starch 130/0.4 vs. 0.9% NaCl fluid replacement in patients with severe sepsis: The CRYSTMAS study. Critical Care 2012, 16:R94
6. Myburgh JA, Finfer S, Bellomo R, et al. Hydroxyethyl starch or saline for fluid resuscitation in intensive care. N Engl J Med 2012;367:1901-11
7. Dart AB, Mutter TC, Ruth CA, et al. Hydroxyethyl starch (HES) versus other fluid therapies: effects on kidney function. Cochrane Database of Systematic Reviews 2010;Jan 20;1:CD007594
8. Haase N, Perner A, Hennings LI, et al. Hydroxyethyl starch 130/0.38-0.45 versus crystalloid or albumin in patients with sepsis: systematic review with meta-analysis and trial sequential analysis. BMJ 2013;1839 doi: 10.1136/bmj.f839

9. Gattas DJ, Dan A, Myburgh J, et al. Fluid resuscitation with 6% hydroxyethyl starch (130/0.4 and 130/0.42) in acutely ill patients: systemic review of effects on mortality and treatment with renal replacement therapy. Intensive Care Med 2013; doi 10.1007/s00134-013-2840-0
10. Zarychanski R, Abou-Setta AM, Turgeon AF et al. Association of hydoxyethyl starch administration with mortality and acute kidney injury in critically ill patients requiring volume resuscitation: a systemic review and meta-analysis. JAMA 2013;309:678-688
11. Patel A, Waheed U, Brett SJ. Randomised trials of 6% tetrastarch (hydroxyethyl starch 130/0.4 or 0.42) for severe sepsis reporting mortality: systematic review and meta-analysis. Intensive Care Med 2013; DOI 10.1007/s00134-013-2863-6
12. Bayer O, Reinhart K, Kohl M, et al. Effects of fluid resuscitation with synthetic colloids alone on shock reversal, fluid balance, and patient outcomes in patients with severe sepsis: a prospective sequential analysis. Crit Care Med 2012;40:2543-2551
13. Rioux JP, Lessard M, De Bortoli B, et al. Pentastarch 10% (250 kDa/0.45) is an independent risk factor of acute kidney injury following cardiac surgery. Critical care medicine 2009;37: 1293-1298
14. Lissauer ME, Chi A, Kramer ME, et al. Association of 6% Hetastarch resuscitation with adverse outcomes in critically ill trauma patients. Am J Surgery 2011;202:53-8
15. Navickis RJ, Haynes GR, Wilkes MM. Effect of hydroxyethyl starch on bleeding after cardiopulmonary bypass: a meta-analysis of randomized trials. J Thorac Cardiovasc Surg 2012:144:223-30

top

Related Information

- **Public Workshop: Risks and Benefits of Hydroxyethyl Starch Solutions (/7993/20170112095648/http://www.fda.gov/BiologicsBloodVaccines/NewsEvents/WorkshopsMeetingsConferences/ucm313370.htm)**
- **Questions and Answers on FDA's Adverse Event Reporting System (FAERS) (/7993/20170112095648/http://www.fda.gov/Drugs/GuidanceComplianceRegulatoryInformation/Surveillance/AdverseDrugEffects/default.htm)**
- **MedWatch: The FDA Safety Information and Adverse Event Reporting Program (/7993/20170112095648/http://www.fda.gov/Safety/MedWatch/default.htm)**

Contact FDA

(800) 835-4709
(240) 402-8010
ocod@fda.hhs.gov (mailto:ocod@fda.hhs.gov)

Consumer Affairs Branch (CBER)
Division of Communication and Consumer Affairs
Office of Communication, Outreach and Development
Food and Drug Administration
10903 New Hampshire Avenue
Building 71 Room 3103
Silver Spring, MD 20993-0002

(2) If no paint contamination is detected on the actuator pistons and the moisture indicator of the trim actuator is pink or white, prior to further flight, replace the trim actuator with a new or serviceable trim actuator and either replace or regenerate the desiccant in accordance with the alert service bulletin.

(3) If any paint contamination is detected on the actuator pistons, prior to further flight, remove the paint in accordance with the alert service bulletin.

Note 2: Aviac Technologies, the manufacturer of the desiccant, has issued Identification Procedure for Desiccant DAV/AP98–214, Revision 0, dated April 22, 1998, as an additional source of service information to determine the level of saturation of the desiccant.

(b) Within 2 months after the effective date of this AD, perform a one-time visual inspection to verify installation of the flat gasket in each end of the flex drive, and to determine if the flat gasket is in good condition (i.e., shows no signs of wear), in accordance with Dornier Alert Service Bulletin ASB–328–27–017, Revision 2, dated July 28, 1998.

(1) If the gasket is installed and in good condition, no further action is required by paragraph (b) of this AD.

(2) If the gasket is missing or is installed and not in good condition, prior to further flight, replace the gasket with a new gasket, and torque the nuts, in accordance with the alert service bulletin.

Note 3: Accomplishment of the actions required by paragraphs (a) and (b) of this AD, prior to the effective date of this AD, in accordance with Dornier Alert Service Bulletin ASB–328–27–017, Revision 1, dated October 1, 1997, is considered acceptable for compliance with the applicable actions specified in paragraphs (a) and (b) of this AD.

(c) An alternative method of compliance or adjustment of the compliance time that provides an acceptable level of safety may be used if approved by the Manager, International Branch, ANM–116, FAA, Transport Airplane Directorate. Operators shall submit their requests through an appropriate FAA Principal Maintenance Inspector, who may add comments and then send it to the Manager, International Branch, ANM–116.

Note 4: Information concerning the existence of approved alternative methods of compliance with this AD, if any, may be obtained from the International Branch, ANM–116.

(d) Special flight permits may be issued in accordance with §§ 21.197 and 21.199 of the Federal Aviation Regulations (14 CFR 21.197 and 21.199) to operate the airplane to a location where the requirements of this AD can be accomplished.

Note 5: The subject of this AD is addressed in German airworthiness directive 97–188, dated July 3, 1997.

Issued in Renton, Washington, on October 1, 1998.

Darrell M. Pederson,

Acting Manager, Transport Airplane Directorate, Aircraft Certification Service.

[FR Doc. 98–26964 Filed 10–7–98; 8:45 am]

BILLING CODE 4910–13–P

DEPARTMENT OF HEALTH AND HUMAN SERVICES

Food and Drug Administration

21 CFR Part 216

[Docket No. 98N–0655]

List of Drug Products That Have Been Withdrawn or Removed From the Market for Reasons of Safety or Effectiveness

AGENCY: Food and Drug Administration, HHS.

ACTION: Proposed rule.

SUMMARY: The Food and Drug Administration (FDA) is proposing to amend its regulations to include a list of drug products that may not be used for pharmacy compounding pursuant to the exemptions under section 503A of the Federal Food, Drug, and Cosmetic Act (the act) because they have had their approval withdrawn or were removed from the market because the drug product or its components have been found to be unsafe or not effective. The list has been compiled under the new statutory requirements of the Food and Drug Administration Modernization Act of 1997 (Modernization Act).

DATES: Comments must be received on or before November 23, 1998.

ADDRESSES: Submit written comments to the Dockets Management Branch (HFA–305), Food and Drug Administration, 5630 Fishers Lane, rm. 1061, Rockville, MD 20852.

FOR FURTHER INFORMATION CONTACT: Wayne H. Mitchell, Center for Drug Evaluation and Research (HFD–7), Food and Drug Administration, 5600 Fishers Lane, Rockville, MD 20857, 301–594–2041.

SUPPLEMENTARY INFORMATION:

I. Background

President Clinton signed the Modernization Act (Pub. L. 105–115) into law on November 21, 1997. One of the issues addressed in this new legislation is the applicability of the act to the practice of pharmacy compounding. Compounding involves a process whereby a pharmacist or physician combines, mixes, or alters ingredients to create a customized medication for an individual patient. Section 127 of the Modernization Act, which adds section 503A to the act (21 U.S.C. 353a), describes the circumstances under which compounded drugs qualify for exemptions from certain adulteration, misbranding, and new drug provisions of the act (i.e., 501(a)(2)(B), 502(f)(1), and 505 of the act (21 U.S.C. 351(a)(2)(B), 352(f)(1), and 355)). Section 127(b) of the Modernization Act provides that section 503A of the act will become effective on November 21, 1998, 1 year from the date of the Modernization Act's enactment.

Section 503A of the act contains several conditions that must be satisfied for pharmacy compounding to qualify for the exemptions under section 503A. One of the conditions is that the licensed pharmacist or licensed physician does not "compound a drug product that appears on a list published by the Secretary in the **Federal Register** of drug products that have been withdrawn or removed from the market because such drug products or components of such drug products have been found to be unsafe or not effective."

II. Rulemaking to Establish the List

In accordance with section 503A of the act, FDA has developed a list of drug products that have been withdrawn or removed from the market because they have been found to be unsafe or not effective. Many of the drug products on the list were withdrawn from the market through official proceedings, including publication of a notice in the **Federal Register**. For these drug products, this preamble to the proposed rule includes the reason for the withdrawal and the citation to the official notice of withdrawal. Other products, both approved and unapproved, were removed from the market voluntarily by the manufacturer or application holder, and FDA has information indicating that the reason for the removal was because the product was unsafe or not effective. In such cases, the reason for the removal is provided, and additional sources of information on the drug can be found in the docket identified by the number found in brackets in the heading of this document.

This proposed rule is the first of a series of rulemaking proceedings to establish the list of withdrawn or removed drug products, as the development and issuance of this list will be an ongoing process. The primary focus of this proposed rule is drug products that have been removed or withdrawn for safety reasons. FDA intends that future rulemaking

proceedings will focus on drug products that were withdrawn for reasons of effectiveness, on drug products that are identified as having been withdrawn for reasons of safety or effectiveness after the preparation of this proposed rule, and on additional drug products that will be proposed for inclusion on the list either during the comment period or subsequently.

FDA is specifically seeking comment on whether additional drug products should be added to the list and whether products now on the list should remain on the list. Persons submitting comments recommending that a drug product be added to the list should include appropriate documentation, including any notices published in the **Federal Register**. In addition, individuals and organizations may petition FDA to amend the list at any time through the regular citizen petition process described in 21 CFR 10.30.

After evaluating the comments on this proposed rule and consulting an advisory committee on compounding, as required by section 503A(d)(1) of the act, FDA will issue the list as a final rule which will be codified in the Code of Federal Regulations. The initial list published as a final rule may include all or some of the products proposed for inclusion on the list in this proposal, depending upon the comments received. Additional products will be added to the list through the rulemaking process after the data on the products are evaluated, and after consultation with the advisory committee on compounding.

III. Description of the Proposed Rule

FDA is proposing that the drug products described in this section be included in the list of drug products that have been withdrawn or removed from the market because such drug products or components of such drug products have been found to be unsafe or not effective. Compounding a drug product that appears on this list is not covered by the exemption provided in section 503A(a) of the act, and may be subject to enforcement action under sections 501(a)(2)(B), 502(f)(1), and 505 (among other applicable provisions) of the act.

The listings are arranged alphabetically by the established name of the active ingredient contained in the drug product. For many of the drugs, the proprietary or trade name of some or all of the drug products which contained the active ingredient are also given in the preamble paragraphs describing the withdrawn or removed drug products. Some of the drugs listed were withdrawn or removed from the market based on problems relating only to one dosage form or route of administration. In such cases, the listing for that drug product reflects that fact, e.g., ''*Neomycin Sulfate*: Parenteral drug products containing neomycin sulfate.'' In other cases, the problem is associated with the active ingredient, or appears to relate to other dosage forms or routes of administration, and the listing reflects that fact, e.g., ''*Adrenal Cortex*: All drug products containing adrenal cortex.'' In several instances, a particular formulation, dosage form, or route of administration is explicitly excluded from an entry on the list because there is an approved drug (that has not been withdrawn or removed from the market) that contains the same active ingredient(s) as the drug product that has been withdrawn or removed from the market. In these instances, the listing includes the appropriate qualification, e.g., ''*Suprofen*: All drug products containing suprofen (except ophthalmic solutions).''

In several cases, the withdrawn drug products are identified according to the established name of the active ingredient, listed as a particular salt or ester of the active moiety, e.g., ''*Dexfenfluramine hydrochloride*: All drug products containing dexfenfluramine hydrochloride.'' Although the specific listing may be limited to a particular salt or ester, other salts or esters of the active moiety will not qualify for the compounding exemptions in section 503A of the act unless (among other requirements) the particular salt or ester is the subject of a United States Pharmacopeia or National Formulary monograph; is a component of an FDA approved drug; or appears on the FDA list of bulk drug substances that may be used for compounding. (See section 503A(b)(1)(A)(i) of the act).

The list is being proposed as § 216.24 of Title 21 of the Code of Federal Regulations. This new section will be included in a new part, part 216, which is currently intended to include all FDA regulations whose primary purpose is implementation of the pharmacy compounding provisions found in section 503A of the act.

The following drug products are proposed for inclusion in proposed § 216.24. The supporting documentation for each listed drug product may be found in the docket identified by the number found in brackets in the heading of this document. The supporting documentation will be arranged alphabetically according to the established name of the active ingredient of the drug products.

Adenosine phosphate: All drug products containing adenosine phosphate. Adenosine phosphate, formerly marketed as a component of Adeno for injection, Adco for injection, and other drug products, was determined to be neither safe nor effective for its intended uses as a vasodilator and an anti-inflammatory. FDA directed the removal of these drug products from the market in 1973.

Adrenal cortex: All drug products containing adrenal cortex. The low level of corticosteroids found in adrenal cortex injection and adrenal cortex extract were determined to present a substantial risk of undertreatment of serious conditions, such as adrenal cortical insufficiency, burns, and hypoglycemia. FDA determined that adrenal cortex for injection and adrenal cortex extract presented a significant potential hazard and directed the removal of these drug products from the market in January 1978.

Azaribine: All drug products containing azaribine. The use of azaribine, formerly marketed as Triazure tablets, was associated with very serious thromboembolic events. Approval of the new drug application (NDA) for Triazure tablets was withdrawn June 10, 1977 (see the **Federal Register** of June 10, 1977 (42 FR 29998)).

Benoxaprofen: All drug products containing benoxaprofen. The use of benoxaprofen, formerly marketed as Oraflex tablets, was associated with fatal cholestatic jaundice among other serious adverse reactions. The holder of the approved application voluntarily withdrew Oraflex tablets from the market on August 5, 1982.

Bithionol: All drug products containing bithionol. Bithionol, formerly marketed as an active ingredient in various topical drug products, was shown to be a potent photosensitizer with the potential to cause serious skin disorders. Approvals of the NDA's for bithionol drug products were withdrawn on October 24, 1967 (see the **Federal Register** of October 31, 1967 (32 FR 15046)).

Bromfenac sodium: All drug products containing bromfenac sodium. The use of bromfenac sodium, formerly marketed as Duract capsules, was associated with fatal hepatic failure. Duract capsules were voluntarily withdrawn from the market by their manufacturer on June 22, 1998.

Butamben: All parenteral drug products containing butamben. The use of a parenteral drug product containing butamben, formerly marketed as Efocaine, was associated with severe adverse reactions, such as severe tissue slough and transverse myelitis.

Approval of the NDA for Efocaine was withdrawn on August 7, 1964 (see the **Federal Register** of August 14, 1964 (29 FR 11656)).

Camphorated oil: All drug products containing camphorated oil. Products containing camphorated oil were associated with poisoning in infants and young children due to accidental ingestion. FDA directed the removal from the market of drug products containing camphorated oil in 1982 (see 21 CFR 310.526 (1997)).

Carbetapentane citrate: All oral gel drug products containing carbetapentane citrate. Carbetapentane citrate gel, formerly marketed as Candette Cough Jel, was determined not to be safe because the inexact methods of measuring the gel by consumers were potentially dangerous. Approval of the NDA for Candette Cough Jel was withdrawn on November 29, 1972 (see the **Federal Register** of November 29, 1972 (37 FR 25249)).

Casein, iodinated: All drug products containing iodinated casein. Iodinated casein, formerly marketed as a component of Neo-Barine, was associated with thyrotoxic side effects. Approval of the NDA for Neo-Barine was withdrawn October 22, 1964 (see the **Federal Register** of October 28, 1964 (29 FR 14676)).

Chlorhexidine gluconate: All tinctures of chlorhexidine gluconate formulated for use as a patient preoperative skin preparation. Chlorhexidine gluconate topical tincture 0.5%, formerly marketed as Hibitane, was associated with chemical and thermal burns when used as a patient preoperative skin preparation. The drug product was voluntarily removed from the market in early 1984. FDA determined that chlorhexidine gluconate topical tincture 0.5% was removed from the market for reasons of safety (see the **Federal Register** of October 6, 1997 (62 FR 52137)).

Chlormadinone acetate: All drug products containing chlormadinone acetate. Chlormadinone acetate, formerly marketed as a component of the combination drug products Estalor-21 and C-Quens tablets, was associated with the development of mammary tumors in dogs. The manufacturer ceased marketing the drug in 1970 and approvals of the NDA's for Estalor-21 and C-Quens tablets were withdrawn by FDA on March 16, 1972 (see the **Federal Register** of March 16, 1972 (37 FR 5516)).

Chloroform: All drug products containing chloroform. National Cancer Institute studies demonstrated that chloroform is carcinogenic in animals. FDA directed the removal from the market of drug products containing chloroform in 1976 (see 21 CFR 310.513 (1997)).

Cobalt: All drug products containing cobalt salts (except radioactive forms of cobalt and its salts and cobalamin and its derivatives). FDA found that cobalt salts were not safe or effective for treatment of iron-deficiency anemia. The toxic effects of cobalt salts include liver damage, claudication, and myocardial damage. FDA directed the removal from the market of drug products containing cobalt salts in 1967 (see 21 CFR 250.106 (1997)).

Dexfenfluramine hydrochloride: All drug products containing dexfenfluramine hydrochloride. Dexfenfluramine hydrochloride, formerly marketed as Redux capsules, was associated with valvular heart disease. The manufacturer of dexfenfluramine hydrochloride capsules voluntarily withdrew the drug from the market in September 1997.

Diamthazole dihydrochloride: All drug products containing diamthazole dihydrochloride. Diamthazole dihydrochloride, formerly marketed as Asterol ointment, powder, and tincture, was associated with neurotoxicity. Approvals of the NDA's for Asterol ointment, powder, and tincture were withdrawn on July 19, 1977 (see the **Federal Register** of July 19, 1977 (42 FR 37057)).

Dibromsalan: All drug products containing dibromsalan. Dibromsalan, formerly marketed in a number of drug products, largely antibacterial soaps, as an antimicrobial, preservative, or for other purposes, was, with other halogenated salicylanilides listed in this proposal, found to be a potent photosensitizer capable of causing disabling skin disorders. FDA directed the removal from the market of drug products containing dibromsalan in 1975 (see § 310.508 (21 CFR 310.508) (1997)).

Diethylstilbestrol: All oral and parenteral drug products containing 25 milligrams (mg) or more of diethylstilbestrol per unit dose. Diethylstilbestrol, marketed in various tablet and parenteral drug products, was associated with adenocarcinoma of the vagina in the offspring of the patient when used in early pregnancy. Approvals of the NDA's for these diethylstilbestrol drug products were withdrawn on February 18, 1975 (see the **Federal Register** of February 5, 1975 (40 FR 5384)).

Dihydrostreptomycin sulfate: All drug products containing dihydrostreptomycin sulfate. Dihydrostreptomycin sulfate, formerly marketed in several parenteral drug products, was associated with ototoxicity. Approvals of the NDA's for dihydrostreptomycin sulfate drug products were withdrawn on July 20, 1970 (see the **Federal Register** of September 3, 1970 (35 FR 13988)).

Dipyrone: All drug products containing dipyrone. Dipyrone, formerly marketed as Dimethone tablets and injection, Protemp oral liquid, and other drug products, was associated with potentially fatal agranulocytosis. Approvals of the NDA's for dipyrone drug products were withdrawn on June 27, 1977 (see the **Federal Register** of June 17, 1977 (42 FR 30893)).

Encainide hydrochloride: All drug products containing encainide hydrochloride. Encainide hydrochloride, formerly marketed as Enkaid capsules, was associated with increased death rates in patients who had asymptomatic heart rhythm abnormalities after a recent heart attack. The manufacturer of Enkaid capsules voluntarily withdrew the product from the market on December 16, 1991.

Fenfluramine hydrochloride: All drug products containing fenfluramine hydrochloride. Fenfluramine hydrochloride tablets, formerly marketed as Pondimin tablets, were associated with valvular heart disease. The manufacturer of fenfluramine hydrochloride tablets voluntarily withdrew the drug from the market in September 1997.

Flosequinan: All drug products containing flosequinan. Flosequinan, formerly marketed as Manoplax tablets, was the subject of a study that indicated the drug had adverse effects on survival, and that beneficial effects on the symptoms of heart failure did not last beyond the first 3 months of therapy. After the first 3 months of therapy, patients on the drug had a higher rate of hospitalization than patients taking a placebo. The manufacturer of Manoplax tablets voluntarily withdrew the drug from the market in July 1993.

Gelatin: All intravenous drug products containing gelatin. Gelatin for intravenous use, formerly marketed as Knox Special Gelatine Solution Intravenous-6 percent, was found not to be suitable as a plasma expander because the drug caused increased blood viscosity, reduced blood clotting, and prolonged bleeding time. Approval of the NDA for Knox Special Gelatine Solution Intravenous-6 percent was withdrawn on April 19, 1978 (see the **Federal Register** of April 7, 1978 (43 FR 14743)).

Glycerol, iodinated: All drug products containing iodinated glycerol. Iodinated glycerol, formerly marketed as Iodur Elixir and other drug products, was

found to have carcinogenic potential. FDA directed the removal from the market of drug products containing iodinated glycerol in April 1993.

Gonadotropin, chorionic: All drug products containing chorionic gonadotropins of animal origin. Chorionic gonadotropins of animal origins, formerly marketed as Synapoidin Steri-Vial, were shown to produce allergic reactions. Approval of the NDA for Synapoidin Steri-Vial was withdrawn on July 6, 1972 (see the **Federal Register** of July 6, 1972 (37 FR 13284)).

Mepazine: All drug products containing mepazine hydrochloride or mepazine acetate. Mepazine hydrochloride, formerly marketed as Pacatal tablets, and mepazine acetate, formerly marketed as Pacatal for injection, were associated with granulocytopenia, granulocytosis, paralytic ileus, urinary retention, seizures, hypotension, and jaundice. Approval of the NDA for Pacatal tablets and Pacatal for injection was withdrawn on May 28, 1970 (see the **Federal Register** of May 28, 1970 (35 FR 8405)).

Metabromsalan: All drug products containing metabromsalan. Metabromsalan, formerly marketed in a number of drug products, largely antibacterial soaps, as an antimicrobial, preservative, or for other purposes, was, with other halogenated salicylanilides listed in this proposal, found to be a potent photosensitizer capable of causing disabling skin disorders. FDA directed the removal from the market of drug products containing metabromsalan in 1975 (see § 310.508 (1997)).

Methamphetamine hydrochloride: All parenteral drug products containing methamphetamine hydrochloride. Parenteral methamphetamine hydrochloride, formerly marketed as Methedrine injection and Drinalfa injection and used as an adjunct treatment for weight reduction, was found to have a history of serious abuse and a severe risk of dependence. Approvals of the NDA's for Methedrine injection and Drinalfa injection were withdrawn on March 30, 1973 (see 21 CFR 310.504 (1997)).

Methapyrilene: All drug products containing methapyrilene. Methapyrilene, formerly marketed in many drug products, was shown to be a potent carcinogen. Manufacturers voluntarily withdrew methapyriline drug products from the market in May and June 1979.

Methopholine: All drug products containing methopholine. Methopholine, formerly marketed as Versidyne tablets, was associated with ophthalmic changes and corneal opacities in dogs. Approval of the NDA for Versidyne tablets was withdrawn on March 22, 1965 (see the **Federal Register** of March 27, 1965 (30 FR 4083)).

Mibefradil dihydrochloride: All drug products containing mibefradil dihydrochloride. Mibefradil dihydrochloride, formerly marketed as Posicor tablets, was associated with potentially harmful interactions with other drugs. Mibefradil dihydrochloride reduced the activity of certain liver enzymes that are important in helping the body eliminate many other drugs. Inhibiting these enzymes can cause some of these drugs to accumulate to dangerous levels in the body. The manufacturer voluntarily removed Posicor tablets from the market on June 8, 1998.

Neomycin sulfate: All parenteral drug products containing neomycin sulfate. Parenteral neomycin sulfate was found to present toxicity problems when used to irrigate wounds and was found not to be acceptable for the treatment of urinary tract infections due to the availability of newer, safer antibiotics that were as effective as, or more effective than, parenteral neomycin sulfate. Approvals of the marketing applications for parenteral neomycin sulfate were withdrawn on January 5, 1989 (see the **Federal Register** of December 6, 1988 (53 FR 49232)).

Nitrofurazone: All drug products containing nitrofurazone (except topical drug products formulated for dermatalogic application). Nitrofurazone, formerly marketed in nasal drops, otic drops, and vaginal suppositories, was associated with mammary neoplasia in rats. Approvals of the NDA's for the nitrofurazone drug products were withdrawn on December 4, 1974, and June 10, 1975 (see the **Federal Register** of December 4, 1974 (39 FR 42018), and May 30, 1975 (40 FR 23502)).

Nomifensine maleate: All drug products containing nomifensine maleate. Nomifensine maleate, formerly marketed as Merital capsules, was associated with an increased incidence of hemolytic anemia. The approved application holder removed Merital capsules from the market on January 23, 1986. FDA published a notice of its determination that Merital capsules were removed from the market for safety reasons (see the **Federal Register** of June 17, 1986 (51 FR 21981)). Approval of the NDA for Merital capsules was withdrawn on March 20, 1992 (see the **Federal Register** of March 20, 1992 (57 FR 9729)).

Oxyphenisatin: All drug products containing oxyphenisatin. Oxyphenisatin, formerly marketed in Lavema Compound Solution and Lavema Enema Powder, was associated with hepatitis and jaundice. The approvals of the NDA's for Lavema Compound Solution and Lavema Enema Powder were withdrawn on March 9, 1973 (see the **Federal Register** of March 9, 1973 (38 FR 6419)).

Oxyphenisatin acetate: All drug products containing oxyphenisatin acetate. Oxyphenisatin acetate, formerly marketed in Dialose Plus capsules, Noloc capsules, and other drug products, was associated with hepatitis and jaundice. Approvals of the NDA's for the oxyphenisatin acetate drug products were withdrawn on February 1, 1972 (see the **Federal Register** of February 1, 1972 (37 FR 2460)).

Phenacetin: All drug products containing phenacetin. Phenacetin, formerly marketed in A.P.C. with Butalbital tablets and capsules and other drug products, was associated with a high potential for harm to the kidneys and the possibility of hemolytic anemia and methemoglobinemia resulting from abuse. The approvals of the NDA's for the phenacetin drug products were withdrawn on November 4, 1983 (see the **Federal Register** of October 5, 1983 (48 FR 45466)).

Phenformin hydrochloride: All drug products containing phenformin hydrochloride. Phenformin hydrochloride, formerly marketed as D.B.I. tablets, Meltrol-50 capsules, and other drug products, was associated with lactic acidosis. Approvals of the NDA's for the phenformin hydrochloride drug products were withdrawn on November 15, 1978 (see the **Federal Register** of April 6, 1979 (44 FR 20967)).

Pipamazine: All drug products containing pipamazine. Pipamazine, formerly marketed as Mornidine tablets and injection, was associated with hepatic lesions. Approval of the NDA for Mornidine tablets and injection was withdrawn on July 17, 1969 (see the **Federal Register** of July 17, 1969 (34 FR 12051)).

Potassium arsenite: All drug products containing potassium arsenite. Potassium arsenite, formerly marketed as Fowler's Solution (oral), was toxic and highly carcinogenic. FDA determined Fowler's Solution was a new drug in April 1980, and the manufacturers removed the drug product from the market.

Potassium chloride: All solid oral dosage form drug products containing potassium chloride that supply 100 mg or more of potassium per dosage unit

(except for controlled-release dosage forms and those products formulated for preparation of solution prior to ingestion). Concentrated solid oral dosage forms of potassium salt were associated with small bowel lesions. Approvals of NDA's for all solid oral dosage form drug products containing potassium chloride that supply 100 mg or more of potassium per dosage unit (except for controlled-release dosage forms and those products formulated for preparation of solution prior to ingestion) were withdrawn on July 29, 1977, and April 29, 1992 (see the **Federal Register** of July 29, 1977 (42 FR 38644), and April 29, 1992 (57 FR 18157)).

Povidone: All intravenous drug products containing povidone. Povidone, marketed as Polyvinylpyrrolidone in Normal Saline, was found to be unsafe for use as a plasma expander in the emergency treatment of shock because povidone accumulates in the body and may cause storage disease with the formation of granulomas. Povidone also interferes with blood coagulation, hemostasis, and blood typing and cross matching. Approval of the NDA for Polyvinylpyrrolidone in Normal Saline was withdrawn on April 19, 1978 (see the **Federal Register** of April 7, 1978 (43 FR 14743)).

Reserpine: All oral dosage form drug products containing more than 1 mg of reserpine. Reserpine, marketed as Reserpoid tablets, Rau-Sed tablets, and other drug products for the treatment of hypertension and psychiatric disorders, was associated with a greater frequency and severity of adverse effects in strengths greater than 1 mg. Approvals of NDA's, or those portions of NDA's, for solid oral dosage form drug products containing more than 1 mg of reserpine were withdrawn on May 9, 1977 (see the **Federal Register** of April 29, 1977 (42 FR 21844)).

Sparteine sulfate: All drug products containing sparteine sulfate. Sparteine sulfate, formerly marketed as Spartocin injection and Tocosamine sterile solution, was found to have unpredicatable effects and was associated with tetanic uterine contractions and obstetrical complications. Approvals of the NDA's for Spartocin injection and Tocosamine sterile solution were withdrawn on August 17, 1979 (see the **Federal Register** of August 7, 1979 (44 FR 46316)).

Sulfadimethoxine: All drug products containing sulfadimethoxine. Sulfadimethoxine, formerly marketed in Madricidin capsules, was associated with Stevens-Johnson syndrome and fatalities. Approval of the NDA for Madricidin capsules was withdrawn on March 11, 1966 (see the **Federal Register** of March 19, 1966 (31 FR 4747)).

Sulfathiazole: All drug products containing sulfathiazole (except those formulated for vaginal use). Sulfathiazole, formerly marketed in Tresamide tablets and several other brands of tablets, was associated with renal complications, rash, fever, blood dyscrasias, and liver damage. Approvals of the NDA's for sulfathiazole tablets were withdrawn on September 28, 1970 (see the **Federal Register** of October 15, 1970 (35 FR 16190)).

Suprofen: All drug products containing suprofen (except ophthalmic solutions). Suprofen, formerly marketed as Suprol capsules, was associated with flank pain syndrome. The manufacturer voluntarily removed Suprol capsules from the market in May 1987.

Sweet spirits of nitre: All drug products containing sweet spirits of nitre. Sweet spirits of nitre, also known as spirit of nitre, spirit of nitrous ether, and ethyl nitrite spirit, was associated with methemoglobinemia in infants. FDA directed the removal from the market of drug products containing sweet spirits of nitre in 1980 (see 21 CFR 310.525 (1997)).

Temafloxacin hydrochloride: All drug products containing temafloxacin hydrochloride. Temafloxacin hydrochloride, formerly marketed as Omniflox tablets, was associated with hypoglycemia in elderly patients, as well as a constellation of multisystem organ involvement characterized by hemolytic anemia, frequently associated with renal failure, markedly abnormal liver tests, and coagulopathy. The approved application holder voluntarily removed Omniflox tablets from the market in Spring 1992. Approval of the NDA for Omniflox tablets was withdrawn on September 25, 1997 (see the **Federal Register** of September 25, 1997 (62 FR 50387)).

Terfenadine: All drug products containing terfenadine. Terfenadine, formerly marketed in Seldane and Seldane-D tablets, was associated with serious heart problems when used concurrently with certain drugs, including certain antibiotics and antifungals. Seldane and Seldane-D tablets were voluntarily removed from the market by their manufacturer in February 1998.

3,3′,4′,5-tetrachlorosalicylanilide: All drug products containing 3,3′,4′,5-tetrachlorosalicylanilide. The halogenated salicylanilide 3,3′,4′,5-tetrachlorosalicylanilide, formerly marketed in a number of drug products, largely antibacterial soaps, as an antimicrobial, preservative, or for other purposes, was, with other halogenated salicylanilides listed in this proposal, found to be a potent photosensitizer capable of causing disabling skin disorders. FDA directed the removal from the market of drug products containing 3,3′,4′,5-tetrachlorosalicylanilide in 1975 (see § 310.508 (1997)).

Tetracycline: All liquid oral drug products formulated for pediatric use containing tetracycline in a concentration greater than 25 mg/milliliter (mL). Concentrated tetracycline was associated with temporary inhibition of bone growth, permanent staining of the teeth, and enamel hypoplasia in children. FDA amended the antibiotic drug regulations so that drug products containing tetracycline formulated for pediatric use in a concentration greater than 25 mg/mL would not be certified (see the **Federal Register** of October 31, 1978 (43 FR 50676)).

Ticrynafen: All drug products containing ticrynafen. Ticrynafen, formerly marketed as Selacryn tablets, was associated with liver toxicity. Selacryn tablets were voluntarily withdrawn from the market by their manufacturer on January 16, 1980. Approval of the NDA for Selacryn tablets was withdrawn on May 20, 1996 (see the **Federal Register** of May 20, 1996 (61 FR 25228)).

Tribromsalan: All drug products containing tribromsalan. Tribromsalan, formerly marketed in a number of drug products, largely antibacterial soaps, as an antimicrobial, preservative, or for other purposes, was, with other halogenated salicylanilides listed in this proposal, found to be a potent photosensitizer capable of causing disabling skin disorders. FDA directed the removal from the market of drug products containing tribromsalan in 1975 (see § 310.508 (1997)).

Trichloroethane: All aerosol drug products intended for inhalation containing trichloroethane. Trichloroethane is potentially toxic to the cardiovascular system and was associated with deaths from misuse or abuse. FDA directed the removal from the market of aerosal drug products intended for inhalation containing trichloroethane in 1977 (see 21 CFR 310.507 (1997)).

Urethane: All drug products containing urethane. Urethane (also known as urethan and ethyl carbamate), formerly marketed as an inactive ingredient in Profenil injection, was determined to be carcinogenic. Approval of the NDA for Profenil

injection was withdrawn on March 28, 1977 (see the **Federal Register** of March 18, 1977 (42 FR 15138)).

Vinyl chloride: All aerosol drug products containing vinyl chloride. The inhalation of vinyl chloride is associated with acute toxicity manifested by dizziness, headache, disorientation, and unconsciousness. FDA directed the removal from the market of aerosol drug products containing vinyl chloride in 1974 (see 21 CFR 310.506 (1997)).

Zirconium: All aerosol drug products containing zirconium. Zirconium, formerly used in several aerosol drug products as an antiperspirant, was associated with human skin granulomas and toxic effects in the lungs and other internal organs of test animals. FDA directed the removal from the market of aerosol drug products containing zirconium in 1977 (see 21 CFR 310.510 (1997)).

Zomepirac sodium: All drug products containing zomepirac sodium. Zomepirac sodium, formerly marketed as Zomax tablets, was associated with fatal and near-fatal anaphylactoid reactions. The manufacturer voluntarily removed Zomax tablets from the market in March 1983.

IV. Environmental Impact

The agency has determined under 21 CFR 25.30(h) that this action is of a type that does not individually or cumulatively have a significant effect on the human environment. Therefore, neither an environmental assessment nor an environmental impact statement is required.

V. Analysis of Impacts

FDA has examined the impacts of the proposed rule under Executive Order 12866, the Regulatory Flexibility Act (5 U.S.C 601–612), and the Unfunded Mandates Reform Act of 1995 (Pub. L. 104–4). Executive Order 12866 directs agencies to assess all costs and benefits of available regulatory alternatives and, when regulation is necessary, to select regulatory approaches that maximize net benefits (including potential economic, environmental, public health and safety, and other advantages; distributive impacts; and equity). Executive Order 12866 classifies a rule as significant if it meets any one of a number of specified conditions, including having an annual effect on the economy of $100 million or adversely affecting in a material way a sector of the economy, competition, or jobs, or if it raises novel legal or policy issues. As discussed in the following paragraphs, the agency believes that this proposed rule is consistent with the regulatory philosophy and principles identified in the Executive Order. In addition, the proposed rule is not a significant regulatory action as defined by the Executive Order and so is not subject to review under the Executive Order.

The agency has not estimated any compliance costs or loss of sales due to this proposal because it prohibits pharmacy compounding of only those drug products that have already been withdrawn or removed from the market. Although the agency is not aware of any routine use of these drug products in pharmacy compounding, the agency invites the submission of comments on this issue and solicits current compounding usage data for these drug products.

Unless an agency certifies that a rule will not have a significant economic impact on a substantial number of small entities, the Regulatory Flexibility Act requires agencies to analyze regulatory options to minimize any significant economic impact of a regulation on small entities. The agency is taking this action in order to comply with Section 503A of the act. This provision specifically directs FDA to develop a list of drug products that have been withdrawn or removed from the market because such products or components have been found to be unsafe or not effective. Any drug product on this list will not qualify for the pharmacy compounding exemptions under section 503A of the act. The drug products on this list were manufactured by many different pharmaceutical firms, some of which may have qualified under the Small Business Administration (SBA) regulations (those with less than 750 employees) as small businesses. However, since the list only includes those drug products that have already been withdrawn or removed from the market for safety or efficacy concerns, this proposal will not negatively impact these small businesses. Moreover, no compliance costs are estimated for any of these small pharmaceutical firms because they are not the subject of this rule and are not expected to realize any further loss of sales due to this proposal. Further, the SBA guidelines limit the definition of small drug stores or pharmacies to those that have less than $5.0 million in sales. Again, the pharmacies that qualify as small businesses are not expected to incur any compliance costs or loss of sales due to this regulation because the products have already been withdrawn or removed from the market, and the agency believes that these drugs would be compounded only very rarely, if ever. Therefore, FDA certifies that this rule will not have a significant economic impact on a substantial number of small entities.

The Unfunded Mandates Reform Act requires (in section 202) that agencies prepare an assessment of anticipated costs and benefits before proposing any expenditure by State, local, and tribal Governments, in the aggregate, or by the private sector of $100 million (adjusted annually for inflation) in any 1 year. The publication of the list of products withdrawn or removed from the market because they were found to be unsafe or ineffective will not result in expenditures of funds by State, local, and tribal governments or the private sector in excess of $100 million annually. Because the agency does not estimate any annual expenditures due to the proposed rule, FDA is not required to perform a cost/benefit analysis according to the Unfunded Mandates Reform Act.

VI. Paperwork Reduction Act of 1995

FDA tentatively concludes that this proposed rule contains no collections of information. Therefore, clearance by the Office of Management and Budget under the Paperwork Reduction Act of 1995 (Pub. L. 104–13) is not required.

VII. Request for Comments

Interested persons may, on or before November 23, 1998, submit to the Dockets Management Branch (address above) written comments regarding this proposal. Two copies of any comments are to be submitted, except that individuals may submit one copy. Comments are to be identified with the docket number found in brackets in the heading of this document. Received comments may be seen in the office above between 9 a.m. and 4 p.m., Monday through Friday.

The agency notes that the comment period in this document is shorter than the 75-day period that is customarily provided by FDA for proposed rules of a technical nature. Likewise, this comment period is less than the 60 days ordinarily provided, as set out in FDA's procedural regulations, § 10.40(b)(2) (21 CFR 10.40(b)(2)). As discussed in the following paragraphs, FDA believes that a 45-day comment period is appropriate in this instance. Executive Order 12889 (58 FR 69681, December 30, 1993), which implemented the North American Free Trade Agreement, states that any agency subject to the Administrative Procedure Act should provide a 75-day comment period for any proposed Federal technical regulation or any Federal sanitary or phytosanitary measure of general application. However, Executive Order 12889 provides an exception to the 75-

day period where the United States considers the measure necessary to address an urgent problem related to the protection of human, plant, or animal health. Similarly, FDA regulations establish a 60-day comment period as ordinary agency practice, but provide that the 60-day period may be shortened if the Commissioner of Food and Drugs finds good cause for doing so.

As discussed in this document, section 503A(a) of the act exempts certain compounded drug products from some specific misbranding and adulteration provisions, as well as the new drug provision, of the act. Section 503A(b)(1)(C) of the act excludes from the exemption drugs that FDA has found were removed from the market or had marketing applications withdrawn because the drug product or some component of the drug product was unsafe or ineffective. Compounding versions of many of these drug products presents a serious risk to human health, either indirectly, because a patient is being provided an ineffective drug product when effective drug products may be available, or directly, due to the toxicity of the drug product. Indeed, many of the drug products listed in this proposed rule have been associated with human fatalities.

Section 127(b) of the Modernization Act provides that section 503A of the act will go into effect on November 21, 1998. If a final regulation issuing the list of drug products that have been withdrawn or removed is not published before November 21, 1998, these drug products may be compounded, exempt from various legal requirements, contrary to the expressed intent of Congress and at a risk to human health. Accordingly, the agency intends to solicit public comment on this proposal, consider the comments submitted, and prepare and publish a final implementing regulation by November 21, 1998. FDA has concluded that the urgency of this matter is sufficient justification for shortening the comment period for this proposal to 45 days, consistent with Executive Order 12889. Similarly, this urgency constitutes good cause within the meaning of § 10.40(b), which justifies shortening the period to 45 days.

List of Subjects in 21 CFR Part 216

Drugs, Pharmacy compounding, Prescription drugs.

Therefore, under the Federal Food, Drug, and Cosmetic Act and under authority delegated to the Commissioner of Food and Drugs, it is proposed that 21 CFR part 216 be added to read as follows:

1. Part 216 is added to read as follows:

PART 216—PHARMACY COMPOUNDING

Subpart A—General Provisions [Reserved]

Subpart B—Compounded Drug Products

Sec.
216.23 [Reserved]
216.24 Drug products withdrawn or removed from the market for reasons of safety or effectiveness.

Authority: 21 U.S.C. 351, 352, 353a, 355, and 371.

Subpart A—General Provisions [Reserved]

Subpart B—Compounded Drug Products

§ 216.23 [Reserved]

§ 216.24 Drug products withdrawn or removed from the market for reasons of safety or effectiveness.

The following drug products were withdrawn or removed from the market because such drug products or components of such drug products were found to be unsafe or not effective. The following drug products may not be compounded under the exemptions provided by section 503A(a) of the Federal Food, Drug, and Cosmetic Act:

Adenosine phosphate: All drug products containing adenosine phosphate.

Adrenal cortex: All drug products containing adrenal cortex.

Azaribine: All drug products containing azaribine.

Benoxaprofen: All drug products containing benoxaprofen.

Bithionol: All drug products containing bithionol.

Bromfenac sodium: All drug products containing bromfenac sodium.

Butamben: All parenteral drug products containing butamben.

Camphorated oil: All drug products containing camphorated oil.

Carbetapentane citrate: All oral gel drug products containing carbetapentane citrate.

Casein, iodinated: All drug products containing iodinated casein.

Chlorhexidine gluconate: All tinctures of chlorhexidine gluconate formulated for use as a patient preoperative skin preparation.

Chlormadinone acetate: All drug products containing chlormadinone acetate.

Chloroform: All drug products containing chloroform.

Cobalt: All drug products containing cobalt salts (except radioactive forms of cobalt and its salts and cobalamin and its derivatives).

Dexfenfluramine hydrochloride: All drug products containing dexfenfluramine hydrochloride.

Diamthazole dihydrochloride: All drug products containing diamthazole dihydrochloride.

Dibromsalan: All drug products containing dibromsalan.

Diethylstilbestrol: All oral and parenteral drug products containing 25 milligrams or more of diethylstilbestrol per unit dose.

Dihydrostreptomycin sulfate: All drug products containing dihydrostreptomycin sulfate.

Dipyrone: All drug products containing dipyrone.

Encainide hydrochloride: All drug products containing encainide hydrochloride.

Fenfluramine hydrochloride: All drug products containing fenfluramine hydrochloride.

Flosequinan: All drug products containing flosequinan.

Gelatin: All intravenous drug products containing gelatin.

Glycerol, iodinated: All drug products containing iodinated glycerol.

Gonadotropin, chorionic: All drug products containing chorionic gonadotropins of animal origin.

Mepazine: All drug products containing mepazine hydrochloride or mepazine acetate.

Metabromsalan: All drug products containing metabromsalan.

Methamphetamine hydrochloride: All parenteral drug products containing methamphetamine hydrochloride.

Methapyrilene: All drug products containing methapyrilene.

Methopholine: All drug products containing methopholine.

Mibefradil dihydrochloride: All drug products containing mibefradil dihydrochloride.

Neomycin sulfate: All parenteral drug products containing neomycin sulfate.

Nitrofurazone: All drug products containing nitrofurazone (except topical drug products formulated for dermatalogic application).

Nomifensine maleate: All drug products containing nomifensine maleate.

Oxyphenisatin: All drug products containing oxyphenisatin.

Oxyphenisatin acetate: All drug products containing oxyphenisatin acetate.

Phenacetin: All drug products containing phenacetin.

Phenformin hydrochloride: All drug products containing phenformin hydrochloride.

Pipamazine: All drug products containing pipamazine.

Potassium arsenite: All drug products containing potassium arsenite.

Potassium chloride: All solid oral dosage form drug products containing potassium chloride that supply 100 milligrams or more of potassium per dosage unit (except for controlled-release dosage forms and those products formulated for preparation of solution prior to ingestion).

Povidone: All intravenous drug products containing povidone.

Reserpine: All oral dosage form drug products containing more than 1 milligram of reserpine.

Sparteine sulfate: All drug products containing sparteine sulfate.

Sulfadimethoxine: All drug products containing sulfadimethoxine.

Sulfathiazole: All drug products containing sulfathiazole (except those formulated for vaginal use).

Suprofen: All drug products containing suprofen (except ophthalmic solutions).

Sweet spirits of nitre: All drug products containing sweet spirits of nitre.

Temafloxacin hydrochloride: All drug products containing temafloxacin hydrochloride.

Terfenadine: All drug products containing terfenadine.

3,3′,4′,5-tetrachlorosalicylanilide: All drug products containing 3,3′,4′,5-tetrachlorosalicylanilide.

Tetracycline: All liquid oral drug products formulated for pediatric use containing tetracycline in a concentration greater than 25 milligrams/milliliter.

Ticrynafen: All drug products containing ticrynafen.

Tribromsalan: All drug products containing tribromsalan.

Trichloroethane: All aerosol drug products intended for inhalation containing trichloroethane.

Urethane: All drug products containing urethane.

Vinyl chloride: All aerosol drug products containing vinyl chloride.

Zirconium: All aerosol drug products containing zirconium.

Zomepirac sodium: All drug products containing zomepirac sodium.

Dated: October 1, 1998.

William B. Schultz,

Deputy Commissioner for Policy.

[FR Doc. 98–26923 Filed 10–2–98; 4:25 pm]

BILLING CODE 4160–01–F

ENVIRONMENTAL PROTECTION AGENCY

40 CFR Part 52

[PA–4076b; FRL–6166–2]

Approval and Promulgation of Air Quality Implementation Plans; Pennsylvania; Approval of VOC and NO_X RACT Determinations for Individual Sources

AGENCY: Environmental Protection Agency (EPA).

ACTION: Proposed rule.

SUMMARY: EPA proposes to approve the State Implementation Plan (SIP) revision submitted by the Commonwealth of Pennsylvania for the purpose of establishing volatile organic compound (VOC) and nitrogen oxides (NO_X) reasonably available control technology (RACT) for four (4) major sources located in Pennsylvania. In the Final Rules section of this **Federal Register**, EPA is approving the Commonwealth's SIP revision as a direct final rule without prior proposal because the Agency views this as a noncontroversial SIP revision and anticipates no adverse comments. A detailed rationale for the approval is set forth in the direct final rule and the accompanying technical support document. If no adverse comments are received in response to this rule, no further activity is contemplated in relation to this rule. If EPA receives adverse comments, the direct final rule will be withdrawn and all public comments received will be addressed in a subsequent final rule based on this proposed rule. EPA will not institute a second comment period on this action. Any parties interested in commenting on this action should do so at this time. If adverse comments are received that do not pertain to all documents subject to this rulemaking action, those documents not affected by the adverse comments will be finalized in the manner described here. Only those documents that receive adverse comments will be withdrawn in the manner described here.

DATES: Comments must be received in writing by November 9, 1998.

ADDRESSES: Written comments should be addressed to David Campbell, Air Protection Division, Mailcode 3AP11, U.S. Environmental Protection Agency, Region III, 1650 Arch St., Philadelphia, Pennsylvania 19103. Copies of the documents relevant to this action are available for public inspection during normal business hours at the Air Protection Division, U.S. Environmental Protection Agency, Region III, 1650 Arch St., Philadelphia, Pennsylvania 19103; and the Pennsylvania Department of Environmental Protection, Bureau of Air Quality Control, P.O. Box 8468, 400 Market Street, Harrisburg, Pennsylvania 17105.

FOR FURTHER INFORMATION CONTACT: David Campbell, (215) 814–2196, at the EPA Region III office or via e-mail at campbell.dave@epamail.epa.gov. While information may be requested via e-mail, comments must be submitted in writing to the above Region III address.

SUPPLEMENTARY INFORMATION: See the information pertaining to this action, VOC and NO_X RACT determinations for individual sources located in Pennsylvania, provided in the Direct Final action of the same title which is located in the Rules and Regulations Section of this **Federal Register**.

Authority: 42 U.S.C. 7401–7671q.

Dated: September 11, 1998.

W. Michael McCabe,

Regional Administrator, Region III.

[FR Doc. 98–26896 Filed 10–7–98; 8:45 am]

BILLING CODE 6560–50–P

ENVIRONMENTAL PROTECTION AGENCY

40 CFR Part 52

[TN–201–9828b; FRL–6169–7]

Approval and Promulgation of Implementation Plans Tennessee: Approval of Revisions to the Nashville/Davidson County Portion of the Tennessee SIP Regarding Control of Volatile Organic Compounds

AGENCY: Environmental Protection Agency (EPA).

ACTION: Proposed rule.

SUMMARY: The EPA proposes to approve revisions to the Nashville/Davidson County portion of the Tennessee State Implementation Plan (SIP) concerning control of volatile organic compounds. The State of Tennessee through the Tennessee Department of Air Pollution Control submitted the revisions to EPA on July 23, 1997. To be consistent with the EPA's Guidelines for ''Control of Volatile Organic Compounds Emissions from Stationary Sources,'' the State of Tennessee amended Regulation No. 7, ''Regulation for Control of Volatile Organic Compounds, Section 7–16, Emission Standards for Surface Coating of Miscellaneous Metal Parts and Products'' of the Nashville/Davidson County portion of the Tennessee SIP (Nashville SIP).

In the final rules section of this **Federal Register**, the EPA is approving the State of Tennessee SIP revision as a direct final rule without prior proposal because the Agency views this as a noncontroversial revision amendment and anticipates no adverse comments. A detailed rationale for the approval is set forth in the direct final rule. If no adverse comments are received in response to the direct final rule, no further activity is contemplated in relation to this proposed rule. If EPA receives adverse comments, the direct final rule will be withdrawn and all public comments received will be addressed in a subsequent final rule based on this proposed rule. The EPA will not institute a second comment period on this document. Any parties interested in commenting on this document should do so at this time.

DATES: To be considered, comments must be received by November 9, 1998.

ADDRESSES: Written comments should be addressed to Mr. Gregory O. Crawford at the EPA Regional Office listed below. Copies of documents relative to this action are available for public inspection during normal business hours at the following locations. The interested persons
