# Supplementary material for: Starch Wars—New Episodes of the Saga. Changes in Regulations on Hydroxyethyl Starch in the European Union
Source: Front Vet Sci. 2019 Jan 18;5:336. doi: 10.3389/fvets.2018.00336 (PMC6345713; doi:10.3389/fvets.2018.00336)
Supplement: Supplementary file 3 [file Data_Sheet_3.PDF]

Appendix 3 – Available online sources for the HES restrictions 2018 used in the article

Index

1. hydroxyethyl-starch-article-107i-referral-notification_Sweden
2. 01/12/2018: PRAC recommends suspending hydroxyethyl-starch solutions for infusion from the market
3. 01/26/2018: hydroxyethyl-starch-article-107i-referral-cmdh-endorses-prac-recommendation_en
4. HES-Open-Letter_EC DGAI 2018
5. Fresenius Kabi Website 01_29_2018
6. B.Braun Germany Website 01_26_2018
7. European Comission meeting 04_2018
8. Meeting highlights PRAC 14-17 May 2018
9. 05/17/2018: hydroxyethyl-starch-article-107i-referral-assessment-report_en
10. 06/29/2018: hydroxyethyl-starch-solutions-cmdh-introduces-new-measures-protect-patients_en
11. hydroxyethyl-starch-article-107i-referral-annex-ii_en

LÄKEMEDELSVERKET
MEDICAL PRODUCTS AGENCY

NOTIFICATION TO THE PRAC/EMA SECRETARIAT OF A REFERRAL UNDER ARTICLE 107i OF DIRECTIVE 2001/83/EC
E-mail: ReferralNotifications@ema.europa.eu

This notification is a referral under Article 107i of Directive 2001/83/EC to the PRAC made by Sweden:

Product Name(s) in the Referring Member State, if applicable	**Voluven 60 mg/ml, DE/H/223/01**<br>**Volulyte 60 mg/ml, DE/H/619/01**<br>**Voluven 100 mg/ml, DE/H/1568/01**<br>**Tetraspan 60 mg/ml, SE/H/609/01**<br>**Tetraspan 100 mg/ml, SE/H/609/02**<br>**Venofundin 60 mg/ml, SE/H/414/01**<br>**Hesra, DE/H/1210/01**<br><br>Hydroxyethyl starch (HES) containing medicinal products and related substances
Active substance(s)	Hydroxyethyl starch
Pharmaceutical form(s)	Solution for infusion
Strength(s)	All
Route(s) of Administration	Parenteral
Marketing Authorisation Holder(s) in the referring Member State	Fresenius Kabi AB<br>B.Braun Melsungen AG<br>Serumwerk Bernburg AG

Background

Hydroxyethyl starch (HES) solutions for infusion include products with starch with different molecular weights (mainly 130kD; 200kD) and substitution ratios (the number of hydroxyethyl groups per glucose molecule). HES solutions for infusion are authorised worldwide with the main indication for the treatment of hypovolaemia.

In 2012 and 2013, the Pharmacovigilance and Risk Assessment Committee (PRAC) reviewed the benefits and risks of HES-containing medicinal products for infusion in the treatment and prophylaxis of hypovolaemia, within Article 31[1] and 107i[2] referral procedures. These reviews were triggered by the results from large randomised clinical studies[3,4,5] which showed an increased risk of mortality in patients with sepsis and an increased risk of kidney injury requiring dialysis in critically ill patients following treatment with HES solutions for infusion.

In October 2013, the PRAC finalised its review as follows. The PRAC considered that the use of HES is associated with an increased risk of mortality and renal replacement therapy or

1 http://www.ema.europa.eu/ema/index.jsp?curl=pages/medicines/human/referrals/Hydroxyethyl_starch-containing_solutions/human_referral_prac_000012.jsp&mid=WC0b01ac05805c516f

2 http://www.ema.europa.eu/ema/index.jsp?curl=pages/medicines/human/referrals/Hydroxyethyl_starch-containing_medicines/human_referral_prac_000029.jsp&mid=WC0b01ac05805c516f

3 Perner A, Haase N, Guttormsen AB et al. Hydroxyethyl starch 130/0.42 versus ringer's acetate in severe sepsis. N Engl J Med 2012;367(2):124-34

4 Brunkhorst FM, Engel C, Bloos F et al. Intensive Insulin Therapy and Pentastarch Resuscitation in Severe Sepsis. N Engl J Med 2008; 358(2):125-39

5 Myburgh J, Finder S, Bellomo R *et al.* Hydroxyethyl starch or saline for fluid resuscitation in intensive care. *N Engl J Med* 2012; 367:1901-11

renal impairment in patients with sepsis, critically ill and burn patients. Furthcr, that thc benefit of HES containing medicinal products outweighs the risk in the treatment of hypovolaemia due to acute blood loss when crystalloids alone are not considered sufficient. The PRAC also concluded that HES containing medicinal products should be contraindicated in patients with sepsis, in critically ill and burn patients.

As conditions to the marketing authorisations, the PRAC requested that further studies be carried out on the use of these medicines in elective surgery and trauma patients, as well as to study drug utilisation to evaluate the effectiveness of the risk minimisation measures taken. Focus for the drug utilisation studies has been to evaluate the adherence to the restricted indications and new contraindications, which were implemented in the product information.

On 5th July 2017 and 9th October 2017, results from two drug utilisation studies on the effectiveness of the risk minimisation measures implemented have become available. These include utilisation data from in total 10 EU Member States. From these studies, it is evident that the implemented restrictions in use are not adhered to. Non-adherence to the revised product information was reported to range from 67 % - 77 %, including 20 – 34 % non-adherence to contraindications. On average, across all EU Member States included in the study, 9 % of patients exposed to HES solutions for infusion were critically ill, 5-8% of patients had renal impairment and 3-4 % of patients had sepsis. However, it should be noted that there was considerable variability in adherence, and thus in some EU Members States, these proportions were considerably higher.

These data raise serious concerns as use of HES solutions for infusion in patient populations which are contraindicated such as those who are critically ill, in patients with renal impairment, or with sepsis, is associated with a scientifically well-established risk for serious harm including mortality[3, 4, 5]. Recent estimations of patient exposure across the EU indicate approximately 750 000 – 1.5 million patients exposed to HES solutions yearly.

In light of scientifically well-established risk for serious harm including mortality when HES solutions for infusion are used in contraindicated populations, together with these newly available data, Sweden seriously questions whether the benefit/risk balance of these medicinal products remains favourable. Consequently in view of the serious public health impact, Sweden considers suspending the marketing authorisations for the above mentioned products, and requests an urgent review of the matter at the European level.

In view of the above, Sweden initiates an urgent union procedure under Article 107i of Directive 2001/83/EC and refers the matter to the PRAC which is requested to give its recommendation as to whether marketing authorisations of these products should be maintained, varied, suspended, or revoked.

As the request results from the evaluation of data resulting from pharmacovigilance activities, the position should be adopted by the CMDh on the basis of a recommendation of the PRAC.

Signed [redacted] Date 2017-10-17

Marie Gårdmark
Direktör VO Tillstånd
Läkemedelsverket

Director Licensing

EUROPEAN MEDICINES AGENCY
SCIENCE MEDICINES HEALTH

12 January 2018
EMA/4068/2018

PRAC recommends suspending hydroxyethyl-starch solutions for infusion from the market

Review finds measures to protect patients have not been sufficiently effective

EMA's Pharmacovigilance Risk Assessment Committee (PRAC) has recommended the suspension of the marketing authorisations for hydroxyethyl-starch (HES) solutions for infusion across the European Union. These products are used as plasma volume replacement following acute (sudden) blood loss, where treatment with alternative products known as 'crystalloids' alone is not considered to be sufficient.

The review was triggered by results from two drug utilisation studies indicating that HES solutions are being used in critically ill patients and those with sepsis and kidney injury despite restrictions introduced in 2013 to reduce the risks of kidney problems and deaths in these patient populations.

In 2013, the PRAC had recommended restrictions on the use of HES solutions, including that they must no longer be used to treat critically ill patients or patients with sepsis, because of an increased risk of kidney injury and mortality seen in clinical trials. The Committee requested that further studies be carried out to verify adherence to these restrictions.

The PRAC has reviewed the results from the drug utilisation studies of HES solutions for infusion together with the currently available data on benefits and risks from clinical trials and observational studies and feedback received from stakeholders and experts. Based on this review, the PRAC has concluded that the restrictions introduced in 2013 have not been sufficiently effective. The Committee explored the possibility of introducing additional measures but concluded that such measures would be ineffective or insufficient.

In view of the serious risks that certain patient populations are exposed to, the PRAC has recommended the suspension of the marketing authorisations for HES solutions. Alternative treatment options are available.

The PRAC recommendation will now be sent to the Coordination Group for Mutual Recognition and Decentralised Procedures – Human (CMDh)[1] for consideration at its meeting on 22-25 January 2018.

[1] The CMDh is a medicines regulatory body representing the European Union (EU) Member States, Iceland, Liechtenstein and Norway.

30 Churchill Place ● Canary Wharf ● London E14 5EU ● United Kingdom
Telephone +44 (0)20 3660 6000 **Facsimile** +44 (0)20 3660 5555
Send a question via our website www.ema.europa.eu/contact

An agency of the European Union

© European Medicines Agency, 2018. Reproduction is authorised provided the source is acknowledged.

More about the medicines

HES solutions for infusion are used for the management of hypovolaemia (low blood volume) caused by acute blood loss, where treatment with alternative infusion solutions known as ‘crystalloids’ alone is not considered to be sufficient. They are given by infusion (drip) into a vein and are used as blood volume expanders to prevent shock following acute bleeding. They belong to the class of medicines known as colloids. Besides blood products, there are two types of medicines used for plasma volume replacement: crystalloids and colloids. Colloids contain large molecules such as starch, whereas crystalloids, such as saline or Ringer’s solutions, are pure electrolyte solutions.

In the European Union, HES solutions for infusion have been approved via national procedures and are available in the Member States under various trade names.

More about the procedure

The review of HES solutions for infusion was initiated on 17 October 2017 at the request of the Swedish Medical Products Agency, under Article 107i of Directive 2001/83/EC.

The review has been carried out by the Pharmacovigilance Risk Assessment Committee (PRAC), the Committee responsible for the evaluation of safety issues for human medicines, which has made a set of recommendations. The PRAC recommendations will now be sent to the Co-ordination Group for Mutual Recognition and Decentralised Procedures – Human (CMDh), which will adopt a position. The CMDh is a body representing EU Member States as well as Iceland, Liechtenstein and Norway. It is responsible for ensuring harmonised safety standards for medicines authorised via national procedures across the EU.

26 January 2018
EMA/35795/2018 corr. 1

Hydroxyethyl-starch solutions for infusion to be suspended – CMDh endorses PRAC recommendation

Suspension due to serious risks of kidney injury and death in certain patient populations

The CMDh[1] has endorsed the recommendation to suspend the marketing authorisations of hydroxyethyl-starch (HES) solutions for infusion across the European Union. These products are used as plasma volume replacement following acute (sudden) blood loss, where treatment with alternative products known as 'crystalloids' alone is not considered to be sufficient.

The review of HES solutions for infusion has been carried out by EMA's Pharmacovigilance Risk Assessment Committee (PRAC). The CMDh endorsed the suspension recommended by the PRAC due to the fact that these medicines have continued to be used in critically ill patients and patients with sepsis despite restrictions on use in these patient populations introduced in 2013 to reduce the risk of kidney injury and death. The final decision, however, will be taken by the European Commission.[2]

The PRAC reviewed results of drug utilisation studies, together with the currently available data on benefits and risks from clinical trials and observational studies and feedback received from stakeholders and experts. Based on this review, the PRAC concluded that the restrictions introduced in 2013 have not been sufficiently effective.

The PRAC also explored the possibility of introducing additional measures to protect patients at risk but concluded that such measures would be ineffective or insufficient.

The CMDh has now agreed with the PRAC recommendation that, in view of the serious risks that certain patients are exposed to, HES solutions for infusion should be suspended. Alternative treatment options are available.

As the CMDh position was adopted by majority vote, the CMDh position will now be sent to the European Commission, which will take an EU-wide legally binding decision.

Information for patients

- HES solutions for infusion are replacement fluids given to patients who have lost blood following injury or surgery.

[1] The CMDh is a medicines regulatory body representing the European Union (EU) Member States, Iceland, Liechtenstein and Norway.
[2] Text modified to clarify next procedural steps. Changes made on 2 February 2018.

30 Churchill Place ● Canary Wharf ● London E14 5EU ● United Kingdom
Telephone +44 (0)20 3660 6000 **Facsimile** +44 (0)20 3660 5555
Send a question via our website www.ema.europa.eu/contact

© European Medicines Agency, 2018. Reproduction is authorised provided the source is acknowledged.

- These products are being suspended in the EU in view of the serious risks that certain patients (for example those who are very ill or have blood poisoning) are exposed to.
- Other treatment options are available for treating blood loss in the EU.
- This suspension does not apply to the use of HES in clinical trials, where patient selection is tightly controlled. Member States where trials with HES solutions for infusion are ongoing may review the way these trials are conducted in light of the outcome of this review.
- If you are in a clinical trial with HES solutions for infusion and have any questions or concerns about these products speak to the doctor who is giving it to you.

Information for healthcare professionals

- The marketing authorisations of HES solutions for infusion are being suspended in the EU because of the risk of kidney injury and death in certain patient populations, including critically ill patients or patients with sepsis. Despite contraindications introduced in 2013, drug utilisation studies show that HES solutions for infusion have continued to be used in these patients.
- Experience in clinical practice suggests that it is difficult to clearly distinguish patients who can be administered HES solutions for infusion from those who should not. In addition, some patients may become critically ill or septic while receiving the product.
- As further measures to minimise the risks are unlikely to be sufficiently effective, HES solutions for infusion are being suspended to protect patient health.
- Alternative therapeutic options are available for routine clinical practice (including albumin, gelatins and dextrans) and should be selected according to relevant clinical guidelines.
- The suspension concerns use of HES solutions for infusion in routine clinical practice, where it was found that the medicine was used in contraindicated populations; it does not apply to their use in clinical trials, where patient selection is tightly controlled. Member States where trials with HES solutions for infusion are ongoing may review the way these trials are conducted in light of the outcome of this review.
- Several clinical trials with HES solutions for infusion are ongoing; these include two studies previously requested by the PRAC in patients with trauma and in elective surgery, which is the population for which the products are currently indicated.
- For the suspension to be lifted, the marketing authorisation holders should provide reliable and convincing evidence on a favourable benefit-risk balance in a well-defined population, with feasible and effective measures to adequately minimise exposure of patients at an increased risk of serious harm.
- Healthcare professionals will be informed in writing of the outcome of the review and the suspension of the marketing authorisations of HES solutions for infusion.

More about the medicine

HES solutions for infusion are used for the management of hypovolaemia (low blood volume) caused by acute blood loss, where treatment with alternative infusion solutions known as 'crystalloids' alone is

not considered to be sufficient. They are given by infusion (drip) into a vein and are used as blood volume expanders to prevent shock following acute bleeding. They belong to the class of medicines known as colloids. Besides blood products, there are two types of medicines used for plasma volume replacement: crystalloids and colloids. Colloids contain large molecules such as starch, whereas crystalloids, such as saline or Ringer's solutions, are pure electrolyte solutions.

In the European Union, HES solutions for infusion have been approved via national procedures and are available in the Member States under various trade names.

More about the procedure

The review of HES solutions for infusion was initiated on 17 October 2017 at the request of the Swedish Medical Products Agency, under Article 107i of Directive 2001/83/EC.

The review was first conducted by the Pharmacovigilance Risk Assessment Committee (PRAC). The PRAC recommendation was sent to Co-ordination Group for Mutual Recognition and Decentralised Procedures – Human (CMDh), which adopted a final position. The CMDh, a body representing EU Member States, is responsible for ensuring harmonised safety standards for medicines authorised via national procedures across the EU.

As the CMDh position was adopted by majority vote, the CMDh position will now be sent to the European Commission, which will take an EU-wide legally binding decision.

Tell us how well we did with this information

Please will you tell us what you think about this information by answering a few questions — it will take up only a couple of minutes of your time.

Start the survey

DGAI Präsident ◆ Direktor der Klinik für Anästhesiologie
Klinikum der Universität München ◆ Marchioninistr. 15
81377 München

PRÄSIDENT

Professor Dr. med. Bernhard Zwißler

Telefon: +49 / 89 / 4400 - 74550
Telefax: +49 / 89 / 4400 - 78885
E-Mail: direktion.anaesthesie@med.lmu.de

European Commission
To His Excellency Mr Vytenis Andriukaitis
EU Commissioner for Health and Food safety
Rue de la Loi / Wetstraat 200
1049 Brussels
Belgium

By email: cab-andriukaitis-webpage@ec.europa.eu

16th of March 2018

Open letter to the European Commission:

Marketing Authorization of colloid solutions containing hydroxyethyl starch (HES)

Ref: Studies HC-G-H-1504 and HC-G-H-1505 Information on Health Authority Activities for HES Products

Dear Mr. Commissioner,

On behalf 19 European Societies of Anesthesiology*****, we would like to express with this open letter our great concern about the current pharmacovigilance procedure for the volume replacement agent hydroxyethyl starch (HES) and ask you to stand up for obtaining marketing authorization from HES. At the same time, we agree with the statements of the Board of Directors of the European Society of Anaesthesiology (ESA) made in their letter from 5th of March 2018.

In 2013, the Referral Procedure concerning the use of hydroxyethyl starch (HES)-containing solutions by the European Medicines Agency (EMA) resulted in changes of the product information such as reduction of the maximum dose (30 ml/kg) and duration of treatment (up to 24 hours) as well as new contraindications including sepsis and critically ill patients. HES is currently indicated for surgical and trauma patients with hypovolaemia due to acute blood loss when crystalloids alone are not considered sufficient.
Since 2013, all accessible data derived from clinical trials have confirmed the safety and efficacy of HES in these patient populations. International high quality guidelines reflect the

current knowledge and consider HES equally safe as crystalloids, because there is no sign of increased mortality or renal insufficiency.[1] Therefore, we were very surprised that the EMA initiated an urgent union procedure according to Article 107i and recommended that HES solutions should be completely suspended in 2017. In the explanatory statement, the EMA bases this recommendation on two imposed retrospective drug utilisation studies (DUS) by two pharmaceutical companies that used the same questionnaires. The retrospective and anonymised documentation is based on over 6000 patient charts that did not reveal any adverse safety signal. The DUS showed that the adherence to the new dose and duration been adhered to by almost 100%.

However, hospital physicians apparently documented a high degree of non-adherence to the revised Summary of Product Characteristics (SmPC), especially the use of HES in dehydrated patients. With regard to the fluid status, it is problematic that the main indication for HES (intravascular hypovolemia) was not provided as an option (tick box) in the electronic patient report form (eCRF), but just dehydration and hyperhydration that both represent contraindications. Since most physicians chose the least wrong answer (i.e. dehydration), the results concerning contraindications are artificially inflated. This is further illustrated by the fact that patients may suffer from both dehydration and hypovolemia. If a patient was documented as having received HES and being classified as having had some degree of dehydration during the hospital stay, it does not imply at all that HES was actually infused to treat dehydration. Due to the limited amount of documented data and the imposed design of the study, a validation of the results is not possible and poses more questions than it gives answers.
In addition, it was documented in the DUS that some of the hospitalised patients receiving HES had also sepsis during their hospital stay. However, it is important to note that the DUS only asked, if HES infusion was “timely related to sepsis”. Every clinician knows that sepsis can occur after major abdominal surgery. The exact time point of diagnosis is difficult or practically impossible to be determined in retrospective trials and is certainly independent of the use of HES.

A post-hoc analysis of 460 patients initially included in the DUS in 5 centres with high non-adherence rates revealed that among patients with an initially documented contraindication, only 8 patients actually had one; none suffered from acute sepsis at the time of HES administration. All these aspects were supported and criticised by the independent ad-hoc expert committee appointed by the EMA.[2] In addition, these experts emphasised that there definitely is a role for HES in the licensed indication and recommended maintaining the marketing authorisation, especially since the benefit/risk balance in perioperative patients is positive.

With consternation we became aware that the PRAC ignored and contradicted every single point not only of their own expert committee, but also statements from international Anaesthesiology Societies and existing well-acknowledged guidelines. The underlying rationale and motivation for such ignorance is not transparent and unacceptable.
Obviously, the EMA followed the opinion of two Sepsis Societies and individual Intensivists. This is especially astounding, as for the patients treated by these specialists there is a clear contraindication which was never challenged in this procedure. Even more incomprehensible is the fact that the discussions about HES are derived from 3 trials performed in critically ill patients.[3-5] VISEP and 6S have been criticised for their methodological shortcomings, contradictive results and large amounts of HES given over

days after initial resuscitation.[6] Initially, in both trials, critically ill septic patients were nearly all resuscitated with colloids and over 50% of the patients in the respective crystalloid groups received HES at some time. In VISEP, 40% of the HES group received drug overdoses. A recently published prospective observational study in 65 German ICUs showed that duration and amounts of colloids infused in daily practice were dramatically lower than in the two above-mentioned trials.[7] There were no signs of increased mortality or renal insufficiency caused by colloids in general nor by HES in particular. The third trial is CHEST, which has aroused suspicion to have manipulated their data, as the original protocol, statistical analysis plan and various numbers have been changed since initial publication.[8] The request for an independent audit and re-analysis of the data proposed by various National Societies, the independent EMA expert committee and international journals (e.g. the BMJ), has been refused by the authors. In the meantime a so called "independent" re-analysis has been published as a letter to the Editor by the original authors, raising more questions than it answers.[9] To our bafflement, the EMA has not initiated any measures to investigate this critical issue. This is especially surprising, since the principle investigators of these questionable trials are among the strongest advocates for the suspension of the HES marketing authorisation. Their recent open letter to the WHO[10] just shows how desperate they are to protect their data and end all discussions.

In contrast to the above mentioned trials, patients included in the CRISTAL trial were randomised before resuscitation, and the study centers strictly abided to the respective groups.[11] In this study, 90-day mortality was significantly improved in the colloid versus the crystalloid group. A subgroup analysis revealed that HES was the only advantageous colloid. This adds to the problem of suspending HES, since alternative colloids are not superior, have a limited availability and are also very expensive (albumin). Gelatin's safety is sparsely evaluated, and this colloid is characterised by a lower effectiveness. Although Dextrans certainly have the worst benefit/risk ratio among all colloids, they are mentioned as alternative for HES on the EMA website.

In the UK, the yellow card pharmacoviligance system never recorded more than 1 serious adverse event of HES per year with about 100 events for crystalloids. Following the termination of HES sales in 2013, the adverse event rate for crytalloids in the UK has increased by 262%, clearly showing that they are not necessarily a better alternative to HES.

Apart from that, several unmet clinical needs such as plasmapheresis, use in paediatrics (especially cardiac surgery) and prevention of hypotesion in paturients undergoing caesarean section with spinal anaesthesia remain unattended.

Two major trials in trauma and surgical patients have been requested by the EMA. These already initiated trials are designed to provide answers to open questions and generate further evidence on the safety and efficacy of HES in these important clinical settings. The decision to suspend HES without any signs of harm in these populations and not awaiting the trial results is a politically driven decision that is neither based on clinical, nor on scientific grounds. This is reflected by the fact that a majority of the National Anaesthesiology Societies in Europe does not support the suspension of the HES marketing authorisation, while only 6 out of 36 National Societies, including 3 Non-EU

members, explicitly do so. It is also noteworthy that the PRAC members with divergent opinions expressed in writing that they disagree not only with the recommendation but especially with the procedure and the handling of this case by the EMA. Similarly, the ad-hoc expert group appointed by the EMA published a letter emphasising that they strongly disagree with the current recommendation and the entire procedure.[2]

The arguments of the EMA a) that doctors ignore contraindications, b) that it is too difficult to distinguish between patients who might profit or be harmed from HES and c) that further measures would be ineffective, are false and are discrediting physicians all over Europe .

If this procedure is to form a precedent, then from now on, no existing drug might be considered as safe any longer, as any politically active group may trigger a suspension based on the fact that certain patients might experience adverse events, and because an off-label may not always be prevented.

We, the National Societies of Anaesthesiology in Europe*, demand that the EMA pays attention to medical experts and reconsiders their completely unfounded decision. It is time to base the decision on clinical facts and scientific data rather than on political standpoints and questionable data that are hidden and not shared by the investigators despite numerous requests.

Sincerely,

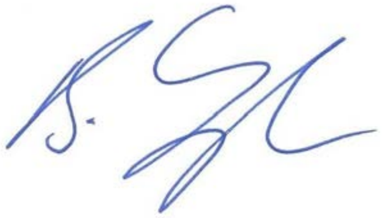

Prof. Dr. B. Zwißler
President

Literature

1. Marx G, Schindler AW, Mosch C, et al. Intravascular volume therapy in adults: Guidelines from the Association of the Scientific Medical Societies in Germany. Eur J Anaesthesiol. 2016 Jul;33(7):488-521.

2. Annane D, Fuchs-Buder T, Zoellner C, Kaukonen M and Scheeren TWL. EMA recommendation to suspend HES is hazardous Lancet, epub ahead of print DOI: https://doi.org/10.1016/S0140-6736(18)30254-X

3 Brunkhorst FM, Engel C, Bloos F, et al. Intensive insulin therapy and pentastarch resuscitation in severe sepsis. N Engl J Med 2008; 358(2):125–39.

4 Perner A, Haase N, Guttormsen AB, et al. Hydroxyethyl Starch 130/0.42 versus Ringer's Acetate in Severe Sepsis. N Eng J Med 2012; 367(2):124–34.

5 Myburgh JA, Finfer S, Bellomo R, et al. Hydroxyethyl starch or saline for fluid resuscitation in intensive care. N Engl J Med 2012;367: 1901–11.

6 Chappell D, Jacob M. Hydroxyethyl starch - the importance of being earnest. Scand J Trauma Resusc Emerg Med. 2013 Aug 9;21:61.

7 Ertmer C, Zwissler B, van Aken H et al. Fluid therapy and outcome: a prospective observational study in 65 German intensive care units between 2010 and 2011. Ann Intensive Care 2018; 8:27

8 Doshi P. Data too important to share: do those who control the data control the message? BMJ 2016;352:i1027.

9 Doshi P. "Independent" reanalysis of landmark starch solutions trial was published by original authors. BMJ. 2017;358:j3552.

10 Roberts I, Shakur H. Bellomo R, et al. Hydroxyethyl starch solutions and patient harm. Lancet epub ahead of print. https://doi.org/10.1016/S0140-6736(18)30255-1

11 Annane D, Siami S, Jaber S, et al. Effects of fluid resuscitation with colloids vs crystalloids on mortality in critically ill patients presenting with hypovolemic shock: the CRISTAL randomized trial. JAMA 2013;310(17):1809–17.

*** This open letter is supported by the following societies of anaesthesiology in Europe:**

Austria Austrian Society of Anaesthesiology, Resuscitation and Intensive Care Medicine
Belgium Society of Anesthesia and Reanimation of Belgium
Czech Republic Czech Society of Anaesthesiology and Intensive Care Medicine
Estonia Estonian Society of Anaesthesiologists
France Société Française d'Anesthésie et de Réanimation
Germany German Society of Anaesthesology and Intensive care Medicine
Greece Hellenic Society of Anaesthesiology
Hungary Hungarian Society of Anaesthesiology and Intensive Therapy
Italy S. I. A. A. R. T. I.
Israel Israel Society of Anaesthesiologists
Lithuania Lithuanian Society of Anaesthesiology and Intensive Care
Netherlands Nederlandse Vereniging voor Anesthesiologie
Portugal Portuguese Society of Anesthesiology
Serbia Serbian Association of Anaesthesiologists and Intensivists
Slovakia Slovak Society of Anaesthesiology and Intensive Medicine
Slovenia Slovenian Society of Anaesthesiology and Intensive Care Medicine
Spain Sociedad Espanola de Anestesiologia, Reanimacion y Terapeutica del Dolor
Switzerland Swiss Society for Anaesthesiology and Resuscitation
Turkey Turkish Society of Anesthesiology and Reanimation

FRESENIUS KABI
caring for life

HES solutions should remain on the market

January 29, 2018

Fresenius Kabi does not concur with the position of the Coordination Group for Mutual Recognition and Decentralised Procedures - Human (CMDh) of the European Medicines Agency (EMA) to suspend the marketing authorisations of hydroxyethyl starch (HES) solutions for infusion in the European Union. Fresenius Kabi is convinced that HES products can be used safely and effectively in the approved indications.

The CMDh formed its position on the basis of two Drug Utilisation Studies (DUS) that retrospectively reviewed the adherence of hospital physicians to the 2014 revised European Product Information for HES products. The CMDh argued that HES products were used outside the approved indications or despite contraindications for certain patient groups. HES products have a positive benefit-risk ratio in approved indications. Therefore, a suspension would not be appropriate, but additional measures could be implemented to avoid off-label use of these products. Fresenius Kabi has proposed such measures in detail to the EMA.

Moreover, a group of renowned independent scientific experts recommended that HES products not be suspended, and voted by large majority in favor of keeping the products on the market. This ad hoc group of experts was set up by the Pharmacovigilance Risk Assessment Committee of the EMA and has concluded that HES products are well tolerated and effective therapeutic options for the treatment of hypovolaemia in surgical and trauma patients.

Fresenius Kabi strongly believes that it took appropriate measures to comprehensively inform health care professionals about the revised European Product Information for its HES products and is committed to implement further measures to increase adherence to it.

The CMDh member states did not reach a unanimous consensus, and the position has been forwarded to the European Commission for a decision. Fresenius Kabi is going to take legal actions in the event of a suspension of HES products, as these solutions should remain a therapy option for the benefit of patients.

Fresenius Kabi is a global health care company that specializes in lifesaving medicines and technologies for infusion, transfusion and clinical nutrition. The company's products and services are used to help care for critically and chronically ill patients. Fresenius Kabi's product portfolio comprises a comprehensive range of I.V. generic drugs, infusion therapies and clinical nutrition products as well as the medical devices for administering these products. Within transfusion technologies, Fresenius Kabi offers products for collection and processing of blood components and for therapeutic treatment of patient blood by apheresis systems. In the field of biosimilars, Fresenius Kabi develops products with a focus on oncology and autoimmune diseases. With its corporate philosophy of "caring for life", the company is committed to putting essential medicines and technologies in the hands of people who help patients and finding the best answers to the challenges they face.

Fresenius Kabi employs over 35,000 people worldwide. In 2016 the company reported sales of about €6 billion. Fresenius Kabi AG is a wholly owned subsidiary of the Fresenius SE & Co. KGaA health care group.

For more information visit the Company's website at www.fresenius-kabi.com

This release contains forward-looking statements that are subject to various risks and uncertainties. Future results could differ materially from those described in these forward-looking statements due to certain factors, e.g. changes in business, economic and competitive conditions, regulatory reforms, results of clinical trials, foreign exchange rate fluctuations, uncertainties in litigation or investigative proceedings, and the availability of financing. Fresenius does not undertake any responsibility to update the forward-looking statements in this release.

Management Board: Mats Henriksson (Chairman), Marc Crouton, John Ducker, Dr. Christian Hauer, Dr. Michael Schönhofen, Philipp Schulte-Noelle, Gerrit Steen
Chairman of the Supervisory Board: Stephan Sturm
Registered Office: Bad Homburg, Germany
Commercial Register: Amtsgericht Bad Homburg - HRB 11654

< Back to overview

Fresenius Kabi AG © October 2018

Go to top

Friday, January 26, 2018

B. Braun: HES should be kept on the market

B. Braun is convinced that HES can be used effectively and safely in the authorized indications.

Melsungen (Germany). B. Braun Melsungen AG stated today that medicinal products containing hydroxyethyl starch (HES) should be kept available for the treatment of patients. HES solutions are used as volume replacement solution in patients suffering acute blood loss due to a severe injury or surgical procedures when crystalloids alone are not considered sufficient. B. Braun is convinced that HES can be used effectively and safely in the authorized indications.

The Coordination Group for Mutual Recognition and Decentralised Procedures - human (CMDh), the committee in charge for the European Medicines Agency of the EU Commission, has recommended suspending the market authorization of HES in the EU through a majority vote. The final decision now lies with the EU Commission. The main reason for the CMDh vote was that HES products were frequently infused outside the authorized uses or despite contraindications for certain patient groups, e.g. in patients who are critically ill or those with sepsis A use outside the approved labelling information is called “off-label”. In these cases measures are required to avoid off-label-use, but cannot be a reason for suspending the authorization in indications with positive benefit-risk-ratio. The company is going to commence legal action against a decision, especially since an expert commission set up by the Pharmacovigilance Risk Assessment Committee (PRAC) of the European Medicines Agency (EMA) had voted by a large majority to continue to allow the use. The commission suggested to wait for the results of two current clinical studies on perioperative use and use in trauma patients to get more evidence about the positive risk/benefit balance. The expert commission came to the conclusion that HES has its place in the treatment of patients and should continue to be available.

Not all products are registered and approved for sale in all countries or regions. Indications of use may also vary by country and region. Please contact your country representative for product availability and information. Product images are for reference only.

Copyright © B. Braun Melsungen AG

EUROPEAN COMMISSION
DIRECTORATE-GENERAL FOR HEALTH AND FOOD SAFETY

Health systems, medical products and innovation
Medicines: policy, authorisation and monitoring

Brussels, 11 April 2018

**MEETING OF
THE STANDING COMMITTEE ON MEDICINAL PRODUCTS FOR HUMAN USE
HELD IN BRUSSELS ON 9 APRIL 2018**

SUMMARY RECORD

Chairperson: Ms O. Solomon

1. Opening and adoption of the Agenda

The agenda of the meeting was adopted without changes.

2. Item on which the Opinion of the Committee was asked under the examination procedure:

Draft Commission Implementing Decision of XXX concerning, in the framework of Article 107i of Directive 2001/83/EC of the European Parliament and of the Council, the marketing authorisations of medicinal products for human use which contain the active substance "hydroxyethyl starch (HES), solutions for infusion"

Pursuant to Article 107i of Directive 2001/83/EC based on concerns resulting from the evaluation of pharmacovigilance data, Sweden initiated a procedure on 17 October 2017 on medicinal products containing hydroxyethyl starch solution for infusion.

On 24 January 2018, the co-ordination group for mutual recognition and decentralised procedures for human use (CMDh), having considered the Pharmacovigilance Risk Assessment Committee (PRAC) recommendation dated 19 January 2018 with regards to medicinal products containing hydroxyethyl starch solution for infusion (HES), reached a position by majority that the marketing authorisations for the concerned medicinal products should be suspended. The CMDh position was forwarded to the European Commission.

On 27 February 2018, on the basis of the CMDh position, the Commission services submitted to the Standing Committee on Medicinal Products for Human Use for an opinion by written procedure a draft decision suspending the marketing authorisations for the medicinal products concerned. During the written procedure the Czech Republic, France and Spain raised concerns about the draft decision and requested the Commission to convene a meeting of the Standing Committee on Medicinal Products for Human Use. In accordance with Article 8(3) of the Rules of Procedure of the Standing Committee on

Medicinal Products for Human Use, the written procedure was terminated and a plenary meeting was convened to consider whether there were questions of a scientific or technical nature which the opinion of the European Medicines Agency ("the Agency") had not addressed.

On 9 April 2018, the Commission presented the above mentioned draft Commission decision to the plenary meeting of the Standing Committee.

The delegations of the Czech Republic, France and Spain presented their scientific and legal concerns regarding the basis of the draft Commission decision.

A number of Member States supported the Commission draft decision. Several members of the Standing Committee raised concerns with regard to CMDh position and PRAC recommendation. Some considered that questions remained regarding any unmet medical need and options for risk minimisation measures.

The written observations of the Member States submitted prior to the meeting and the content of the discussion during the meeting led the Chair of the Standing Committee to conclude that there were new questions of a scientific or technical nature, in particular with regard to any unmet medical need and the feasibility and likely effectiveness of risk minimisation measures, which had not been sufficiently addressed in the CMDh position and in the PRAC recommendation on which the position was based.

In light of these new questions, it was agreed that the Commission would suspend the decision making procedure and refer the CMDh position/PRAC recommendation back to the Agency for further consideration.

3. AOB

No additional point was added on the agenda.

Attendance List for the Standing Committee for Human Medicinal Products

9 April 2018

**Member State**	**Organisation**
Austria	Federal Ministry of labour, Social Affairs, Health and Consumer Protection
Belgium	Federal Agency for Medicines and Health Products
Croatia	Agency for Medicinal Products and Medical Devices
Cyprus	Ministry of Health, Cyprus
Czech Republic	State Institute for Drug Control
Czech Republic	Permanent Representation of Czech Republic to the EU
Denmark	Danish Medicines Agency
EMA	European Medicines Agency
Estonia	State Agency of Medicines
Finland	Finnish Medicines Agency (Fimea)
France	ANSM - Agence nationale de sécurité du médicament et des produits de santé
Germany	Federal Institute for Drugs and Medical Devices (BfArM)
Germany	Federal Ministry of Health
Hungary	Permanent Representation of Hungary to the EU
Ireland	Department of Health
Ireland	Health Products Regulatory Authority
Italy	Agenzia Italiana del Farmaco (AIFA) - Italian Medicines Agency
Latvia	State Agency of Medicines
Luxembourg	Ministry of Health Luxembourg, Health Directorate, Division of Pharmaceuticals
Malta	Malta Medicines Authority
Netherlands	Medicines Evaluation Board (CBG-MEB)

Poland	Permanent Representation of Poland to the EU
Portugal	INFARMED (National Authority of Medicines and Health Products, I.P.)
Romania	National Agency for Medicines and Medical Devices
Slovakia	Ministry of Health
Slovenia	Permanent Representation of the Republic of Slovenia to the EU
Spain	Spanish Agency of Medicines and Medical Devices
Sweden	Swedish Medical Products Agency
United Kingdom	Medicines and Healthcare products Regulatory Agency (MHRA)

Meeting highlights from the Pharmacovigilance Risk Assessment Committee (PRAC) 14-17 May 2018

Share

News 18/05/2018

PRAC concludes two referrals, maintains recommendation on HES solutions for infusion and issues precautionary advice on HIV medicine

PRAC confirms its recommendation to suspend hydroxyethyl-starch (HES) solutions for infusion in the EU

Following a request from the European Commission to look into certain aspects related to the suspension of the marketing authorisations for hydroxyethyl starch (HES) solutions for infusion, the European Medicines Agency's (EMA) Pharmacovigilance Risk Assessment Committee (PRAC) has confirmed its recommendation to suspend these products across the European Union (EU).

In January 2018, the PRAC recommended suspending the marketing authorisations for HES solutions for infusion because they continued to be used in critically ill patients and patients with sepsis despite restrictions introduced in 2013 to reduce the risk of kidney injury and death in such patients.

This recommendation was endorsed by the CMDh and sent to the European Commission. In April 2018, the Commission requested the PRAC to consider whether suspending the marketing authorisations could result in an unmet medical need. It also requested the PRAC to

consider the feasibility and likely effectiveness of additional risk minimisation measures.

After having assessed the relevant data on these specific aspects, the PRAC confirmed its previous recommendation that HES solutions for infusion should be suspended. The PRAC recommendation will now be sent to the CMDh for consideration at its meeting on 28-30 May 2018.

PRAC recommends new measures to minimise risk of rare but serious liver injury with Esmya for fibroids

The PRAC has completed its review of Esmya (ulipristal acetate), used to treat moderate to severe symptoms of uterine fibroids (benign tumours of the womb), following reports of serious liver injury.

After considering all the evidence, the PRAC concluded that the medicine must not be used in women with liver problems and that certain other patients may start new treatment courses provided they have regular liver tests. In addition, PRAC recommended that Esmya should be used for more than one treatment course only in women who are not eligible for surgery.

More information is provided below.

PRAC review of Zinbryta confirms medicine's risks outweigh benefits

Finalising its review of Zinbryta (daclizumab beta), the PRAC has confirmed the finding from its preliminary assessment that this multiple sclerosis medicine poses a risk of serious and potentially fatal immune reactions affecting the brain, liver and other organs.

On 6 March 2018, while the review was ongoing, the PRAC recommended suspension of the marketing authorisation of Zinbryta in the EU and a recall of the product from pharmacies and hospitals, following 12 reports of serious inflammatory brain disorders worldwide, including encephalitis and meningoencephalitis. On 27 March 2018, the European Commission withdrew the marketing authorisation of the medicine at the request of the marketing authorisation holder Biogen Idec Ltd.

The PRAC has now concluded its review of the available evidence on Zinbryta and maintained its initial recommendation. However, there are no immediate consequences of the PRAC's review as Zinbryta is no longer authorised in the EU.

More information is provided below.

New study suggests risk of birth defects in babies born to women on HIV medicine dolutegravir

The PRAC is evaluating preliminary results from a study which found four cases of birth defects such as spina bifida (malformed spinal cord) in babies born to mothers who became pregnant while taking the HIV medicine dolutegravir.

While the Committee is assessing the new evidence, it has issued precautionary advice. Dolutegravir should not be prescribed to women seeking to become pregnant and women who can become pregnant should use effective contraception while taking dolutegravir medicines.

More information is provided below.

Agenda

Agenda - PRAC draft agenda of meeting 14-17 May 2018

Recommendations by PRAC

Article-20 procedure: Esmya

Summary of recommendation

Article-20 procedure: Zinbryta

Summary of recommendation

Safety signal evaluation

New study suggests risk of birth defects in babies born to women on HIV medicine dolutegravir

Ongoing referrals

Article-31 referral: Quinolone- and fluoroquinolone-containing medicinal products	Under evaluation	EMA received a total of 113 registrations for the public hearing and reviewed all applica to compile a list of speakers. The PRAC adopted the agenda and the list of proposed speakers. EMA will now contact all registrants to inform them about the outcome of the selection. Further information will be made available soon.
Article-20 procedure: Xofigo	Under evaluation	PRAC discussed a list of experts for an ad-hoc expert group meeting and continued its assessment.
Article-31 procedure: Methotrexate containing medicinal products	Under evaluation	PRAC continued its assessment.

Related content

- Esmya: Article 20 procedures
- Hydroxyethyl starch (HES) containing medicinal products: Article 107i procedures
- Zinbryta: Article 20 procedures

Related content

- PRAC recommends new measures to minimise risk of rare but serious liver injury with Esmya for fibroids (18/05/2018)
- EMA review of Zinbryta confirms medicine's risks outweigh its benefits (18/05/2018)
- New study suggests risk of birth defects in babies born to women on HIV medicine dolutegravir (18/05/2018)
- Public hearings
- Pharmacovigilance
- Pharmacovigilance Risk Assessment Committee (PRAC): 14-17 May 2018
- PRAC: Agendas, minutes and highlights

- Pharmacovigilance Risk Assessment Committee (PRAC)

Related documents

Acronyms and abbreviations used in PRAC minutes (PDF/223.84 KB)

First published: 05/10/2012
Last updated: 22/07/2014
EMA/645658/2012 Rev. 2

Contact point

EMA press office
Tel. +44 (0)20 3660 8427
E-mail: press@ema.europa.eu
Follow us on Twitter @EMA_News

How useful was this page?

Add your rating ★★★★★

Product emergency hotline
OUTSIDE WORKING HOURS

ABOUT

Who we are
Human regulatory
Veterinary regulatory
Committees

ASK EMA

Send a question
FAQs
Access to documents

LINKS

Legal
Privacy
Complaints
Contacts
Glossary
Business hours and holidays

CONTACT

EMA
30 Churchill Place
Canary Wharf
London E14 5EU
United Kingdom

Tel: +44 (0)20 3660 6000

Fax: +44 (0)20 3660 5550

RSS feed Twitter YouTube

© 1995-2018 European Medicines Agency

European Union agencies network

An agency of the European Union

17 May 2018
EMA/171888/2018
Pharmacovigilance Risk Assessment Committee (PRAC)

Assessment report

Referral under Article 107i of Directive 2001/83/EC resulting from pharmacovigilance data

INN: hydroxyethyl starch

Procedure number: EMEA/H/A-107i/1457

Note:

Assessment report as adopted by the PRAC and considered by the CMDh with all information of a commercially confidential nature deleted.

30 Churchill Place ● Canary Wharf ● London E14 5EU ● United Kingdom
Telephone +44 (0)20 3660 6000 **Facsimile** +44 (0)20 3660 5555
Send a question via our website www.ema.europa.eu/contact

An agency of the European Union

© European Medicines Agency, 2018. Reproduction is authorised provided the source is acknowledged.

Table of contents

1. Information on the procedure 3

2. Scientific discussion 3

2.1. Introduction... 3

2.2. Data on efficacy 4

2.2.1. Abdominal surgery... 4

2.2.2. Orthopaedic surgery 5

2.2.3. Urological surgery... 5

2.2.4. Cardiac surgery 5

2.2.5. Paediatric surgery... 5

2.2.6. Neurosurgery 6

2.2.7. Caesarean section 6

2.2.8. Trauma patients 6

2.3. Data on safety 22

2.3.1. Estimated patients exposure in the European Union 22

2.3.2. Risks of renal impairment and mortality 22

2.3.3. Spontaneous reporting 32

2.3.4. Literature review 34

2.3.5. Stakeholder submissions 34

2.3.6. Risk minimisation measures... 35

2.3.7. Conclusion on safety 37

3. Expert consultation and Stakeholders inputs... 37

4. Benefit-risk balance 38

5. Revision of PRAC recommendation 40

5.1. PRAC discussion on unmet medical need 41

5.2. PRAC discussion on additional risk minimisation measures... 44

5.3. PRAC discussion on additional information received since January, 2018 PRAC recommendation... 47

5.4. Impact of the new information on the previously concluded benefit-risk balance 48

6. Condition for lifting the suspension of the marketing authorisations 49

7. Revised grounds for PRAC Recommendation 49

References 52

Appendix 1 60

Listing of stakeholders, including MAHs, who submitted responses to the Agency for EMEA/H/A-107i/1457 60

Appendix 2 63

Divergent positions 63

1. Information on the procedure

In 2013, following a review of the risk of kidney injury and mortality related to hydroxyethyl starch (HES) solutions for infusion, the Pharmacovigilance and Risk Assessment Committee (PRAC) recommended risk minimisation measures such as restrictions in use of these medicinal products. PRAC also recommended a drug utilisation study to evaluate the effectiveness of these risk minimisation measures.

Results from two drug utilisation studies, submitted by the concerned Marketing Authorisation Holders ("MAHs") in 2017, have shown that the recommended restrictions in use are not being adhered to.

On 17 October 2017, the Swedish Medical Products Agency raised serious concerns about the use of HES solutions for infusion and considered the suspension of the marketing authorisations, triggering consequently an urgent Union procedure under Article 107i of Directive 2001/83/EC, and requested the PRAC to assess the impact of the newly available data on the benefit-risk balance of HES solutions for infusion and to issue a recommendation on whether the relevant marketing authorisations should be maintained, varied, suspended or revoked.

2. Scientific discussion

2.1. Introduction

Hydroxyethyl starch (HES) solutions for infusion contain starch with different molecular weights (mainly 130kD; 200kD) and substitution ratios (the number of hydroxyethyl groups per glucose molecule). HES solutions for infusion are authorised worldwide for the treatment of hypovolaemia associated with various conditions.

In 2012 and 2013, PRAC reviewed the benefits and risks of HES solutions for infusion in the treatment and prophylaxis of hypovolaemia, within Article 31[1] and 107i[2] referral procedures. These reviews were triggered by the results from large randomised clinical studies[3,4,5] which showed an increased risk of mortality in patients with sepsis and an increased risk of kidney injury requiring dialysis in critically ill patients following treatment with HES solutions for infusion.

As result of the reviews, the PRAC recommended that use of HES solutions for infusion should be restricted to the treatment of hypovolaemia due to acute blood loss when crystalloids alone are not considered sufficient. The PRAC also contraindicated the use of HES in patients with sepsis or who are critically ill. Furthermore, the PRAC requested that, as conditions to the marketing authorisations of these medicinal products, further studies should be carried out on the use of these medicines in elective surgery and in trauma patients. The PRAC also required that drug utilisation should be studied to evaluate the effectiveness of the risk minimisation measures. The focus for the drug utilisation studies (DUSs) has been to evaluate the adherence to the restrictions in use, implemented in the product information, concerning the indication, posology, and contraindication for HES.

[1] http://www.ema.europa.eu/ema/index.jsp?curl=pages/medicines/human/referrals/Hydroxyethyl_starch-containing_solutions/human_referral_prac_000012.jsp&mid=WC0b01ac05805c516f
[2] http://www.ema.europa.eu/ema/index.jsp?curl=pages/medicines/human/referrals/Hydroxyethyl_starch-containing_medicines/human_referral_prac_000029.jsp&mid=WC0b01ac05805c516f
[3] Perner A, Haase N, Guttormsen AB et al. Hydroxyethyl starch 130/0.42 versus ringer's acetate in severe sepsis. N Engl J Med 2012;367(2):124-34
[4] Brunkhorst FM, Engel C, Bloos F et al. Intensive Insulin Therapy and Pentastarch Resuscitation in Severe Sepsis. N Engl J Med 2008; 358(2):125-39
[5] Myburgh J, Finder S, Bellomo R *et al*. Hydroxyethyl starch or saline for fluid resuscitation in intensive care. *N Engl J Med* 2012; 367:1901-11

On 5th July 2017 and 9th October 2017, results from two DUSs on the effectiveness of the implemented risk minimisation measures became available. These include drug utilisation data from 11 EU Member States. These data raise serious concerns as they showed use of HES solutions for infusion in patient populations which are contraindicated such as those who are critically ill, or with sepsis[3,4,5]. In light of the well-established risk for serious harm when HES solutions for infusion are used in patients with critical illness, including sepsis, together with the above-mentioned newly available data, Sweden triggered, on 17th October 2017, an urgent Union procedure under Article 107i of Directive 2001/83/EC. Due to the serious public health impact, Sweden was considering suspending the marketing authorisations for the above mentioned medicinal products, and therefore requested an urgent review of the matter at the European level, and asked the PRAC to assess the impact of the above concerns on the benefit-risk balance of HES solutions for infusion and issue a recommendation on whether the marketing authorisations of these products should be maintained, varied, suspended or revoked.

In its assessment, the PRAC considered the totality of evidence which includes all newly available data since the previous referral procedures, including results from DUSs, clinical studies, meta-analyses of clinical studies, post-marketing experience, Eudravigilance data, literature review, responses submitted by the marketing authorisation holders (MAHs) in writing and at oral explanations as well as stakeholders' submissions and views expressed by experts during an ad-hoc experts meeting, taking into account also the characterisation of benefits and risks concluded in the previous referral procedures.

2.2. Data on efficacy

The PRAC reviewed all the data available from clinical studies and meta-analyses since the previous referrals in order to assess whether this would provide new information on the efficacy of HES solutions for infusion.

The evidence for the authorised indication is based on clinical studies for which the sample size and the duration of follow-up are limited. Is it also noted that although the benefit of HES solutions for infusion has been demonstrated in terms of a volume-sparing effect, and there is some support for effects on short-term hemodynamic effects, it remains uncertain to what extent this translates into more patient-relevant outcomes.

The evidence, which has become available since the previous referral procedures, related to efficacy is summarised below.

2.2.1. Abdominal surgery

Results from 13 published studies in abdominal surgery have been reviewed. Nine (9) of these are RCTs, 4 of them double-blind and the other open-label. Of the 4 double-blind trials one was conducted in two centres while the rest were single-centre trials. The number of patients exposed to HES solutions for infusion in the double-blind RCTs range from 19-104. Overall, the 13 studies submitted by the MAHs provide some support for the expected volume-sparing effect of HES solutions for infusion but there is no convincing support that this effect translates into benefit in more clinically meaningful outcomes. While one of the more robust studies suggests an impact on complications, the results suggest some baseline imbalance in the study in spite of randomisation that could contribute to this observation (Joosten, A., et al., 2017). Another of the more robust studies suggests more complications in the HES solutions for infusion group (Yates, D.R., et al., 2014). In this study, 4 patients in the HES solutions for infusion group developed renal failure compared to none in the control group. These data confirm a volume-sparing effect but fail to provide new evidence for patient

benefit in terms of other down-stream outcomes. An unpublished systematic review and meta-analysis provided as a stakeholder submission arrives at a similar conclusion (Reinhart, K. and W. Schummer, 2017).

2.2.2. Orthopaedic surgery

The MAHs have submitted published results from 4 studies in orthopaedic surgery. The number of patients exposed to HES range from 19-59. The largest study does not provide information on use of HES solutions for infusion according to the approved indication (Zhang, Y., et al., 2017). The PRAC considers, therefore, that no relevant new information to support efficacy of HES solutions for infusion is provided.

2.2.3. Urological surgery

The MAHs have submitted published results from 3 studies in urological surgery. Two (2) of these are RCTs including respectively 18 and 57 patients exposed to HES solutions for infusion. The PRAC considers, therefore, that no new demonstration of efficacy beyond a volume-sparing effect is provided. No adverse effects on renal function are detected but the sensitivity of these studies to detect such effects is questioned.

2.2.4. Cardiac surgery

The MAHs have submitted published results from 21 studies in cardiac surgery. Six (6) of these are RCTs, 4 of them double-blind and the others open-label. The number of patients exposed to HES solutions for infusion in the double-blind RCTs range from 19-81. Two of the studies (Kimenai et al. 2013 and Joosten et al. 2016) are not considered by the PRAC relevant for assessment of the benefit-risk balance compared to crystalloid solutions because they did not include crystalloids as a comparator. The largest study suggests a negative impact of HES solutions for infusion on both coagulation and renal function (Skhirtladze, K., et al., 2014).

The data from cardiac surgery confirms a volume-sparing effect but fails to provide new evidence for patient benefit in terms of other down-stream outcomes. The safety data suggests an adverse effect on coagulation and bleeding in cardiac surgery associated with the administration of HES, and consequently confirms the warning introduced in 2013 that the use of HES solutions for infusion is not recommended in patients undergoing open heart surgery in association with cardiopulmonary bypass due to the risk of excess bleeding.

2.2.5. Paediatric surgery

The MAHs have provided four studies in paediatric patients. Three of these are in cardiac surgery - two small RCTs (30 and 35 patients exposed to HES solutions for infusion) and one observational study. The PRAC considers that the volume-sparing effect of HES solutions for infusion did not translate into any other benefit compared to Ringer’s lactate.

The forth study is a RCT in 60 children aged 1–12 years undergoing intracranial tumour resection and studied preloading with HES solutions for infusion compared to human albumin (Peng, Y., et al., 2017). This is not an approved indication. No differences were detected and no new information regarding current safety concerns were provided.

The PRAC considers that the large observational study in cardiac surgery which compares HES solutions for infusion to human albumin has such limitations from the design that meaningful conclusions of relevance for this referral cannot be drawn (Van der Linden, P., et al., 2015).

In the Stakeholder submissions, two further paediatric studies were identified (Van Der Linden, P., et al., 2013; Akkucuk, F.G., et al., 2013). Both were from cardiac surgery and did not provide any new information regarding benefit or safety.

Currently, the SmPCs for HES solutions for infusion state that data are limited in children; it is therefore recommended not to use HES solutions for infusion in this population. The data submitted does not provide any new meaningful information regarding the use of HES solutions for infusion in the paediatric population.

2.2.6. Neurosurgery

A stakeholder submission refers to a study, conducted in 40 neurosurgical patients with supratentorial mass lesions (Xia, J., et al., 2014). Patients were randomly assigned to receive HES solutions for infusion or Ringer's lactate in a goal-directed protocol. The volume-sparing effect of HES did not confer any benefits in brain relaxation scales, or measures of cerebral oxygenation and metabolism. Given the small size of this study, the PRAC considers that this data has limited value for assessing benefits and risks of HES solutions for infusion in this population.

2.2.7. Caesarean section

The MAHs have provided 13 references of medium to low quality relating to use of HES solutions for infusion for preloading before spinal anaesthesia for elective caesarean section. This is used in prophylaxis to prevent hypovolaemia expected from the vasodilatation caused by neuraxial anaesthesia. This is not an approved indication for HES solutions for infusion. The references submitted do not provide any meaningful information concerning efficacy in approved indications.

2.2.8. Trauma patients

The MAHs have provided six (6) studies to support a benefit from HES solutions for infusion in the trauma population. With regards to the James study from 2011 (James, M.F., et al., 2011) that was assessed as part of the previous referrals, the PRAC does not agree with the MAHs conclusions. In penetrating trauma with low degree of tissue damage, the use of HES 130/0.4 possibly has some advantages over saline in terms of lactate clearance. This is a surrogate outcome measure not entirely easy to interpret. For blunt trauma, no clinical significant differences between the fluids are seen. The limited observation time of up to 30 days prohibits any conclusions of the overall safety profile of HES 130/0.4 solutions for infusion. The small subgroups concluded on leave the results prone to bias.

In a meta-analysis (Zarychanski, R., et al., 2013), trauma was included as a pre-specified subgroup analysis. Based on pooling of six studies (James, M.F., et al., 2011; Myburgh, J.A., S. Finfer, and L. Billot, 2013; Myburgh, J.A., et al., 2012; Younes, R.N., et al., 1998; Nagy, K.K., et al., 1993; Carli, P., et al., 2000), there is no signal of benefit, with an increase in mortality associated with HES solutions for infusion but the estimate has limited precision due to the relatively low number of events.

Among the new studies submitted, one large observational study suggest that HES solutions for infusion is an independent risk factor for acute kidney injury (AKI) (Eriksson, M., et al., 2015) and another large observational study finds HES associated with development of the systemic inflammatory response syndrome (SIRS) and sepsis (Sprengel, K., et al., 2016). Changing fluid resuscitation protocols over the study period was not adjusted for in one of the studies (Sprengel, K., et al., 2016). The study by Eriksson is a well-performed study in a well-controlled setting and does not provide any reassurance regarding the use of HES solutions for infusion in trauma. The results suggest that HES is a risk factor for acute kidney injury in trauma patients (adjusted OR = 2.52; 95% CI 1.37 to 4.63),

independent of factors such as age, comorbidity, diabetes, injury severity, massive transfusion, admission systolic blood pressure, and sepsis.

Discussion on Efficacy

Based on the above, the PRAC concluded that the clinical studies which have become available since the previous referral procedures have small sample size, the duration of follow-up is limited, outcomes studied are surrogate endpoints, and they suffer from methodological limitations. It is, therefore, concluded by the PRAC that they do not provide any new significant clinical information on the efficacy of HES solutions for infusion.

Based on the totality of evidence, PRAC considered that there is no new meaningful data that changes the current characterisation of the efficacy profile and the demonstrated modest benefits of HES solutions for infusion in the approved indication.

Table 1. New evidence since 2013 of HES in **abdominal surgery** based on single studies

Study id / reference Study design	Population	Treatment	Key objectives/endpoints Outcome/Result	Rapp comment/conclusion
**Joosten et al. (2017)** RCT, double-blind, two centres	Adults, elective open abdominal surgery expected to last >3 h. Quite healthy patients selected.<br><br>N = 80/80	Multiple 100-ml fluid challenges (crystalloid vs. HES) guided by closed loop goal-directed strategy, using a stroke volume.	The total volume of study fluid was 40% lower in the colloid group. Lower Post-Operative Morbidity Survey score at day 2 post surgery and a lower incidence of postoperative complications.	Some support for a volume-sparing effect of colloids using specific GDVT protocol. Some imbalance with more high risk surgery and longer surgery duration in crystalloid group that could contribute to difference in complications. Long-term outcomes such as renal function not yet available.
**Yates et al. (2014)** RCT, double-blind, single-centre	Medium- to high-risk elective colorectal surgery<br><br>N = 104/98	6% HES (130/0.4, Volulyte) vs. crystalloid (Hartmann's solution) in a goal-directed therapy protocol.	No difference in GI morbidity on postoperative day 5 [30% in the HES group vs 32% in the crystalloid group; adjusted OR=0.96 (0.52–1.77)]. Subjects in the crystalloid group received more fluid [median (IQR) 3175 (2000–3700) vs 1875 (1500–3000) ml, P<0.001] and had a higher 24 h fluid balance [+4226 (3251–5779) vs +3610 (2443–4519) ml, P<0.001].	While there was a volume sparing effect this did not result in any difference in clinically relevant postoperative outcomes. There were more complications observed in the HES group compared to the crystalloid group. There were 4 patients with renal failure in the HES group and none in the crystalloid group.
**Feldheiser et al. (2013)** RCT, double-blind, single-centre	Adults, laparoscopic cyto-reductive surgery (ovarian cancer).<br><br>N = 24/24	HES vs. crystalloid administered to optimize stroke volume measured by oesophageal Doppler within a goal-directed haemodynamic algorithm.	Less amount of IV administered study fluids used in HES group during surgery. Intra- and postoperative urine output and perioperative plasma levels of creatinine were similar in both groups. No differences in the length of intensive care unit and hospital stay were found.	Small pilot study. Some support for a volume-sparing effect. No other differences observed. No meaningful information regarding current safety concerns.
**Amin et al. (2016)** Randomised, open-label, single-centre	Adults undergoing laparoscopic gastric bypass surgery<br><br>N = 42/41	Preoperative HES 130/0.4 10 ml/kg to a maximum volume 1000 ml compared to ringer acetate solution 10 ml/kg to a maximum volume of 1000 ml.	Increased creatinine clearance and urine output in HES group.	Journal not indexed in MEDLINE. Small unblinded study of preoperative HES without bleeding. Not approved indication. Uncertain estimates due to low sample size. No information regarding current safety concerns.

**Ghodraty et al. (2017)**<br>RCT, open-label, single-centre	ASA I-III patients 18 to 70 years of age, undergoing upper and lower open resection and anastomosis of GI tract.<br><br>N = 45/46	Intraoperative blood loss replaced with lactated Ringer solution 3:1 vs. 6% HES 130/0.4 in 1:1 ratio. Third space fluid loss replaced with 6 mL/kg/h infusion of lactated Ringer vs. 2 mL/kg/h infusion of 6% HES 130/0.4.	The time for the first flatus or bowel movement was recorded and used as the primary end point of the study. The total volume of crystalloid/colloid infused in crystalloid group was 54.0 ± 20.4 mL/kg while patients in colloid group were infused with 42.6 ± 12.5 mL/kg. There was no difference between crystalloid group and colloid group in the occurrence of postoperative AKI and anastomotic leak.	Imbalances between groups in important baseline characteristics in spite of randomisation. The results suggest a 12 hour reduction of time with postoperative ileus, related to fluid volume. This did not have any impact on length of hospital stay.
**Hung et al. (2014)**<br>RCT, open-label, single-centre	Adults with major abdominal surgery.<br><br>N = 41/39	Perioperative 0.6% HES 130/0.4 vs. lactated Ringer's solution to maintain a predefined target of MAP between 65 and 90 mmHg or CVP between 8 and 12 mmHg.	The total amount of fluid administrated was 1547.9 ± 424.0 mL in HES group and 2303.1 ± 1033.7 mL in LR group ($p < 0.001$). The tissue perfusion and did not differ significantly between group. Effects seen on coagulation in HES group.	Small study. Some support for a volume-sparing effect. No meaningful information regarding current safety concerns.
**Juri et al. (2017)**<br>Non-randomised, open-label, single-centre study	Laparoscopic surgery.<br><br>N = 45/45	HES 130/0.4 vs. Ringer's solution at a rate of 25 ml/min in both groups.	Fewer patients with hypotension (SBP < 90 mmHg or 80% of baseline) and smaller CO decreases in the HES group ($p < 0.001$). Lower incidence and slower onset of hypotension.	Severe methodological concerns. Not an approved indication. No new information on efficacy and no meaningful information regarding current safety concerns.
**Kammerer et al. (2017)**<br>RCT, open-label, single-centre study	Elective cystectomy<br><br>N = 53/47	Albumin 5% vs. balanced hydroxyethyl starch 6% (130/0.4) in a goal directed protocol.	Median cystatin C ratio between the last visit at day 90 and the first preoperative visit was 1.11 (IQR 1.01 to 1.23) in the albumin and 1.08 (IQR 1.00 to 1.20) in the hydroxyethyl starch group (median difference = 0.03; 95% CI, –0.09 to 0.08).	Comparison between HES and albumin. No meaningful new information regarding efficacy and current safety concerns.
**Kanda et al. (2015)**<br>RCT, open-label, single-centre study	Elective arterial bypass procedure on lower extremity<br><br>N = 11/11	A 500 mL infusion of HES vs. saline	CO increased after HES (3.5±1.1 L/min to 3.9±1.3 L/min, $P<0.05$), but there was no change in CO after fluid loading in the Saline group (3.4±1.1 L/min to 3.3±1.0 L/min, $P>0.05$).	Small study suggesting benefit from HES regarding short-term surrogate hemodynamic endpoints in vascular surgery.

**Kajdi et al. (2014)**<br>Retrospective observational study	Cytoreductive surgery due to peritoneal carcinomatosis<br><br>N = 54 patients underwent 57 procedures.	In addition to crystalloids, 51 patients received gelatine and 14 were also given HES, in a ratio of approximately 2.5:1.	Administration of HES had a significant negative impact on renal function (the Box-Cox transformed glomerular filtration rate (GFR) measured postoperatively (day one and two), in patients younger than 60 years (P <0.001). Adjustment made for preoperative GFR, blood loss, urine output, and different intravenous fluid preparations. Three patients (5%) suffered from acute deterioration of renal function during their hospital stay.	The MAHs description of the study does not fully recognise that the study found that administration of HES had a significant negative impact on renal function in patients younger than 60 years. The problem with an observational study in this context is agreed and discussed in the introduction of this report. This does not preclude that all available data should be carefully considered. The finding of reduced renal function associated with HES administration during elective surgery, is considered informative.
**Li et al. (2015)**<br>Non-randomised, open-label	Major abdominal cancer surgery (83% by laparoscopy), ASA I-II<br><br>N = 41/39	Four sequential fluid programs. First-line treatment with 9 ml/kg of either 6% HES 130/0.4 or Ringer's lactate after induction of anaesthesia; second-line infusion with 12 ml/kg of either HES or Ringer's lactate over 1 h.	Administration of ≥ 2 L of Ringer's lactate increased the duration of paralytic ileus by 0.7 days and of food intolerance by 2 days. Only surgical complications prolonged the length of hospital stay.	Not an approved indication. Severe methodological concerns. No meaningful new information regarding efficacy and current safety concerns.
**El-Fandy et al. (2014)**<br>Unclear design	Adults undergoing major abdominal surgery<br><br>N = 50	Comparing HES to gelatin.	No significant difference in the volumes of both colloids given to achieve the required haemodynamic endpoints.	Study Published in the Journal of Egypt Soc Parasitol. The full text article is not provided in the submission. Information provided does not suggest that it is of relevance to the current safety issues or provides meaningful efficacy data.
**Spies et al. (unpublished)**<br>RCT, double-blind	Elective pancreatic surgery<br><br>N = 19/21/21	Up to 30 mL/kg HES 10% and up to 50 mL/kg of HES 6% during surgery, vs. balanced crystalloid solution	A statistically significant lower amount of HES solution in the 10% compared to the 6% group (p=0.0024). Stroke volume increased in HES groups. The median time until fully on oral (solid) diet did not differ between groups.	Unpublished results from pilot phase of study that was terminated due to futility. Small study suggesting benefit from HES regarding short-term surrogate hemodynamic endpoints. No meaningful new information regarding efficacy and current safety concerns.

Table 2. New evidence since 2013 of HES in **orthopaedic surgery** based on single studies

Study id / reference Study design	Population	Treatment	Key objectives/endpoints Outcome/Result	Rapp comment/conclusion
**Kancir et al. (2014)**<br>RCT, double-blinded, single-centre.	Elective hip-replacement under spinal anaesthesia.<br><br>N = 19/19	6% HES 130/0.4 or isotonic saline 0.9%; 7.5 ml/kg during the first hour of surgery and 5 ml/kg during the following hours.	No significant differences in neutrophil gelatinase–associated lipocalin (u-NGAL), plasma creatinine, and urine albumin during the study. U-NGAL and urine albumin increased significantly in both groups the morning after surgery but was normalized at follow-up after 10 to 12 days. Mean arterial pressure was significantly higher during the recovery period in the HES group (91 [13] and 83 [6] mmHg, mean [SD], P < 0.03) but not during surgery. No difference in use of phenylephrine	Small RCT that did not detect a nephrotoxic effect of HES in these healthy patients undergoing elective surgery. Assay sensitivity can be questioned. Not adequately powered to analyse hemodynamic differences between HES and saline in detail. No meaningful new information regarding efficacy and current safety concerns.
**Zhang et al. (2017)**<br>RCT	Patients aged >65 years, ASA I–III, undergoing hip arthroplasty under spinal anaesthesia<br><br>N = 59/59	6% HES 130/0.4 or sodium lactate Ringer's solution 7.5 mL/kg during the first hour of surgery.	The groups were balanced in MAP, urine and plasma NGAL, plasma IL-18 and creatinine, urine β2 microalbumin and albumin (P > 0.05). Urine IL-18 was dramatically elevated in both groups after surgery (P < 0.05), but did not vary significantly between the groups (P > 0.05).	Administration of HES not according to label. Minimal blood loss during surgery. No follow-up beyond 5 days after surgery. Sensitivity to detect any adverse effects of HES questioned.
**Hamaji et al. (2013)**<br>?	Hip arthroplasty<br><br>N = 48	6% HES 130/0.4 vs. lactated Ringer's solution		Study published in Portuguese not further critically assessed in detail. Based on the MAH description the study does not add meaningful information regarding the current safety issues.
**Pinar et al. (2015)**<br>RCT, open-label, single-centre study	Knee arthroscopy and below-knee minor orthopaedic surgery. ASA I-II. 18-65 years of age.<br><br>N = 20/20	Fluid preloading NaCl 0.9% 10 mL/kg for 20 minutes vs. HES 6% for an equal period.	No significant difference between groups with respect of hemodynamic data.	Administration of HES not according to label. No meaningful new information regarding efficacy and current safety concerns.

Table 3. New evidence since 2013 of HES in **urological surgery** based on single studies

Study id / reference Study design	Population	Treatment	Key objectives/endpoints Outcome/Result	Rapp comment/conclusion
**Kancir et al. (2015)** RCT, double-blinded, single-centre.	Radical prostatectomy under general anaesthesia.<br><br>N = 18/18	6% HES 130/0.4 or isotonic saline 0.9%; 7.5 ml/kg during the first hour of surgery and 5 ml/kg during the following hours.	No significant differences in markers of renal injury during the study. Hemodynamic stability and infused fluid volume were the same in both groups. We observed an increased blood loss in the group given 6% HES 130/0.4.	Small RCT that did not detect a nephrotoxic effect of HES with a 15 day follow-up in these patients undergoing elective surgery. Assay sensitivity can be questioned. Not adequately powered to analyse hemodynamic differences between HES and saline in detail. No meaningful new information regarding efficacy and current safety concerns.
**Südfeld et al. (2016)** Retrospective observational study	Radical prostatectomy. Selected patients with complete cystatin C and fluid therapy data available at postoperative days 1, 3, and 5<br><br>N = 179	Median HES 130/0.4 dose of 1,000 mL and a median crystalloid dose of 3,500 mL.	Median HES (25th to 75th percentile) dose of 1000 mL (1000 to 1000 mL). Baseline $eGFR_{cyst\ C}$ was 109.4 mL/min (100.3 to 118.7 mL/min). $eGFR_{cystC}$ on postoperative days 1, 3, and 5 was 120.4 mL/min (109.4 to 134.0 mL/min), 120.4 mL/min (109.4 to 132.9 mL/min), and 117.9 mL/min (106.6 to 129.8 mL/min), respectively ($p < 0.001$ compared with baseline). No patient had an $eGFR_{cystC}$-decrease of ≥25 % from baseline.	No adverse effects on renal function compared to baseline detected in this observational study.
**Szturz et al. (2014)** RCT	Urological patients<br><br>N = 57/58	HES 6 % 130/0.4 vs. Ringer's solution and administration of vasoactive drugs	Compared volume effectiveness of crystalloid and colloid substitution aimed to maintain the cardiac index (CI) between 2.6 and 3.8 $l/min/m^2$ as measured by transesophageal Doppler. 5000 ml of crystalloids was administered vs. 1500 ml colloid.	Full text article not provided in the submission. Volume-sparing effect as expected. No meaningful new information regarding efficacy and current safety concerns.

Table 4. New evidence since 2013 of HES in **cardiac surgery** based on single studies

Study id / reference Study design	Population	Treatment	Key objectives/endpoints Outcome/Result	Rapp comment/conclusion
**Skhirtladze et al. (2014)** RCT, double-blinded, single-centre.	Elective cardiovascular surgery (coronary artery bypass grafting (CABG), valve repair or replacement, and surgery of the ascending aorta on cardio-pulmonary bypass.<br><br>N = 81/76/79	Up to 50 ml/kg/day of either HA, HES, or Ringer's lactate (RL) as the main perioperative fluid.	Blood loss was not different between the groups. However, 35% of RL patients required blood products, compared with 62% (HA) and 64% (HES; P=0.0003). More study solution had to be administered in the RL group compared with the colloid groups. Total perioperative fluid balance was least positive in the HA group compared with the HES and RL. Both colloids affected coagulation and caused slight increases in serum creatinine.	Authors' conclusion: "Despite equal blood loss from chest drains, both colloids interfered with blood coagulation and produced greater haemodilution, which was associated with more transfusion of blood products compared with crystalloid use only… the use of large amounts of HES and HA in elective cardiovascular surgery, as it was the case in this trial, might be harmful, since it appears to be associated with an increased risk for blood transfusion and the need for renal replacement therapy."
**Skytte Larsson et al. (2015)** RCT, open-label, single-centre.	Elective coronary artery by-pass surgery with cardiopulmonary bypass.<br><br>N = 15/15	Postoperative administration during 20-30 minutes of HES 60 mg/mL, 130/0.62 10 mL/kg vs. crystalloid 20 mL/kg.	Despite an increase in cardiac index and renal blood flow with both fluids, neither of the fluids improved renal oxygen delivery. They both induced haemodilution. The GFR increased in the crystalloid (28%) but not in the colloid group. The crystalloid increased the filtration fraction (24%) and renal oxygen extraction (23%).	Physiological study using HES not according to approved indication. No meaningful new information regarding efficacy and current safety concerns.
**Schramko et al. (2015)** RCT, double-blinded, single-centre.	Coronary artery bypass grafting or a valve procedure.<br><br>N = 19/15<br>(Randomization was stopped prematurely)	6% HES130/0.42 or Ringer-acetate solution for cardiopulmonary bypass (CPB) circuit priming.	Patients in the HES group needed more blood products. The total volume administered into the CPB circuit was lower in the HES than in the Ringer (RIN) group, 2905±1049 mL versus 3973±1207 mL (p=0.011), but there was no statistically significant difference in total fluid balance (5086±1660 mL in the HES group versus 5850±1514 mL in the crystalloid group, respectively).	Randomization was stopped prematurely after 35 randomised patients because of the published report where HES130/0.42 was associated with impaired renal function. An apparent volume-sparing effect did not translate into a meaningful difference in postoperative fluid balance and HES use was associated with a need for more blood products.

**Datzmann et al. (2017)**<br>Post-hoc analysis of RCT (HEPCON trial)	Low risk elective coronary artery bypass grafting<br><br>N = 22/22	No specified protocol administration of HES or colloids	Higher cumulative fluid balance within the first 24 hours after surgery for balanced HES 130/0.42 as compared to crystalloids (p=0.055). Blood coagulation was more compromised in the HES group at ICU arrival (factor II, p=0.0012; factor X, p=0.0031; thrombocytes, p=0.0010). Blood losses, volume balances and vasopressor dosages tended to be higher in HES-treated patients.	Small post-hoc analysis suggesting adverse impact of HES on coagulation as expected in this patient population.
**Boom et al. (2013)**				Comparison between HES and hyperosmolar sodium lactate during cardiac surgery. The comparison does not generate relevant data for this procedure.
**Mazer et al. (2015)**<br>Abstract RCT				Conference abstract of study funded by Fresenius Kabi. Insufficient information for detailed assessment. Not considered to provide meaningful new information regarding efficacy and current safety concerns.
**Hans et al. (2015)**<br>Retrospective study	Adult patients undergoing cardiac surgery<br>On CPB.<br><br>N = 240 propensity score matched patients in final analyses.	Balanced HES (130/0.4) or balanced crystalloids used for pump prime and intraoperative fluid therapy.	40% of the colloid group and 23% of the crystalloid group received blood products (OR=2.1 [1.2-3.8]). After bypass HES patients had lower haemoglobin levels and a higher cumulative chest drain output after 3 hours. HES patients required more transfusions, owing to greater haemodilution, HES-induced clotting disturbances, and bleeding.	As expected in this patient population HES was associated with increased need for blood products due to coagulation disorder.
**You et al. (2016)**<br>Retrospective study	Coronary artery bypass surgery<br><br>N = 149			Full text article not provided in the submission. Insufficient information for detailed assessment.
**Vives et al. (2016)**<br>Multicentre prospective cohort study	All consecutive adult cardiac surgery procedures (except heart transplantation)<br><br>N = 1058 (350 exposed to HES)	6% HES 130/0.4 or anything else.	After multivariable risk adjustment, HES use was not associated with AKI (adjusted OR 1.01, 95% CI 0.71–1.46, P=0.91). These results were confirmed by propensity score-matched analyses.	No adverse effects of HES detected but the data collected for the study did not capture the amount of 6% HES 130/0.4 given. It is a major limitation that exposure is not quantitative. The precision in the estimation is modest. Further, follow-up for AKI ended 48 hours after surgery.

**Lagny et al. (2016)** Retrospective single-centre observational study	Adult cardiac surgery on CPB<br><br>N = 606 (247 exposed to HES)	In early period balanced HES 130/0.4 as a pump prime (1,500 mL) and for intraoperative fluid therapy (1,000 mL). In later period only a balanced crystalloid solution was used.	HES associated with increased risk for postoperative AKI (adjusted OR 2.26; 95% CI, 1.40-3.80). HES patients also had a more positive fluid balance and a lower urinary output during the first 48 hours. The incidence of surgical revision for bleeding was greater in the HES group (4.6% v 1.4%).	Results suggest harmful effects of HES. The study design (comparison to historical control) is vulnerable to bias from other concomitant changes occurring over time.
**Gurbuz et al. (2013)** open-label, single-centre study	Isolated on-pump CABG procedure.<br><br>N = 100/100	HES 130/0.4 or balanced electrolyte solution as priming solution for CPB.	Postoperative exploration for bleeding, postoperative atrial fibrillation, and renal dysfunction more frequent in the HES group.	While the study is described as randomised this is not agreed. Every other patient was apparently consecutively given HES. If anything, the results may suggest harm from HES in terms of bleeding and renal dysfunction.
**Ryhammer et al. (2017)** Prospective observational study from 3 university hospitals.	Adult patients undergoing cardiac surgery<br><br>N = 17 742	HES versus crystalloids, HA versus crystalloids, and HES versus HA.	HES had no impact on new dialysis and 30-day mortality. A Cox proportional regression analysis showed that HES had no impact on 6-month mortality and new postoperative ischemic events.	Some support for the absence of harmful effects from HES when used in cardiac surgery. There is, however, a major concern that high postoperative drainage output, and the use of vasoconstrictors, inotropes, and transfusion were adjusted for in the analysis. This may have introduced a conservative bias.
**Kim et al. (2017)** RCT, open-label, single-centre	Off-pump coronary artery bypass graft surgery<br><br>N = 60/60	6% HES (670 kD/0.75) up to 20 ml/kg vs. crystalloid in GDVT protocol.	HES resulted in less volume used but did not cause less endothelial glycocalyx degradation or improved microvascular reactivity. HES was associated with impaired coagulation, more use of platelets, more postoperative bleeding, and lower postoperative urine output.	Some evidence of a volume sparing effect that did not translate into any other beneficial effect. Adverse effects in terms of coagulation and bleeding.
**Kimenai et al. 2013** RCT	CABG on CPB<br><br>N = 30/30	Priming of CPB with HES vs. Gelatin in combination with Ringer's lactate.	Total post-operative chest tube output was 500 ± 420 ml in the HES group versus 465 ± 390 ml in the Gelo group (p = 0.48). No significant differences were observed in coagulation tests or number of transfusions.	Use of Bonferroni correction in a small trial with many safety outcomes not considered appropriate. No adverse effects detected compared to Gelatin.
**Tobey et al. (2016)** Retrospective, single-centre	CABG and/or valve surgery on CPB<br><br>N = 1265 (887 colloid)	6% HES 130/0.4 or 6% HES 670/0.75.	Fresh frozen plasma, cryoprecipitate, and platelet transfusions were significantly higher with larger volumes of HES. HES also associated with increased risk for overall postoperative complications.	Backward selection procedure (P>0.05) not appropriate for variable selection. Results suggest impact of HES on bleeding and postoperative complications.

**Min et al. (2017)**<br>Retrospective	Off-pump CABG<br><br>N = 413 /249	Median volume of 6% HES 130/0.4 16mL/kg vs. no-HES group. Change of practice at specific time	Similar postoperative 24 hours blood loss. Bleeding-related reoperation (OR 2.44; 95% CI 0.64–9.34) associated with HES but poor precision in the estimate. Postoperative AKI occurred more frequently with HES than control.	Results suggest harmful effects of HES. The study design (comparison to historical control period) is vulnerable to bias from other concomitant changes occurring over time.
**Momeni et al. (2017)**<br>Retrospective	Elective/emergency cardiac surgery with or without CPB.<br><br>N = 1501	HES dose of <30 mL/kg vs. ≥30 mL/kg intra- and postoperatively.	In conditional regression analysis performed on the matched groups a lower weight-adjusted dose of HES was significantly associated with a reduced incidence of AKI (OR = 0.825 (95% CI 0.727–0.936).	Results suggest harmful effects on the kidney of HES. In the observational setting, however, confounding by indication is likely when comparing different HES doses.
**Lim et al. (2016)**<br>Observational study				Abstract only, not further assessed. Not sufficient detail for meaningful assessment and regulatory conclusions.
**Mahmood et al. (2015)**				Full text article not provided in the submission.
**Joosten et al. (2016)**<br>RCT, blinded, single-centre	Elective cardiac surgery with CPB.<br><br>N = 59/59	6% (HES) 130/0.40 vs. 130/0.42 (originate from different vegetable sources)	No difference in terms of bleeding or kidney function.	In line with the conclusions in the 2013 referral no difference is assumed between different sources for HES. This is confirmed by the results in this study.
**Moerman et al. (2016)**<br>RCT, double-blind	Elective CABG with CPB.<br><br>N = 20/20	HES 6% 130/0.4 in a balanced electrolyte solution vs. Gelatin for priming of CPB.	Differences in microvascular reactivity.	The clinical relevance of the measured differences uncertain. The comparison to gelatine of minor, if any, relevance to the present safety concerns.

Table 5. New evidence since 2013 of HES use in **paediatric patients** based on single studies

Study id / reference Study design	Population	Treatment	Key objectives/endpoints Outcome/Result	Rapp comment/conclusion
**Miao et al. (2014)**<br>RCT,	First open heart CPB surgery for congenital heart disease (only ASD and VSD).<br><br>N = 30/30	6% HES 130/0.4 vs. conventional 3.3% HA for priming of CPB circuit.	HES caused higher preoperative colloid osmotic pressure (p<0.01) and lower operative renal function and postoperative allogeneic blood volumes than the HA. No differences observed in serum creatinine, glucose, hematocrit or lactic acid levels.	Not approved indication. Only 6 h follow-up. No reassurance for current safety issues.
**Patel et al. (2016)**<br>RCT, double-blind, single-centre	Paediatric cardiac surgery patients age up to 3 years & weight up to 15 kg<br><br>N = 35/35/35	HES130/0.4 6% 20 ml/kg vs. HA 10 ml/kg vs. Ringer's lactate for priming of CPB circuit.	HA had higher perioperative platelet count, lesser postoperative blood loss and blood products requirement. HES had lower level of platelets postoperatively than Ringer lactate group but not associated with increase blood loss. HES did not affect renal function and haemostasis in this dose. Patients receiving Ringer lactate had positive fluid balance intraoperatively. All three groups have similar effect on renal & liver function, urine output, time to extubation, ICU stay and outcome.	The volume-sparing effect of HES did not translate into any other benefit compared to Ringer's lactate.
**Peng et al. (2017)**<br>RCT, double-blind, single-centre	Elective intracranial tumour resection<br><br>N = 30/30	Preloading either with HES 130/0.4 or 5% HA (20 mL/kg bw)	No differences in coagulation as assessed by TEG. Blood loss, blood products used, hemodynamic changes, and clinical outcomes were also similar.	Not approved indication. No new information regarding current safety concerns.
**Van der Linden et al. (2015)**<br>Retrospective	Children who underwent cardiac surgery between January 2002 and December 2010.<br><br>N = 1495 (1832 children reviewed)	4% HA was used until 2005; it was replaced by HES thereafter.	Intraoperative use of HES associated with a less positive fluid balance. Perioperative blood loss, volume of red blood cells and fresh frozen plasma administered, were lower in the HES group. No difference in incidence of postoperative renal failure requiring renal replacement therapy or of morbidity and mortality.	The comparison to historic controls severely limits the value of the study. Too many changes are expected to occur over time that can introduce confounding not measured and adjusted for.
**Akkucuk et al. (2013)**<br>RCT, open-label, single-centre	Cardiac surgery under CPB, aged 2–16 years<br><br>N = 12/12	Priming of CPB circuit either with HES (130/0.4) or RL.	No negative effects on renal function.	Given the small size of this study, not approved indication, and observation period limited to 48 hours after surgery, it adds no substantial information on HES in this population.

**Van der Linden et al. (2013)** RCT, double-blind, two-centre	Elective surgery for congenital heart disease in children aged 2–12 years<br><br>N = 31/30	HES vs. HA for perioperative volume replacement.	HES showed equivalence to HA with regard to volume replacement therapy.	A postmarketing commitment to the FDA. Comparison to HA of limited interest for the current safety concerns. Study not powered for safety endpoints.

Table 6. New evidence since 2013 of HES use in **caesarean section** based on single studies

Study id / reference Study design	Population	Treatment	Key objectives/endpoints Outcome/Result	Rapp comment/conclusion
**Mercier et al. (2014)** RCT, double-blind, multi-centre	Healthy parturients undergoing elective caesarean section.<br><br>N = 82/85	Preloaded before spinal anaesthesia with 500 ml of 6% HES (130/0.4) + 500 ml of RL vs. 1000 ml of RL	Incidence of both hypotension and symptomatic hypotension was lower in the HES group: 36.6% vs 55.3% (one-sided P=0.025) and 3.7% vs 14.1%. There was no difference in total phenylephrine requirements. The decrease in maternal haemoglobin value the day after surgery was similar in the two groups. Neonatal outcomes were comparable.	Not approved indication. The use of a one-sided P-value not endorsed. Study not considered formally positive. Hemodynamic benefit did not translate into any measurable difference in fetal safety.
**Alimian et al. (2014)** RCT, double-blind, single-centre	Healthy parturients undergoing elective caesarean section<br><br>N = 30/30/30	Preloaded before spinal anaesthesia with lactated Ringer's solution (1000 ml), saline 0.9% (1000 ml) or HES (7.5 mL/kg).	The incidence of hypotension and required dose of ephedrine was lower in HES group (p=0.008). There was no difference in umbilical cord blood PH or Apgar scores.	Not approved indication. Hemo-dynamic benefit did not translate into any measurable difference in fetal safety.
**Mitra et al. (2014)** RCT, multi-centre	Healthy parturients undergoing elective caesarean section.<br><br>N = 32/32	Preloaded before spinal anaesthesia with 10 ml/kg HES 130/0.4; 10 ml/kg SG (4% modified fluid gelatin) and 20 ml/kg RL	Fall in SBP (<100 mm Hg) in 5 (15.6%), 12 (37.5%) and 14 (43.8%) in groups HES, SG, RL respectively. Phenylephrine use and APGAR scores were comparable. Lower preloading volume and less intra-operative vasopressor requirement was noted in HES group for maintaining BP though it has no clinical significance.	Not approved indication. Methodologically weak study. Authors' conclusion: "*RL which is cheap, physiological and widely available crystalloid can preload effectively and maintain hemodynamic stability well in cesarean section and any remnant hypotension can easily be manageable with vasopressor*."
**Saghafinia et al. (2017)** RCT, open-label, single-centre	Elective caesarean section.<br><br>N = 60/60	7 mL/kg HES 6%vs. 15 mL/kg normal saline.	No significant difference in mean arterial pressure. Total dose of ephedrine and atropine were similar. No significant difference in Apgar score.	No beneficial effect of HES compared to crystalloid.

**Arora et al. (2015)**<br>RCT, open-label, single-centre	Elective caesarean section.<br><br>N = 30/30/30	10 ml/kg of 6% HES prior to spinal anaesthesia vs. 10 ml/kg of 6% HES after spinal anaesthesia vs. 10 ml/kg of Ringer's Lactate prior to spinal anaesthesia.	Incidence of hypotension and ephedrine use was higher with RL.	Not approved indication. Inappropriate volumes compared and results therefore not informative.
**Romdhani et al. (2014)**<br>RCT, open-label, single-centre	Elective caesarean section.<br><br>N = 105	Preloaded before spinal anaesthesia with 0.5L HES 130/0.4, vs. 1.5L saline	Incidence of hypotension lower in the HES group and compared to the saline group (p=0.028). There were no significant differences in ephedrine dose or nausea and vomiting. Neonatal outcomes were comparable.	Full text not provided or accessible. Hemodynamic benefit not clinically relevant and did not translate into any measurable difference in fetal safety.
**Matsota et al. (2015)**<br>RCT, open-label, single-centre	Healthy parturients undergoing elective caesarean section.<br><br>N = 16/16	Preloaded before spinal anaesthesia with 0.5 L HES 6 % 130/0.42 vs. 1 L Ringer's lactate	Incidence of hypotension was 73.3 % with crystalloid and 46.7 % with HES. Shorter duration of hypotensive episodes (p<0.001), and less usage of ephedrine and phenylephrine (p = 0.015 and p = 0.029, respectively). No difference in neonatal outcome.	Not approved indication. Very small open-label study. Hemodynamic benefit did not translate into any measurable difference in fetal safety.
**Bennasr et al. (2014)**<br>RCT, open-label, single-centre	Spinal anesthesia for elective caesarean section.<br><br>N = 60/60	Preloaded before spinal anaesthesia with 500 mL of HES 130/0.4 vs. 500 mL of normal saline.	Hypotension occurred in 24 patients with HES and 43 patients with saline (p = 0.001). Ephedrine consumption was significantly lower with HES (P = 0.005). Nausea, vomiting and headache incidence was higher with saline (p = 0.006). Apgar scores and umbilical blood gazes were comparable between groups.	Not approved indication. Article in French and not therefore not assessed in detail. Inappropriate volumes compared. Hemodynamic benefit did not translate into any measurable difference in fetal safety.
**Unlugenc et al. (2015)**<br>RCT(?)	Elective caesarean section.<br><br>N = 30/30/30	After induction of spinal anaesthesia, 10 mL/kg/h RL plus 1 L HES 130/0.4 vs. 1 L RL or a "keep the vein open" infusion of RL.	Incidence of hypotension 20% with HES and 43% with crystalloid (p<0.05). No difference in ephedrine dose.	Full text article not provided in the submission. Not sufficient detail for meaningful assessment and regulatory conclusions. Inappropriate volumes compared and results therefore anyway not informative.
**Terkawi et al. (2016)**<br>Retrospective, single-centre	Elective caesarean section.<br><br>N = 196/182	HES vs. crystalloid	No association between HES and increased perioperative blood loss. A statistically significant (but clinically irrelevant) difference in haematocrit, and ephedrine consumption in favour of the crystalloid group.	Observational study suggesting no benefit of HES vs. crystalloid. No difference in blood loss.
**Ghanei et al. (2016)**				Full text article not provided in the submission. Not sufficient detail for meaningful assessment and regulatory conclusions.
**Sun et al. (2016)**				Abstract only, not further assessed. Not sufficient detail for meaningful assessment and regulatory conclusions.

**Tör et al. (2016)**				Full text article not provided in the submission. Not found in PubMed. Not sufficient detail for meaningful assessment and regulatory conclusions.

Table 7. New evidence since 2013 of HES use in **trauma** based on single studies

Study id / reference Study design	Population	Treatment	Key objectives/endpoints Outcome/Result	Rapp comment/conclusion
**James et al. 2011** RCT, double-blind, single-centre	Penetrating or blunt trauma requiring >3 L volume resuscitation<br><br>Penetrating: N = 67 (36/ 31). Blunt: N = 44 (20/22)		Volume first 24 h. Penetrating trauma: HES vs. saline 5093 ± 2733 vs. 7473 ± 4321 mL. Blunt trauma: 6113 ± 1919 vs. 6295 ± 2197 mL. Blood transfusion first 24 h. Pen HES vs. saline 1553 ±1562 vs. 1796 ± 1361 mL. Blunt 2943 ± 1628 vs 1473 ± 1071 mL. Mortality equal between arms at day 30. Renal injury 0% in HES, 16% in saline. Lactate clearance ”better” in HES for penetrating, ”same” in blunt trauma. In penetrating a saline/HES ratio of 1:1.5 was found, in blunt trauma 1:1.	In penetrating trauma with low degree of tissue damage the use of HES 130/0.4 seems to have some advantages over saline. For blunt trauma no clinical significant differences between the fluids are seen. Observational time up to 30 days. The limited observation time prohibit any conclusions of the overall safety profile of HES 130/0.4.
**Eriksson et al. (2015)** Retrospective, single-centre	Trauma patients admitted to the ICU<br><br>N = 422	Resuscitation with HES 130/0.4 and other fluids	In multivariable regression analysis male sex, age, diabetes mellitus, nondiabetic somatic comorbidity, ISS >40, massive transfusion, and administration of HES (adjusted OR = 2.52; 95% CI 1.37 to 4.63) were independent risk factors for AKI.	Results suggest HES is independent risk factor for AKI. The potential for residual confounding remains a concern.
**Sprengel et al. (2016)** Retrospective, single-centre	Trauma patients with Injury Severity Score (ISS) >16<br><br>N = 2969	Patients who did not receive colloids and those who received <5L colloids and >5L colloids within the first 48 h	The SIRS score increased with the amount of colloid used. However, the predictive quality was low, with an area under the ROC of 0.693 for SIRS and 0.669 for sepsis. Binary logistic regression revealed colloids as an independent factor for the development of SIRS and sepsis (OR: SIRS 3.325 and sepsis 8.984; P < 0.001).	Changing fluid resuscitation protocols over the study period not adjusted for.

**Leberle et al. (2015)**<br>Retrospective, single-centre	All patients admitted to the Trauma Centre<br><br>N = 260	<2000 ml HES 130/0.4 vs. >2000 ml HES during the first 24 h	Although high dose HES group had higher injury severity score, the incidence of AKI and RRT were comparable. Patients older than 59 years of age also similar results regarding incidence of AKI and the rate of RRT.	No comparison to patients not receiving HES. High dose HES group younger age and more severely injured. No adjustment for confounding. Authors conclude that "major differences between the groups could not be controlled". In the analysis of elderly only 12 patients in the high dose HES group. No meaningful new efficacy or safety data provided.
**Masoumi et al. (2016)**<br>RCT, open-label, single-centre	Trauma patients with haemorrhagic shock.<br><br>N = 88	1.5 L of normal saline + 0.5 L of HES vs.<br>2 L of normal saline in emergency department.	Difference in base excess following intervention.	Small open-label study comparing inappropriate fluid volumes for a comparison of colloid to crystalloid. Insufficient description of analyses. Surrogate outcome measure that is difficult to interpret. A larger reduction following intervention could indicate better reperfusion and suggest a better outcome. Poor quality study that provides no meaningful new efficacy or safety data.
**Pshenisnov et al. (2016)**				Article in Russian. Not sufficient detail in abstract for meaningful assessment and regulatory conclusions.

2.3. Data on safety

An increased risk of mortality in patients with sepsis and an increased risk of kidney injury requiring dialysis in critically ill patients following treatment with HES solutions for infusion have been characterised based on results from large randomised clinical studies evaluated in the previous referral procedures ([1,2]).

It is noted that a randomised clinical trial (RCT) is the only type of study able to reliably detect and quantify these risks as caused by HES solutions for infusion. Spontaneous adverse event reporting cannot be informative of these outcomes related to HES solutions for infusion for several reasons. It is expected that the baseline risk for renal injury and death is high in many of the concerned populations, and there is a direct causal link between the indication for treatment with HES solutions for infusion (i.e. degree of hypovolaemia) and the risk for e.g. renal injury and death. The outcomes have also been observed only after long-term (90-day) follow-up in randomised controlled trials.

Results from observational studies of HES solutions for infusion are for the same reasons prone to residual bias. The absence of reliable and routinely used dynamic measures of degree of hypovolaemia makes it difficult to adjust for confounding by indication in statistical analyses. Observational studies must therefore also be interpreted with great caution.

2.3.1. Estimated patients exposure in the European Union

Overall in the EU, there has been a general decrease in patient exposure across HES solutions for infusion during the last ten year, period and from 2012 onwards. In 2008, the patient exposure was estimated at approximately 13.4 million patients, in 2012, at approximately 3.2 million patients and in 2016 at approximately 1.8 million patients, coinciding with the availability of new data as considered in the previous referral procedures and the PRAC recommendations related to the restriction of use of these products.

It is also noted that there are marked differences in the estimated patient exposure and extent of reduction between the EU Member States and between the HES solutions for infusion. In some EU Member States, exposure data shows a marginal decrease from 2012, suggesting only small changes in clinical practice despite the PRAC recommendations.

2.3.2. Risks of renal impairment and mortality

The PRAC reviewed the totality of the data available since the previous referrals in order to assess whether this would provide new information on these risks.

2.3.2.1. Clinical studies

The MAH submitted clinical studies conducted and/or published since the previous referrals involving the use of HES solutions for infusion in a range of clinical indications. These included clinical studies in surgery and trauma patients and studies in settings which has been contraindicated (sepsis and critical illness).

In this section, a summary of the key safety data which were assessed in the previous referrals is presented, as these are the reasons for the current recommendations in the product information, and explain the serious concerns expressed due to use in contraindicated populations. Thereafter, a summary of data which have become available after the previous referral procedures is given. Focus of this review is on any new data from clinical trials. Data have been submitted by the MAHs as well as by Stakeholders. The EMA has also undertaken a literature review, and a search in Eudravigilance.

Summary of key safety data from previous referral procedures

The main safety concerns are increased risk of mortality and adverse renal effects, in vulnerable patient populations. Treatment with HES solutions for infusion has been associated with increased risk of mortality at day 90 in two large randomised clinical trials in patients with sepsis and septic shock (6S, VISEP), see further below (Perner, A., et al., 2012; Brunkhorst, F.M., et al., 2008).

The potential mechanism behind adverse renal effects associated with HES solutions for infusion is not fully elucidated. Adverse renal effects of HES solutions for infusion, independent of the molecular weight or other differences in the product composition were reported in several clinical studies.

The VISEP study was conducted as a multicentre, two-by-two factorial trial, in 600 patients (537 included for ITT analysis) with severe sepsis randomised to receive either intensive insulin therapy to maintain glycemia or conventional insulin therapy and either 10% pentastarch, a high-molecular-weight 10% HES (HES200/0.5; hypertonic), or modified Ringer's lactate for fluid resuscitation (Brunkhorst, F.M., et al., 2008). The rate of death at 28 days and the mean score for organ failure were co-primary end points. The study results showed an increased rate of renal failure in patients with severe sepsis treated with HES (200/0.5) compared to patients treated with Ringer's lactate. At day 90, patients who had received HES, even when they received lower HES doses, were more likely to have renal failure than those who had received Ringer's lactate (30.9% vs. 21.7%, P = 0.04) and were more likely to need renal-replacement therapy (25.9% vs. 17.3%, P = 0.03). The PRAC acknowledged that a number of patients received higher dose of HES (200/0.5) (>22 ml/kg/d), however, the risk of RRT was also seen in patients treated with HES (200/0.5) at the recommended daily doses.

The 6S trial is a pragmatic randomised, multicentre, parallel-group, blinded trial which was conducted in 798 patients with severe sepsis receiving fluid resuscitation in ICU with either 6% HES (130/0.42) (n=398) or Ringer's acetate (n=400) at a dose of up to 33 ml per kilogram of ideal body weight per day[1]. Septic shock was present in 84% of patients of both groups. After 90 days 201 patients (51%) assigned to HES had died, as compared with 172 patients (43%) assigned to Ringer's acetate (relative risk, 1.17; 95% CI: 1.01 to 1.36). There was also a significantly higher risk for RRT in patients treated with HES (130/0.42) (22% (87/398) compared to patients treated with Ringers' acetate (16% (65/400)) (RR: 1.35; 95% CI: 1.01-1.80; P=0.04). The results were supported by multivariable analyses, with adjustment for known risk factors for death or acute kidney injury at baseline.

The PRAC considered that the 6S study was well-designed and adequately powered. Due to the double blinding and the multi-centre design of the study there is a low risk of bias. The 6S study showed a significant and clinically relevant higher risk for mortality at day 90 and need for RRT during the course of treatment in patients with severe sepsis and septic shock treated with HES (130/0.42) compared to Ringer's acetate.

The CHEST study is a randomised, multicentre, blinded, controlled study which was conducted in 7000 patients who had been admitted to an ICU in a 1:1 ratio to receive either 6% HES (130/0.4) in 0.9% sodium chloride or 0.9% sodium chloride (saline) for all fluid resuscitation until ICU discharge, death, or 90 days after randomisation (Myburgh, J.A., et al., 2012). The main subgroups were: surgical (approximately 42%), sepsis (approximately 29%) and trauma (approximately 8%) patients. Adult patients admitted to the ICU and whom the treating physician judged to require fluid resuscitation (bolus of intravenous fluid over and above that required for maintenance or replacement fluids) were included. It should be noted that some of the patients have been treated before randomisation. Fluid was administered to correct hypovolaemia at any time during the patients ICU admission. Patients who had received more than 1000ml of HES before screening were excluded.

In this study, RRT was administered to 7.0% (235/3352 patients) of patients treated with HES and in 5.8% (196/ 3375 patients) of patients treated with saline (RR: 1.21; 95% CI: 1.00-1.45; P = 0.04).

Being a pragmatic clinical trial the indication for RRT was according to clinical practice non-standardised and subjective. The decision when to start and stop RRT was purely dependent on the opinion of the physician (who were unaware of study group assignments) and may have included reasons other than reduced kidney function, such as over-hydration. This made it unlikely that the difference was caused by variations in the thresholds for initiating therapy.

This study also evaluated RIFLE criteria for adverse renal effects, which are composite of effects on serum creatinine levels and urine output. The results showed that renal risk (RIFLE-R) occurred significantly more often in the saline group (57.3%) as compared to the HES 130/0.4 group (54%; p=0.007). Likewise, renal injury (RIFLE-I) occurred more often in the saline group (38%) as compared to the HES 130/0.4 group (34.6%; p=0.005). In view of these results, a post hoc analysis was conducted. The results showed that serum creatinine levels were significantly increased in the HES group suggesting a progressive reduction in creatinine clearance, and urine output was significantly decreased in the HES group, as compared with the saline group, during the first 7 days (P = 0.004 and 0.003, respectively).

The PRAC noted that the number of patients who had chronic kidney disease at baseline has not been published, and the status of chronic kidney disease was also not specified. However, the following baseline data have been presented in the study publication. Serum creatinine in HES group was 101.5±57.1 µmol/l and 100.1±58 µmol/l in the saline group. Urine output 6 hours before randomisation was 453.5±418.3 ml in the HES group and 426.6±422.9 ml in the saline group. Therefore, there was no significant difference between both groups at baseline.

In conclusion, the CHEST study has shown an increased risk of RRT in patients treated with HES solutions for infusion compared to the patients treated with 0.9% NaCl solution.

CRISTAL, a pragmatic, open-label multicentre randomised clinical trial in critically ill patients conducted from February 2003 until August 2012 in 57 ICUs in France, Belgium, North Africa, and Canada was considered also in the 2013 Art 107i referral procedure (Annane, D., et al., 2013). The study included 2857 ICU patients that received either colloids (gelatins, dextrans, HES or albumin) or crystalloids (isotonic saline, hypertonic saline or any other buffered solution) open-label for all fluid interventions other than fluid maintenance throughout the ICU stay. 70% of patients in the colloid group received HES solutions for infusion as the colloid.

The results suggest no impact on the primary outcome death within 28 days (RR=0.96, 95% CI 0.88 to 1.04), death within 90 days (RR=0.92, 0.86 to 0.99), or renal replacement therapy (RR=0.93, 95%CI, 0.83 to 1.03). More days alive without mechanical ventilation was observed in the colloids group vs the crystalloids group by 7 days (2.1 vs 1.8 days, P = 0.01) and by 28 days (14.6 vs 13.5 days, P = 0.01).

The results from the study should be interpreted with caution. There are obvious concerns with the long duration of the trial and its open-label design. While the pragmatic approach used in the study may be endorsed, it carries an obvious risk that the selection of treatment (such as choice of fluid and treatment strategy) is strongly related to centre. This is a particular problem when the primary outcome (28-day mortality) can be expected to be strongly related to other centre-specific factors and with only a minor impact from the fluid treatment under investigation. In the study design, randomisation was stratified for diagnosis and centre. The final analysis, however, was apparently only stratified for diagnosis. Remaining centre effects may therefore have introduced a bias of the results.

Meta-analyses

Risk for increased mortality and adverse renal effects were confirmed by meta-analyses (Zarychanski, R., et al., 2013; Perel, P., I. Roberts, and K. Ker, 2013). The meta-analysis included 38 trials with 10,880 critically ill patients and compared HES solutions for infusion with crystalloids, albumins or gelatine (Zarychanski, R., et al., 2013). When 7 trials were excluded from an investigator whose subsequent research had been retracted because of scientific misconduct, HES was found to be associated with increased risk of mortality among 10290 patients (RR: 1.09; 95% CI): 1.02-1.17; (heterogeneity) I^2 0%). A subgroup analysis of 12 randomised clinical studies that used 6% HES 130/0.4 formulations only, confirmed the increased risk of mortality in patients treated with HES.

In the Cochrane review, a 10 % higher mortality rate was shown for patients who received HES (RR: 1.10; 95% CI 1.02 - 1.19) (Perel, P., I. Roberts, and K. Ker, 2013). It should be noted that two studies (Perner, A., et al., 2012; Myburgh, J.A., et al., 2012) contributed to 80% of the weight in the meta-analyses which were adequately powered and blinded, multicentre studies.

Summary of new data after the 2013 referral - sepsis

Several posthoc analyses based on the 6S trial have been published. One such focuses on the risk for bleeding and the results suggest association between HES solutions for infusion and bleeding in these patients with severe sepsis (Haase, N., et al., 2013). A posthoc analysis must be interpreted with caution but the results are plausible e.g. based on known effects on coagulation seen in cardiac surgery. The impact on renal function has also been further analysed and confirm the association between HES solutions for infusion and development of kidney injury measured as AKI stage in these patients with severe sepsis (Muller, R.G., et al., 2013). Another posthoc analysis suggests that variations in volumes of fluid administered are associated with clinical practice more than explained by patient characteristics (Hjortrup, P.B., et al., 2016).

A double-blind, randomised, controlled monocentric study (BaSES) was conducted from May 2005 to May 2011 on consecutive patients with sepsis, severe sepsis and septic shock in Basel, Switzerland (Siegemund, unpublished data). The purpose of this study was to determine whether initial infusion therapy with HES solutions for infusion and Ringer's lactate in septic patients reduces ICU and hospital length of stay without impairment of renal function. Patients were randomly assigned to volume therapy with Ringer's lactate and saline or HES 130/0.4 in the first five days of intensive care treatment. The study was completed in May 2011 and preliminary results were discussed within the 2013 Art 107i referral procedure. The study remains unpublished and cannot be assessed in detail. It is therefore not considered to provide relevant information for the current safety issues.

A multicentre prospective cohort study conducted in the emergency department (ED) of 33 academic hospitals in China enrolled 1095 patients with septic shock, haemorrhagic and traumatic shock, cardiogenic shock, neurogenic shock, anaphylactic shock, and burn shock (Guo, S.B., Y.X. Chen, and X.Z. Yu, 2017). The authors report that HES was given to in 29.6% of septic shock patients, and the mortality of septic patients who received HES was much higher than those who did not (38.2% vs. 25.1%, P = 0.006) but HES application was not an independent predictor of mortality in septic patients. A direct correlation between the indication for treatment with HES along with the dose of HES required, with the risk for renal injury and death is to be expected. The use of HES solutions for infusion and the volume required is therefore expected to be correlated with mortality risk. This problem is also illustrated in this study by bicarbonate and second-choice vasopressors being independent predictors of mortality. The study results are not considered to provide meaningful new information regarding the safety issues at hand.

In a network meta-analysis from 2014 (Rochwerg, B., et al., 2014) also the Annane trial discussed above was included, which was not the case for meta-analyses reviewed by the PRAC in the 2013 referral. Fourteen studies (18 916 patients) were included with 15 direct comparisons. The authors caution that the trials are heterogeneous in case mix, fluids evaluated, duration of fluid exposure, and risk of bias. Imprecise estimates for several comparisons in this network meta-analysis contribute to low confidence in most estimates of effect. The results suggest that balanced crystalloids are superior to saline (OR, 0.78 [CrI, 0.58 to 1.05]; low confidence), high-molecular-weight starch (OR, 0.82 [CrI, 0.60 to 1.13]; moderate confidence), and low-molecular-weight starch (OR, 0.75 [CrI, 0.58 to 0.97]; moderate confidence). They conclude that among patients with sepsis, resuscitation with balanced crystalloids or albumin compared with other fluids seems to be associated with reduced mortality.

Stakeholders have raised concerns regarding the conduct of the CHEST study. An independent analysis of this study has therefore been by conducted by the Duke Clinical Research Institute (DCRI) (Kajdi, M.E., et al., 2014). The PRAC has in the previous review noted potential limitations of the studies, including the CHEST trial. However, the PRAC considered that the data which were collected from these large randomised clinical trials were robust enough to establish a potential harm associated with HES, in particular with regard to the risk of mortality and renal failure. In the independent analysis of the CHEST study conducted by the Duke Clinical Research Institute (DCRI) (Kajdi, M.E., et al., 2014), minor differences in some secondary and tertiary outcomes were observed that did not affect the conclusions. The PRAC finds the reanalysis sufficiently reassuring and confirm the key results from the CHEST trial.

Discussion on safety in sepsis

Two MAHs conclude that “Considering the higher significance of RCTs and the large number of subjects enrolled in the CRISTAL trial, the MAHs of HES 130 conclude that, according to recently published data on HES in septic patients, use of HES 130 in septic patients in need of volume replacement is safe and might be beneficial in terms of reduced mortality, provided that the maximum daily dose of HES is not exceeded.”

The above MAHs’ conclusion is not endorsed by the PRAC. The conclusions by PRAC following the referral procedures in 2013 regarding harm in patients with sepsis remain unchanged after assessment of the data submitted. The PRAC noted the limitations of the CRISTAL trial, the additional concerns provided by posthoc analyses of the 6S trial. Furthermore, the network meta-analysis reviewed above that includes the Annane trial confirms a benefit for balanced crystalloids compared to HES (Rochwerg, B., et al., 2014). The PRAC considers that the MAHs statement that use of HES 130 in septic patients in need of volume replacement is safe and might be beneficial in terms of reduced mortality, provided that the maximum daily dose of HES is not exceeded, is not supported by data. The PRAC is of the view that harm in terms of renal injury and increased mortality has been established in patients with sepsis.

With regards to the CHEST study, an independent reanalysis has been conducted by the Duke Clinical Research Institute (DCRI) (Patel, A., et al., 2017). As detailed above, the PRAC did not concur with the criticism of this reanalysis and concluded that the reanalysis is reassuring.
Having carefully assessed new studies and taken into consideration their methodological limitations, the PRAC concluded that they do not provide new and robust data on the safety profile of HES solutions for infusion in sepsis.

Summary of data in critically ill (ICU patients)

No randomised controlled trials investigating on HES solutions for infusion administration ICU patients with other diagnoses than sepsis/septic shock or trauma have been published since October 2013. The CRISTAL trial was assessed already in the previous referral. It was stratified for ICU patients with hypovolaemic shock without sepsis or trauma (Annane, D., et al., 2013), but not specifically by the colloid used and thus no HES-specific results can be referred to this subset of patients.

The PRAC considered that the results from CRISTAL (Annane, D., et al., 2013) should be interpreted with caution. While the pragmatic approach used in the study may be endorsed it carries an obvious risk that the selection of treatment (such as choice of fluid and treatment strategy) is strongly related to centre. This is a particular problem when the primary outcome (28-day mortality) can be expected to be strongly related to other centre-specific factors and with only a minor impact from the fluid treatment under investigation. In the study design randomisation was stratified for diagnosis and centre. The final analysis, however, was apparently only stratified for diagnosis. Remaining centre effects may therefore have introduced a bias of the results. There are obvious concerns with the long duration of the trial and its open-label design. Other limitations are that in the colloid arm only 68.8% of patients received HES, the remaining patients received other colloids, including albumin and gelatin. It also appears that there was some overlap between treatments with the different colloids, and that some patients received more than one type of colloid. It is also noted that a high proportion of patients in both arms (585/1414 in the colloid arm and 685/1443 in the crystalloid arm) who received colloids prior to ICU administration.

Having carefully reviewed available studies and taken into consideration their methodological limitations, the PRAC concluded that they do not provide new and robust data on the safety profile of HES solutions for infusion in critically ill patients.

Summary of new data after the 2013 referral – Elective surgery and trauma

Data on potential harm in elective surgery and trauma remain inconclusive and conflicting. Two RCTs in elective abdominal surgery that report conflicting results regarding potential harmful effects (Joosten, A., et al., 2017; Yates, D.R., et al., 2014).

While the problems with an observational study in this context is agreed and previously discussed in this report, this does not preclude that all available data should be carefully considered. The results from an observational study on cytoreductive surgery are of interest (Kajdi, M.E., et al., 2014). The finding of reduced renal function associated with HES administration during elective surgery, and after adjustment for measured confounders, should be given due consideration and is considered informative. The selected study population is of particular interest since it represents very extensive surgery of long duration.

No new high quality data from RCTs in trauma is available. Observational studies suggest harm in terms of HES turning out as an independent risk factor for SIRS, sepsis and AKI (Eriksson, M., et al., , 2015; Sprengel, K., et al., 2016). The PRAC considers that these data should, however, be interpreted with caution.

Overall, no conclusive evidence of harm in elective surgery or trauma has been identified but the new data at hand does not add further reassurance. The conclusions regarding the need for further studies from 2013 remain valid.

Other clinical settings

Studies have also been presented for prevention of hypotension following spinal anaesthesia for caesarean section, which is not within the authorised indication and also in paediatric patients, for whom the use is currently not recommended. With regards to the hypotension in caesarean section, no new safety information is provided. In a recent Cochrane review of techniques for preventing hypotension during spinal anaesthesia for caesarean section, the authors are of the view that no conclusions can be drawn as regards to rare adverse effects associated with use of the interventions (for example colloids) due to the relatively small numbers of women studied (Chooi, C., et al., 2017).

This section should be read in combination with section 2.2 where further element on other clinical settings not covered above is also discussed.

Table 8. Evidence of HES use in **Sepsis/Septic shock** based on single studies

Study id / reference Study design	Population	Treatment	Key objectives/endpoints Outcome/Result	Rapp comment/conclusion
**Annane et al. 2013** Pragmatic RCT, open-label, multi-centre,	2857 ICU patients, stratified by sepsis, trauma, or hypovolaemic shock without sepsis or trauma<br><br>N = 1414/1443	Colloids (gelatins, dextrans, HES or albumin) or crystalloids (isotonic saline, hypertonic saline, or any other buffered solution). 70% in colloid group treated with HES.	Death within 28 days (RR=0.96, 95% CI 0.88 to 1.04). Death within 90 days RR=0.92, 0.86 to0.99). Renal replacement therapy RR=0.93 (95%CI, 0.83 to 1.03). More days alive without mechanical ventilation in the colloids group vs the crystalloids group by 7 days (2.1 vs 1.8 days, P = .01) and by 28 days (14.6 vs 13.5 days, P = .01).	No difference in primary endpoint between crystalloids and colloids. Limitations include open-label design and a recruitment period of 9 years. The lack of stratification for centre in analysis a potential limitation.
**Lyu et al. 2015**				Only abstract available, article in Chinese
**Siegemund - unpublished** RCT				The study is unpublished and cannot be assessed in detail. It is therefore not considered to provide relevant information for the current safety issues.
**Haase et al. 2013** Posthoc analysis	Posthoc analysis of 6S trial		HES associated with bleeding (RR=1.55; 95 % CI 1.16–2.08), severe bleeding (RR=1.52; 0.94–2.48), mostly during the first day. Adjusted HR for death among severe bleeding 1.74 (1.20–2.53).	A posthoc analysis must be interpreted with caution but the results suggest association between HES and bleeding in these patients with severe sepsis. The results are plausible e.g. based on known effects on coagulation seen in cardiac surgery.
**Müller et al. 2014** Posthoc analysis	Posthoc analysis of 6S trial		Maximal AKI stage was higher in the HES vs. Ringer's group within the first 5 days after randomisation (P = 0.03). An increase in AKI stage was associated with mortality (HR=1.35; 95% CI 1.22 to 1.49).	A posthoc analysis must be interpreted with caution but the results confirm the association between HES and development of kidney injury in these patients with severe sepsis.
**Hjortrup et al. 2016** Posthoc analysis	Posthoc analysis of 6S trial		After adjustment of baseline variables, multivariate analyses revealed that individual trial sites administered significantly different volumes of fluid resuscitation.	The results suggest that variations in volumes of fluid are associated with clinical practice not explained by patient characteristics.
**Anthon et al. 2017** Posthoc analysis	Posthoc analysis of 6S trial		Similar delta cytokine concentrations in the HES vs. Ringer's group.	A posthoc analysis that selects ~30 % of the original study population. May cause bias.
**Müller et al 2015** Posthoc analysis	Posthoc analysis of 6S trial		Resuscitation with HES vs Ringer decreased early endothelial damage. Although this finding should be interpreted with caution, it indicates that the increased mortality observed with HES in the 6S trial may not be explained by endothelial damage.	A posthoc analysis that selects ~30 % of the original study population. May cause bias. The results points to the challenge of using surrogate markers as outcome. No new relevant information for the current safety issues.

**Guo et al 2017** Multicentre prospective cohort study	EDs of 33 large academic hospitals located in 16 Chinese provinces			

Table 9. New evidence since 2013 of HES use in **critically ill (ICU patients)** based on single studies

Study id / reference Study design	Population	Treatment	Key objectives/endpoints Outcome/Result	Rapp comment/conclusion
**Taylor et al. 2016** Subset RCT	3450 (49%) of the 7000 patients in the CHEST trial.	HES vs. saline (see CHEST trial)	Although longer term clinical outcomes did not differ between HES and saline, the probability that hydroxyethyl starch is cost effective in these patients is low.	Health economic analysis of subset from CHEST trial. No benefit from HES compared to saline. No safety data of relevance for current safety issues.
**Wang et al. 2015** Observational, prospective, single-centre	Critically ill patients<br><br>N = 314	HES 130/0.4	HES administration and daily maximum dose of HES were not risk factors of AKI in critically ill patients (both P > 0.05). SOFA score, hypertension, blood glucose level on ICU admission, and presence of shock were independent predictors of AKI in critically ill patients. The cumulative dose of HES was not an independent risk factor for AKI.	This small observational study does not find an independent association between HES and AKI. This does not provide any further reassurance in the light of the results from the large randomised CHEST trial.
**Li et al. 2014** Retrospective, single-centre	ICU patients with diabetes<br><br>N = 1036		HES associated with higher risk of mortality (adjusted OR=1.60; 95%CI: 1.04-2.45)	Abstract only. Detailed assessment therefore not possible.
**Albrecht et al. 2016** Retrospective, single-centre	Surgical intensive care patients<br><br>N = 515/540/497	HES 6%, 130/0.4 exchanged to 4 % gelatin in June 2006 based on safety considerations.	ARF more frequent in the HES group compared to the crystalloid group. Mortality and maximum daily dose of HES was significantly correlated, but mortality and total amount of crystalloid or total fluid intake were not significantly correlated. Cumulative amounts of fluids given were significantly higher in HES group compared with crystalloid only. The need for renal replacement therapy and 30-day mortality were significantly higher, and intensive care unit and hospital stay was longer, compared with crystalloids.	The study provides no reassurance regarding the safety issues at hand. Acknowledging the difficulties with this type of study design for these products and outcomes, the findings suggesting adverse effects of HES on renal function and mortality, must be interpreted with caution.

Drug Utilisation Studies (DUSs)

Two DUSs sponsored by two MAHs (B. Braun and Fresenius Kabi) have been conducted in parallel and independently from each other in order to assess the effectiveness of the risk minimisation measures previously implemented via the product information by the PRAC.

The studies were similar in design – both were non-interventional multicentre studies, conducted by retrospective review of charts of patients who received HES solutions for infusion during the study period. Where possible, electronic patient data management systems were used to capture patients exposed to HES. One study also measured amount of HES in use captured by the study in relation to the estimated average total HES amount provided/used per study site. This proportion was found to be high. The two studies provide drug utilisation data from 11 EU Member States in total, including 3,890 and 3,055 patients, respectively. Data collection covered 5 months for the DUS sponsored by Fresenius Kabi and 10 months for the DUS sponsored by B. Braun. The DUSs were not designed to evaluate change of pattern during the period of the studies, nor to draw conclusions on patient outcomes.

Non-adherence to the product information was reported to range from 68% - 77%, including 20 – 34 % non-adherence to contraindications. On average, across all EU Member States included in the study, 9 % of patients exposed to HES solutions for infusion were critically ill, 5-8% of patients had renal impairment and 3-4 % of patients had sepsis. It should be noted that there was considerable variability in adherence, and thus in some EU Member States, these proportions of patients administered with HES outside the authorised conditions of use were considerably higher.

In terms of *adherence with the indication*, even though the challenges due to the retrospective review of patient charts were acknowledged, the results are considered by the PRAC sufficiently robust and reliable. Both studies showed consistent (>20%) usage in prophylaxis (rather than treatment) of hypovolaemia, which is not covered by the approved indication.

In terms of *adherence to the dose*, both of the DUSs showed high levels of adherence to the dose (30 ml/kg) and duration of treatment (maximum time interval of 24 hours). Considering that it is not possible to identify a cut-off level below which harm is either not present or is significantly diminished, and that evidence demonstrating harm includes evidence generated in patients treated at doses consistent with current recommendations, it cannot be concluded that the use in contraindicated patients seen in the DUSs is safe because of the doses used.

Of particular concern was the use in *patients with contraindications*, and especially in patients with sepsis, renal impairment, cardiac failure and critically ill patients which was shown consistently across both studies. Taken together the overall estimated yearly patient exposure from 2014 to 2017 of 1.5 to 2 million patients yearly during this period, the reported extent of exposure of patients with sepsis from the two DUSs (3 and 4 %, respectively), and despite taking into account potential uncertainties in these estimations, it is projected that at least tens of thousands of patients with sepsis have been exposed to HES solutions for infusion in the EU per year after 2013.

The PRAC also noted the variability in the results from the DUS across different countries points which points towards national differences in the use of HES solutions for infusion. This is further supported by the estimated patient exposure data, which shows marginal reduction in use of HES solutions for infusion in several member states since 2013, and more substantial reduction in other Member States.

The detailed PRAC assessment of the results has been shared with all the MAHs concerned by the referral procedure enabling a sufficient knowledge of the data underlying the assessment and full understanding of the conclusions of the PRAC and the reasons and grounds supporting these conclusions. Comments during the assessment were received by Fresenius Kabi, B. Braun, Serumwerk and Infomed Fluids. These comments were discussed and addressed by PRAC.

The limitations with the studies raised by the MAHs were considered by the PRAC. MAHs argued that some centres might have misclassified some events due to ambiguity with regards to the definitions of some recorded variables. It is noted that the study protocols developed by the MAHs and approved by the PRAC contain operational definitions of relevant co-morbidities present before HES-administration to be documented in the electronic Case Report Form (eCRF), as well as measures to ensure data quality, including that the respective CRO was responsible for training of each study site and that support from the CRO was available all working hours to investigators. Further, no potential problems regarding difficulties in recording of specific variables were reflected in the study reports. Thus, the issues stated by the MAHs seem to have been well addressed in the planning of the study, and relevant information was available to the investigators.

With regards to submitted post-hoc analyses of the results aiming to identify false positive data entries, they suffer from methodological limitations as they were not pre-defined. For instance, the potential for false negatives were disregarded, and selection of the subset of sites for re-analysis was done after the overall results were known, and was not done randomly. This analysis is, therefore, not considered sufficiently reliable by the PRAC. Besides, the methodological limitations of post-hoc analysis performed, the potential limitations of the DUSs suggested by the MAHs do not affect the key components in the results, namely use in sepsis patients, critically ill, and patients with renal failure. A concern regarding potential underestimation of the presence of sepsis is notable. Retrospective identification of sepsis based on ICD-9/10 coding (which was part of the operational definition of this contraindication in these studies) has been found to have low sensitivity but very high specificity (Gedeborg R., Furebring M., Michaëlsson K., 2007).

Considering that the data comes from two different DUSs conducted independently, that the sample is representative (different hospitals and departments in 11 Member States) and the magnitude and the consistency of the results, these studies are considered sufficiently robust and reliable and establish a degree of non-adherence to the product information of HES solutions for infusion that raises important public-health concerns.

2.3.3. Spontaneous reporting

Spontaneously reported data on suspected adverse drug reactions (ADRs), and the number of patients with suspected ADRs reported in association with HES solutions for infusion are available from Eudravigilance and from the submissions by the MAHs. The MAHs pointed to a reduction in spontaneous reports for HES solutions for infusion since the distribution of the DHPC in 2013, together with the absence of safety signals in the same period. However, number of reports before 2013 was not commented.

In addition, the available data for these products in Eudravigilance were reviewed by year from 2010-2017 at “total cases”, “SOC” and “PT” levels, including a specific review of the medication error SMQ. Overall, a relatively small number of cases have been received each year (Figure 1). Among the total number of cases reported, 0 – 2 cases with terms within the medication error SMQ were reported per year during this period.

As seen from Figure 1 below, numbers of cases as well as suspected ADRs have fluctuated during the period presented; 2010- 2017. In 2010 and 2013, the highest number was reported (n= 37 and 31 respectively). In 2011, 2012, and 2014, the numbers were lower, 15-18 patients / year, and fewer (n= 4-7) reported over 2015-2017 (although the full year data is not available for 2017). These data have not considered the reduced patients exposure which has emerged during the same time period.

A similar fluctuation in numbers is seen for renal events (figure 2), specifically with no events 2010 and 2011, few cases 2012, a peak in 2013, and thereafter a reduction but still with higher levels than

before 2013, in 2014 and 2015. Regarding the safety issues at stake it is noted (figure 2) that the number of reported events is very low or non-existing in the period before general attention to the PRAC review of data on harm from RCTs in 2013, and that there is an increase following 2013 despite a notable reduction of overall patient exposure.

It should be noted that the overall number of reports of suspected ADRs is very low in relation to millions of exposed patients, while the risks involved in this procedure are clearly established from several RCTs. Further changes in the levels of reporting, where the absolute numbers of reports are low, are especially difficult to interpret.No new safety signals have been identified from the spontaneous reporting data. However, this can not be interpreted as reassurance of lack of risk, as argued by the MAHs. Given the nature of these products, and the fact that they have been on the market for decades, a considerable level of underreporting of ADRs may be expected. In particular, these products are used in complex or emergency situations in which a patient is receiving multiple therapies; it is therefore difficult to identify a possible causative agent for any ADR experienced which may further impact level of reporting. The potential for a time delay between an acute exposure to HES and occurrence of renal impairment or death may also make it less likely for HES to be identified as a possible causative agent, further contributing to underreporting. Considering these limitations of spontaneous reporting, the risk of increased mortality and renal failure has been established and confirmed based on data from RCTs and not spontaneous reporting.

Figure 1 Number of Spontaneous Reports from the EEA for all HES solutions for infusion from Eudravigilance Database (2010 to 2017)

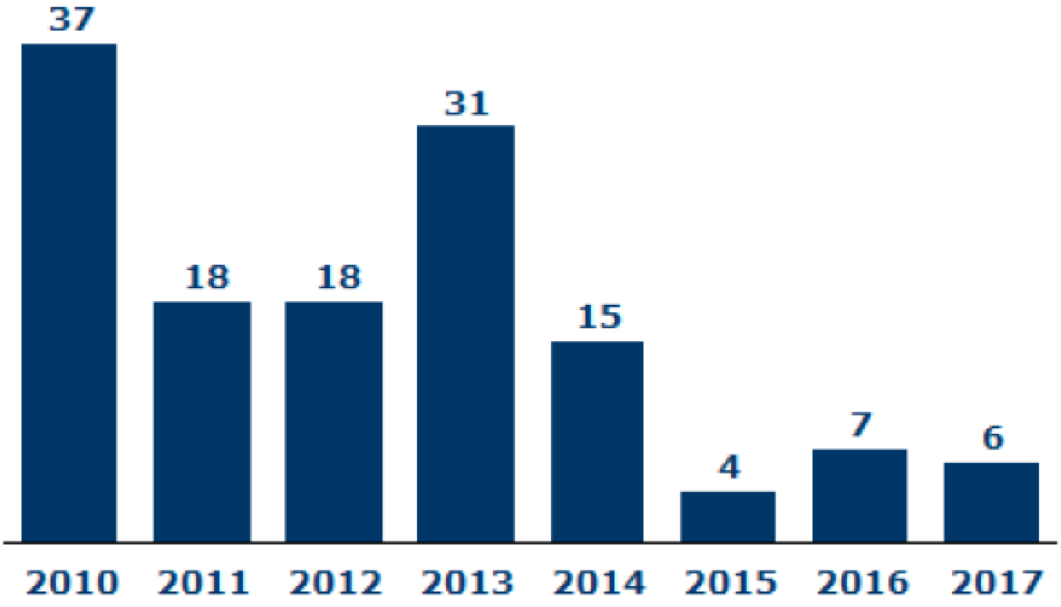

Figure 2 Number of adverse event reports for HES solutions for infusion in Eudravigilance in SOC Renal and urinary disorders in relation to overall estimated patient exposure to HES solutions for infusion as supplied by the MAH with the dominating market share. It should be noted that the number of events relates to all HES solutions for infusion.

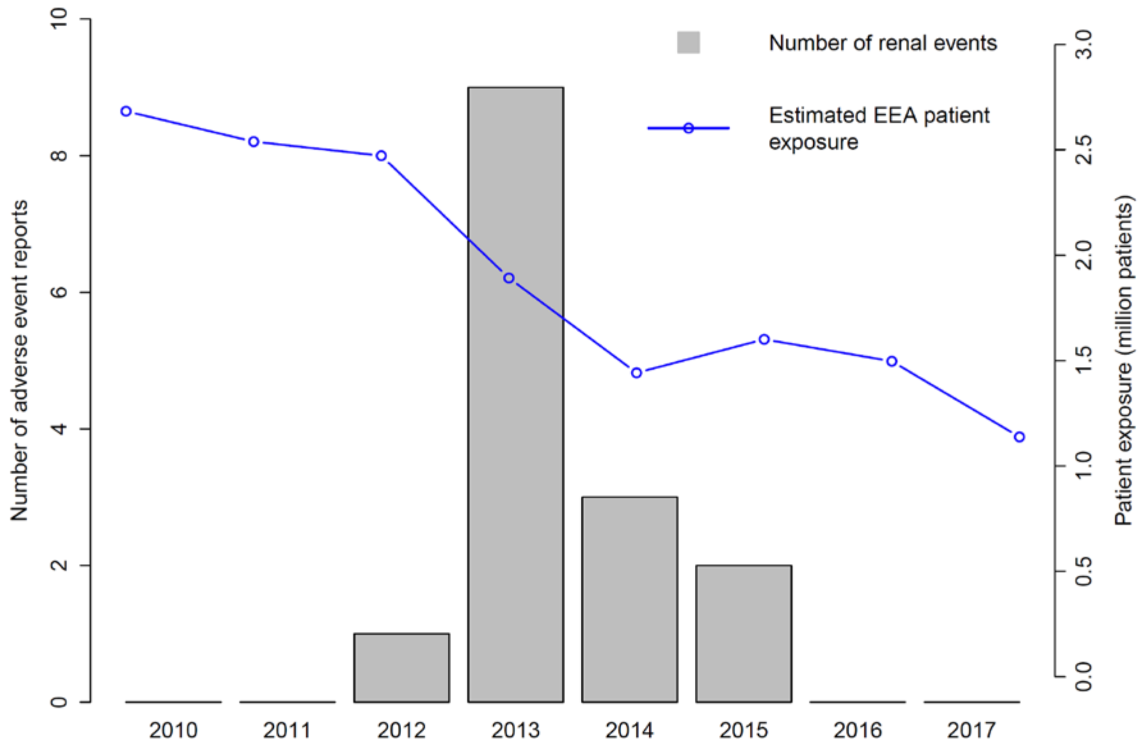

2.3.4. Literature review

A literature review covering the period from the previous referrals has been conducted by the EMA. The PRAC noted this literature review. The conclusions of the EMA review are consistent with other submissions in that most of the new evidence comes from small studies, and the results are not conclusive.

In the context of the submissions from the MAHs and other stakeholders, the PRAC considered that this literature review does not contribute with any significant new information related to the safety of HES solutions for infusion.

2.3.5. Stakeholder submissions

In total 30 responses were received from other stakeholders than the MAHs concerned by the procedure (cf. Annexed Listing of stakeholders, including MAHs, who submitted responses to the Agency for EMEA/H/A-107i/1457). The majority of comments came from individuals practising in anaesthesia or critical care medicine expressing their personal opinion with respect to use of HES solutions for infusion. Responses were also received from a number of professional societies, from a patient organisation and from a CRO.

These contributions provided feedback on clinical use, experience on the safety of HES solution for infusion safe and opinion on potential regulatory action such as maintenance, variation or suspension. The PRAC noted the following comments:

- In some EU Member States, the risk minimisation measures introduced in 2013 are not consistent with current clinical national guidelines, where a more extensive use is recommended. Several other stakeholders do also not agree with the PRAC regarding the patient populations in whom HES solutions for infusion should be used. One stakeholder referred to a survey among about 500 prescribers, which is stated to show that many of those also do not agree with the restricted use implemented by the PRAC.
- Some argued that there is no robust evidence that HES solutions for infusion can cause increased mortality or postoperative acute kidney injury in patients treated peri-operatively, or even at all, and it is stated in one submission that use in some of the contraindicated populations has not been shown to be harmful.
- Criticism of existing studies in critically ill patients, such as the CHEST trial which formed part of the evidence to support the restrictions introduced in 2013.
- Some pointed to an insufficient evidence base to justify continued use in the authorised indication. This included that data on benefits are weak, as even though volume sparing effects in comparisons with crystalloids have been shown, data are lacking on clinically significant benefits for patients receiving HES. Furthermore, that there is no scientific reason that the adverse effects of HES solutions for infusion are limited to sepsis and ICU patients, and that there are some data in support of this conclusion.
- Limitations of some alternative treatment options were discussed. This included a high cost of albumin and the potential for hypersensitivity reactions with gelatin, and the potential for tissue oedema and fluid overload following use of crystalloids.
- Some stakeholders query the value of the PHOENICS and TETHYS trials, noting the small sample size of the TETHYS (trauma) study and the use of surrogate endpoints in the PHOENICS study. In contrast, some responses call for any regulatory action to be postponed until these clinical trials are concluded.
- Several of the comments received points toward that the lack of adherence to the restrictions is an active choice rather than lack of awareness of the restrictions implemented in 2013.

The PRAC reviewed the 30 above-mentioned Stakeholders submissions and noted that a range of views is expressed from both individuals and organisations regarding the place of HES solutions for infusion in therapy of acute hypovolaemia. The PRAC concluded that these submissions contain no substantive new evidence on the safety and efficacy of HES solutions for infusion, but provide further support for the conclusion that the PRAC recommendations for use, as set out in 2013, are not agreed and/or followed by all prescribers, and therefore that lack of adherence to key restrictions is not solely due to lack of awareness from the prescribers.

2.3.6. Risk minimisation measures

Based on the above, the PRAC concluded that the risk minimisation measures introduced by the previous referral procedures have not been sufficiently effective as the evidence examined by PRAC demonstrates that the restrictions of the use of HES are not adhered to at a level sufficient to address the identified safety risks.

The MAHs submitted proposals for further risk minimisation measures. These consisted of changes to the product information, a DHPC, warning on the primary packaging and new educational materials.

MAHs also proposed involving learned societies such as European Society of Anaesthesiology (ESA) and European Society of Intensive Care Medicine (ESICM).

Furthermore, a sign-in for medication form and prescription sheet/checklists were proposed.

The PRAC also took into consideration the proposals from the ad hoc expert group as detailed in Section 3 of this Assessment Report.

The PRAC considered whether these risk minimisation measures proposed would be feasible and effective.

In particular PRAC took into account the stakeholder submissions, some of which implied that in some centres lack of adherence to the restrictions of use for HES established in 2013 is an active choice rather than lack of awareness of the restrictions implemented in 2013, and the fact that some of the restrictions implemented in 2013 were reported in the DUSs to be followed to a high degree, namely recommendations on dose and duration of treatment. PRAC considered that the lack of adherence to key restrictions is not solely due to lack of awareness from the prescribers. For that reason, further communication by the means of DHPC or educational materials/training would not alter prescribing behaviours sufficiently.

Updated national or European therapeutic guidelines from recognised learned societies that would reflect the conditions of use of HES solutions for infusion *as per* the terms of their MA may have some impact. However, this is beyond the measures that can be formally implemented by the EMA, the European Commission or the competent authorities of the EU Member States, and it is also doubtful that it would be feasible or could be achieved within a timeframe proportionate to the identified serious risks associated with exposure in contra-indicated settings. Indeed, these therapeutic guidelines are defined by learned societies, not by the national competent authorities or the MAHs.

With regards to the proposal to add a warning on the primary packaging "*Do not use in septic and renally impaired patients*", the PRAC considered that this measure would not be sufficiently effective as generally the prescriber does not administer the HES solution for infusion to the patient. The PRAC also expressed some concerns about highlighting these two specific contraindications in the primary packaging as this could detract from the importance of the other contraindications that may also lead to serious harms for the patient. Finally, the PRAC emphasised that the use of the HES solutions for infusion should be considered in the light of the totality of the product information including the indication, the warnings, the posology and duration of treatment and not highlight only certain contra-indications.

Proposal for a medication form and prescribers checklist were also discussed. The MAHs argued that this would ensure an informed prescription as well as support adequate use of HES solutions for infusion. However, the PRAC considered these measures raise feasibility issues in an emergency setting.

Changes to the product information with regard to the indication and contra-indications were also discussed. The main reason was comments that the current indication should be clearer. Some proposals aimed at better defining the target population eligible to HES solutions for infusion were made but this was neither supported by sufficient data, nor would have effectively led to a sufficient change of behaviour of the prescribers for the same reasons as presented above. In contrast, it is noted that the proposed revised indication from two MAHs would not have made the target population clearer and would have widened the indication for use. One MAH proposed user testing by prescribers of a revised product information. The PRAC noted this proposal, but did not agree on this measure as the readability is not a concern, and this would not address the finding that lack of adherence to the restrictions is an active choice by some centres rather than lack of awareness of the restrictions imposed in 2013 in the use of HES. Furthermore, changes of the indication or readability testing would not address the issue that the patients in the approved indication may become critically ill or septic

shortly after receiving HES solutions for infusion and these patients cannot be identified prospectively. This complicates effective risk minimisation in these patients.

Taken together, the PRAC concluded that the proposed further risk minimisation measures to ensure safe and effective use of HES solutions for infusion would not be effective or feasible in a reasonable timeframe, where important number of patients at high risk for serious harm would continue to be exposed.

A registry was also proposed but this additional pharmacovigilance activity would not prevent the administration in the population at risks.

2.3.7. Conclusion on safety

Having assessed the totality of the available data, the PRAC confirmed the previously established increased risk of mortality and renal impairment when HES solutions in infusion is used in septic patients and a greater risk of renal impairment when used in critically ill patients. The new data provided since the previous referral procedures are of insufficient sample size and/or quality to be able to draw further conclusions about safety, and does not provide any meaningful information with regards to any potential change to the safety profile of HES solutions for infusion.

The DUSs which have become available, despite a potential limitation due to possible misclassification, are considered representative of the clinical usage in the European Union and key results are reliable. The results indicate that the implemented restrictions in use since the previous referral procedures are not sufficiently adhered to. Overall non-adherence to the revised product information was reported to be high, and PRAC was particularly concerned that approximately 9% of patients exposed to HES solutions for infusion were critically ill, approximately 5-8% of patients had renal impairment and approximately 3-4% of patients had sepsis.

The non-adherence is also confirmed by the Stakeholder submissions.

The PRAC noted the overall exposure to HES solutions for infusion in the EU, estimated to about 1.5 to 2 million patients per year since 2014. In view of this exposure and the results from the two DUSs, the PRAC concluded that the estimated level of continued usage in populations where serious harm has been demonstrated raises important public health concerns, including a potentially increased mortality.

3. Expert consultation and Stakeholders inputs

The PRAC consulted an ad-hoc expert group and considered carefully views expressed during the meeting that took place on 18 December 2017. The consultation of the ad-hoc expert group was mainly focused on the place of HES solutions for infusion in the therapeutic strategy and on additional risk minimisation measures to prevent exposure for patients at risk.

The expert group agreed that there is a place for HES solutions for infusion in the authorised indication particularly in patients with hypovolaemia due to acute bleeding. One expert was of the opposite view.

Additional specific clinical settings/patient populations where HES meets an unmet clinical need were discussed and it was agreed that this can apply for example in case of plasmapheresis in acutely-ill patients, patients in shock with contraindications to other colloids (i.e. allergic to albumins) who are also refractory to volume treatment with crystalloids.

The expert group considered that alternative colloid medicinal products exist, but there are also some limitations in data about use of these products.

The PRAC noted that plasmapheresis in acutely-ill patients is not an authorised indication for HES solutions for infusion and that alternatives replacement fluids during plasmapheresis are available. The PRAC also noted that refractory shock in patients with contraindications to all other colloids is expected to be a very rare occurrence.

The PRAC duly considered the view of the majority of the experts in the meeting that HES is used in clinical practice. PRAC also noted the view of an expert concerning shared clinical experience in handling cases in a EU Member State where HES solutions for infusion are not available and where no medical need is raised.

This reflects a long-standing controversy among health-care professionals and echoes the range of stakeholder responses received in the current review.

Overall, taking into account the divergences amongst the experts, the position from the PRAC members on the national situation regarding the clinical use of these products and the stakeholders submissions, PRAC did not consider the clinical utility of these products to outweigh the risk of mortality and renal failure to the proportion of patients with critical illness or sepsis that continue to be exposed to HES solutions for infusion.

With respect to measures to minimise the risks, the expert group considered that there is a need for education of physicians with regards to current standards of fluid therapy in general. In this context, there is a need to acknowledge that fluids should be considered as any other medicines where a) an indication for its use must be followed, b) side-effects can occur so that inappropriate therapy could be harmful. The experts also expressed their opinion that part of the indication as is currently worded may be difficult to be interpreted as it was considered not entirely clear how to establish in clinical practice that “crystalloids alone are not considered sufficient“. The PRAC considered the proposal from the experts and was of the view that amendment of the indication wording from ‘crystalloids alone are not considered sufficient’ to ‘in patients treated with crystalloids’ would not have any significant impact on understanding and/or prescribing behaviour. The current wording was introduced to limit exposure to HES solutions for infusion in the treatment of hypovolaemia, due to recognition of the remaining uncertainties regarding the benefits and risks in the authorised indications. This is also reflected in the warnings (section 4.4 of the SmPC) and the obligation to conduct RCTs in elective surgery and trauma. This restriction to second-line use would be lost with the proposal from the expert group, and the proposal is therefore not supported.

The suggestion to specify the extent of blood loss to a specific volume was not endorsed by the PRAC. The difficulty to estimate blood loss in terms of a specific volume should be recognised, and there is no robust evidence to support such a specified volume of blood loss in the indication.

Regarding the proposed additional risk minimisation measures, the experts also admitted that, although supported (for instance labelling of some of the contraindications on the bag), these may not be effective if efforts from the companies are not supported by the learned societies in the field (in particular European Society of Anaesthesiology [ESA] and European Society of Intensive Care Medicine [ISICM]).

The PRAC considered these risk minimisation measures (as discussed in section 2).

4. Benefit-risk balance

The PRAC reviewed all newly available data since the last referral procedures, including results from DUSs, clinical studies, meta-analyses of clinical studies, post-marketing experience, Eudravigilance data, literature review, responses submitted by the marketing authorisation holders (MAHs) in writing and at oral explanations, other stakeholders’ submissions and views expressed by experts during an

ad-hoc experts group meeting. The evaluation of these data was put in context of the totality of the data already assessed in the previous referral procedures.

The PRAC also considered views from individual PRAC members on the benefit/risk balance of HES solutions for infusion as well as on the use of these products at the national level. These views are based on PRAC members' routine review processes and preparation. These views, along with all substantial data and information crucial for the full understanding of these views have been either shared with all the parties involved or otherwise made available in the course of the procedure.

With regards to efficacy, PRAC considered that there is no new significant information related to the approved indication. Overall, the evidence for this indication is based on studies for which the sample size and the duration of follow-up are limited. It is also noted that although the benefit has been demonstrated in terms of a volume-sparing effect, and there is some support for short-term hemodynamic effects, it remains uncertain to what extent this translates into more patient-relevant outcomes. The benefits in the approved indication therefore remain modest.

With regards to the safety data related to these products, the PRAC reviewed all available evidence since the last referral and concluded that the previous conclusions that HES solutions for infusion is associated with an increased risk of mortality and renal failure in patients with sepsis or critical illness were confirmed and that the available information, including more recently submitted clinical data, do not change the established risk in these patient populations.

Treatment of hypovolaemia should replace lost blood volume in order to restore tissue perfusion and oxygenation to ultimately prevent renal injury and death. There is a direct relation between the degree of hypovolaemia and the risk for renal injury and death. A more pronounced hypovolaemia requires a greater volume (dose) of HES solutions for infusion and is also associated with a greater risk for renal injury and death. Consequently, a direct correlation is to be expected between the indication for treatment with HES solutions for infusion, the dose of HES solutions for infusion required and with the risk for renal injury and death. It should also be noted that the ultimate benefit expected from HES solutions for infusion (and treatment of hypovolaemia in general) is a reduction of mortality and lower incidence of renal failure. The safety concerns of primary importance in this referral are increased mortality and a higher incidence of renal failure – the opposite of the benefit expected.

Amongst other data related to safety, the PRAC reviewed the results from two separate DUSs conducted to assess the effectiveness of the risk minimisation measures imposed as an outcome of the 2013 referral, and concluded that these studies despite a potential limitation of possible misclassification, are representative of the clinical usage in the European Union and that key results are reliable. The results indicate that the implemented restrictions in use are not sufficiently adhered to. Overall non-adherence to the revised product information was reported to be high, and PRAC was particularly concerned that approximately 9% of patients exposed to HES solutions for infusion were critically ill, approximately 5-8% of patients had renal impairment and approximately 3-4% of patients had sepsis.

In view of the overall exposure to HES solutions for infusion in the EU, estimated to about 1.5 to 2 million patients per year since 2014 and the reported extent of usage in patients with sepsis from the two DUSs, the estimated level of continued usage in populations where serious harm has been demonstrated raises important public health concerns, including a potentially increased mortality.

The PRAC considered other further risk minimisation measures to sufficiently minimise this exposure, including changes to the product information, direct health care professional communication, educational materials, warning on the primary container of the products, sign-in for medication form, prescription sheet/checklists. However, the available evidence shows that the non-adherence is not only due to a lack of awareness of the restrictions by prescribers but also due in some cases to

deliberate choice, rendering further communication and education unlikely to be sufficiently effective to address the risks identified. The medication form/checklists would also raise feasibility issues in an emergency setting. Proposals to amend the indications and contraindications were not considered sufficient to have a significant impact on prescriber behaviour. The PRAC also noted that the current clinical experience suggests that it is difficult to clearly separate patient populations where randomised clinical trials have shown serious harm from populations targeted by the approved indication. Patients in the approved indication may become critically ill or septic shortly after receiving HES solutions for infusion and these patients cannot be identified prospectively. This complicates effective risk minimisation in these patients.

The PRAC concluded that no additional risk minimisation measures to ensure safe and effective use of HES solutions for infusion would be effective or feasible in a reasonable timeframe, where important number of patients at high risk would continue to be exposed.

The PRAC duly considered the view of the majority of the experts in the meeting that HES is used in clinical practice. PRAC also noted the view of an expert concerning shared clinical experience in handling cases in a EU Member State where HES solutions for infusion are not available and where no medical need is raised.

This reflects a long-standing controversy among health-care professionals and echoes the range of stakeholder responses received in the current review.

Overall, taking into account the divergences amongst the experts on some important issues, the position from the PRAC members on the national situation regarding the clinical use of these products and the stakeholders submissions, PRAC did not consider the clinical utility of these products to outweigh the risk of mortality and renal failure to the proportion of patients with critical illness or sepsis that continue to be exposed to HES solutions for infusion.

In view of the seriousness of the safety issues and that the proportion of patients who are exposed to these risks in the absence of effective risk minimisation measures could have important public health consequences including a potentially increased mortality, the PRAC concluded that the benefit risk balance of hydroxyethyl starch solutions for infusion is no longer favourable and recommended the suspension of the marketing authorisations.

The PRAC noted that the clinical studies imposed following previous referrals procedures (TETHYS and PHOENICS) to characterise the efficacy and safety in trauma and elective surgery, which is currently the target population for which the product is indicated are ongoing.

A DHPC will be required to inform healthcare professionals about the suspension of the marketing authorisation.

5. Revision of PRAC recommendation

During the decision-making process, at a meeting of the Standing Committee on Medicinal Products for Human Use, some EU Member States raised new questions of technical nature which they considered had not been sufficiently addressed in the PRAC recommendation and CMDh position. In light of this, the PRAC recommendation and CMDh position were referred back to the Agency by the European Commission for further consideration of any possible unmet medical need that could result from the suspension of the marketing authorisations for the medicinal products concerned by the referral, as well as the feasibility and likely effectiveness of additional risk minimisation measures.

The PRAC discussed the above two points at its May meeting, taking into consideration information provided by the EU Member States.

5.1. PRAC discussion on unmet medical need

In response to questions raised by the PRAC, EU Member States provided information concerning the availability of alternatives treatment options (e.g. crystalloids, dextran, gelatines and/or albumin or other relevant blood products) in their territory. The EU Member States also provided answers to the question on whether the suspension of the marketing authorisations for HES solutions for infusion would result in an unmet medical need.

The PRAC took also into consideration the views from the MAHs involved in the procedure who have provided comments regarding the questions raised by the EC and the feedback provided by the EU Member States, in writing or during an Oral Explanation.

Alternatives

All EU Member States who provided information on their national situation (26), as well as Norway, have indicated that other medicinal products are available as an alternative to HES solutions for infusion. This includes crystalloids, dextran, gelatins and/or albumin. In 13 EU Member States all classes of products are authorised, in 12 EU Member States all classes of products are authorised except dextran and in one EU Member State all classes of product are authorised except gelatin. Norway states that relevant alternatives to HES solutions for infusion are available i.e. crystalloids and albumin.

Four EU Member States expressed a concern that the suspension of the marketing authorisations for the medicinal products concerned by the referral could result in an increase of use of albumin with a potential risk of shortage, and one EU Member State expressed the opinion that the suspension of marketing authorisations for HES solutions for infusion could lead to the shortage of other colloids. It is also noted that the use of HES solutions for infusion has already declined substantially from 2008 to 2017. In particular a rapid reduction of the use of HES solutions for infusion was noted in 2012-2013 without PRAC being informed about a shortage of alternatives. PRAC also noted that the 26 EU Member States who replied, and Norway, confirmed the existence of several alternatives available on their territory.

Therefore, PRAC considered that the evidence does not indicate that a critical shortage of alternative therapeutic options is likely should the marketing authorisations for HES solutions for infusion be suspended.

Potential unmet medical need

Regarding the potential for an unmet medical need, fifteen EU Member States and Norway have not identified an unmet medical need should the marketing authorisations for HES solutions for infusion be suspended. One of the fifteen EU Member States mentioned the existence of a preference among some anaesthetists to use HES solutions for infusion for priming of cardiopulmonary bypass for cardiothoracic surgery, obstetric surgery and hypotension in Caesarean section, major abdominal surgery or as a rapid blood volume expansion agent. This corresponds to some use which is neither covered by the terms of the marketing authorisation or which is not supported by appropriate evidence.

Three EU Member States expressed some uncertainty regarding a potential unmet medical need due to either a lack of information on the use of the HES solutions for infusion in their territory or conflicting messages from the learned societies at national level.

Eight EU Member States have stated that the suspension of the marketing authorisations for HES solutions for infusion would have an impact on the national clinical practices as HES solutions for infusion currently fulfill a medical need in their territory.

Indeed, three of the eight EU Member States expressed a position that HES solutions for infusion provide a rapid volume blood expansion facilitated by infusion bags being compressible and less volume being lost to extravascular space than crystalloids products. Whilst there is a theoretical benefit expected, this has not been clinically proven by adequate evidence. The PRAC also noted that European treatment guidelines reflect current clinical practice in Europe (Rossaint, R., et al., 2016). While bleeding as the primary cause of preventable mortality during the first 24 hours following trauma clearly should be acknowledged, according to these guidelines, the key management principles should be prevention of further bleeding and achievement of bleeding control, not fluid administration. Use of a restricted volume replacement strategy during initial resuscitation is encouraged. Regarding the choice of fluid, it is recommended that fluid therapy using isotonic crystalloid solutions be initiated in the hypotensive bleeding trauma patient. The guidelines further recommend that the use of colloids be restricted due to the adverse effects on haemostasis.

One of the eight EU Member States mentioned that the use of HES solutions for infusion could be relevant for patients at increased risk for fluid overload and tissue oedema in view of claimed volume-sparing effect. However, such population is not defined and supported by relevant evidence. This could include patients with renal failure or patients with pathological capillary leakage (e.g. as due to inflammatory activation in sepsis or critical illness), however renal failure and critical illness/sepsis are contraindications for HES solutions for infusion. Furthermore, whilst data provide some support on a volume-sparing effect of HES solutions for infusion, there is insufficient data to support that this effect translates into benefit in terms of more clinically meaningful outcomes.

Three of the eight EU Member States mentioned that HES solutions for infusion could be used as a second-line treatment for patients refractory to crystalloids. Use of HES solutions for infusion for population refractory to crystalloids that would have a more favorable outcome with HES solutions for infusion is not supported by relevant evidence. The restriction of indication in the previous referral in 2013 was intended to limit the exposure to HES solutions for infusion in view of modest benefit and the uncertainty on the risks in the authorised indication. There is no clear definition of a population refractory to crystalloids, and no data demonstrating efficacy and safety for HES solutions for infusion in such a putative population.

One of the 8 EU Member States mentioned that in their territory HES solutions for infusion are used in major abdominal surgery to lower the post-operative morbidity. The same EU Member State also claimed that the use of HES solutions for infusion as a colloid provide a benefit in terms of volume-sparing effect and reduce the need for blood transfusion. The PRAC assessed the available studies in abdominal surgery (see section 2.2.1) and concluded that they provide some support for the expected volume-sparing effect of HES solutions for infusion but there is no convincing support that this effect translates into benefit in more clinically meaningful outcomes. Regarding the benefit of colloids in terms of volume-sparing effects, whilst it is established that HES solutions for infusion have a volume-sparing effect, it was not demonstrated that it translates into meaningful clinical outcomes. Finally, the claim of reduced need for blood transfusion is not supported by data. On the contrary, in cardiothoracic surgery, the use of HES solution for infusion was associated with increased bleeding and increased need for blood products.

One of the 8 EU Member States reported some medical use of HES solutions for infusion in the priming of cardiopulmonary bypass for cardiothoracic surgery. This use is not covered by the terms of the marketing authorisation and is not recommended due to the risk of excess bleeding. The PRAC assessed the available data in cardiac surgery (see section 2.2.4) which confirms a volume-sparing effect but fails to provide new evidence for patient benefit in terms of other down-stream outcomes. The safety data suggests an adverse effect on coagulation and bleeding in cardiac surgery associated with the administration of HES solutions for infusion, and consequently confirms the warning in the

product information introduced in 2013 that the use of HES solutions for infusion is not recommended in patients undergoing open heart surgery in association with cardiopulmonary bypass due to the risk of excess bleeding.

One of the 8 EU Member States highlighted cases where a patient could be allergic to albumin and another Member State highlighted that some patients may refuse to be treated with products of animal origin. The PRAC noted that hypersensitivity reactions to albumin occur infrequently (*Gales et al, 1993*). In this rare situation, crystalloids, other synthetic colloids or other relevant blood products remain treatment alternatives. The PRAC also noted that in situations where a patient refuses to be treated by products of animal origin, crystalloids and other synthetic colloids remain alternative treatments.

Finally 6 of the 8 EU Member States considered that the alternatives to HES solutions for infusion do not offer a better benefit-risk balance and consider there is a place in the therapeutic strategy for HES solutions for infusion. It is noted by the PRAC that this comparative claim of a better benefit/risk balance for HES solutions for infusions is not supported by relevant data. One of these MSs referred this claim to the following studies, which PRAC reviewed:

- One study referenced in one EU Member State's comment is a systematic review and meta-analysis (*Ripollés et al, 2016*) of intraoperative goal-directed hemodynamic therapy in non-cardiac surgery. The study does not provide data on comparisons between different fluids, and consequently not on HES solutions for infusion vs. other fluids. That goal directed hemodynamic therapy should be able to reduce harm associated specifically with HES solutions for infusion, and not only fluids in general, remains speculative without support by this study or other data.
- This is an observational study of 4545 adult patients from 65 German intensive care units (*Ertmer et al, 2018*), receiving IV fluid therapy during the period June 2010 to May 2011. The PRAC considered that no conclusions could be drawn for the following reasons:
  - 24% of patients in the crystalloid group, compared to only 2% in the colloid+crystalloid group, were removed from analysis due to missing information. Two (2) percent of patients eligible for multivariable analysis in the crystalloid group, compared to 16% in the colloid+crystalloid group, had no 90-day follow-up. There is therefore a substantial risk for a selection bias that affects the outcome. This problem has not been addressed in the analyses.
  - Survival status was determined by contacting survivors by postal mail, and if needed telephone and registration office. It is noted that unknown status for 90-day follow-up was associated with lower SAPS II and APACHE II scores on admission. This is, at least partly, addressed by sensitivity analysis.
  - In the multivariable analysis the authors performs what they term a "full model" analysis including covariates derived during ICU stay (AUC of SOFA score until event or end of stay, cumulative volume of red blood cell products, cumulative volume of other blood products, cumulative fluid balance, application of vasopressor equivalent >0.6 mg/h and daily crystalloid infusion). Adjustments for variables derived from the observation period must be made cautiously and with specific analytical approaches (such as Marginal Structural Models). Several of the variables adjusted for are likely in the causal pathway and some may even be colliders (potentially introducing a Berksonian bias). The increased risk for serious harm associated with colloid vs crystalloid use seen when only adjusting for baseline factors, is removed using the "full models". The 'full model adjusted' results must therefore be interpreted with caution.

- A systematic review and meta-analysis of colloids versus crystalloids in critically ill, trauma and surgical patients (*Qureshi et al, 2015*). The result in patients undergoing general surgical operations suggests increased mortality compared with crystalloid administration (OR 2.61, 95% CI 0.59 to 11.49), but the estimate has very poor precision. Lack of statistical significance should not be used to conclude the absence of an association.

The PRAC carefully considered all the information provided in relation to a potential unmet medical need at national level should the marketing authorisations for HES solutions for infusion be suspended. PRAC considered that despite arguments raised by some EU Member States, the potential for unmet medical need is not established. Besides, most of the arguments refer to the use of HES solutions for infusion outside the terms of the MA or to claimed benefits that are not clinically significant or supported by robust data.

The PRAC also considered the data submitted by the MAH, regarding spontaneously reported suspected adverse reactions from Eudravigilance and the UK yellow card system to support the claim that alternative treatment options do not provide more adequate/satisfactory treatment and do not lead to risk reduction. The PRAC concluded that spontaneous reporting data, from Eudravigilance or the UK Yellow card system are inadequate to establish reporting rates, or to compare the safety profiles across products. Additionally, the Yellow Card data presented for 'standard solutions' (i.e. crystalloids) concern all the products with sodium chloride as an active ingredient, including crystalloid solutions but also other products such as peritoneal dialysis fluids, parenteral nutrition and eye drops. Overall the PRAC considered that data submitted does not allow any conclusion with regards to the specific serious safety concerns in focus for this referral procedure, or the overall safety profiles of HES solutions for infusion and alternative products.

The PRAC noted the recommendations from some Member States that the ongoing clinical studies imposed following previous referral procedures (TETHYS and PHOENICS) to characterise the efficacy and safety in trauma and elective surgery, which is currently the target population for which the product is indicated, should be awaited before further reviewing the benefit/risk balance. The results from these RCTs, while being of a general scientific interest for the approved indications, cannot address the problem of harm from use in patients with critical illness and/or sepsis, or provide data on benefit that can outweigh such harm. Namely, data in terms of benefit and impact on mortality and renal failure cannot be expected from these trials, due to the unfeasibility for such objectives, as clarified in the CHMP scientific advice provided for the design of these trials.

5.2. PRAC discussion on additional risk minimisation measures

The PRAC also discussed whether risk minimisation measures would be feasible and effective in ensuring adherence to the product information and specifically to contraindications for use of HES solutions for infusion, including in patients with critical illness and/or sepsis.

The PRAC carefully reviewed all the elements provided by the EU Member States and the MAHs on the adequacy of risk minimisation measures to address the use of HES solutions for infusion in septic and critically ill patients. The PRAC took also into consideration all data provided in the initial phase of the procedure, as well as the measures proposed by the ad hoc expert group consulted on December 2017.

Warning label on the immediate packaging on the contraindications in septic or critically ill patients

This option was discussed by PRAC at its January 2018 plenary meeting and considered again by PRAC at its May 2018 plenary meeting.

From the contributions from EU Member States, whilst a few EU Member states was of the view that this measure would have some effect, the vast majority expressed significant concerns about its effectiveness as the prescriber would not usually be the person who administers the solution and would typically not see the warning label when prescribing. It was also considered not appropriate to highlight only a limited number of the contraindications on the packaging, which could potentially detract from the consideration given to other important contraindications.

The PRAC agreed with the issues mentioned by the majority of the EU Member States and confirmed its previous position that this measure is unlikely to be effective.

Mandatory signing of a form mentioning the authorised condition of use by healthcare professionals for each patient receiving HES solutions for infusion

This option was discussed by PRAC at its January 2018 plenary meeting and considered again by PRAC at its May 2018 plenary meeting.

The PRAC took into consideration the responses from the EU Member States and the proposals by the MAHs.

Whilst the objective of this measure is to prompt the healthcare professional to respect the authorised conditions of use for HES solutions for infusion to ensure that the medicinal product is administered in compliance with the terms of the marketing authorisation, 22 Member States expressed concerns about its feasibility.

The PRAC acknowledged a potential effect however confirmed feasibility concerns. Indeed, requesting healthcare professionals to fill administrative forms before the use of the product in an emergency setting would not be feasible. Indeed it would interfere with timely treatment of patients in urgent need of medical care

An alternative proposal was to implement a form signed retrospectively after the administration of HES solutions for infusion to be added to the patient's medical records. However, PRAC considered that documenting use of HES solutions for infusion after their administration would not help to ensure HES solutions for infusion are only administered to eligible patients, i.e. in line with the terms of the marketing authorisation. It was also questioned whether this measure would be sustainable in clinical practice.

The PRAC therefore confirmed its previous position that a mandatory form to be signed by the healthcare professionals would not be an adequate risk minimisation measure.

Restricting access to HES containing solutions, e.g. to specific hospital departments and/or prescribers

The PRAC evaluated different options regarding limiting the access of HES solutions for infusions to particular hospitals, hospital departments or physicians. In particular, the PRAC reviewed the proposal from the MAHs to condition the distribution of HES solutions for infusion to hospitals and physicians who have followed a specific training.

The PRAC considered the responses provided by the EU Member States who all clarified that it would raise feasibility issues.

The main feasibility concern raised relates to the criteria for the selection of prescribers / departments / hospitals that could use appropriately HES solutions for infusion. Indeed, considering the characteristics of HES solutions for infusion, and particularly the type of their distribution and indication, these products are not currently limited to specific prescribers, hospital departments or hospitals.

One proposal as a selection criterion for a restricted access is to limit to physicians that have been dispenses a specific training dispensed. However it is doubtful that this would be sufficiently effective to impact on the prescriber's behaviour as the lack of adherence to key restrictions is not solely due to lack of awareness from the prescribers. Concerns on the feasibility were also raised on the implementation of such condition, in particular on the handling of accreditation of physicians throughout their career.

With regards to other selection criteria such as limiting to certain hospital departments, patients within the authorised indications and patients with contraindications for the use of HES solutions for infusion would often be present in the same hospital departments. For these reasons restricting distribution to specific hospital departments was not considered feasible or likely to be effective as a risk minimisation measure.

Lastly, it was noted that products for fluid resuscitation, such as HES solutions for infusion, are generally supplied in bulk from hospital pharmacies to relevant clinical areas, in order that they are available for immediate use when they are prescribed. As such it is not feasible to restrict access at a pharmacy level in the same way as for products which are dispensed in response to a prescription for a specific patient.

Therefore in the absence of effective and appropriate selection criteria for restricted access for specific hospital departments and/or prescribers, this measure was deemed not feasible by PRAC.

Other additional risk minimisation measures

The PRAC discussed the following risk minimisation measures:

Update of the product information

Changes to the product information with regard to the indication and contra-indications were discussed by PRAC at its January plenary meeting and further considered in its May plenary meeting.

The PRAC reviewed in particular a proposal to simplify the indication proposed by the MAHs and the inclusion of a box warning to further emphasise relevant information.

The PRAC considered that the changes proposed are neither supported by sufficient data, nor would effectively led to change of behaviour of the prescribers as the lack of adherence to the product information is not solely due to the lack of awareness of, or clarity in, the wording of the indication and/or contraindications.

Communication

DHCP or other proactive communication

The PRAC discussed this measure in its January plenary meeting and considered it again at its May plenary meeting.

Ad hoc communication to healthcare professionals, for instance in the form of a DHPC, would have some utility to highlight to healthcare professionals the authorised conditions of use for HES solutions for infusion and could be periodically repeated. However, such communication was not sufficiently effective after 2013 in view of the substantial rate of non-compliance to key restrictions shown in from the DUSs. There was no support that repeating the same type of communication would be effective, leading to the behavioural change in clinical practice required, noting that such non-compliance is not solely due to lack of awareness from the prescribers.

In view of the above, the PRAC therefore confirmed its previous position that this measure would not be sufficiently effective to minimise the risk.

Educational programme including trainings

The PRAC discussed this measure in its January plenary meeting and considered it again at its May plenary meeting. The PRAC carefully considered the proposal from the MAHs and the contribution from Member States on this measure.

In view of the significant rate of non-compliance and that the lack of adherence to key restrictions is not solely due to lack of awareness from the prescribers, it is doubtful that further communication and educational measures would have a sufficient impact on the prescribers' behaviour.

In addition, reaching all the prescribers is challenging. The proposal for incentives such as 'continuing medical education credit' is acknowledged however would not ensure full participation as it would still be on a voluntary basis. It was noted also that such systems could not be implemented in all Member States.

The proposals to involve the learned societies is noted, but concerns are raised regarding participation of all relevant learned societies and agreement on common key messages for risk minimisation measures, in reasonable timeframe, taking into account divergent national clinical guidelines and divergent views expressed by learned societies during the procedure and following the PRAC recommendation from January 2018.

Combination of risk minimisation measures

The PRAC assessed the feasibility and likely effectiveness of each risk minimisation measure and concluded that each measure either alone or in combination would not be sufficiently effective or feasible or could not be implemented in a reasonable timeframe.

5.3. PRAC discussion on additional information received since January, 2018 PRAC recommendation

Since the PRAC discussion in January 2018, correspondences from various stakeholders have also been received by the EMA or have been made publicly available. Different views were expressed on the benefits and risks of the HES solutions for infusion. Overall, PRAC considered that no new elements were identified which impacted the previous PRAC assessment or contributed to the responses to the questions raised by the EC.

One MAH submitted the following studies:

- A propensity score matched, controlled observational study Pagel et al. (2018) that compared Ringer's acetate combined with HES 130/0.4 (RA-HES) vs. Ringer's acetate alone (RA) in 9085 patients undergoing various surgical procedures regarding the incidence of postoperative acute kidney failure (AKF) in perioperative settings. In this publication, the authors concluded that there was no association between intraoperative HES therapy and postoperative kidney failure in a mixed cohort of elective surgical patients.

  This is an observational study, using propensity score matching, aimed at comparing acute post-interventional adverse events compared to Ringer's acetate alone in a perioperative setting. The PRAC considered that there are several methodological aspects that limit the value of the study, including uncertainty from the presentation of length of follow up and duration of postoperative serum creatinine monitoring that was not controlled. The results suggest that HES 130/0.4 did not induce an increased frequency of acute kidney failure, or mortality, or need for intensive care. On the other hand, perioperative blood loss was about doubled in the HES group compared with crystalloids, and treatment with HES 130/0.4 was associated with a prolonged length of hospital stay. The PRAC considered that given the

methodological aspects of this study, the data are of limited value, and do not affect the previous assessment of benefits and risks of HES solutions for infusion. The results do not address the major concern of continued use of HES in patient populations where serious harm has been demonstrated.

- Another study was also presented. Kammerer et al. (2018) compared renal function in 100 patients undergoing cystectomy (i.e. elective surgery in non-critically ill patients), who received balanced 6% HES 130/0.4 or 5% albumin. The authors concluded that with respect to renal function and kidney injury, this study indicates that albumin 5% and balanced 6% HES have comparable safety profiles in non-critically ill patients undergoing major surgery.

Regarding the study by Kammerer et al. 2018, the PRAC noted the results, namely no direct difference between albumin and HES in the studied setting, in a small study. The PRAC noted that studies identifying harm have used crystalloids as comparators. The PRAC considers that these data do not provide substantial new evidence in relation to benefits and risks of HES solutions for infusion in the approved indication. The results do not address the major concern of continued use of HES solutions for infusion, compared to crystalloids, in patient populations where serious harm has been demonstrated. Regarding newly published data referred to above, no new evidence that changes previous scientific conclusions from the PRAC have been made available.

5.4. Impact of the new information on the previously concluded benefit-risk balance

The PRAC has considered all elements expressed in relation to the impact of a suspension of HES solutions for infusion on a potential unmet medical need at national level, including comments submitted by the MAHs in writing and at oral explanations, responses from Member States and other stakeholders' views.

With regard to the impact on a suspension of the marketing authorisations for HES solutions for infusion, fifteen EU Member States and Norway mentioned that no unmet medical need is expected in case of suspension of the marketing authorisations for HES solutions for infusion.

The PRAC carefully considered all the information provided in relation to a potential unmet medical need at national level should the marketing authorisations for HES solutions for infusion be suspended. Eight EU Member States have mentioned that a suspension of the marketing authorisations for HES solutions for infusion would have an impact in the national clinical practice as HES fulfils currently a medical need in their territory. PRAC considered that despite arguments raised by some member states, the potential for unmet medical need is not established. Most of the arguments refer to the use of HES solutions for infusion outside the terms of the MA or to claimed benefits that are not clinically significant or supported by robust data.

The PRAC concluded that the claimed clinical utility for these products does not outweigh the risk of mortality and renal failure to the proportion of patients with critical illness or sepsis that continues to be exposed to HES solutions for infusion.

The PRAC have also further considered for the feasibility and likely effectiveness of risk minimisation measures.

The PRAC considered further risk minimisation measures which could potentially sufficiently minimise this exposure, including restricted access / distribution to hospitals and physicians, changes to the product information, direct health care professional communication, educational materials to be distributed in cooperation with some learned societies, warning on the primary container of the

products, medication form and follow-up questionnaire. However, the available evidence shows that the non-adherence is not only due to a lack of awareness of the restrictions by prescribers but also due to deliberate choice, rendering further communication and education unlikely to be sufficiently effective to address the risks identified. A restricted distribution system to accredited hospitals or physicians would raise serious feasibility concerns and would be unlikely to be effective considering the particular type of distribution and usage of HES solutions for infusion. A medication form to be filled before administration would also raise feasibility issues in an emergency setting. A follow-up questionnaire to be filled after administration would not be effective in minimising the risk. Proposals to amend the indications and contraindications were not considered have a sufficient impact on prescriber behaviour and were not supported by appropriate scientific evidence.

In conclusion, no risk minimisation measures or combination of measures have been identified which would be sufficiently effective or feasible to implement in a reasonable timeframe, when an important number of patients at high risk for serious harm, would continue to be exposed.

In the light of the above information, the PRAC confirmed at its May 2018 plenary meeting, its previous scientific conclusions that the benefit risk of HES solutions for infusion is negative and recommended to suspend the marketing authorisations of these medicinal products.

6. Condition for lifting the suspension of the marketing authorisations

For the suspension to be lifted, the Marketing Authorisation Holder(s) shall provide reliable and convincing evidence on a favourable benefit risk balance in a well-defined population, with feasible and effective measures to adequately minimise exposure of patients at an increased risk of serious harm.

7. Revised grounds for PRAC Recommendation

Whereas,

- The Pharmacovigilance Risk Assessment Committee (PRAC) considered the procedure under Article 107i of Directive 2001/83/EC, for hydroxyethyl starch (HES) solution for infusion (see Annex I).
- The PRAC reviewed all newly available data, including results from Drug Utilisation Studies (DUS), clinical studies, meta-analyses of clinical studies, post-marketing experience, Eudravigilance data, literature review, responses submitted by the marketing authorisation holders (MAHs) in writing and at oral explanations, stakeholders’ submissions and views expressed by experts during an ad-hoc experts meeting. The PRAC also reviewed responses from EU Member States in relation to the potential unmet medical need and proposals for additional risk minimisation measures.
- With regards to the efficacy, PRAC considered that there is no new significant information related to the approved indication. Overall, the evidence for this indication is based on studies for which the sample size and the duration of follow-up are limited. Is it also noted that although the benefit has been demonstrated in terms of a volume-sparing effect, and there is some support for short-term hemodynamic effects, it remains uncertain to what extent this translates into more patient-relevant outcomes. The benefits in the approved indication therefore remain modest.
- With regards to the two separate DUSs conducted to assess the effectiveness of the risk minimisation measures imposed as an outcome of the 2013 referral, PRAC concluded that

these studies despite limitations due to possible misclassification representative of the clinical usage in the European Union and that key results are reliable. The results indicate that the implemented restrictions in use are not adhered to. Overall non-adherence to the revised product information was reported to be high, and PRAC was particularly concerned that approximately 9% of patients exposed to HES solutions for infusion were critically ill, approximately 5-8% of patients had renal impairment and approximately 3-4% of patients had sepsis.

- The PRAC conclusions of previous reviews under Article 31 of Directive and Article 107i of Directive 2001/83/EC were that HES solutions for infusion are associated with an increased risk of mortality and renal failure in patients with sepsis or critical illness. PRAC confirmed that the available information, including more recent submitted clinical data, do not change the established risk of increased mortality and renal failure related to the use of HES solutions for infusion in these patients. The new data provided does not change the conclusions from the previous 2013 referral that the benefits of HES solutions for infusion do not outweigh the serious risks in patients with sepsis or critical illness.
- The PRAC also noted the overall exposure to HES solutions for infusion in the EU, estimated to about 1.5 to 2 million patients per year since 2014. In view of this exposure and the results from the two DUSs, the PRAC concluded that the estimated level of continued usage in populations where serious harm has been demonstrated raises important public health concerns, including a potentially increased mortality.
- The PRAC further acknowledged that the current clinical experience suggests that it is difficult to clearly separate patient populations where randomised clinical trials have shown serious harm from populations targeted by the approved indication. Patients in the approved indication may become critically ill or septic shortly after receiving HES solutions for infusion and these patients cannot be identified prospectively. This complicates effective risk minimisation in these patients.
- Furthermore, the PRAC considered options for measures to further mitigate these risks, including changes to the product information, direct health care professional communication, educational materials, warning on the primary container of the products, sign-in for medication form, prescription sheet/checklists, restricted access and distribution system to accredited hospitals/physicians. However, the available evidence shows that the non-adherence is not only due to a lack of awareness of the restrictions by prescribers, rendering further communication and education unlikely to be sufficiently effective. The medication form/checklists would also raise feasibility issues in an emergency setting, and implementation of a restricted access/distribution program is unlikely to be feasible and sufficiently effective across EU Member States considering the particular type of distribution and usage of HES solutions for infusion and some national limitations. The PRAC concluded that no additional risk minimisation measure or combination of risk minimisation measures, to sufficiently ensure safe and effective use of HES solutions for infusion could be identified.

In view of the above, the PRAC concluded that pursuant to Article 116 of Directive 2001/83/EC the risks related to the use of HES outweigh their benefits and thus the benefit-risk balance of HES solutions for infusion is no longer favourable.

Therefore, the PRAC recommends the suspension of the marketing authorisations for all medicinal products referred to in Annex I.

For lifting the suspension, the MAHs should provide reliable and convincing evidence on a favourable benefit risk balance in a well-defined population, with feasible and effective measures to adequately minimise exposure of patients at an increased risk of serious harm.

References

ACCM/SCCM, American College of Chest Physicians/Society of Critical Care Medicine Consensus Conference: definitions for sepsis and organ failure and guidelines for the use of innovative therapies in sepsis. Crit Care Med, 1992. 20(6): p. 864-74.

Akkucuk, F.G., et al., The effect of HES (130/0.4) usage as the priming solution on renal function in children undergoing cardiac surgery. Ren Fail, 2013. 35(2): p. 210-5.

Albrecht, Frederic W.; Glas, Michael; Rensing, Hauke; Kindgen-Milles, Detlef; Volk, Thomas; Mathes, Alexander M. (2016): A change of colloid from hydroxyethyl starch to gelatin does not reduce rate of renal failure or mortality in surgical critical care patients. Results of a retrospective cohort study. In: Journal of critical care 36, S. 160–165. DOI: 10.1016/j.jcrc.2016.07.005.

Alimian, M., et al., Comparison of hydroxyethyl starch 6% and crystalloids for preloading in elective caesarean section under spinal anesthesia. Med Arch, 2014. 68(4): p. 279-81.

Amin, S.M. and S.M. Fathy, Effect of preoperative hypervolemic hemodilution with hydroxyethyl starch (130/0.4) on hemodynamics, blood loss and renal function after laparoscopic gastric bypass surgery. Egyptian Journal of Anaesthesia, 2016(32): p. 77-81.

Annane, D., et al., Effects of fluid resuscitation with colloids vs crystalloids on mortality in critically ill patients presenting with hypovolaemic shock: the CRISTAL randomised trial. JAMA, 2013. 310(17): p. 1808-17.

Annane, D.; Siami, S.; Jaber, S.; Martin, C.; Elatrous, S.; Declere, A. D. et al. (2013): Effects of Fluid Resuscitation With Colloids vs Crystalloids on Mortality in Critically Ill Patients Presenting With Hypovolaemic Shock: The CRISTAL Randomised Trial. In: JAMA., S. 10.

Anthon, CT.; Muller, RB.; Haase, N.; Hjortrup, PB.; Moller, K.; Lange, T. et al. (2017): Effects of hydroxyethyl starch 130/0.42 vs. Ringer's acetate on cytokine levels in severe sepsis. In: Acta anaesthesiologica Scandinavica 61 (8), S. 904–913. DOI: 10.1111/aas.12929.

Arora, P., et al., Fluid Administration Before Caesarean Delivery: Does Type and Timing Matter? J Clin Diagn Res, 2015. 9(6): p. UC01-4.

Bellomo, R., et al., Acute renal failure - definition, outcome measures, animal models, fluid therapy and information technology needs: the Second International Consensus Conference of the Acute Dialysis Quality Initiative (ADQI) Group. Crit Care, 2004. 8(4): p. R204-12.

Benes, J., et al., The effects of goal-directed fluid therapy based on dynamic parameters on post-surgical outcome: a meta-analysis of randomised controlled trials. Crit Care, 2014. 18(5): p. 584.

Bennasr, L., et al., [Prevention of hypotension during spinal anesthesia for elective caesarean section: coloading with HAE 130/0.4 vs normal saline solution]. Ann Fr Anesth Reanim, 2014. 33(12): p. 643-7.

Boom, C.E., et al., Effect of hyperosmolar sodium lactate infusion on haemodynamic status and fluid balance compared with hydroxyethyl starch 6% during the cardiac surgery. Indian J Anaesth, 2013. 57(6): p. 576-82.

Brunkhorst, F.M., et al., Intensive insulin therapy and pentastarch resuscitation in severe sepsis. N Engl J Med, 2008. 358(2): p. 125-39.

Carli, P., et al., Remplissage vasculaire prehospitalier en taumatologie: hesteril 6% versus plasmion. Jeur, 2000. 13(1-2): p. 101-105.

Cecconi, M., et al., Clinical review: Goal-directed therapy-what is the evidence in surgical patients? The effect on different risk groups. Crit Care, 2013. 17(2): p. 209.

Chooi, C., et al., Techniques for preventing hypotension during spinal anaesthesia for caesarean section. Cochrane Database Syst Rev, 2017. 8: p. CD002251.

Claus, R.A., M. Sossdorf, and C. Hartog, The effects of hydroxyethyl starch on cultured renal epithelial cells. Anesth Analg, 2010. 110(2): p. 300-1.

Datzmann, T., et al., Influence of 6% HES 130/0.4 vs. crystalloid solution on structural renal damage markers after coronary artery bypass grafting – a post hoc subgroup analysis of a prospective trial. J Cardiothorac Vasc Anesth, 2017.

Doshi, P., Data too important to share: do those who control the data control the message? BMJ, 2016. 352: p. i1027.

El-Fandy, G.G., et al., Fluid optimization with hydroxyethyl starch 130/0.4 compared with modified fluid gelatin guided by esophageal Doppler during major abdominal surgeries. J Egypt Soc Parasitol, 2014. 44(1): p. 151-60.

Eriksson et al. 2015 J Trauma Acute Care Surg 2015; 79(3):407-412

Eriksson, M.; Brattstrom, O.; Martensson, J.; Larsson, E.; Oldner, A. (2015): Acute kidney injury following severe trauma: Risk factors and long-term outcome. In: The journal of trauma and acute care surgery 79 (3), S. 407–412. DOI: 10.1097/TA.0000000000000727.

Feldheiser, A., et al., Balanced crystalloid compared with balanced colloid solution using a goal-directed haemodynamic algorithm. Br J Anaesth, 2013. 110(2): p. 231-40.

Gedeborg R, Furebring M, Michaëlsson K. Diagnosis-dependent misclassification of infections using administrative data variably affected incidence and mortality estimates in ICU patients. J Clin Epidemiol. 2007 Feb;60(2):155-62.)

Ghanei et al. 2016 The IIOAB Journal 2016; 7: 323-328

Ghodraty, M.R., et al., Crystalloid versus colloid fluids for reduction of postoperative ileus after abdominal operation under combined general and epidural anesthesia. Surgery, 2017. 162(5): p. 1055-1062.

Guidet, B.; Martinet, O.; Boulain, T.; Philippart, F.; Poussel, J. F.; Maizel, J. et al. (2012): Assessment of hemodynamic efficacy and safety of 6% hydroxyethylstarch 130/0.4 versus 0.9% NaCl fluid replacement in patients with severe sepsis: The CRYSTMAS study. In: Critical care (London, England) 16 (3), R94. DOI: 10.1186/cc11358.

Gulland, A., EU urges full transparency in science research. BMJ, 2016. 353: p. i2032.

Guo, SB.; Chen, YX.; Yu, XZ. (2017): Clinical Characteristics and Current Interventions in Shock Patients in Chinese Emergency Departments: A Multicentre Prospective Cohort Study. In: Chinese medical journal 130 (10), S. 1146–1154. DOI: 10.4103/0366-6999.205862.

Gurbuz, H.A., et al., Hydroxyethyl starch 6%, 130/0.4 vs. a balanced crystalloid solution in cardiopulmonary bypass priming: a randomised, prospective study. J Cardiothorac Surg, 2013. 8: p. 71.

Haase, N.; Perner, A.; Hennings, L. I.; Siegemund, M.; Lauridsen, B.; Wetterslev, M.; Wetterslev, J. (2013a): Hydroxyethyl starch 130/0.38-0.45 versus crystalloid or albumin in patients with sepsis:

systematic review with meta-analysis and trial sequential analysis. In: BMJ. 346:f839. doi: 10.1136/bmj.f839, f839.

Haase, N.; Wetterslev, J.; Winkel, P.; Perner, A. (2013b): Bleeding and risk of death with hydroxyethyl starch in severe sepsis: post hoc analyses of a randomised clinical trial. In: Intensive care medicine 39 (12), S. 2126–2134. DOI: 10.1007/s00134-013-3111-9.

Hamaji, A., et al., Volume Replacement Therapy during Hip Arthroplasty using Hydroxyethyl Starch (130/0.4) Compared to Lactated Ringer Decreases Allogeneic Blood Transfusion and Postoperative Infection. Rev Bras Anestesiol, 2013. 63(1): p. 27-44.

Hans, G.A., et al., The effect of intraoperative 6% balanced hydroxyethyl starch (130/0.4) during cardiac surgery on transfusion requirements. J Cardiothorac Vasc Anesth, 2015. 29(2): p. 328-32.

Hartog, C.S., M. Kohl, and K. Reinhart, A systematic review of third-generation hydroxyethyl starch (HES 130/0.4) in resuscitation: safety not adequately addressed. Anesth Analg, 2011. 112(3): p. 635-45.

He, B.; Xu, B.; Xu, X.; Li, L.; Ren, R.; Chen, Z. et al. (2015): Hydroxyethyl starch versus other fluids for non-septic patients in the intensive care unit: a meta-analysis of randomised controlled trials. In: Crit Care. %19;19 (1), S. 92–833. DOI: 10.1186/s13054-015-0833-9.

Hjortrup, P. B.; Haase, N.; Wetterslev, J.; Perner, A. (2016): Associations of Hospital and Patient Characteristics with Fluid Resuscitation Volumes in Patients with Severe Sepsis: Post Hoc Analyses of Data from a Multicentre Randomised Clinical Trial. In: PLoS.One. 11 (5), e0155767. DOI: 10.1371/journal.pone.0155767.

Hung, M.H., et al., New 6% hydroxyethyl starch 130/0.4 does not increase blood loss during major abdominal surgery - a randomised, controlled trial. J Formos Med Assoc, 2014. 113(7): p. 429-35.

James, M.F., et al., Resuscitation with hydroxyethyl starch improves renal function and lactate clearance in penetrating trauma in a randomised controlled study: the FIRST trial (Fluids in Resuscitation of Severe Trauma). Br J Anaesth, 2011. 107(5): p. 693-702.

Joosten, A., et al., Crystalloid versus Colloid for Intraoperative Goal-directed Fluid Therapy Using a Closed-loop System: A Randomised, Double-blinded, Controlled Trial in Major Abdominal Surgery. Anesthesiology, 2017.

Joosten, A., et al., Impact of balanced tetrastarch raw material on perioperative blood loss: a randomised double blind controlled trial. Br J Anaesth, 2016. 117(4): p. 442-449.

Juri, T., et al., Hydroxyethyl starch 130/0.4 versus crystalloid co-loading during general anesthesia induction: a randomised controlled trial. J Anesth, 2017. 31(6): p. 878-884.

Kajdi, M.E., et al., Anaesthesia in patients undergoing cytoreductive surgery with hyperthermic intraperitoneal chemotherapy: retrospective analysis of a single centre three-year experience. World J Surg Oncol, 2014. 12: p. 136.

Kammerer, T., et al., Comparison of 6% hydroxyethyl starch and 5% albumin for volume replacement therapy in patients undergoing cystectomy (CHART): study protocol for a randomised controlled trial. Trials, 2015. 16: p. 384.

Kancir, A.S., et al., Lack of nephrotoxicity by 6% hydroxyethyl starch 130/0.4 during hip arthroplasty: a randomised controlled trial. Anesthesiology, 2014. 121(5): p. 948-58.

Kancir, A.S., et al., The effect of 6% hydroxyethyl starch 130/0.4 on renal function, arterial blood pressure, and vasoactive hormones during radical prostatectomy: a randomised controlled trial. Anesth Analg, 2015. 120(3): p. 608-18.

Kanda et al. 2015 Ther Clin Risk Manag 2015; 11:1619-25

Kanda, H., et al., Effect of fluid loading with normal saline and 6% hydroxyethyl starch on stroke volume variability and left ventricular volume. Int J Gen Med, 2015. 8: p. 319-24.

Kastrup, M., et al., Clinical impact of the publication of S3 guidelines for intensive care in cardiac surgery patients in Germany: results from a postal survey. Acta Anaesthesiol Scand, 2013. 57(2): p. 206-13.

Kim, T.K., et al., Microvascular reactivity and endothelial glycocalyx degradation when administering hydroxyethyl starch or crystalloid during off-pump coronary artery bypass graft surgery: a randomised trial. Anaesthesia, 2017. 72(2): p. 204-213.

Kimenai, D.M., et al., Effect of the colloids gelatin and HES 130/0.4 on blood coagulation in cardiac surgery patients: a randomised controlled trial. Perfusion, 2013. 28(6): p. 512-9.

Knaus, W.A., et al., APACHE II: a severity of disease classification system. Crit Care Med, 1985. 13(10): p. 818-29.

Knaus, W.A., et al., The APACHE III prognostic system. Risk prediction of hospital mortality for critically ill hospitalized adults. Chest, 1991. 100(6): p. 1619-36.

Lagny, M.G., et al., Hydroxyethyl Starch 130/0.4 and the Risk of Acute Kidney Injury After Cardiopulmonary Bypass: A Single-Centre Retrospective Study. J Cardiothorac Vasc Anesth, 2016. 30(4): p. 869-75.

Leberle, R., et al., Association of high volumes of hydroxyethyl starch with acute kidney injury in elderly trauma patients. Injury, 2015. 46(1): p. 105-9.

Li, S.; Yu, C.; Li, Y.; Zhang, R.; Ma, Y.; Hou, Q. et al. (2014): Hydroxyethyl Starch And Hospitalized Mortality In Icu Patients With Diabetes. Database Study From A Chinese Tertiary Hospital. In: Value in health : the journal of the International Society for Pharmacoeconomics and Outcomes Research 17 (7), A742-3. DOI: 10.1016/j.jval.2014.08.150.

Li, Y., et al., Ringer's lactate, but not hydroxyethyl starch, prolongs the food intolerance time after major abdominal surgery; an open-labelled clinical trial. BMC Anesthesiol, 2015. 15: p. 72.

Lim et al. 2016 Congress abstract Anaesthesia 2016, 71, (Suppl. 2) 8–88

Lyu, J.; Li, T.; Liu, F.; An, Y. (2015): [The influence of hydroxyethyl starch on exogenous coagulation and active protein C in patients with septic shock]. In: Zhonghua wei zhong bing ji jiu yi xue 27 (1), S. 28–32. DOI: 10.3760/cma.j.issn.2095-4352.2015.01.007.

Ma, P. L.; Peng, X. X.; Du, B.; Hu, X. L.; Gong, Y. C.; Wang, Y.; Xi, X. M. (2015): Sources of Heterogeneity in Trials Reporting Hydroxyethyl Starch 130/0.4 or 0.42 Associated Excess Mortality in Septic Patients: A Systematic Review and Meta-regression. In: Chin Med.J.(Engl.). 128 (17), S. 2374–2382.

Mahmood et al. 2015 Anesth Pain Med. 2016; 6(1): e30326

Masoumi, K., et al., Comparison of the Effectiveness of Hydroxyethyl Starch (Voluven) Solution With Normal Saline in Hemorrhagic Shock Treatment in Trauma. J Clin Med Res, 2016. 8(11): p. 815-818.

Matsota, P., et al., The effect of 0.5 L 6% hydroxyethyl starch 130/0.42 versus 1 L Ringer's lactate preload on the hemodynamic status of parturients undergoing spinal anesthesia for elective cesarean delivery using arterial pulse contour analysis. J Anesth, 2015. 29(3): p. 352-359.

Mercier, F.J., et al., 6% Hydroxyethyl starch (130/0.4) vs Ringer's lactate preloading before spinal anaesthesia for Caesarean delivery: the randomised, double-blind, multicentre CAESAR trial. Br J Anaesth, 2014. 113(3): p. 459-67.

Miao, N., et al., Comparison of low molecular weight hydroxyethyl starch and human albumin as priming solutions in children undergoing cardiac surgery. Perfusion, 2014. 29(5): p. 462-8.

Min, J.J., et al., Effects of 6% hydroxyethyl starch 130/0.4 on postoperative blood loss and kidney injury in off-pump coronary arterial bypass grafting: A retrospective study. Medicine (Baltimore), 2017. 96(18): p. e6801.

Mitra, T., et al., Prevention of altered hemodynamics after spinal anesthesia: A comparison of volume preloading with tetrastarch, succinylated gelatin and ringer lactate solution for the patients undergoing lower segment caesarean section. Saudi J Anaesth, 2014. 8(4): p. 456-62.

Moerman, A., et al., The effect of hydroxyethyl starch 6% 130/0.4 compared with gelatin on microvascular reactivity. Anaesthesia, 2016. 71(7): p. 798-805.

Momeni, M., et al., The dose of hydroxyethyl starch 6% 130/0.4 for fluid therapy and the incidence of acute kidney injury after cardiac surgery: A retrospective matched study. PLoS One, 2017. 12(10): p. e0186403.

Mueller, R. B.; Haase, N.; Lange, T.; Wetterslev, J.; Perner, A. (2014): Acute kidney injury with hydroxyethyl starch 130/0.42 in severe sepsis. In: Acta anaesthesiologica Scandinavica. DOI: 10.1111/aas.12453.

Mueller, R. B.; Ostrowski, S. R.; Haase, N.; Wetterslev, J.; Perner, A.; Johansson, P. I. (2015): Markers of endothelial damage and coagulation impairment in patients with severe sepsis resuscitated with hydroxyethyl starch 130/0.42 vs Ringer acetate. In: Journal of critical care. DOI: 10.1016/j.jcrc.2015.11.025.

Muller, R.B., et al., Acute kidney injury with hydroxyethyl starch 130/0.42 in severe sepsis. Acta Anaesthesiol Scand, 2015. 59(3): p. 329-36.

Muller, R.G., et al., Effects of hydroxyethyl starch in subgroups of patients with severe sepsis: exploratory post-hoc analyses of a randomised trial. Intensive Care Med, 2013. 39(11): p. 1963-1971.

Myburgh, J. A.; Finfer, S.; Bellomo, R.; Billot, L.; Cass, A.; Gattas, D. et al. (2012): Hydroxyethyl starch or saline for fluid resuscitation in intensive care. In: N.Engl.J.Med. 367 (20), S. 1901–1911.

Myburgh, J.A., S. Finfer, and L. Billot, Hydroxyethyl starch or saline in intensive care. N Engl J Med, 2013. 368(8): p. 775.

Nagy, K.K., et al., A comparison of pentastarch and lactated Ringer's solution in the resuscitation of patients with hemorrhagic shock. Circ Shock, 1993. 40(4): p. 289-94.

Patel, A., et al., Reanalysis of the Crystalloid versus Hydroxyethyl Starch Trial (CHEST). N Engl J Med, 2017. 377(3): p. 298-300.

Patel, J., et al., Comparison of Albumin, Hydroxyethyl Starch and Ringer Lactate Solution as Priming Fluid for Cardiopulmonary Bypass in Paediatric Cardiac Surgery. J Clin Diagn Res, 2016. 10(6): p. UC01-4.

Pearse, R.M., et al., Effect of a perioperative, cardiac output-guided hemodynamic therapy algorithm on outcomes following major gastrointestinal surgery: a randomised clinical trial and systematic review. JAMA, 2014. 311(21): p. 2181-90.

Peng, Y., et al., Effects of colloid pre-loading on thromboelastography during elective intracranial tumor surgery in pediatric patients: hydroxyethyl starch 130/0.4 versus 5% human albumin. BMC Anesthesiol, 2017. 17(1): p. 62.

Perel, P., I. Roberts, and K. Ker, Colloids versus crystalloids for fluid resuscitation in critically ill patients. Cochrane Database Syst Rev, 2013. 2: p. CD000567.

Perner, A.; Haase, N.; Guttormsen, A. B.; Tenhunen, J.; Klemenzson, G.; Aneman, A. et al. (2012): Hydroxyethyl starch 130/0.42 versus Ringer's acetate in severe sepsis. In: The New England journal of medicine 367 (2), S. 124–134. DOI: 10.1056/NEJMoa1204242.

Pinar, H.U., et al., Effect of hydroxyethyl starch 130/0.4 on ischemia-reperfusion determinants in minor lower extremity surgery with tourniquet application. J Clin Anesth, 2015. 27(2): p. 105-10.

Principal investigator: C. Spiess et al. (Berlin, Deutschland) [Unpublished]

Principal investigator: D. Mazer (Toronto, Canada) [unpublished]

Pshenisnov, K.V., et al., [Features of Fluid Therapy in Children with Severe Major Trauma]. Anesteziol Reanimatol, 2016. 61(1): p. 28-32.

Qureshi, S. H.; Rizvi, S. I.; Patel, N. N.; Murphy, G. J. (2015): Meta-analysis of colloids versus crystalloids in critically ill, trauma and surgical patients. In: The British journal of surgery. DOI: 10.1002/bjs.9943.

Ravi, P.R. and B. Puri, Fluid resuscitation in haemorrhagic shock in combat casualties. Disaster Mil Med, 2017. 3: p. 2.

Reinhart, K. and W. Schummer, No new compelling evidence for the clinical benefit or safety of hydroxyethyl starch: a systematic review of recent data in perioperative settings. Unpublished, 2017.

Rochwerg, B.; Alhazzani, W.; Sindi, A.; Heels-Ansdell, D.; Thabane, L.; Fox-Robichaud, A. et al. (2014): Fluid Resuscitation in Sepsis: A Systematic Review and Network Metaanalysis. In: Annals of internal medicine 161 (5), S. 347–355. DOI: 10.7326/M14-0178.

Romdhani, C., et al., Lower incidence of hypotension following spinal anesthesia with 6% hydroxyethyl starch preload compared to 9 per thousand saline solution in caesarean delivery. Tunis Med, 2014. 92(6): p. 406-10.

Rossaint, R., et al., The European guideline on management of major bleeding and coagulopathy following trauma: fourth edition. Critical Care, 2016. 20: p. 100.

Ryhammer, P.K., et al., Colloids in Cardiac Surgery-Friend or Foe? J Cardiothorac Vasc Anesth, 2017. 31(5): p. 1639-1648.

Saghafinia, M., et al., The Effects of Hydroxyethyl Starch 6% and Crystalloid on Volume Preloading Changes following Spinal Anesthesia. Adv Biomed Res, 2017. 6: p. 115.

Schramko, A., et al., The use of balanced HES 130/0.42 during complex cardiac surgery; effect on blood coagulation and fluid balance: a randomised controlled trial. Perfusion, 2015. 30(3): p. 224-32.

Serpa, Neto A.; Veelo, D. P.; Peireira, V. G.; Assuncao, M. S. de; Manetta, J. A.; Esposito, D. C.; Schultz, M. J. (2013): Fluid resuscitation with hydroxyethyl starches in patients with sepsis is associated with an increased incidence of acute kidney injury and use of renal replacement therapy: A

systematic review and meta-analysis of the literature. In: Journal of critical care. DOI: 10.1016/j.jcrc.2013.09.031.

Shackelford, S.A., et al., Association of Prehospital Blood Product Transfusion During Medical Evacuation of Combat Casualties in Afghanistan With Acute and 30-Day Survival. Jama, 2017. 318(16): p. 1581-1591.

Skhirtladze, K., et al., Comparison of the effects of albumin 5%, hydroxyethyl starch 130/0.4 6%, and Ringer's lactate on blood loss and coagulation after cardiac surgery. Br J Anaesth, 2014. 112(2): p. 255-64.

Skytte Larsson, J., et al., Effects of acute plasma volume expansion on renal perfusion, filtration, and oxygenation after cardiac surgery: a randomised study on crystalloid vs colloid. Br J Anaesth, 2015. 115(5): p. 736-42.

Sprengel, K., et al., Resuscitation with polymeric plasma substitutes is permissive for systemic inflammatory response syndrome and sepsis in multiply injured patients: a retrospective cohort study. Eur J Med Res, 2016. 21(1): p. 39.

Sudfeld, S., et al., Impact of perioperative administration of 6 % hydroxyethyl starch 130/0.4 on serum cystatin C-derived renal function after radical prostatectomy: a single-centre retrospective study. BMC Anesthesiol, 2016. 16(1): p. 69.

Sun et al. 2016 Congress abstract

Szturz, P., et al., Individual goal-directed intraoperative fluid management of initially hypovolaemic patients for elective major urological surgery. Bratisl Lek Listy, 2014. 115(10): p. 653-9.

Taylor, C., et al., Hydroxyethyl starch versus saline for resuscitation of patients in intensive care: long-term outcomes and cost-effectiveness analysis of a cohort from CHEST. Lancet Respir Med, 2016. 4(10): p. 818-825.

Taylor, K.; Thompson, S.; Finfer, A.; Higgins, S.; Jan, Q.; Li, B. et al. (2016): Hydroxyethyl starch versus saline for resuscitation of patients in intensive care. Long-term outcomes and cost-effectiveness analysis of a cohort from CHEST. In: The Lancet Respiratory Medicine 4 (10), S. 818–825. DOI: 10.1016/S2213-2600(16)30120-5.

Terkawi, A.S., et al., Effects of hydroxyethyl starch 6 % (130/0.4) on blood loss during cesarean delivery: a propensity-matched analysis. J Anesth, 2016. 30(5): p. 796-802.

Tobey, R., et al., Postoperative Acute Kidney Injury and Blood Product Transfusion After Synthetic Colloid Use During Cardiac Surgery. J Cardiothorac Vasc Anesth, 2017. 31(3): p. 853-862.

Tör et al. 2016 Niger J Clin Pract 2016; 19(1):115-20

Unlugenc, H., et al., Rapid Fluid Administration and the Incidence of Hypotension Induced by Spinal Anesthesia and Ephedrine Requirement: The Effect of Crystalloid Versus Colloid Coloading. Middle East J Anaesthesiol, 2015. 23(3): p. 273-81.

Vallet, B., et al., [Guidelines for perioperative haemodynamic optimization. Socie te franc aise d'anesthe sie et de re animation]. Ann Fr Anesth Reanim, 2013. 32(6): p. 454-62.

Van der Linden, P., et al., Efficacy and safety of 6% hydroxyethyl starch 130/0.4 (Voluven) for perioperative volume replacement in children undergoing cardiac surgery: a propensity-matched analysis. Crit Care, 2015. 19: p. 87.

Van Der Linden, P., et al., Safety of modern starches used during surgery. Anesth Analg, 2013. 116(1): p. 35-48.

Velasquez, T.; Mackey, G.; Lusk, J.; Kyle, U. G.; Fontenot, T.; Marshall, P. et al. (2016): ESICM LIVES 2016. Part three. In: Intensive Care Medicine Experimental 4 (1), S. 28. DOI: 10.1186/s40635-016-0100-7.

Vincent, J.L., et al., The SOFA (Sepsis-related Organ Failure Assessment) score to describe organ dysfunction/failure. On behalf of the Working Group on Sepsis-Related Problems of the European Society of Intensive Care Medicine. Intensive Care Med, 1996. 22(7): p. 707-10.

Vives, M., et al., Modern hydroxyethyl starch and acute kidney injury after cardiac surgery: a prospective multicentre cohort. Br J Anaesth, 2016. 117(4): p. 458-463.

Wang, Zhengguang; Zhang, Mucheng; Wang, Jianlei; Fang, Xiangqun; Zheng, Shaopeng; Zhang, Quchu (2015): Investigation regarding the correlation between hydroxyethyl starch administration and acute kidney injury in critically ill patients. In: Zhonghua wei zhong bing ji jiu yi xue 27 (5), S. 338–342. DOI: 10.3760/cma.j.issn.2095-4352.2015.05.004.

Xia, J., et al., The brain relaxation and cerebral metabolism in stroke volume variation-directed fluid therapy during supratentorial tumors resection: crystalloid solution versus colloid solution. J Neurosurg Anesthesiol, 2014. 26(4): p. 320-7.

Yates, D.R., et al., Crystalloid or colloid for goal-directed fluid therapy in colorectal surgery. Br J Anaesth, 2014. 112(2): p. 281-9.

You et al. 2016 Int J Clin Exp Med 2016; 9(2): 4340-7

Younes, R.N., et al., Use of pentastarch solution in the treatment of patients with hemorrhagic hypovolaemia: randomised phase II study in the emergency room. World J Surg, 1998. 22(1): p. 2-5.

Zarychanski, R., et al., Association of hydroxyethyl starch administration with mortality and acute kidney injury in critically ill patients requiring volume resuscitation: a systematic review and meta-analysis. JAMA, 2013. 309(7): p. 678-88.

Zhang, Y., et al., Administration of HES in elderly patients undergoing hip arthroplasty under spinal anesthesia is not associated with an increase in renal injury. BMC Anesthesiol, 2017. 17(1): p. 29.

Gales, B.J. and B.L. Erstad, *Adverse reactions to human serum albumin.* Ann Pharmacother, 1993. 27(1): p. 87-94.

Ripolles, J., et al., Intraoperative goal directed hemodynamic therapy in noncardiac surgery: a systematic review and meta-analysis. Braz J Anesthesiol, 2016. 66(5): p. 513-28.

Ertmer, C., et al., Fluid therapy and outcome: a prospective observational study in 65 German intensive care units between 2010 and 2011. Ann Intensive Care, 2018. 8(1): p. 27.

Appendix 1

Listing of stakeholders, including MAHs, who submitted responses to the Agency for EMEA/H/A-107i/1457

The following stakeholders submitted responses:

MAHs
B.BRAUN
Fresenius Kabi
Serumwerk Bernburg AG
Infomed Fluids Srl

Other stakeholders
European Society of Anaesthesiology
Healthcare professional (Anaesthesiology; DE)
Healthcare professional (Anaesthesiology; AT)
Healthcare professional (AT)
Czech Society of Anaesthesiology and Intensive Care Medicine
Healthcare professional (Anaesthesiology; DE)
Healthcare professional (Anaesthesiology; DE)
The BMJ
Healthcare professional (Intensive Care Medicine; BE)
Healthcare professional (Internal Medicine; Intensive Care Medicine; AT)
Healthcare professional (Anaesthesiology; DE)
Healthcare professional (Anaesthesiology; BE)
Healthcare professional (Intensive Care Medicine; Russia)
Healthcare professional (Anaesthesiology; ES)
Healthcare professional (Intensive Care Medicine, Anaesthesiology; BE)
Healthcare professional (Intensive Care Medicine; China)
German Society of Anaesthesiology and Intensive Care Medicine (DGAI)
Public Citizen, Health Research Group (USA)
SepNet Critical Care trials group (DE)
Healthcare professional (Intensive Care Medicine, Anaesthesiology; BE)
Global Sepsis Alliance (DE)
Healthcare professional (Intensive Care Medicine, Anaesthesiology; DE)
Healthcare professional (Intensive Care Medicine, Anaesthesiology; ES)
Healthcare professional (Anaesthesiology; AT)

Other stakeholders
Industry
Healthcare professional (Intensive Care Medicine, Anaesthesiology; DE)
Healthcare professional (Intensive Care Medicine, BE)
Healthcare professional (Intensive Care Medicine, BE)
Healthcare professional (Anaesthesiology; BE)
German Society of Transfusion Medicine and Immunohematology (DGTI)

Appendix 2

Divergent positions

Article 107i of Directive 2001/83/EC resulting from pharmacovigilance data

Procedure No: EMEA/H/A-107i/1457

Solutions for infusion containing hydroxyethyl starch (HES)

Divergent statement

The undersigned PRAC members disagree with the recommendations of PRAC to suspend the marketing authorisation of HES-containing products. This recommendation is based on the findings of two drug utilisation studies (DUS) aimed to assess the effectiveness of the risk minimisation measures put in place after Article 107i Referral finalised in September 2013. The results of the DUS have shown that risk minimisation measures were not completely effective mainly due to non-adherence or poor adherence to revised indication and contraindications. Causes of the non-adherence are not clear and should be further investigated.

It is acknowledged that results from the two conducted DUSs show some use outside the terms of the marketing authorisation. However, adherence to revised maximum daily dose and treatment duration was satisfactory in both studies. Current daily dose and treatment duration differ from those used in the studies which showed an increased risk of renal damage and mortality in critically ill patients. As the PRAC Rapporteurs mentioned in the presentation at PRAC plenary in January 2018, the DUS design has some limitations and possibility of misclassification. No new robust safety data have been provided with current conditions of use and therefore it is uncertain if the same level of risk is shared with dose and duration restrictions.

The PRAC Members signed below have consulted national experts in the field and in line with the conclusions of the EMA Expert Group (SAG) and the European Society of Anaesthesiology, they consider that HES-containing products play a role in the therapeutic armamentarium of hypovolaemic shock in patients who cannot be stabilised with crystalloids alone. SAG considered that a suspension of the marketing authorisation of HES-containing medicinal products could lead to an unmet medical need in some hypovolaemic situations. SAG also acknowledged that suspension of HES-containing products could increase the problem of fluid overload in some patients.

Since 2013 there are no data about any new risk, therefore benefit-risk balance of HES-containing products remains unchanged. HES-containing products provide a more rapid volume expansion with less volume given and consequently avoid the adverse consequences of fluid overload associated to excessive crystalloids infusion. Other alternatives are not devoid of risks, unsuitable in some clinical situations, and some of them subject to frequent shortages.

Two clinical trials have already started with the aim of assessing HES-containing products safety and efficacy in elective surgery and trauma patients. Such trials were requested at the time of the first Article 107i referral and should provide relevant information in order to assess the role of these products in clinical use. Based on the results of these studies, a full benefit risk assessment could take place again. Suspension of the marketing authorisation in the European Union at this stage will certainly threaten the completion of these studies and therefore lead to the non-collection of additional important data on efficacy and safety.

It is well understood that further additional risk minimisation measures should be taken so that current indications and contraindications are fully met. In this regard, several measures such as further

communication both from marketing authorisation holders and national competent authorities, interactions at national level with involved learned societies and the development of a simple algorithm reminding how to use the product, could be certainly helpful. In fact, previous risk minimisation measures have had already a positive impact on the way how HES-containing products are being used (important decrease in usage in all EU Member States after measures taken in 2013 and very good compliance with maximal dose and duration of treatment).

On April 2018, the European Commission considered that there were new questions, in particular with regard to any unmet medical need, the availability of alternatives, the impact of HES suspension and the feasibility and likely effectiveness of risk minimisation measures. In view of these issues, it is important to take into consideration the following:

- several Member States have identified an unmet medical need as also expressed by 16 European Societies of Anaesthesiology in a letter addressed on the 14th of March 2018 to the European Commission;
- many Member States proposed some risk minimisation measures (RMMs) such as inclusion of a warning label on the immediate packaging (bag), sending a new DHPC or other proactive communication to healthcare professionals to further highlight the restrictions on use and the results of the DUS studies and recommend that learned societies should be closely involved with the process of further minimising the risks of HES solutions. The Marketing Authorization Holders have also presented a joint program which could minimise the risk of use of HES in incorrect indications and in contraindications;

In conclusion, the undersigned PRAC members disagree with the PRAC position to say that there is no identified unmet medical need and that no risk minimisation measures which would be feasible to implement and be sufficiently effective in ensuring adherence to the product information. Thus they maintain their divergent opinion and consider that the suspension of marketing authorisation of HES-containing products is currently not risk proportionate as it is not based on any new data about risks and it may negatively impact the management of some patients facing emergency situations.

PRAC Members expressing a divergent opinion:

Dolores Montero Corominas (ES)	17 May 2018
Eva Jirsová (CS)	17 May 2018
Tatiana Magalova (SK)	17 May 2018
Ghania Chamouni (FR)	17 May 2018

Gabriela Jazbec (SI)	17 May 2018
Herve Le Louet (Co-opted member)	17 May 2018
Julia Pallos (HU)	17 May 2018
Jean-Michel Dogné (BE)	17 May 2018
Marcel Bruch (LU)	17 May 2018
Roxana Dondera (RO)	17 May 2018
Sofia Trantza (GR)	17 May 2018
Thierry Trenque (Co-opted member))	17 May 2018
Željana Margan Koletić (HR)	17 May 2018

Article 107i of Directive 2001/83/EC resulting from pharmacovigilance data

Procedure No: EMEA/H/A-107i/1457

Solutions for infusion containing hydroxyethyl starch (HES)

Divergent statement

The undersigned PRAC members disagree with the recommendations of PRAC to suspend the marketing authorisation of HES-containing medicinal products. This recommendation is based on the findings of two drug utilisation studies (DUS) aimed to assess the effectiveness of the risk minimisation measures put in place after Article 107i Referral finalised in September 2013. The results of the DUS have shown that risk minimisation measures were not completely effective mainly due to non-adherence or poor adherence to revised indication and contraindications. It is fully acknowledged that results from the two conducted DUSs show use outside the terms of the marketing authorisation.

In this regard, measures to further minimize the risks should be strengthened so that current indications and contraindications are fully met. Several routine and additional risk minimisation measures have been proposed, such as amendments to the product information, further communication to HCPs, warnings on the immediate packaging, restricted access to HES-containing products and the development of an educational programme also involving professional and learned societies. These measures as outlined in detail in the PRAC assessment report are considered helpful and suitable as a total package in order to increase the awareness towards the important risks associated with HES and could result in a more careful and restrictive use of HES in hospitals. Furthermore, previously implemented risk minimisation measures have already had an impact on the way HES-containing medicinal products are being used showing overall a decrease in usage across the EU.

The PRAC Members signed below have consulted national experts in the field and in line with the conclusions of the EMA Expert Group (SAG) and the German Society of Anaesthesiology and Intensive Care (DGAI), they consider that HES-containing medicinal products play a role in the therapeutic armamentarium of hypovolaemic shock in patients who cannot be stabilised with crystalloids alone. In addition, it should be considered that HES has the greatest effect in terms of intravasal retention time and volume fill effects as a ratio of plasma volume increase to supplied volume. This is particularly important for patients who are sensitive to the administration of large volumes. The SAG considered that a suspension of the marketing authorisation of HES-containing medicinal products could lead to an unmet medical need in some hypovolaemic situations.

Two clinical trials have already started with the aim of assessing the safety and efficacy of HES-containing medicinal products in elective surgery and trauma patients. Such trials were requested at the time of the first Article 107i referral and should provide relevant information in order to assess the role of these products in clinical use. Based on the results of these studies, a full benefit-risk assessment could take place again. It is therefore considered appropriate to first await the final results of these trials.

In conclusion, it is considered that the suspension of marketing authorisation of HES-containing medicinal products is currently not risk proportionate as it may negatively impact the management of some patients facing emergency situations.

PRAC Members expressing a divergent opinion:

Martin Huber (DE)	17 May 2018
Brigitte Keller-Stanislawski (Co-opted member)	17 May 2018

EUROPEAN MEDICINES AGENCY
SCIENCE MEDICINES HEALTH

29 June 2018
EMA/422341/2018

Hydroxyethyl starch solutions: CMDh introduces new measures to protect patients

Medicines to remain on the market provided that training, controlled access and warnings on the packaging are implemented

The CMDh[1] has decided that hydroxyethyl starch (HES) solutions for infusion should remain on the market provided that a combination of additional measures to protect patients is implemented. This follows further reflection, in consultation with EU Member States, on whether it would be feasible to introduce new measures that would effectively reduce the risks with these medicines.

HES solutions for infusion are used to replace plasma volume following acute (sudden) blood loss, where treatment with alternative products known as ‘crystalloids’ alone is not considered sufficient.

In January 2018, EMA’s safety committee PRAC recommended suspending the marketing authorisations of these medicines because they continued to be used in critically ill patients and patients with sepsis despite restrictions introduced in 2013 due to the risk of kidney injury and death in these patients.

The CMDh agreed with the PRAC’s assessment of the serious risks in critically ill patients and patients with sepsis. However, the CMDh gave further consideration to the place of HES in the clinical practice of some countries, noted that previous risk minimisation measures had some effect, and considered that a combination of new risk minimisation measures would effectively ensure that HES solutions are not used in patients at risk.

The new measures are:

- the implementation of a controlled access programme by the companies holding the marketing authorisations to ensure that only accredited hospitals will be supplied with these medicines. The accreditation would require that relevant healthcare professionals receive training on the safe use of HES solutions for infusion. Further details about the training and the controlled access programme will be provided to hospitals and healthcare professionals in due time;
- warnings in the medicines’ packaging and at the top of the summaries of product characteristics (SmPCs) reminding healthcare professionals that these medicines must not be used in patients with sepsis or kidney impairment or in critically ill patients;

[1] The CMDh is a medicines regulatory body representing the European Union (EU) Member States, Iceland, Liechtenstein and Norway.

30 Churchill Place ● Canary Wharf ● London E14 5EU ● United Kingdom
Telephone +44 (0)20 3660 6000 **Facsimile** +44 (0)20 3660 5555
Send a question via our website www.ema.europa.eu/contact

An agency of the European Union

© European Medicines Agency, 2018. Reproduction is authorised provided the source is acknowledged.

- writing directly to healthcare professionals to ensure that they are fully aware of the conditions of use of the medicines and the groups of patients that must not receive them due to an increased risk of kidney injury and death.

The CMDh also requested marketing authorisation holders to conduct studies to check that only patients who should be treated with these medicines are receiving them. This is in addition to ongoing studies on the benefits and risks of HES solutions in patients with trauma and those undergoing elective surgery.

The CMDh position was adopted by majority vote and the matter will now be sent to the European Commission, which will take an EU-wide legally binding decision.

Information for patients

- HES solutions for infusion are used to replace fluids in the body after acute (sudden) blood loss.
- Because of the risk of kidney injury and death, HES solutions for infusion must not be used in patients with blood infection or kidneys problems or in critically ill patients.
- If you are given a HES infusion, your doctor will monitor your kidneys to check that they are working well enough.
- Patients who have questions or concerns should speak to their treating doctor.

Information for healthcare professionals

- Because of the risk of kidney injury and mortality, HES solutions for infusion are contraindicated in patients with **sepsis or in critically ill patients**.
- HES solutions for infusion should be used for managing hypovolaemia due to acute blood loss only when crystalloids alone are not considered sufficient. HES solutions should not be used for fluid maintenance.
- Use of HES solutions for infusion should be restricted to the initial phase of volume resuscitation with a maximum time interval of 24 hours. Treatment should be guided by continuous haemodynamic monitoring so that the infusion is stopped as soon as haemodynamic goals have been achieved.
- Additional studies are ongoing with HES solutions in patients with trauma and those undergoing elective surgery to further investigate the long-term safety of HES prescribed according to the recommendations for use (dose less than 30 ml/kg and duration less than 24 hours).
- The expected benefit of treatment should be carefully weighed against the uncertainties with regard to long-term safety.
- Alternative therapeutic options are available for routine clinical practice and should be considered according to relevant clinical guidelines.
- HES solutions for infusion are contraindicated in patients with **renal impairment or undergoing renal replacement therapy**. The use of HES must be discontinued at the first sign of renal injury. An increased need for renal replacement therapy has been reported up to 90 days after HES administration. Patients' kidney function should be monitored after HES administration.

- HES solutions for infusion are contraindicated in **severe coagulopathy**. HES solutions should be discontinued at the first sign of coagulopathy. Blood coagulation parameters should be monitored carefully in case of prolonged use.
- HES solutions for infusion are also contraindicated in **dehydrated patients, hyperhydrated patients, patients with intracranial or cerebral haemorrhage, burn injuries, severe hyperkalaemia, hypernatraemia, hyperchloraemia, congestive heart failure, organ transplant patients and patients with impaired hepatic function.**

Healthcare professionals will be informed in writing of the outcome of the review and the introduction of the new risk minimisation measures, which include the introduction of a controlled access programme requiring training of relevant healthcare professionals on the safe use of these medicines. This programme will be implemented by the companies holding the marketing authorisations. Further details about the training and the controlled access programme will be provided to hospitals and healthcare professionals in due time.

More about the medicine

HES solutions for infusion are used for the management of hypovolaemia (low blood volume) caused by acute blood loss, where treatment with alternative infusion solutions known as 'crystalloids' alone is not considered sufficient. They are given by infusion (drip) into a vein and are used as blood volume expanders to prevent a dangerous drop in blood pressure following acute bleeding. They belong to the class of medicines known as colloids. Besides blood products, there are two types of medicines used for plasma volume replacement: crystalloids and colloids. Colloids contain large molecules such as starch, whereas crystalloids, such as saline or Ringer's solutions, are electrolyte solutions.

In the European Union, HES solutions for infusion have been approved via national procedures and are available in the Member States under various trade names.

More about the procedure

The review of HES solutions for infusion was initiated on 17 October 2017 at the request of the Swedish Medical Products Agency, under Article 107i of Directive 2001/83/EC.

The review was first conducted by the Pharmacovigilance Risk Assessment Committee (PRAC), EMA's Committee responsible for the evaluation of safety issues for human medicines, which made a set of recommendations on 12 January 2018. The PRAC recommendations were sent to Co-ordination Group for Mutual Recognition and Decentralised Procedures – Human (CMDh), which adopted a position on 24 January 2018. The CMDh, a body representing EU Member States, is responsible for ensuring harmonised safety standards for medicines authorised via national procedures across the EU.

As the CMDh position was adopted by majority vote, it was sent to the European Commission. In April 2018, the European Commission requested that the PRAC and the CMDh further consider any possible unmet medical need that could result from a suspension, as well as the feasibility and likely effectiveness of additional risk minimisation measures.

After looking at these specific aspects, in May 2018 the PRAC confirmed its previous recommendation for suspension and sent a revised recommendation to the CMDh. The CMDh has now concluded that

HES solutions for infusion should remain on the market provided that a combination of additional measures to protect patients is implemented.

As the CMDh position was adopted by majority vote, the CMDh position will now be sent back to the European Commission, which will take an EU-wide legally binding decision.

Annex II

Scientific conclusions and CMDh's detailed explanation on the scientific grounds for differences with the PRAC recommendation

Scientific conclusions

In 2013, following a review of the risk of kidney injury and mortality related to hydroxyethyl starch (HES) solutions for infusion when administered in patients with sepsis or critical illness, the Pharmacovigilance and Risk Assessment Committee (PRAC) recommended risk minimisation measures such as restrictions in use of these medicinal products. PRAC also recommended a drug utilisation study to evaluate the effectiveness of these risk minimisation measures.

Results from two drug utilisation studies, submitted by the concerned Marketing Authorisation Holders ('MAHs') in 2017, have shown that the recommended restrictions in use are not being sufficiently adhered to.

On 17 October 2017, Sweden triggered an urgent Union procedure under Article 107i of Directive 2001/83/EC, and requested the PRAC to assess the impact of the above non-adherence to the product information on the benefit-risk balance of hydroxyethyl starch (HES) solutions for infusion and issue a recommendation on whether the marketing authorisations of these products should be maintained, varied, suspended or revoked.

The PRAC adopted a recommendation on 11 January 2018 which was then considered by the CMDh, in accordance with Article 107k of Directive 2001/83/EC.

Overall summary of the scientific evaluation by the PRAC

Hydroxyethyl starch (HES) solutions for infusion contain starch with different molecular weights (mainly 130kD; 200kD) and substitution ratio (the number of hydroxyethyl groups *per* glucose molecule). HES solutions for infusion are authorised worldwide for the treatment of hypovolaemia associated with various conditions.

In 2012 and 2013, PRAC reviewed the benefits and risks of HES solutions for infusion in the treatment and prophylaxis of hypovolaemia, within Article 31[1] and 107i[2] referral procedures. These reviews were triggered by the results from large randomised clinical studies[3,4,5] which showed an increased risk of mortality in patients with sepsis and an increased risk of kidney injury requiring dialysis in critically ill patients following treatment with HES solutions for infusion.

As result of the reviews, the PRAC recommended that use of HES solutions for infusion should be restricted to the treatment of hypovolaemia due to acute blood loss when crystalloids alone are not considered sufficient. The PRAC also contraindicated the use of HES solutions for infusion in patients with sepsis or who are critically ill. Furthermore, the PRAC requested that, as conditions to the marketing authorisations of these medicinal products, further studies should be carried out on the use of these medicines in elective surgery and in trauma patients. The PRAC also required that drug utilisation should be studied to evaluate the effectiveness of the risk minimisation measures. The focus for the drug utilisation studies (DUSs) has been to evaluate the adherence to the restrictions in use, implemented in the product information, concerning the indication, posology, and contraindication for HES solutions for infusion.

---

[1] http://www.ema.europa.eu/ema/index.jsp?curl=pages/medicines/human/referrals/Hydroxyethyl_starch-containing_solutions/human_referral_prac_000012.jsp&mid=WC0b01ac05805c516f

[2] http://www.ema.europa.eu/ema/index.jsp?curl=pages/medicines/human/referrals/Hydroxyethyl_starch-containing_medicines/human_referral_prac_000029.jsp&mid=WC0b01ac05805c516f

[3] Perner A, Haase N, Guttormsen AB et al. Hydroxyethyl starch 130/0.42 versus ringer's acetate in severe sepsis. N Engl J Med 2012;367(2):124-34

[4] Brunkhorst FM, Engel C, Bloos F et al. Intensive Insulin Therapy and Pentastarch Resuscitation in Severe Sepsis. N Engl J Med 2008; 358(2):125-39

[5] Myburgh J, Finder S, Bellomo R *et al*. Hydroxyethyl starch or saline for fluid resuscitation in intensive care. *N Engl J Med* 2012; 367:1901-11

On 5th July 2017 and 9th October 2017, results from two DUSs on the effectiveness of the implemented risk minimisation measures have become available. These include drug utilisation data from 11 EU Member States. These data raise serious concerns as they showed use of HES solutions for infusion in patient populations which are contraindicated such as those who are critically ill, or with sepsis[3,4,5]. In light of the well-established risk for serious harm when HES solutions for infusion are used in patients with critical illness, including sepsis, together with the above-mentioned newly available data, Sweden triggered, on 17th October 2017, an urgent Union procedure under Article 107i of Directive 2001/83/EC. Due to the serious public health impact, Sweden was considering suspending the marketing authorisations for the above mentioned medicinal products, and therefore requested an urgent review of the matter at the European level, and asked the PRAC to assess the impact of the above concerns on the benefit-risk balance of HES solutions for infusion and issue a recommendation on whether the marketing authorisations of these products should be maintained, varied, suspended or revoked.

In its assessment, the PRAC considered the totality of evidence which includes all newly available data since the previous referral procedures, including results from DUSs, clinical studies, meta-analyses of clinical studies, post-marketing experience, Eudravigilance data, literature review, responses submitted by the marketing authorisation holders (MAHs) in writing and at oral explanations as well as stakeholders' submissions and views expressed by experts during an ad-hoc experts meeting, taking into account also the characterisation of benefits and risks concluded in the previous referral procedures.

The PRAC also considered views from individual PRAC members on the benefit/risk balance of HES solutions for infusion as well as on the use of these products at the national level. These views are based on PRAC members' routine review processes and preparation. These views, along with all substantial data and information crucial for the full understanding of these views have been either shared with all the parties involved or otherwise made available in the course of the procedure.

With regards to efficacy, PRAC considered that there is no new significant information related to the approved indication. Overall, the evidence for this indication is based on studies for which the sample size and the duration of follow-up are limited. Is it also noted that although the benefit has been demonstrated in terms of a volume-sparing effect, and there is some support for short-term hemodynamic effects, it remains uncertain to what extent this translates into more patient-relevant outcomes. The benefits in the approved indication therefore remain modest.

With regards to the safety data related to these products, the PRAC reviewed all available evidence since the last referral and concluded that the previous conclusions that HES solutions for infusion is associated with an increased risk of mortality and renal failure in patients with sepsis or critical illness were confirmed and that the available information, including more recently submitted clinical data, do not change the established risk in these patient populations.

Treatment of hypovolemia should replace lost blood volume in order to restore tissue perfusion and oxygenation to ultimately prevent renal injury and death. There is a direct relation between the degree of hypovolemia and the risk for renal injury and death. A more pronounced hypovolemia requires a greater volume (dose) of HES solutions for infusion and is also associated with a greater risk for renal injury and death. Consequently, a direct correlation is to be expected between the indication for treatment with HES solutions for infusion, the dose of HES solutions for infusion required and with the risk for renal injury and death. It should also be noted that the ultimate benefit expected from HES solutions for infusion (and treatment of hypovolemia in general) is a reduction of mortality and lower incidence of renal failure. The safety concerns of primary importance in this referral are increased mortality and a higher incidence of renal failure – the opposite of the benefit expected.

Amongst other data related to safety, the PRAC reviewed the results from two separate DUSs conducted to assess the effectiveness of the risk minimisation measures imposed as an outcome of the 2013 referral, and concluded that these studies despite a potential limitation of possible misclassification, are representative of the clinical usage in the European Union and that key results are reliable. The results indicate that the implemented restrictions in use are not sufficiently adhered to. Overall non-adherence to the revised product information was reported to be high, and PRAC was particularly concerned that approximately 9% of patients exposed to HES solutions for infusion were critically ill, approximately 5-8% of patients had renal impairment and approximately 3-4% of patients had sepsis.

In view of the overall exposure to HES solutions for infusion in the EU, estimated to about 1.5 to 2 million patients per year since 2014 and the reported extent of usage in patients with sepsis from the two DUSs, the estimated level of continued usage in populations where serious harm has been demonstrated raises important public health concerns, including a potentially increased mortality.

The PRAC considered other further risk minimisation measures to sufficiently minimise this exposure, including changes to the product information, direct health care professional communication, educational materials, warning on the primary container of the products, sign-in for medication form, prescription sheet/checklists. However, the available evidence shows that the non-adherence is not only due to a lack of awareness of the restrictions by prescribers but also due in some cases to deliberate choice, rendering further communication and education unlikely to be sufficiently effective to address the risks identified. The medication form/checklists would also raise feasibility issues in an emergency setting. Proposals to amend the indications and contraindications were not considered sufficient to have a significant impact on prescriber behaviour. The PRAC also noted that the current clinical experience suggests that it is difficult to clearly separate patient populations where randomised clinical trials have shown serious harm from populations targeted by the approved indication. Patients in the approved indication may become critically ill or septic shortly after receiving HES solutions for infusion and these patients cannot be identified prospectively. This complicates effective risk minimisation in these patients.

The PRAC concluded that no additional risk minimisation measures to ensure safe and effective use of HES solutions for infusion would be effective or feasible in a reasonable timeframe, where important number of patients at high risk would continue to be exposed.

The PRAC also consulted an ad-hoc expert group and considered carefully views expressed during the meeting that took place on 18 December 2017. The PRAC duly considered the view of the majority of the experts in the meeting that HES is used in clinical practice. The PRAC also noted the view of an expert concerning shared clinical experience in handling cases in a EU Member State where HES solutions for infusion are little used and where no medical need is raised.

This reflects a long-standing controversy among health-care professionals and echoes the range of stakeholder responses received in the current review.

Overall, taking into account the divergences amongst the experts on some important issues, the position from the PRAC members on the national situation regarding the clinical use of these products and the stakeholders submissions, PRAC did not consider the clinical utility of these products to outweigh the risk of mortality and renal failure to the proportion of patients with critical illness or sepsis that continue to be exposed to HES solutions for infusion.

In view of the seriousness of the safety issues and that the proportion of patients who are exposed to these risks in the absence of effective risk minimisation measures could have important public health consequences including a potentially increased mortality, the PRAC concluded that the benefit risk

balance of hydroxyethyl starch solutions for infusion is no longer favourable and recommended the suspension of the marketing authorisations.

The PRAC noted that the clinical studies imposed following previous referrals procedures (TETHYS and PHOENICS) to characterise the efficacy and safety in trauma and elective surgery, which is currently the target population for which the product is indicated are ongoing.

Having reviewed the PRAC recommendation[6], the CMDh agreed by a majority on 24 January 2018 with the PRAC overall conclusions and grounds for recommendation. The position was afterwards forwarded to the European Commission, the Member States, to Iceland and Norway and to the marketing authorisation holders for the above mentioned medicinal products, together with its annexes and appendices.

Revision of PRAC recommendation

During the decision-making process, at a meeting of the Standing Committee on Medicinal Products for Human Use, some EU Member States raised new questions of technical nature which they considered had not been sufficiently addressed in the PRAC recommendation and CMDh position. In light of this, the PRAC recommendation and CMDh position were referred back to the Agency by the European Commission for further consideration of any possible unmet medical need that could result from the suspension of the marketing authorisations for the medicinal products concerned by the referral, as well as the feasibility and likely effectiveness of additional risk minimisation measures.

The PRAC discussed the above two points at its May meeting, taking into consideration information provided by the Member States.

The PRAC has considered all elements expressed in relation to the impact of a suspension of the marketing authorisation for HES solutions for infusion on a potential unmet medical need at national level, including comments submitted by the MAHs in writing and at oral explanations, responses from Member States and other stakeholders' views.

With regard to the impact on a suspension of the marketing authorisations for HES solutions for infusion, fifteen EU Member States and Norway mentioned that no unmet medical need is expected in case of suspension of the marketing authorisations for HES solutions for infusion.

The PRAC carefully considered all the information provided in relation to a potential unmet medical need at national level should the marketing authorisations for HES solutions for infusion be suspended. Eight EU Member States have mentioned that a suspension of the marketing authorisations for HES solutions for infusion would have an impact in the national clinical practice as HES solutions for infusion fulfils currently a medical need in their territory. The PRAC considered that despite arguments raised by some Member States, the potential for unmet medical need is not established. Most of the arguments refer to the use of HES solutions for infusion outside the terms of the MA or to claimed benefits that are not clinically significant or supported by robust data.

The PRAC concluded that the claimed clinical utility for these products does not outweigh the risk of mortality and renal failure to the proportion of patients with critical illness or sepsis that continues to be exposed to HES solutions for infusion.

The PRAC have also further considered for the feasibility and likely effectiveness of risk minimisation measures.

[6] PRAC Recommendation EMA/PRAC/1707/2018 Corr.1 and PRAC AR EMA/PRAC/808891/2017 Corr.1

The PRAC considered further risk minimisation measures which could potentially sufficiently minimise this exposure, including restricted access / distribution to hospitals and physicians, changes to the product information, direct health care professional communication, educational materials to be distributed in cooperation with some learned societies, warning on the primary container of the products, medication form and follow-up questionnaire. However, the available evidence shows that the non-adherence is not only due to a lack of awareness of the restrictions by prescribers but also due to deliberate choice, rendering further communication and education unlikely to be sufficiently effective to address the risks identified. A restricted distribution system to accredited hospitals or physicians would raise serious feasibility concerns and would be unlikely to be effective considering the particular type of distribution and usage of HES solutions for infusion. A medication form to be filled before administration would also raise feasibility issues in an emergency setting. A follow-up questionnaire to be filled after administration would not be effective in minimising the risk. Proposals to amend the indications and contraindications were not considered have a sufficient impact on prescriber behaviour and were not supported by appropriate scientific evidence.

In conclusion, no risk minimisation measures or combination of measures have been identified which would be sufficiently effective or feasible to implement in a reasonable timeframe, when an important number of patients at high risk for serious harm, would continue to be exposed.

In the light of the above information, the PRAC confirmed at its May 2018 plenary meeting, its previous scientific conclusions that the benefit risk of HES solutions for infusion is negative and recommended to suspend the marketing authorisations of these medicinal products.

Revised grounds for PRAC recommendation

Whereas,

- The Pharmacovigilance Risk Assessment Committee (PRAC) considered the procedure under Article 107i of Directive 2001/83/EC, for hydroxyethyl starch (HES) solution for infusion (see Annex I).
- The PRAC reviewed all newly available data, including results from Drug Utilisation Studies (DUS), clinical studies, meta-analyses of clinical studies, post-marketing experience, Eudravigilance data, literature review, responses submitted by the marketing authorisation holders (MAHs) in writing and at oral explanations, stakeholders' submissions and views expressed by experts during an ad-hoc experts meeting. The PRAC also reviewed responses from Member States in relation to the potential unmet medical need and proposals for additional risk minimisation measures.
- With regards to the efficacy, PRAC considered that there is no new significant information related to the approved indication. Overall, the evidence for this indication is based on studies for which the sample size and the duration of follow-up are limited. Is it also noted that although the benefit has been demonstrated in terms of a volume-sparing effect, and there is some support for short-term hemodynamic effects, it remains uncertain to what extent this translates into more patient-relevant outcomes. The benefits in the approved indication therefore remain modest.
- With regards to the two separate DUSs conducted to assess the effectiveness of the risk minimisation measures imposed as an outcome of the 2013 referral, PRAC concluded that these studies, despite limitations due to possible misclassification, are representative of the clinical usage in the European Union and that key results are reliable. The results indicate that

the implemented restrictions in use are not adhered to. Overall non-adherence to the revised product information was reported to be high, and PRAC was particularly concerned that approximately 9% of patients exposed to HES solutions for infusion were critically ill, approximately 5-8% of patients had renal impairment and approximately 3-4% of patients had sepsis.

- The PRAC conclusions of previous reviews under Article 31 of Directive and Article 107i of Directive 2001/83/EC were that HES solutions for infusion are associated with an increased risk of mortality and renal failure in patients with sepsis or critical illness. PRAC confirmed that the available information, including more recent submitted clinical data, do not change the established risk of increased mortality and renal failure related to the use of HES solutions for infusion in these patients. The new data provided does not change the conclusions from the previous 2013 referral that the benefits of HES solutions for infusion do not outweigh the serious risks in patients with sepsis or critical illness.
- The PRAC also noted the overall exposure to HES solutions for infusion in the EU, estimated to about 1.5 to 2 million patients *per* year since 2014. In view of this exposure and the results from the two DUSs, the PRAC concluded that the estimated level of continued usage in populations where serious harm has been demonstrated raises important public health concerns, including a potentially increased mortality.
- The PRAC further acknowledged that the current clinical experience suggests that it is difficult to clearly separate patient populations where randomised clinical trials have shown serious harm from populations targeted by the approved indication. Patients in the approved indication may become critically ill or septic shortly after receiving HES solutions for infusion and these patients cannot be identified prospectively. This complicates effective risk minimisation in these patients.
- Furthermore, the PRAC considered options for measures to further mitigate these risks, including changes to the product information, direct health care professional communication, educational materials, warning on the primary container of the products, sign-in for medication form, prescription sheet/checklists, restricted access and distribution system to accredited hospitals/physicians. However, the available evidence shows that the non-adherence is not only due to a lack of awareness of the restrictions by prescribers, rendering further communication and education unlikely to be sufficiently effective. The medication form/checklists would also raise feasibility issues in an emergency setting, and implementation of a restricted access/distribution program is unlikely to be feasible and sufficiently effective across EU Member States considering the particular type of distribution and usage of HES solutions for infusion and some national limitations. The PRAC concluded that no additional risk minimisation measure or combination of risk minimisation measures, to sufficiently ensure safe and effective use of HES solutions for infusion could be identified.

In view of the above, the PRAC concluded that pursuant to Article 116 of Directive 2001/83/EC the risks related to the use of HES outweigh their benefits and thus the benefit-risk balance of HES solutions for infusion is no longer favourable.

Therefore, the PRAC recommends the suspension of the marketing authorisations for all medicinal products referred to in Annex I.

For lifting the suspension, the MAHs should provide reliable and convincing evidence on a favourable benefit risk balance in a well-defined population, with feasible and effective measures to adequately minimise exposure of patients at an increased risk of serious harm.

Revised CMDh position

Having reviewed the revised PRAC recommendation[7], the CMDh disagreed with the PRAC overall conclusions and grounds for recommendation.

Detailed explanation of the scientific grounds for the differences from the PRAC recommendation

The CMDh took into consideration the revised PRAC recommendation to suspend the marketing authorisations for solutions for infusion adopted at May PRAC plenary meeting. The CMDh took also into consideration the responses to the questions raised by the European Commission and the elements collected by the PRAC, as well as the information provided by the MAH during the oral explanations held on 28 May 2018 and 25 June 2018.

- ***Impact on the clinical practice of potential suspension of MA for HES solutions for infusion***

The benefit of HES solutions for infusion has been demonstrated in terms of volume-sparing effect, and there is some support for effects on short-term hemodynamic effects, although there is some uncertainty to what extent this translates into more patient-relevant outcomes.

A positive benefit-risk balance has been established in elective surgery and trauma patients. As an outcome of the 2013 referral, post-authorisation studies were imposed to the MAHs in these clinical settings and are a condition to the terms of the MAs for HES solutions for infusion. These studies as well as on-going voluntary clinical studies (e.g. FLASH study) would further characterise the efficacy and safety profile in elective surgery and trauma patients. The CMDh emphasised the importance of having meaningful results from these studies as soon as possible. The CMDh noted the PRAC conclusions that no new safety data was provided that would change the conclusions on the safety profile established in the previous referrals for HES solutions for infusion.

The CMDh also noted that treatment characteristics in some of the previously assessed clinical studies such as strength, dose or length of treatment may differ from current practice.

The CMDh considered also the divergence of positions of the learned societies in the EU. The European Society of Anaesthesiology and some national learned societies, stated that HES solutions for infusion play a role in the therapeutic armamentarium of hypovolemic shock in patients who cannot be stabilised with crystalloids alone. On the contrary, the Scandinavian Society of Anaesthesiology and Intensive Care Medicine (SSAI) and five Scandinavian learned societies supported the suspension of the marketing authorisations for HES solutions for infusion.

These views often reflected the current national medical practice and the debate in the medical community.

The CMDh acknowledged the complexity of managing patient suffering from hypovolemia due to an acute blood loss, and the fact that these patients require an individual evaluation. It was also considered that HES solutions for infusion are used for the treatment of life-threatening medical conditions.

The CMDh took into consideration all the new elements available since the previous CMDh position. In particular, the CMDh noted the results of a consultation of Member States where the suspension of the

[7] PRAC Recommendation EMA/PRAC/1707/2018 Rev.1 and PRAC AR EMA/PRAC/808891/2017 Rev.1 Corr.1

marketing authorisation for HES solutions for infusion would have an impact on the clinical practice where HES is reported as an adequate therapeutic option.

The CMDh noted that eight EU Member States have shared concerns about a medical need created in case of suspension of the marketing authorisation for HES solutions for infusion. Whilst some concerns related to the use of these products in clinical settings not covered by the terms of the MA, the CMDh acknowledged concerns on the medical need related to the authorised use and therefore should be taken into account, even if it might be rare.

- ***Proposals for additional risk minimisation measures and pharmacovigilance activities***

The CMDh discussed whether measures to mitigate the risks associated with HES solutions for infusion, including in particular the risk of increased mortality and renal failure in critically ill patients and patients with sepsis would be effective and feasible, taking into account new elements provided by the MAHs and Member States. In particular, the CMDh took into consideration the additional details from the MAHs on the proposed controlled access program.

The CMDh is of the position that the proposed additional measures described below would be feasible and effective to sufficiently minimise the risks by increasing awareness of HCPs, as well as ensuring that access to HES solutions for infusion is reserved to HCPs that have received adequate training.

1. Amendments to be included in the product information

The DUSs results showed that approximately 9% of patients exposed to HES solutions for infusion were critically ill, approximately 5-8% of patients had renal impairment and approximately 3-4% of patients had sepsis. It is proposed to mention explicitly in the new warnings ‘renal impairment’ in addition to ‘sepsis’ and ‘critically ill patients’; this would add further emphasis on this specific critically ill patients.

> *(1) Addition of the following warning on the outer packaging and the immediate packaging: “Do not use in sepsis, renal impairment, or critically ill patients. See all contraindications in the SmPC.”*

The CMDh acknowledged that the prescribers would not often administer the solution and therefore could not see this warning label. However, this measure should be seen as part of the complete program of risk minimisation measures. Hence:

- This measure will act as a reminder at time of administration, which is important in an emergency setting. It will complement other measures such as training to HCPs.
- It will allow targeting HCPs in charge of the administration of the medicinal product who have an important role in ensuring adherence to the adequate use of HES solutions for infusion.
- The continued presence of this warning on the packaging will also contribute to long-term awareness of HPCs.
- A reference to section 4.3 of the SmPC will avoid undermining the importance of other contraindications, and ensure these continue to be adhered to.

In terms of readability, the warning should be strongly emphasised (e.g. capitalized, bold letters or use of colours). The warning and its visual details should be subject to a user test in line with the

"Guideline on the Readability of the labelling and package leaflet on medicinal products for human use", to be submitted within 1 month from the Commission Decision.

(2) Addition of a prominent warning at the top of the SmPC and PL.

In order to prompt the attention of the recipients of the product information on the clinical situations above described, a warning to not use HES solutions for infusion in septic, renal impaired and critically ill patients should be mentioned at the top of the SmPC and PL.

2. Controlled access program

The objective of such program is to ensure the delivery of HES solutions for infusion to only hospitals/centres where HCPs expected to prescribe or administer (hereinafter ‘relevant HCPs’) have been trained adequately on the appropriate use of HES solutions for infusion, irrespective of the department(s) where they operate,.

The CMDh noted the concerns raised by PRAC on some models of controlled access program for HES solutions for infusion, in particular regarding feasibility due to the difficulty to define relevant prescribers / departments / hospitals and their effectiveness.

The CMDh through its representatives of NCAs have further discussed the national specificities of the healthcare systems and was of the view that the proposed controlled access program is likely to be feasible for the following reasons:

- Whilst patients eligible to treatment with HES solutions for infusion cannot be restricted to specific hospitals or hospital departments, it is possible to identify within a hospital/centre the relevant HCPs who would be prescribing/administrating HES solutions for infusion.
- The emergency setting in which the products are used does not impede the implementation of a controlled access program based on the delivery of appropriate training to the relevant HCPs as the educational program can be organised and delivered well in advance of the use of the products.
- Whilst acknowledging the difficulties of continuous training, centralising such organisation and monitoring by the MAH will address the challenges raised.

Therefore, the CMDh is of the position that each MAH shall be responsible for the implementation and the supervision of the access controlled program as follows:

- The MAH should develop the training materials for relevant HCPs expected to prescribe / administer HES solutions for infusion and agree the exact content and format of these materials with the relevant national competent authorities.
- The MAH should deliver the training on the appropriate use of HES solutions for infusion to relevant HCPs on a regular basis, as agreed with the national competent authorities. The MAH should also ensure these HCPs are provided with the following:
    - The Summary of product characteristics,
    - Training materials.
- The MAH should manage the accreditation of the hospitals / centres, ensuring that all relevant HCPs intended to prescribe/ administer HES solutions for infusion have been adequately trained. This includes recordings of trainings and accreditation.

- The MAH should ensure that HES solutions for infusion are only delivered to accredited hospitals/centres.

The above training materials should be based on the following key elements:

- the risks related to the use of HES solutions for infusion outside the terms of the MA,
- a reminder of the indication, dose, duration of treatment and contraindications and the need to comply with the product information,
- the new additional risk minimisation measures,
- the results from the DUSs.

The training materials should be based on interactive learning tools in order to ensure the active involvement of the HCPs.

The training materials should be distributed to all relevant HCPs intended to prescribe/administer HES solutions for infusion (e.g. anaesthiologists, intensive care physicians, nurses…).

In order to optimise the uptake by HCPs and their adherence to the adequate conditions of use of HES solutions for infusion, the learned societies should be involved in the development and distribution of such training materials.

The final training materials, including communication media and distribution modalities should be agreed with the National Competent Authorities.

The CMDh finally encouraged the integration of the training activities abovementioned in the continuing medical education at national level.

In view of the above and whilst noting the PRAC's reservations on the expected effectiveness of this measure, notably in view of the non-adherence by prescribers not only due to a lack of awareness, the CMDh considered that:

- The assumption that the non-adherence is not only due to lack of awareness is not supported by sufficient evidence and might not be representative of a significant portion of the prescribers population.
- This controlled access program is a key measure to ensure adherence of HCPs to the terms of the MA by increasing both the awareness of the relevant HCPs to the risks associated to the use of HES solutions for infusions and ensuring that they have received appropriate training before they can use HES solutions for infusion. It is expected that the involvement of the learned societies will have an important role in channelling the messages of the training. The effectiveness of this measure should be considered in combination with other measures.
- This measure will ensure long term effectiveness through reminders sent to the trained HCPs and training of new ones. The frequency of the training / reminders should be discussed at national level taking consideration particularities of each national healthcare system.
- In view of the seriousness of the risks related to the use of HES solutions for infusion outside the terms of the MA, the CMDh considered this measure as proportionate.

In conclusion, the CMDh is of the position that a controlled access program is feasible and likely to be effective to minimise the risk, in combination with other risk minimization measures.

This controlled access program should be described in a Risk Management Plan which shall be submitted to the National Competent Authorities for assessment within 3 months from the Commission Decision.

The details of the controlled access program, the modalities of its implementation and the final training materials, including communication media and distribution modalities should be agreed with the National Competent Authorities.

Having considered the specificities of the national systems and the need for agreement with the national competent authorities on the details on the implementation of the controlled access and the time required to complete the adequate training to all HCPs intended to use HES products and the accreditation of the hospitals/ centres, the CMDh requested that the controlled access program should be effectively implemented at the latest 9 months after Commission Decision.

3. Communication measures

Acknowledging the need of increasing the compliance to the indication and contraindications, the CMDh is of the position that a targeted communication through DHPC would be effective to this aim.

Indeed, the CMDh noted the results of the DUSs whereby there was adherence to some of the key restrictions of use recommended following the 2013 referrals (i.e. the maximum daily dose and treatment duration). In addition, a significant decrease of the usage of HES solutions for infusion was noted in most EU Member States. This suggests that the previous communication measures although not sufficient to ensure full compliance, have had some effectiveness. It is therefore considered that a DHPC with more targeted messages and with the involvement of the learned societies in the distribution of this DHPC is likely to reinforce the effectiveness of this measure.

Besides, the CMDh acknowledged the PRAC's concern that non-adherence would not only be due to a lack of awareness by prescribers of the restrictions imposed to HES solutions for infusion. However, the CMDh considered that this assumption is not supported by sufficient evidence and might not be representative of a significant portion of the prescribers population and that in any case, the learned societies will have an important role in channelling the messages of the DHPC.

Therefore, the CMDh adopted a DHPC to inform HCPs of the results from the DUSs, the terms of the MA and the risks related to the use of HES solutions for infusion outside these terms, as well as the new additional risk minimisation measures. The CMDh also agreed on a communication plan for the dissemination of this DHPC.

As an overall conclusion, the CMDh considered that the risk minimisation described above would be feasible and effective and will have a synergistic effect as they allow targeting specific HCPs at all the steps of the prescription and administration of HES solutions for infusion.

4. Drug Utilisation Study (DUS)

In addition, the CMDh is of the view that the MAHs shall perform a Drug Utilisation Study, in order to assess the effectiveness of the new measures recommended. The conduct of this study will be a condition to the marketing authorisations for HES solutions for infusion.

The DUS protocol to be submitted by the MAHs should include clear objectives, focusing on prescribing decisions, especially adherence to indications and contraindications. Protocols should include a representative sample of EU Member States. They should also allow adequate description of national contexts to guarantee the adequate analysis and extrapolation of the outcome and justify potential

adjustments (i.e. successful/unsuccessful distribution of safety communications, adherence to the controlled access program, qualitative feedback from prescribers, etc). For this protocol, the MAH should take into account the experience gathered from the previously conducted DUS. The protocol should also include a measure of the primary outcomes at baseline. The primary outcomes should be common to all studied EU Member States.

The DUS protocol should be submitted for assessment to the PRAC within 3 months of the Commission Decision.

Progress on the ongoing DUS shall be reported within the upcoming PSUR. The DUS final study report shall be submitted within 24 months from the Commission Decision.

Grounds for differences with the PRAC recommendation

Whereas

- The CMDh took into consideration the revised PRAC recommendation as well as all the new elements submitted by the MAHs and the Member States regarding potential medical need and the feasibility and likely effectiveness of additional risk minimisation measures and available since the previous CMDh position adopted in January 2018.
- In particular, the CMDh noted the results of a consultation of Member States where the suspension of the marketing authorisation for HES solutions for infusion would have an impact on the clinical practice and concerns raised by those EU Member States about a potential medical need.
- The CMDh, through its representatives of Member States also considered feasibility at national level of some additional risk minimisation measures for which PRAC raised feasibility questions. In view of the insight that the CMDh has on the national healthcare systems, it agreed that the following additional risk minimisation measures are feasible and likely to be effective to minimise the risk of using HES solutions for infusion in contraindicated populations: the inclusion of an emphasised warning on the SmPC, PL, primary and secondary packaging, the circulation of a targeted DHPC and the implementation of a controlled access program.
- The CMDh also considered that the effectiveness of these additional risk minimisation measures must be assessed through the conduct of a drug utilisation study.

As a consequence, the CMDh is of the position that the benefit-risk balance of HES solutions for infusion remains favourable subject to the agreed amendments to the product information and the conditions to the marketing authorisation. The CMDh also concluded that the annual PSUR cycle should remain unchanged and would enable periodic review of the benefit-risk balance of these products and the impact of any data that would be generated.

Therefore the CMDh recommends the variation to the terms of the marketing authorisations for HES solutions for infusion.
