# Supplementary material for: Starch Wars—New Episodes of the Saga. Changes in Regulations on Hydroxyethyl Starch in the European Union
Source: Front Vet Sci. 2019 Jan 18;5:336. doi: 10.3389/fvets.2018.00336 (PMC6345713; doi:10.3389/fvets.2018.00336)
Supplement: Supplementary file 4 [file Data_Sheet_4.PDF]

1600 20th Street, NW • Washington, D.C. 20009 • 202/588-1000 • www.citizen.org

Citizen Petition

Date: February 8, 2017

On behalf of Public Citizen, a consumer advocacy organization with more than 400,000 members and supporters nationwide, the undersigned submit this petition under Section 355(e) of the Federal Food, Drug, and Cosmetic Act and under Food and Drug Administration (FDA) regulations at 21 C.F.R. § 10.30 to request the Commissioner of Food and Drugs to immediately require the removal from the market of hydroxyethyl starch (HES) intravenous (IV) solutions because (1) the solutions' risks outweigh their limited benefits and (2) there are a number of other, safer alternative IV solutions for the uses for which HES solutions are approved.

A. ACTION REQUESTED

Immediately require the removal from the market of HES IV solutions.

B. STATEMENT OF GROUNDS

1. Background

a. About HES

There are two main categories of IV solutions: crystalloid and colloid. Crystalloid solutions contain water; necessary minerals or salts, such as sodium chloride; and sometimes glucose. Colloidal solutions, including HES, have largely the same content as crystalloid solutions but with added protein, starch, or other large molecules in varying amounts.

HES solutions are synthetic carbohydrate polymers derived from maize (corn) starches that are approved by the FDA for the treatment of hypovolemia.[1] HES solutions are marketed in three different molecular weights and vary in their molar substitution ratio (**Table 1**).[2]

---

[1] Food and Drug Administration. Laurence Landow, MD, Medical Officer with the Center for Biologics Evaluation and Research. Regulatory Overview of Hydroxyethyl Starch (HES) Solutions. September 6, 2012. http://www.fda.gov/downloads/BiologicsBloodVaccines/NewsEvents/WorkshopsMeetingsConferences/UCM325456.ppt. Accessed January 4, 2017.

[2] The molar substitution ratio refers to the average number of hydroxyethyl groups per unit of glucose within the molecule. Both molar substitution ratios and molecular weight are associated with rates of metabolism and clearance, with lower molecular weights and molar substitution ratios thought to result in more rapid rates of hydrolysis by serum amylase and consequently more rapid plasma clearance. (Sources: von Roten I, Madjdpour C, Frascarolo P, et al. Molar substitution and C2/C6 ratio of hydroxyethyl starch: influence on blood coagulation. *Br J Anaesth*. 2006;96(4):455-463; and Food and Drug Administration. September 23, 2014 Pediatric Advisory Committee memorandum. NDA BN070012: Voluven. http://www.fda.gov/downloads/AdvisoryCommittees/CommitteesMeetingMaterials/PediatricAdvisoryCommittee/U

Table 1. HES solutions currently marketed in the U.S.[3,4]

Brand Name	FDA Approval	Molecular Weight (kilodaltons)	Molar Substitution Ratio	Half-Life (hours)
**Hespan** (and generic equivalents)	1972	600	0.7	46
**Hextend**	1999	670	0.7	46
**Voluven**	2007	130	0.4	12

b. Early history of HES solutions

The first HES solution approved by the FDA, in 1972, was HES 600/0.7 (Hespan).[5] The studies that supported its approval would likely have been deemed inadequate by today's regulatory standards. These trials were uncontrolled, with a small number of subjects and short periods of observation (less than 24 hours).[6] Data submitted to support the subsequent approval of newer HES products were similarly inadequate to evaluate safety, based as they were on non-inferiority studies with older (insufficiently studied) HES and gelatin solutions as the comparators.[7] Gelatin was withdrawn from the U.S. market in 1978 due to safety concerns over increased blood viscosity and impaired blood coagulation.[8]

CM414088.pdf. Accessed January 9, 2017.) However, this does not seem to translate into lower rates of tissue deposition, with low-molecular-weight and low-molar-substitution-ratio formulations of HES displaying higher rates of tissue uptake than their high-molecular-weight/molar-substitution-ratio counterparts in a 2012 systematic review of studies assessing HES pharmacokinetics in the first 24 hours after infusion. (Source: Bellmann R, Feistritzer C, Wiedermann CJ. Effect of molecular weight and substitution on tissue uptake of hydroxyethyl starch: a meta-analysis of clinical studies. *Clin Pharmacokinet*. 2012;51(4):225-236.)

[3] Food and Drug Administration. September 23, 2014 Pediatric Advisory Committee memorandum. NDA BN070012: Voluven. http://www.fda.gov/downloads/AdvisoryCommittees/CommitteesMeetingMaterials/PediatricAdvisoryCommittee/UCM414088.pdf. Accessed January 4, 2017.

[4] Food and Drug Administration. Center for Biologics Evaluation and Research. Currently Approved CBER NDAs/ANDAs, by applicant as of January 3, 2017. http://www.fda.gov/downloads/BiologicsBloodVaccines/BloodBloodProducts/ApprovedProducts/NewDrugApplicationsNDAs/UCM149972.pdf. Accessed February 3, 2017.

[5] *Ibid*.

[6] Hartog CS, Natanson C, Sun J, et al. Concerns over use of hydroxyethyl starch solutions. *BMJ*. 2014;349:g5981.

[7] Food and Drug Administration. NDA Review Memo (Mid-Cycle). BN070012: 6% hydroxyethyl starch 130/0.4 in 0.9% sodium chloride. March 6, 2007. http://www.fda.gov/downloads/BiologicsBloodVaccines/BloodBloodProducts/ApprovedProducts/NewDrugApplicationsNDAs/UCM083393.pdf. Accessed January 4, 2017. PDF pp. 3-4.

[8] Food and Drug Administration. List of drug products that have been withdrawn or removed from the market for reasons of safety or effectiveness. 63 FR 54082. October 8, 1998. http://www.fda.gov/ohrms/dockets/98fr/100898b.txt. Accessed January 4, 2017.

Soon after Hespan was approved in 1972, serious concerns about its safety, related to coagulation interference[9] and uptake in tissue,[10] were reported. Later in the 1990s, evidence of renal abnormalities associated with HES solution use were also reported.[11,12] By 2001, French guidelines for use of HES products had been expanded to include restrictions on dosing and treatment duration, among other measures, in response to reports of hemostatic disorders (acquired type I von Willebrand's disease) in patients with subarachnoid hemorrhage who had received pentastarch formulations of HES.[13] The same year, a randomized trial was published showing a higher risk of acute kidney failure with HES solutions compared with gelatin-based IV fluids in patients with severe sepsis or septic shock.[14] In August 2003, the FDA approved the addition of a warning to the labeling of one HES product, 6% hetastarch (Hespan), that advised against using the product in cardiopulmonary bypass surgery patients during the intraoperative and immediate post-operative periods because of an increased risk of coagulation abnormalities and bleeding in patients whose coagulation status is already impaired.[15]

c. Recent EMA actions

Beginning in 2008, pivotal evidence began to emerge that even more definitively demonstrated the dangers of HES products. Over the next four years, three large multicenter randomized clinical trials were published showing increased rates of renal failure, bleeding, and mortality associated with HES solution use in critically ill patients, including those with sepsis.[16,17,18,19]

In response to these findings, in 2012, the German Federal Institute for Drugs and Medical Devices requested that the European Medicines Agency's (EMA's) Pharmacovigilance Risk Assessment Committee (PRAC) review the risks and benefits of using HES.[20] In June 2013, the PRAC recommended that the marketing authorizations for HES products be suspended because

---

[9] Alexander B, Odake K, Lawlor D, Swanger M. Coagulation, hemostasis, and plasma expanders: a quarter century enigma. *Fed Proc*. 1975;34(6):1429-1440.

[10] Bellmann R, Feistritzer C, Wiedermann CJ. Effect of molecular weight and substitution on tissue uptake of hydroxyethyl starch: a meta-analysis of clinical studies. *Clin Pharmacokinet*. 2012;51(4):225-236.

[11] Legendre C, Thervet E, Page B, et al. Hydroxyethylstarch and osmotic-nephrosis-like lesions in kidney transplantation. *Lancet*. 1993;342(8865):248-249.

[12] Cittanova ML, Leblanc I, Legendre C, et al. Effect of hydroxyethylstarch in brain-dead kidney donors on renal function in kidney-transplant recipients. *Lancet*. 1996;348(9042):1620-1622.

[13] Jonville-Béra AP, Autret-Leca E, Gruel Y. Acquired type I von Willebrand's disease associated with highly substituted hydroxyethyl starch. *N Engl J Med*. 2001;345(8):622-623.

[14] Schortgen F, Lacherade JC, Bruneel F, et al. Effects of hydroxyethylstarch and gelatin on renal function in severe sepsis: a multicentre randomised study. *Lancet*. 2001;357(9260):911-916.

[15] Haynes GR, Havidich JE, Payne KJ. Why the Food and Drug Administration changed the warning label for hetastarch. *Anesthesiology*. 2004;101(2):560-561.

[16] Hartog CS, Natanson C, Sun J, et al. Concerns over use of hydroxyethyl starch solutions. *BMJ*. 2014;349:g5981.

[17] Myburgh JA, Finfer S, Bellomo R, et al. Hydroxyethyl starch or saline for fluid resuscitation in intensive care. *N Engl J Med*. 2012;367(20):1901-1911.

[18] Brunkhorst FM, Engel C, Bloos F, et al. Intensive insulin therapy and pentastarch resuscitation in severe sepsis. *N Engl J Med*. 2008;358(2):125-139.

[19] Perner A, Haase N, Guttormsen AB, et al. Hydroxyethyl starch 130/0.42 versus Ringer's acetate in severe sepsis. *N Engl J Med*. 2012;367(2):124-134.

[20] European Medicines Agency. Notification of a referral under article 31 of Directive 2001/83/EC. November 20, 2012. http://www.ema.europa.eu/docs/en_GB/document_library/Referrals_document/Solutions_for_infusion_containing_hydroxyethyl_starch/Procedure_started/WC500135590.pdf. Accessed January 4, 2017.

its review found that the benefits of HES solutions no longer outweighed their risks.[21] The PRAC stated the following in explaining its decision:[22]

> The PRAC was of the opinion that, when compared with crystalloids, patients treated with HES were at a greater risk of kidney injury requiring dialysis and had a greater risk of mortality.

The PRAC further noted "that the **available data only showed a limited benefit of HES in hypovolaemia**, which did not justify its use considering the known risks" [emphasis added]. It is noteworthy that the PRAC did not find any compelling evidence that the benefit-risk profile of HES solutions was different in surgical and trauma patients, concluding that HES products should remain banned "unless the marketing authorisation holder **can provide convincing data to identify a group of patients in whom the benefits of the medicines outweigh their risks**" [emphasis added].

However, several HES solution manufacturers requested a re-examination of this PRAC recommendation.[23] In addition, the United Kingdom's Medicines and Healthcare products Regulatory Agency notified the EMA member states of its consideration of the need to remove HES solutions from the U.K. market.[24] Because of both of these actions, a second PRAC committee was convened to reanalyze the evidence. In October 2013, the committee reversed the initial recommendation to completely ban HES solutions and recommended that HES solutions remain on the market for use "in patients with hypovolemia caused by acute blood loss where treatment with alternative infusions [*sic*] solutions known as 'crystalloids' alone are not considered to be sufficient."[25]

Only 19 of the 33 committee members, however, voted to reverse the original decision.[26] The 14 dissenting members articulated their arguments for maintaining the original decision to ban HES solutions in documents known as "divergent statements" at the end of the EMA's publicly released review of its decision (see Appendix 1).[27] These 14 members based their decision on four main arguments:

---

[21] European Medicines Agency. PRAC recommends suspending marketing authorisations for infusion solutions containing hydroxyethyl starch. June 14, 2013. http://www.ema.europa.eu/ema/index.jsp?curl=pages/news_and_events/news/2013/06/news_detail_001814.jsp&mid=WC0b01ac058004d5c1. Accessed January 4, 2017.
[22] *Ibid.*
[23] European Medicines Agency. Assessment report for solutions for infusion containing hydroxyethyl starch. November 11, 2013. http://www.ema.europa.eu/docs/en_GB/document_library/Referrals_document/Hydroxyethyl_starch-containing_medicines_107/Recommendation_provided_by_Pharmacovigilance_Risk_Assessment_Committee/WC500154254.pdf. Accessed January 4, 2017. PDF pp. 3-4.
[24] *Ibid.*
[25] European Medicines Agency. PRAC confirms that hydroxyethyl-starch solutions (HES) should no longer be used in patients with sepsis or burn injuries or in critically ill patients. October 11, 2013. http://www.ema.europa.eu/docs/en_GB/document_library/Referrals_document/Solutions_for_infusion_containing_hydroxyethyl_starch/Recommendation_provided_by_Pharmacovigilance_Risk_Assessment_Committee/WC500151963.pdf. Accessed January 5, 2017.
[26] Hartog CS, Natanson C, Sun J, et al. Concerns over use of hydroxyethyl starch solutions. *BMJ*. 2014;349:g5981.
[27] European Medicines Agency. Assessment report for solutions for infusion containing hydroxyethyl starch. November 11, 2013.

- First, that "the evidence to demonstrate a lower risk of renal injury and mortality in any settings other than sepsis and the critically ill is weak."
- Second, that "the data evaluating benefit in the perioperative setting and trauma setting do not adequately support a clinically relevant beneficial effect for HES."
- Third, that there exists "an overlap between those patient groups where the benefit-risk balance of HES is judged to be negative (septic and critically ill patients), and the patients who will receive HES under the revised indication allowing use in patients with hypovolaemia due to acute bleeding (e.g. including the trauma and perioperative settings)."
- Fourth, that "[t]he mechanism underlying the risks of renal injury and mortality observed with HES is not well understood, and therefore these should be considered to be potential risks in all patient groups."

In response to the EMA's decision to reverse the ban, 76 globally recognized researchers in intensive care and other disciplines sent an open letter to the executive director of the EMA expressing concern about the EMA's decision and the risk of harm to which patients treated with HES products would be exposed (see Appendix 2).[28] They posed the following, fundamental question to the PRAC: "What assumptions or clinical data would indicate that the same pathological mechanisms do not apply in patients with hypovolaemia from blood loss?" The signatories also pointed out that the PRAC's recommendations to monitor kidney function in patients for at least 90 days after HES solution administration and not to use HES solutions for more than 24 hours are insufficient to prevent adverse effects.

d. Recent FDA actions

For its part, the FDA held a public workshop on September 6-7, 2012, to discuss the evidence on the risks and benefits of HES products.[29] After reviewing data presented at this public workshop, the FDA, like the EMA's PRAC, concluded that HES solutions should not be used in critically ill patients, including patients with sepsis and those admitted to an intensive care unit. The FDA announced in June 2013 a new black-box warning in the labeling for all HES products about the risk of mortality and severe renal injury in these patient populations.[30] (The FDA issued an updated, more detailed communication in November 2013.[31]) The FDA based this decision

---

http://www.ema.europa.eu/docs/en_GB/document_library/Referrals_document/Hydroxyethyl_starch-containing_medicines_107/Recommendation_provided_by_Pharmacovigilance_Risk_Assessment_Committee/WC500154254.pdf. Accessed January 4, 2017. PDF pp. 36-38, 40-41.

[28] Bellomo R, Bion J, Finfer S, et al. Open letter to the Executive Director of the European Medicines Agency concerning the licensing of hydroxyethyl starch solutions for fluid resuscitation. *Br J Anaesth*. 2014;112(3):595-600.

[29] Food and Drug Administration. Public workshop: Risks and benefits of hydroxyethyl starch solutions. September 6-7, 2012. http://www.fda.gov/BiologicsBloodVaccines/NewsEvents/WorkshopsMeetingsConferences/ucm313370.htm. Accessed January 5, 2017.

[30] Food and Drug Administration. Hydroxyethyl starch solutions: FDA safety communication — boxed warning on increased mortality and severe renal injury and risk of bleeding. June 11, 2013. http://www.fda.gov/Safety/MedWatch/SafetyInformation/SafetyAlertsforHumanMedicalProducts/ucm358349.htm. Accessed January 5, 2017.

[31] Food and Drug Administration. FDA safety communication: boxed warning on increased mortality and severe renal injury, and additional warning on risk of bleeding, for use of hydroxyethyl starch solutions in some settings.

largely on the same evidence as that relied on by the EMA's PRAC, including increased mortality and need for RRT associated with HES solutions seen in three randomized controlled trials (the CRYSTMAS, 6S, and CHEST trials) and several meta-analyses.

In the same safety communication, the FDA noted that the agency had required an additional label warning of excessive bleeding associated with use of HES products in patients undergoing cardiopulmonary bypass,[32] based on a meta-analysis published in 2012.[33] However, the FDA opted not to extend its warning against the use of Hespan in these patients to all HES products.[34] Finally, in its 2013 communication, the FDA advised that use of HES products in patients with severe liver disease was contraindicated and recommended monitoring of liver function in all patients receiving HES products.[35]

e. Current, unnecessary use of HES

According to IMS data cited in an August 25, 2014, memorandum prepared for the September 23, 2014, meeting of the FDA's Pediatric Advisory Committee, an estimated 187,025 U.S. patients were treated with one HES product, Voluven, between March 2009 and December 31, 2013; four-fifths (149,620 of 187,025) of these were surgical patients.[36] Importantly, according to IMS data compiled by CSL Behring, the use of HES solutions declined considerably in the U.S. from the first quarter of 2011 (249,000 units sold) to the final quarter of 2013 (81,000 units), with the largest drop in sales occurring during the quarter following the FDA's initial June 2013 safety communication.[37]

Moreover, the dissenters from the PRAC's decision had pointed out that HES products are certainly not essential for good medical care, citing "a survey of 391 ICUs [intensive care units] worldwide conducted in 2010 (Finfer et al, 2010) [that] showed no use of HES [in the centers surveyed] in the United States or Australia."[38] As **Table 2** shows, there are several alternative IV

November 25, 2013. http://www.fda.gov/BiologicsBloodVaccines/SafetyAvailability/ucm358271.htm. Accessed January 5, 2017.

[32] *Ibid.*

[33] Navickis RJ, Haynes GR, Wilkes MM. Effect of hydroxyethyl starch on bleeding after cardiopulmonary bypass: A meta-analysis of randomized trials. *J Thorac Cardiovasc Surg* 2012:144(1):223-230.

[34] Food and Drug Administration. FDA safety communication: Boxed warning on increased mortality and severe renal injury, and additional warning on risk of bleeding, for use of hydroxyethyl starch solutions in some settings. November 25, 2013. http://www.fda.gov/BiologicsBloodVaccines/SafetyAvailability/ucm358271.htm. Accessed January 5, 2017.

[35] *Ibid*.

[36] Food and Drug Administration. September 23, 2014 Pediatric Advisory Committee memorandum. NDA BN070012: Voluven. http://www.fda.gov/downloads/AdvisoryCommittees/CommitteesMeetingMaterials/PediatricAdvisoryCommittee/UCM414088.pdf. Accessed January 5, 2017.

[37] CSL Behring. HES sales analysis: U.S., China & EU5. March 27, 2014. Based on IMS Health data, upon payment.

[38] European Medicines Agency. Assessment report for solutions for infusion containing hydroxyethyl starch. November 11, 2013. p. 36. http://www.ema.europa.eu/docs/en_GB/document_library/Referrals_document/Hydroxyethyl_starch-containing_medicines_107/Recommendation_provided_by_Pharmacovigilance_Risk_Assessment_Committee/WC500154254.pdf. Accessed January 4, 2017. Citing: Finfer S, Liu B, Taylor C, et al. Resuscitation fluid use in critically ill adults: an international cross-sectional study in 391 intensive care units. *Crit Care*. 2010;14(5):R185.

solutions to HES products for surgical and trauma patients that do not carry the same serious safety concerns and are just as effective at achieving desired clinical outcomes.

Table 2. Major IV solutions on the U.S. market.

Crystalloids
Saline
Dextrose
Ringer's solutions
Colloids
Albumin
Dextran
HES

2. Potential mechanisms for HES toxicity

After infusion and distribution into plasma, HES undergoes hydrolysis and renal excretion.[39] However, it has been known for decades that HES molecules are also deposited in various bodily tissues and organs after infusion,[40] in some cases for years.[41] Within the intracellular space, HES molecules are incorporated into lysosomes and become resistant to subsequent degradation.[42]

A 2014 systematic review assessing the accumulation of HES in tissues included 37 human studies with a total of 615 patients and 11 animal studies.[43] Among human studies, a large proportion of subjects (46%) were surgical patients. HES deposition was found mainly in the skin (17 studies), kidney (12), liver (8), and bone marrow (5). Other organs involved were the lymph nodes, spleen, lung, pancreas, intestine, muscle, trophoblast, and placental stroma. HES deposition was rapid, occurring within 30 minutes in the liver and 90 minutes in the skin in two studies. It was also cumulative, increasing in proportion to dose, though 15% of patients had evidence of product storage even at the lowest cumulative doses (0.4 grams per kilogram). HES deposits were found in the skin 4-8 years after exposure. HES-related symptoms can persist for years as a result; one patient continued to suffer from pruritis and disfiguring periocular edema more than 4 years after exposure. In another study, osmotic "nephrosis-like lesions" persisted for an average of 6.4 years after exposure in liver transplant patients.

[39] Food and Drug Administration. September 23, 2014, Pediatric Advisory Committee memorandum. NDA BN070012: Voluven. http://www.fda.gov/downloads/AdvisoryCommittees/CommitteesMeetingMaterials/PediatricAdvisoryCommittee/UCM414088.pdf. Accessed January 5, 2017.
[40] Bellmann R, Feistritzer C, Wiedermann CJ. Effect of molecular weight and substitution on tissue uptake of hydroxyethyl starch: a meta-analysis of clinical studies. *Clin Pharmacokinet*. 2012;51(4):225-236.
[41] Wiedermann CJ, Joannidis M. Accumulation of hydroxyethyl starch in human and animal tissues: a systematic review. *Intensive Care Med*. 2014;40(2):160-170.
[42] Bellmann R, Feistritzer C, Wiedermann CJ. Effect of molecular weight and substitution on tissue uptake of hydroxyethyl starch: a meta-analysis of clinical studies. *Clin Pharmacokinet*. 2012;51(4):225-236.
[43] Wiedermann CJ, Joannidis M. Accumulation of hydroxyethyl starch in human and animal tissues: a systematic review. *Intensive Care Med*. 2014;40(2):160-170.

The review found that HES tissue accumulation was associated with various adverse effects, including pruritis, bleeding, renal failure, liver dysfunction, and bone marrow suppression. In one randomized trial involving renal transplant subjects, the likelihood of requiring renal replacement therapy in the first eight days post-transplant was more than sixfold higher in transplant recipients who received kidneys from brain-dead donors infused with HES 200/0.62 plus gelatin solution prior to organ harvest compared with recipients who received kidneys from donors infused with gelatin only.[44] Three recipients of kidneys from HES-plus-gelatin-infused donors and six recipients of kidneys from gelatin-only-infused donors underwent biopsies of the donor kidney within six weeks of transplantation. All three kidneys from HES-plus-gelatin-infused donors exhibited osmotic nephrosis, a finding not seen in any kidney from the gelatin-only-infused donors.

It had been thought that newer formulations of HES solutions with lower molecular weight and molar substitution ratios presented less of a risk of tissue uptake than did older formulations. However, a 2012 systematic review of 25 studies presenting data on HES tissue uptake found that both low-molecular-weight and lower-molar-substitution HES solutions actually had significantly higher rates of tissue uptake during the first 24 hours after infusion than high-molecular-weight and higher-molar-substitution solutions, respectively.[45] A 2014 *in vitro* study also found that all molecular weights of HES led to significantly reduced proximal tubular kidney cell viability compared with control fluids.[46]

Furthermore, there is physiologic evidence that low-molecular-weight HES may, in fact, be more likely to be deposited in the kidney than high-molecular-weight HES. The primary site of kidney uptake of HES is the proximal tubule.[47] Only HES molecules below 45-60 kilodaltons (kDA) can pass through the glomerular filtration barrier and be taken up by the proximal tubular cells, leading to osmotic nephrosis and long-term storage.[48]

Thus, it has been demonstrated that both high- and low-molecular-weight HES products accumulate in multiple bodily tissues and organ systems, in some cases for years, and that this tissue storage predisposes patients to serious and long-term side effects.

3. Responses to main arguments in favor of the continued use of HES solutions

a. *Argument #1 (The FDA's rationale): The unfavorable risk-benefit profile of HES solutions is limited to critically ill patients, including those with sepsis, and does not apply to surgical and trauma patients*

---

[44] *Ibid.* Citing: Cittanova ML, Leblanc I, Legendre C, et al. Effect of hydroxyethylstarch in brain-dead kidney donors on renal function in kidney-transplant recipients. *Lancet*. 1996;348(9042):1620-1622.

[45] Bellmann R, Feistritzer C, Wiedermann CJ. Effect of molecular weight and substitution on tissue uptake of hydroxyethyl starch: a meta-analysis of clinical studies. *Clin Pharmacokinet*. 2012;51(4):225-236.

[46] Bruno RR, Neuhaus W, Roewer N, et al. Molecular size and origin do not influence the harmful side effects of hydroxyethyl starch on human proximal tubule cells (HK-2) in vitro. *Anesth Analg*. 2014;119:570-577.

[47] Wiedermann CJ, Joannidis M. Accumulation of hydroxyethyl starch in human and animal tissues: a systematic review. *Intensive Care Med*. 2014;40(2):160-170.

[48] *Ibid.*

The FDA opted to keep HES solutions on the market as it concluded that the safety concerns seen in critically ill patients, including those with sepsis (see Appendix 3), had not been definitively shown to apply to surgical and trauma patients. Because the agency has not released a full accounting of its rationale to the public, we relied solely on the 2013 safety communication to determine the data on which the FDA based this decision.[49] The FDA cited three meta-analyses[50,51 ,52] and a retrospective observational study[53] evaluating HES solutions that included surgical or trauma patients without sepsis and who were not critically ill, the only patient populations for which HES solutions remain indicated.

One of the three meta-analyses (Van Der Linden et al.) is reviewed in detail later in this section. Another of the three, of 18 randomized controlled trials, found increased risks of bleeding and need for blood transfusion in surgical subjects treated with HES solutions compared to those given the colloidal solution albumin, with no significant differences in risk between high- and low-molecular-weight HES solutions.[54] The retrospective observational study showed an increased risk of acute kidney injury in surgical patients.[55]

The sole study cited by the FDA in its Safety Communication that found no increased risk of adverse events from HES solutions in surgical or trauma patients without sepsis or critical illness, in contrast to the increased risk seen in critically ill or septic patients, was a 2010 Cochrane meta-analysis of randomized controlled trials of HES solutions.[56] That study found that an increased risk of adverse renal outcomes was limited to septic subjects and was not seen in HES-treated trauma or surgery subjects (although the authors noted that this latter finding may have been due to inadequate power to detect a risk).

However, that meta-analysis has since been updated by Mutter et al. in 2013.[57] A key difference between the two reviews was that, unlike the 2010 review, the 2013 update excluded nine studies (seven of which had been retracted) authored by Joachim Boldt,[58] the discredited researcher

---

[49] Food and Drug Administration. FDA safety communication: Boxed warning on increased mortality and severe renal injury, and additional warning on risk of bleeding, for use of hydroxyethyl starch solutions in some settings. November 25, 2013. http://www.fda.gov/BiologicsBloodVaccines/SafetyAvailability/ucm358271.htm. Accessed January 5, 2017.

[50] Van Der Linden P, James M, Mythen M, Weiskopf RB. Safety of modern starches used during surgery. *Anesth Analg*. 2013;116(1):35-48.

[51] Dart AB, Mutter TC, Ruth CA, Taback SP. Hydroxyethyl starch (HES) versus other fluid therapies: effects on kidney function. *Cochrane Database Syst Rev*. 2010;1:CD007594.

[52] Navickis RJ, Haynes GR, Wilkes MM. Effect of hydroxyethyl starch on bleeding after cardiopulmonary bypass: A meta-analysis of randomized trials. *J Thorac Cardiovasc Surg.* 2012;144(1):223-230.

[53] Rioux JP, Lessard M, De Bortoli B, et al. Pentastarch 10% (250 kDa/0.45) is an independent risk factor of acute kidney injury following cardiac surgery. *Crit Care Med*. 2009;37(4):1293-1298.

[54] Navickis RJ, Haynes GR, Wilkes MM. Effect of hydroxyethyl starch on bleeding after cardiopulmonary bypass: A meta-analysis of randomized trials. *J Thorac Cardiovasc Surg.* 2012;144(1):223-230.

[55] Rioux JP, Lessard M, De Bortoli B, et al. Pentastarch 10% (250 kDa/0.45) is an independent risk factor of acute kidney injury following cardiac surgery. *Crit Care Med*. 2009;37(4):1293-1298.

[56] Dart AB, Mutter TC, Ruth CA, Taback SP. Hydroxyethyl starch (HES) versus other fluid therapies: effects on kidney function. *Cochrane Database Syst Rev*. 2010;1:CD007594.

[57] Mutter TC, Ruth CA, Dart AB. Hydroxyethyl starch (HES) versus other fluid therapies: effects on kidney function. *Cochrane Database Syst Rev*. 2013;7:CD007594.

[58] *Ibid*.

accused of falsifying data and other wrongdoing in studies of HES solutions.[59] The authors of the updated review (described in more detail below) concluded unequivocally: "The current evidence suggests that all HES products increase the risk [of acute kidney injury] and [renal replacement therapy] in **all** patient populations and a safe volume of any HES solution has yet to be determined. In most clinical situations it is likely that these risks outweigh any benefits, and alternate volume replacement therapies should be used in place of HES products."[60] [emphasis added]

Therefore, the FDA did not offer sufficient justification, in its 2013 Safety Communication, for the conclusion that the risks of HES solutions for critically ill patients, including those with sepsis, do *not* apply to surgical and trauma patients.

What follows is our own review of the literature on the use of HES solutions in surgical and trauma patients, which we believe demonstrates, on balance, that the risks of HES products apply to these two patient populations, resulting in an unfavorable risk-benefit profile.

Randomized trials

A number of randomized controlled trials of HES solutions in surgical and trauma patients have been published in recent years, but these have largely been small, usually with fewer than 100 subjects.[61,62,63,64,65,66] (A notable exception is the 2012 CHEST trial, described in further detail as part of the Wilkes and Navickis 2014 meta-analysis below, in which surgical subjects made up 43% of subjects in each study arm.) We therefore focused our analysis on a series of meta-analyses of these generally small and short-duration trials in surgical or trauma subjects.

Meta-analyses and systematic reviews

- An updated 2013 Cochrane meta-analysis (Mutter et al.) analyzed 42 randomized controlled clinical trials comparing HES solutions with other IV fluids in 11,399 subjects,[67] including 11 trials in 5,911 non-septic surgical (10 trials) or trauma (1 trial)

---

[59] Wise J. Boldt: the great pretender. *BMJ*. 2013;346:f1738.
[60] Mutter TC, Ruth CA, Dart AB. Hydroxyethyl starch (HES) versus other fluid therapies: effects on kidney function. *Cochrane Database Syst Rev*. 2013;7:CD007594.
[61] Kancir AS, Pleckaitiene L, Hansen TB, et al. Lack of nephrotoxicity by 6% hydroxyethyl starch 130/0.4 during hip arthroplasty: A randomized controlled trial. *Anesthesiology*. 2014;121(5):948-958.
[62] Kancir AS, Johansen JK, Ekeloef NP, Pedersen EB. The effect of 6% hydroxyethyl starch 130/0.4 on renal function, arterial blood pressure, and vasoactive hormones during radical prostatectomy: A randomized controlled trial. *Anesth Analg*. 2015;120(3):608-618.
[63] Szturz P, Kula R, Tichy J, et al. Individual goal-directed intraoperative fluid management of initially hypovolemic patients for elective major urological surgery. *Bratisl Lek Listy*. 2014;115(10):653-659.
[64] Zeng K, Li Y, Liang M, et al. The influence of goal-directed fluid therapy on the prognosis of elderly patients with hypertension and gastric cancer surgery. *Drug Des Devel Ther*. 2014 Oct 29;8:2113-2119.
[65] Allison KP, Gosling P, Jones S, et al. Randomized trial of hydroxyethyl starch versus gelatine for trauma resuscitation. *J Trauma*. 1999;47(6):1114-1121.
[66] James MF, Michell WL, Joubert IA, et al. Resuscitation with hydroxyethyl starch improves renal function and lactate clearance in penetrating trauma in a randomized controlled study: the FIRST trial (Fluids in Resuscitation of Severe Trauma). *Br J Anaesth*. 2011;107(5):693-702.
[67] Mutter TC, Ruth CA, Dart AB. Hydroxyethyl starch (HES) versus other fluid therapies: effects on kidney function. *Cochrane Database Syst Rev*. 2013;7:CD007594.

subjects. The primary endpoints in the updated Cochrane meta-analysis were the need for renal replacement therapy, author-defined kidney failure, and acute kidney injury measured using the five-category risk, injury, failure, loss, and end-stage kidney injury (RIFLE) criteria. HES products primarily included 6% solutions of 130/0.4, 200/0.5, 200/0.6, and 450/0.7, while control fluids included crystalloid, albumin, gelatin, and balanced plasma solutions.

Overall, HES-treated subjects had an increased need for renal replacement therapy (19 studies, 9,857 subjects; RR 1.31; 95% CI 1.16 – 1.49), author-defined kidney failure (15 studies, 1,361 subjects; RR 1.59; 95% CI 1.26 – 2.00), and renal failure based on the most severe RIFLE category, RIFLE-Failure (15 studies, 8,402 subjects; RR 1.14; 95% CI 1.01 – 1.30) compared with subjects treated with control fluids. Importantly, the significantly increased risk for the need for renal replacement therapy applied to both high-molecular-weight (9 studies, 1,183 subjects; RR 1.56; 95% CI 1.15 – 2.11) and low-molecular-weight (10 studies, 8,353 subjects; RR 1.26; 95% CI 1.09 – 1.45) HES products versus comparator fluids.

In the 11 trials of surgical and trauma subjects, there was a numerical but non-significant increase in the risk for renal replacement therapy in HES-treated subjects (RR 1.25; 95% CI 0.96 – 1.61). However, in subgroup analyses, there were no significant differences in the risk for either renal replacement therapy or RIFLE-Failure between septic and non-septic (surgical and trauma) subjects.

While there were statistically significant differences in the less severe categories of RIFLE-Risk and RIFLE-Injury between septic and non-septic (surgical and trauma) subjects, these results were deemed by the authors "to be of little clinical significance given the absence of subgroup differences for the more important RIFLE-Failure and [renal replacement therapy] outcomes, where HES solution use was associated with worse outcomes in both patient population subgroups."

- A 2013 meta-analysis (Martin et al.) reviewed all randomized controlled trials of subjects undergoing surgical procedures in which at least one study group received HES 130/0.4 solutions (data on HES 130/0.42 solutions was excluded) and at least one control group received a solution other than HES 130/0.4.[68] The meta-analysis found no increased risk of elevated serum creatinine levels or acute renal failure with this formulation of HES solution compared with comparator fluids.

  However, the study had several important limitations, as pointed out in a letter to the editor responding to the study's publication.[69] First, for eight of the 17 studies, comprising 605 (49%) of the 1,230 subjects included in the meta-analysis, the control subjects received another HES product or gelatin,[70,71] both of which also have been

[68] Martin C, Jacob M, Vicaut E, Guidet B, Van Aken H, Kurz A. Effect of waxy maize-derived hydroxyethyl starch 130/0.4 on renal function in surgical patients. *Anesthesiology*. 2013;118(2):387-394.
[69] Wiedermann CJ. Hydroxyethyl starch 130/0.4: Safe for the kidney in surgical patients? *Anesthesiology*. 2013;119(3):735-736.
[70] *Ibid.*

associated with adverse renal outcomes. Second, the review included a total of only 1,230 subjects, or just 21% of the 5,911 surgical and trauma subjects in the 2013 Cochrane meta-analysis,[72] thereby raising a question as to whether the review had sufficient power to detect the relatively rare outcome of severe renal failure.[73] Finally, the letter to the editor claimed that most studies had a follow-up period of five days or less,[74] a crucial point given the known delay in diagnosis of many cases of HES-induced renal injury (a median of 16 days, according to the authors of the letter to the editor) and in initiation of renal replacement therapy following HES solution exposure (up to 90 days, according to the FDA[75]).

- A 2013 systematic review (Van Der Linden et al.) analyzed 59 publications reporting on the results of randomized controlled trials of 4,529 subjects undergoing elective or emergency surgery, or treatment for trauma or burns, that compared HES 130/0.4 or HES 130/0.42 solutions to various crystalloid or colloid (including other HES) fluids.[76] This review found no significant differences between HES 130 solutions and comparator fluids in increases in serum creatinine, need for renal replacement therapy, rates of increased blood loss or need for blood transfusion, or mortality. The study had similar flaws to the 2013 Martin et al. meta-analysis, including inclusion of several studies with other HES formulations as the comparator fluid and the author-acknowledged "relatively short" duration of follow-up in the trials evaluated.[77]

  A subsequent letter to the editor claimed that the study omitted 18 deaths (two-thirds of which were from the HES study arms) from its analysis of mortality rates, included studies with comparator fluids that may have falsely decreased the relative risk of blood transfusion in the HES study arms, and was underpowered to detect differences in the rate of renal replacement therapy.[78]

- A 2014 meta-analysis (Wilkes and Navickis) of 15 randomized controlled trials in 4,409 adult subjects undergoing surgery found that use of HES solutions significantly increased the need for renal replacement therapy compared to that of non-HES IV solutions (pooled

---

[71] Martin C, Jacob M, Vicaut E, Guidet B, Van Aken H, Kurz A. Effect of waxy maize-derived hydroxyethyl starch 130/0.4 on renal function in surgical patients. *Anesthesiology*. 2013;118(2):387-394.
[72] Mutter TC, Ruth CA, Dart AB. Hydroxyethyl starch (HES) versus other fluid therapies: effects on kidney function. *Cochrane Database Syst Rev*. 2013;7:CD007594.
[73] *Ibid.* The 2013 Cochrane meta-analysis found that just 4.2% of HES-treated non-septic subjects and 9.0% of all subjects (including septic subjects) receiving low-molecular-weight HES required renal replacement therapy.
[74] Wiedermann CJ. Hydroxyethyl starch 130/0.4: safe for the kidney in surgical patients? *Anesthesiology*. 2013;119(3):735-736.
[75] Food and Drug Administration. FDA safety communication:bBoxed warning on increased mortality and severe renal injury, and additional warning on risk of bleeding, for use of hydroxyethyl starch solutions in some settings. November 25, 2013. http://www.fda.gov/BiologicsBloodVaccines/SafetyAvailability/ucm358271.htm. Accessed January 5, 2017.
[76] Van Der Linden P, James M, Mythen M, Weiskopf RB. Safety of modern starches used during surgery. *Anesth Analg*. 2013;116(1):35-48.
[77] *Ibid.*
[78] Takala J, Hartog C, Reinhart K. Safety of modern starches used during surgery: Misleading conclusions. *Anesth Analg*. 2013;117(2):527-528.

RR 1.44; 95% CI 1.04 – 2.01).[79] This meta-analysis included trials comparing HES solutions to crystalloids, albumin, or gelatin. Twelve (80%) of the trials included only elective surgical procedures. Importantly, 10 (67%) of the 15 trials, comprising 90% of analyzed subjects, used only low-molecular-weight HES solutions (130/0.4).

The largest such trial, CHEST, contributed 65% of the total sample size. In this trial comparing HES 130/0.4 solutions to normal saline, surgical patients made up 43% of subjects in each arm.[80] A subgroup analysis of the CHEST trial showed that 4.3% of surgical subjects receiving HES solutions required renal replacement therapy, compared with 3.0% of subjects in the normal saline group (RR 1.38; 95% CI 0.94 – 2.01). By comparison, the need for renal replacement therapy among non-surgical HES subjects was 9.0%, compared to 7.8% in the crystalloid group (RR 1.16; 95% CI 0.94 – 1.43).[81] The relative risks for renal replacement therapy were not significantly different between surgical and non-surgical subjects, leading the meta-analysis' authors to conclude that "there was no evidence from CHEST that surgical patients might be less susceptible to HES-induced renal failure prompting [renal replacement therapy] than their non-surgical counterparts."[82]

- A 2014 meta-analysis (Gillies et al.) included 19 randomized controlled trials of 1,567 subjects undergoing surgery in which perioperative administration of 6% HES solutions was compared with that of any non-starch fluid.[83] Sixteen of the 19 trials used HES with molecular sizes of 200 kDA or less. There was no significant difference in hospital mortality, need for renal replacement therapy, or acute kidney injury between subjects receiving HES solutions and those given comparator fluid. Importantly, just seven of the 19 trials exclusively used crystalloid as the comparator fluid, with the remainder using gelatin (eight trials), albumin (three), or plasma protein fraction (one).

Some authors have distinguished cardiac from non-cardiac surgery with regard to analyzing the effects of HES solutions versus those of other fluids. For this reason, the following three meta-analyses were limited to trials of subjects undergoing either cardiac or non-cardiac surgery:

- A 2012 meta-analysis (Navickis et al.) included 18 trials of HES solutions versus albumin in 970 subjects undergoing cardiopulmonary bypass surgery and found a significant increase in the risk of post-operative blood loss, reoperation for bleeding, and transfusion of red blood cells, fresh-frozen plasma, and platelets in patients receiving HES.[84] Importantly, no significant differences were detected in these outcomes in head-to-head comparisons between HES 130/0.4 solutions and HES 200/0.5 solutions. The FDA

[79] Wilkes MM, Navickis RJ. Postoperative renal replacement therapy after hydroxyethyl starch infusion: a meta-analysis of randomised trials. *Neth J Crit Care.* 2014;18(4): 4-9.
[80] Myburgh JA, Finfer S, Bellomo R, et al. Hydroxyethyl starch or saline for fluid resuscitation in intensive care. *N Engl J Med*. 2012;367(20):1901-1911.
[81] Wilkes MM, Navickis RJ. Postoperative renal replacement therapy after hydroxyethyl starch infusion: a meta-analysis of randomised trials. *Neth J Crit Care.* 2014;18(4): 4-9.
[82] *Ibid.*
[83] Gillies MA, Habicher M, Jhanji S, et al. Incidence of postoperative death and acute kidney injury associated with i.v. 6% hydroxyethyl starch use: systematic review and meta-analysis. *Br J Anaesth*. 2014;112(1):25-34.
[84] Navickis RJ, Haynes GR, Wilkes MM. Effect of hydroxyethyl starch on bleeding after cardiopulmonary bypass: A meta-analysis of randomized trials. *J Thorac Cardiovasc Surg*. 2012;144(1):223-230.

subsequently cited this meta-analysis in support of its conclusion in the 2013 Drug Safety Communication that the agency "considers excess bleeding a **class effect** warranting addition of this new safety information to the Warning and Precautions Section of the [product information]." [emphasis added][85]

- A 2014 meta-analysis (Jacob et al.) reviewed 49 randomized controlled trials of HES solutions versus other fluids in 3,439 subjects undergoing cardiac surgery.[86] The meta-analysis found that higher-molecular-weight HES solutions (those with molecular weight greater than 200 kDA) were associated with greater blood loss and transfusion requirements than comparator fluids. This risk was not seen with lower-molecular-weight HES 130/0.4 solutions, which were associated with less blood loss and fewer transfusion requirements than albumin and gelatin, with no significant differences on these outcomes compared with crystalloids.

  Gelatin was the comparator fluid in a large proportion of trials included in the meta-analysis (such trials contributed 49% and 61% of subjects assessed for blood loss and transfusion requirements, respectively.) The FDA removed all gelatin-containing IV solutions from the U.S. market in 1978 because "the drug caused increased blood viscosity, reduced blood clotting, and prolonged bleeding time."[87] In addition, the authors of the 2012 meta-analysis of HES solutions in cardiac surgery (Navickis et al.) expressed concerns that the results of the new meta-analysis by Jacob et al. may have been biased in favor of HES through the imputation of missing data, the inclusion of two trials where subjects received both study fluids, and the omission of another trial that showed higher blood loss with low-molecular-weight starch than with its comparator fluid.[88]

- A 2016 meta-analysis (Raiman et al.) analyzed 13 randomized controlled trials that compared HES solutions with crystalloid solutions in 741 subjects undergoing non-cardiac surgery.[89] Eight of the 13 trials used low-molecular-weight HES 130/0.4 solutions. The review noted that the trials were generally small (range of n = 20 to n = 202), with "low event rates," thus potentially inhibiting the detection of significant differences in outcomes between the solutions.

  Nevertheless, the authors found a nonsignificant trend towards increased 90-day mortality with HES solutions (3.5% vs. 0.8%; risk ratio 2.97; 95% CI 0.96 – 9.19), although there were uncertainties in this estimate due to five deaths in the HES group in

---

[85] Food and Drug Administration. FDA safety communication: boxed warning on increased mortality and severe renal injury, and additional warning on risk of bleeding, for use of hydroxyethyl starch solutions in some settings. http://www.fda.gov/BiologicsBloodVaccines/SafetyAvailability/ucm358271.htm. Accessed January 5, 2017.
[86] Jacob M, Fellahi JL, Chappell D, Kurz A. The impact of hydroxyethyl starches in cardiac surgery: a meta-analysis. *Crit Care*. 2014;18(6):656.
[87] Food and Drug Administration. List of drug products that have been withdrawn or removed from the market for reasons of safety or effectiveness. 63 FR 54082. October 8, 1998. http://www.fda.gov/ohrms/dockets/98fr/100898b.txt. Accessed January 4, 2017.
[88] Navickis RJ, Haynes GR, Wilkes MM. Tetrastarch in cardiac surgery: error, confounding and bias in a meta-analysis of randomized trials. *Crit Care*. 2015;19:187.
[89] Raiman M, Mitchell CG, Biccard BM, Rodseth RN. Comparison of hydroxyethyl starch colloids with crystalloids for surgical patients: A systematic review and meta-analysis. *Eur J Anaesthesiol*. 2016;33(1):42-48.

one trial having been deemed "unrelated" to the intervention. When these five deaths were removed from the analysis, the risk ratio for mortality decreased to 2.33 (95% CI 0.68 – 7.96).

There was no difference in rates of acute kidney injury or need for renal replacement therapy, nor in major infectious complications. Subjects receiving HES solutions tended to have shorter hospital stays (mean difference -1.52 days; 95% CI -2.87 – -0.18), but the authors noted that trial heterogeneity was high for this comparison (I^2 = 90%), making this result inconclusive.

<u>Observational studies</u>

We detail here just a few of the myriad observational studies that have demonstrated an association between HES solution administration and adverse outcomes in surgical[90,91,92] and trauma[93] patients (others have not shown an association[94,95]):

- A 2014 retrospective study from the Cleveland Clinic assessed the risk of developing post-operative acute kidney injury in 29,360 patients undergoing inpatient, non-cardiac surgery and receiving either HES solutions (670/0.75; Hextend; n = 14,680) or a crystalloid (n = 14,680).[96] The risk of developing an increased level of acute kidney injury was significantly higher among the HES group (adjusted OR 1.21; 97.5% CI 1.06 – 1.38). A significant association was found between the total HES solution volume that was given and the risk of developing a more advanced level of acute kidney injury (adjusted OR 1.44; 97.5% CI 1.26 – 1.64), meaning that each additional 500 mL of HES solution was associated with a 44% increased risk of developing more serious acute kidney injury. There was no association between HES solution use and risk of in-hospital or 90-day mortality.

- A 2014 retrospective study of consecutive admissions to a hospital trauma service assessed the risk of acute kidney injury and 90-day mortality among 1,410 patients who experienced blunt or penetrating trauma and who received 6% HES 450/0.7 solutions and/or blood products.[97] Among blunt trauma patients, HES solution use was associated

---

[90] Ahn HJ, Kim JA, Lee AR, et al. The risk of acute kidney injury from fluid restriction and hydroxyethyl starch in thoracic surgery. *Anesth Analg*. 2016;122(1):186-193.
[91] Lagny MG, Roediger L, Koch JN, et al. Hydroxyethyl starch 130/0.4 and the risk of acute kidney injury after cardiopulmonary bypass: A single-center retrospective study. *J Cardiothorac Vasc Anesth*. 2016;30(4):869-875.
[92] Albrecht FW, Glas M, Rensing H, et al. A change of colloid from hydroxyethyl starch to gelatin does not reduce rate of renal failure or mortality in surgical critical care patients: Results of a retrospective cohort study. *J Crit Care*. 2016;36:160-165.
[93] Eriksson M, Brattström O, Mårtensson J, Larsson E, Oldner A. Acute kidney injury following severe trauma: Risk factors and long-term outcome. *J Trauma Acute Care Surg*. 2015;79(3):407-412.
[94] Kim SK, Choi SS, Sim JH, et al. Effect of hydroxyethyl starch on acute kidney injury after living donor hepatectomy. *Transplant Proc*. 2016;48(1):102-106.
[95] Allen CJ, Ruiz XD, Meizoso JP, et al. Is hydroxyethyl starch safe in penetrating trauma patients? *Mil Med*. 2016;181(5 Suppl):152-155.
[96] Kashy BK, Podolyak A, Makarova N, et al. Effect of hydroxyethyl starch on postoperative kidney function in patients having noncardiac surgery. *Anesthesiology*. 2014;121(4):730-739.
[97] Allen CJ, Valle EJ, Jouria JM, et al. Differences between blunt and penetrating trauma after resuscitation with hydroxyethyl starch. *J Trauma Acute Care Surg*. 2014;77(6):859-864.

with a significantly increased risk of acute kidney injury (OR 2.54; 95% CI 1.24 – 5.19) and mortality (OR 3.77; 95% CI 1.63 – 8.69) compared to patients not receiving HES. These risks were not seen in penetrating trauma patients for either acute kidney injury (OR 0.90; 95% CI 0.23 – 3.60) or mortality (OR 0.72; 95% CI 0.13 – 3.94).

- A 2015 prospective observational study assessed the impact of the administration of HES solutions after neurologic determination of death in kidney donors on recipient renal graft outcomes.[98] Data were obtained on 986 kidneys transplanted from 529 donors. Kidneys from donors who received HES solutions had a higher rate of delayed graft function in recipient patients (41% vs. 31%, $p < 0.001$). After accounting for the propensity of donors to receive HES solutions, HES solution administration was independently associated with an increased risk of delayed graft function in recipients (OR 1.41; 95% CI 1.02 – 1.95). A dose-response relationship was also evident: A larger proportion of kidneys from donors who received > 750 mL of HES solution had a higher risk of developing delayed graft function compared to those receiving 1-750 mL of HES solution and no HES (42% vs. 37.5% vs. 31%, respectively; $p = 0.003$ across all three groups).

b. Argument #2: Newer, low-molecular-weight HES solutions do not carry the same risks as their high-molecular-weight predecessors

Opponents of banning HES solutions claim that new, low-molecular-weight formulations of HES, specifically Voluven, do not cause the same adverse effects as higher-molecular-weight versions. The best evidence to date, however, contradicts this assertion.

First, on a physiological level, as explained above ("Potential mechanisms for HES toxicity"), a 2012 systematic review of 25 studies on the tissue uptake of HES molecules found that both low-molecular-weight and lower-molar-substitution HES actually had significantly higher rates of tissue uptake during the first 24 hours after infusion than did high-molecular-weight and higher-molar-substitution HES, respectively.[99] There is evidence that low-molecular-weight HES may, in fact, be more likely to be deposited in the kidney than high-molecular-weight HES. The primary site of kidney uptake of HES is the proximal tubule. Only HES molecules below 45-60 kDA can pass through the glomerular filtration barrier and be taken up by the proximal tubular cells, leading to osmotic nephrosis and long-term storage.[100]

In terms of clinical outcomes, the most rigorous recent trials that demonstrated increased risks with HES solutions were conducted on low-molecular-weight HES formulations. The CHEST trial, by far the largest HES trial ever conducted, found that low-molecular-weight (130/0.4) HES solution increased the risk of need for renal replacement therapy.[101] And the 6S trial

---

[98] Patel MS, Niemann CU, Sally MB, et al. The impact of hydroxyethyl starch use in deceased organ donors on the development of delayed graft function in kidney transplant recipients: A propensity-adjusted analysis. *Am J Transplant*. 2015;15(8):2152-2158.

[99] Bellmann R, Feistritzer C, Wiedermann CJ. Effect of molecular weight and substitution on tissue uptake of hydroxyethyl starch: A meta-analysis of clinical studies. *Clin Pharmacokinet*. 2012;51(4):225-236.

[100] Wiedermann CJ, Joannidis M. Accumulation of hydroxyethyl starch in human and animal tissues: a systematic review. *Intensive Care Med*. 2014;40(2):160-170.

[101] Myburgh JA, Finfer S, Bellomo R, et al. Hydroxyethyl starch or saline for fluid resuscitation in intensive care. *N Engl J Med*. 2012;367(20):1901-1911.

demonstrated that low-molecular-weight (130/0.4) HES solution significantly increased the risk of all-cause mortality and renal replacement therapy and numerically increased the risk of severe bleeding in subjects with severe sepsis who required fluid resuscitation in the ICU.[102] (See Appendix 3 for more details of both trials.)

The 2013 Cochrane analysis (Mutter et al.), described in detail above, remains the largest meta-analysis of the safety of HES products compared with other IV solutions.[103] A subgroup analysis found a significantly increased risk of renal replacement therapy with both high-molecular-weight (RR 1.56; 95% CI: 1.15 – 2.11) and low-molecular-weight (RR 1.26; 95% CI: 1.09 – 1.45) HES formulations compared with comparator fluids. The authors concluded that "there is insufficient evidence to support that [low-molecular-weight] 6% 130/0.4's favourable pharmacokinetics … compared to older HES products result in improved kidney outcomes compared to higher [molecular weight] and [degree of molar substitution] HES products." And citing the 2012 systematic review of 25 studies that analyzed the tissue uptake of HES, the authors noted that "[n]ewer evidence in fact has shown that the newer products actually exhibit increased tissue uptake as a potential mechanism of toxicity."[104]

The 2014 Wilkes and Navickis meta-analysis of 15 randomized controlled trials in 4,409 adult subjects undergoing surgery, also summarized above, found that HES solutions significantly increased the need for renal replacement therapy (pooled RR 1.44; 95% CI 1.04 – 2.01).[105] Ten (67%) of the 15 trials, comprising 90% of analyzed subjects, used only low-molecular-weight HES (130/0.4) solutions.

c. Argument #3: The adverse effects of HES solutions can be avoided through the use of lower concentrations/doses

In a 2013 review of randomized controlled trials from four 2013 meta-analyses of randomized controlled trials that compared HES solutions with crystalloids in critically ill patients, the authors concluded that, if administered according to a clinical algorithm that restricted the use of HES to acutely hypovolemic patients and below the maximum recommended dose, HES would not confer the same risks as those found in many of the trials published up to that point.[106] They made this suggestion despite acknowledging that "[t]he most important question, whether or not HES may be harmful when it is limited to immediate haemodynamic stabilisation cannot be answered yet. Currently, no study is available that sufficiently addresses this question." In a letter to the editor, Myburgh et al. observed that the algorithm proposed by the authors had never been validated and that the aggregate doses of HES administered to subjects in three large

---

[102] Perner A, Haase N, Guttormsen AB, et al. Hydroxyethyl starch 130/0.42 versus Ringer's acetate in severe sepsis. *N Engl J Med*. 2012;367(2):124-134.
[103] Mutter TC, Ruth CA, Dart AB. Hydroxyethyl starch (HES) versus other fluid therapies: effects on kidney function. *Cochrane Database Syst Rev*. 2013;7:CD007594.
[104] *Ibid.*, citing: Bellmann R, Feistritzer C, Wiedermann CJ. Effect of molecular weight and substitution on tissue uptake of hydroxyethyl starch: a meta-analysis of clinical studies. *Clin Pharmacokinet*. 2012;51(4):225-236.
[105] Wilkes MM, Navickis RJ. Postoperative renal replacement therapy after hydroxyethyl starch infusion: a meta-analysis of randomised trials. *Neth J Crit Care.* 2014;18(4): 4-9.
[106] Meybohm P, Van Aken H, De Gasperi A, et al. Re-evaluating currently available data and suggestions for planning randomised controlled studies regarding the use of hydroxyethyl starch in critically ill patients — a multidisciplinary statement. *Crit Care*. 2013;17(4):R166.

clinical trials in which HES had been associated with renal damage had ranged from 5 to 70 milliliters per kilogram (mL/kg).[107]

In the 2013 Cochrane meta-analysis (Mutter et al.), the risk of renal replacement therapy applied to both high-volume ($\geq$ 2L; RR 1.43; 95% CI: 1.20 – 1.71) and low-volume (< 2L; RR 1.22; 95% CI: 1.02 – 1.46) HES solution administration.[108] Another 2013 meta-analysis (Patel et al.; see Appendix 3 for details) found an increased risk of 90-day mortality and renal replacement therapy with low-molecular-weight HES solution administered to subjects with severe sepsis at a median infusion total volume of 37 mL/kg (range 30-43 mL/kg) or median daily dose of 14 mL/kg/day (range 8-37 mL/kg/day),[109] well below the 50mL/kg/day maximum approved infusion volume for the low-molecular-weight HES product Voluven.[110]

Three retrospective observational studies, published in 2016, also found increased risks of acute kidney injury and need for renal replacement therapy in surgical patients at relatively low median or mean infusion volumes (< 50 mL/kg) of HES solutions.[111,112,113]

d. *Argument #4: HES solutions must remain on the market given the lack of good alternatives for surgical and trauma patients*

Proponents of the continued use of HES products point to their fluid-saving oncotic effects, claiming that patients require significantly less HES than crystalloids to maintain hemodynamic stability. However, there are two other colloidal solutions (albumin and dextran) available in the U.S. for fluid resuscitation.

Furthermore, while HES, like other colloidal solutions, results in less extravasation (movement of fluid out of the intravascular space) and consequently greater intravascular pressures at lower infusion volumes than crystalloids, the crucial questions are whether these differences relative to crystalloids (a) are clinically significant; (b) translate to real benefits to patients; and (c) outweigh the known increased risks of HES.

---

[107] Myburgh J, Finfer S, Bellomo R. Patient-centered outcomes and trials of hydroxyethyl starch. *Crit Care*. 2013;17(5):452.
[108] Mutter TC, Ruth CA, Dart AB. Hydroxyethyl starch (HES) versus other fluid therapies: effects on kidney function. *Cochrane Database Syst Rev*. 2013;7:CD007594.
[109] Patel A, Waheed U, Brett SJ. Randomised trials of 6% tetrastarch (hydroxyethyl starch 130/0.4 or 0.42) for severe sepsis reporting mortality: systematic review and meta-analysis. *Intensive Care Med*. 2013;39(5):811-822.
[110] Hospira Inc. Label: VOLUVEN (hydroxyethyl starch). February 2015. https://dailymed.nlm.nih.gov/dailymed/getFile.cfm?setid=c2c45e02-5d9a-4a54-f291-e5b54671b08b&type=pdf&name=c2c45e02-5d9a-4a54-f291-e5b54671b08b. Accessed January 9, 2017.
[111] Ahn HJ, Kim JA, Lee AR, et al. The risk of acute kidney injury from fluid restriction and hydroxyethyl starch in thoracic surgery. *Anesth Analg*. 2016;122(1):186-193.
[112] Lagny MG, Roediger L, Koch JN, et al. Hydroxyethyl starch 130/0.4 and the risk of acute kidney injury after cardiopulmonary bypass: A single-center retrospective study. *J Cardiothorac Vasc Anesth*. 2016;30(4):869-875.
[113] Albrecht FW, Glas M, Rensing H, et al. A change of colloid from hydroxyethyl starch to gelatin does not reduce rate of renal failure or mortality in surgical critical care patients: Results of a retrospective cohort study. *J Crit Care*. 2016;36:160-165.

<u>Differences in fluid volumes in surgical patients</u>

- A 2011 systematic review (Hartog et al.) of 56 randomized controlled trials, 45 of which were conducted in patients undergoing elective surgery, assessed the safety and effectiveness of low-molecular-weight HES (130/0.4) solutions compared with other fluids.[114] The authors concluded that the studies were too small, too short, and insufficiently similar in design to reach robust conclusions about the relative safety and effectiveness of HES 130/0.4 solutions.

  A subgroup analysis assessed the ratio of the volume of crystalloids to HES solutions infused across six of the trials in subjects undergoing abdominal surgery with goal-directed fluid therapy. The authors noted a common belief within the medical community that 3 to 4 times more crystalloid than colloid volume is needed to achieve similar hemodynamic effects. By contrast, their review found a mean crystalloid-to-HES solution volume ratio of just 1.8 (range: 1.6-2.0).

- The 2012 CHEST trial reported the effects of fluid therapy and treatment between HES solutions and 0.9% sodium chloride (saline).[115] During the first 4 days, subjects on HES solutions received significantly less study fluid (mean [± standard deviation] daily average, 526 ± 425 mL vs. 616 ± 488 mL; $p < 0.001$) and non-study fluid (851 ± 675 mL vs. 1,115 ± 993 mL; $p < 0.001$) compared to those on saline, respectively. However, while there was a significant difference in net fluid balance between the two groups, it was exceedingly small (921 ± 1,069 mL with HES solutions vs. 982 ± 1,161 mL with saline; $p = 0.03$). Furthermore, net fluid balance was slightly less positive for the HES group subjects compared with saline group subjects on study days 0 and 1, whereas net fluid balance was slightly more positive for the HES group subjects on study days 2 and 3.[116] Central venous pressures were significantly higher in the HES group (11.3 ± 4.8 mmHg vs.10.4 ± 4.4 mmHg, $P < 0.001$), but there were no significant differences between the groups in heart rate, mean arterial pressure, or lactate levels during the first 4 days.

  And this slight effect on net fluid balance did not translate to clinically relevant benefits; on the contrary, HES solution use resulted in net harm to subjects. In the first 4 days, the HES group received more blood products than the saline group (78 ± 250 mL vs. 60 ± 190 mL, $p < 0.001$) and, as indicated above, subjects receiving HES solutions exhibited significantly greater need for renal replacement therapy.

- A 2013 prospective cohort study was conducted in 6,478 consecutive patients undergoing cardiopulmonary bypass surgery.[117] The institution changed the type of fluid

---

[114] Hartog CS, Kohl M, Reinhart K. A systematic review of third-generation hydroxyethyl starch (HES 130/0.4) in resuscitation: Safety not adequately addressed. *Anesth Analg*. 2011;112(3):635-645.
[115] Myburgh JA, Finfer S, Bellomo R, et al. Hydroxyethyl starch or saline for fluid resuscitation in intensive care. *N Engl J Med*. 2012;367(20):1901-1911.
[116] *Ibid.* Supplementary Appendix, Figure S1.
[117] Bayer O, Schwarzkopf D, Doenst T, et al. Perioperative fluid therapy with tetrastarch and gelatin in cardiac surgery — a prospective sequential analysis. *Crit Care Med*. 2013;41(11):2532-2242.

administered to patients over time due to safety concerns, from 6% HES solutions, predominantly 130/0.4 (n = 2,137), to gelatin (n = 2,324) and finally crystalloids (n = 2,017). The study found a significantly increased risk of the need for renal replacement therapy with HES (OR 1.46; 95% CI 1.08 – 1.97) and gelatin solutions (OR 1.72; 95% CI 1.33 – 2.24) compared to crystalloids, after propensity score stratification. In addition, the authors reported that colloids were not more effective than crystalloids on surrogate measures of euvolemia. Time to vasopressor cessation, normalization of serum lactate, and normalization of mean arterial pressure did not differ among the three fluids.

Furthermore, subjects on crystalloids needed just 1.4 times more fluids than subjects on HES solutions, and fluid intake was higher for patients receiving crystalloids during only the first 20 hours, with all three groups achieving a negative fluid balance by the third day after surgery.

- A 2014 randomized controlled trial in 202 subjects undergoing elective colorectal surgery compared outcomes between low-molecular-weight HES 130/0.4 solution and a crystalloid (Hartmann's solution).[118] The primary outcome was gastrointestinal morbidity on postoperative day 5, with postoperative complications, hospital length of stay, and the effect on coagulation and inflammation making up the secondary outcomes. No significant difference was seen on the primary outcome (OR 0.96; 95% CI 0.52 – 1.77). There were also no significant differences in postoperative complications or hospital length of stay between the groups. There were significant differences between the groups, at various points after surgery, in a few coagulation parameters (reaction time, clot formation rate, and maximum amplitude),[119] but these were of unknown clinical significance.

  In the first 24 hours after the start of surgery, patients receiving crystalloids received just 18% more total fluid ($p < 0.01$) and had a 17% higher net fluid balance ($p < 0.001$) than subjects receiving HES solutions. Because this modest difference in fluid balance did not translate to any clinical benefits, the study's authors concluded that "HES confers no benefit over crystalloids when used for routine haemodynamic optimization within a goal-directed protocol in the moderate to high-risk colorectal surgical patient. Lower volumes may be required to reach haemodynamic endpoints with HES, but targeted crystalloid administration would appear to be an effective alternative."

- A 2015 meta-analysis evaluated the crystalloid/colloid ratios in studies comparing the two types of fluids in various patient groups.[120] The study found that patients required 50% more crystalloids than starch solutions and that surgical patients required 51-54% more crystalloids than colloids; both differences were statistically significant. When looking only at HES solutions, patients required 41% more crystalloids than low-

[118] Yates DR, Davies SJ, Milner HE, Wilson RJ. Crystalloid or colloid for goal-directed fluid therapy in colorectal surgery. *Br J Anaesth*. 2014;112(2):281-289.
[119] *Ibid.* Supplementary material, Figure S5.
[120] Orbegozo Cortés D, Gamarano Barros T, Njimi H, Vincent JL. Crystalloids versus colloids: exploring differences in fluid requirements by systematic review and meta-regression. *Anesth Analg*. 2015;120(2):389-402.

molecular-weight HES solutions and 80% more crystalloids than high-molecular-weight HES solutions, which, again, were statistically significant differences.

Taken together, these findings indicate that (a) the volumes of IV fluid infused do not differ between HES solutions and crystalloids by as much as generally thought in the medical community; (b) the slight differences in fluid volume and net fluid balance are restricted largely to the first day or two following a surgery or ICU admission; and (c) any such differences do not translate to clinically significant benefits for patients receiving HES.

4. Limited evidence on HES use in children indicates that it likely carries the same risks as in adult patients

The increased risks of HES solutions seen in adults may also apply to children. However, there are relatively few studies evaluating the risks and benefits of HES products in pediatric patients, as recognized by FDA reviewers in a briefing memorandum prepared for the Pediatric Advisory Committee's (PAC's) September 23, 2014, meeting regarding Voluven's use in children. The memorandum stated: "A review of published literature from 08 DEC 2009 (the date of the previous PAC presentation) to 31 DEC 2013 did not identify adequate studies for assessing these risks with pediatric patients due to small study size and short follow-up periods."[121] One of these studies was for the post-marketing commitment for Voluven and consisted of a randomized controlled trial of just 61 pediatric subjects. The study was not powered to detect safety outcomes.[122]

A 2015 meta-analysis (Li et al.) reviewed 13 randomized controlled trials of 1,156 pediatric subjects given 6% low-molecular-weight HES (130/0.4 & 200/0.5) solutions or a comparator fluid of albumin, gelatin, dextran, fresh frozen plasma, or crystalloids.[123] Nine of the 13 trials, comprising 44% of the study population, involved subjects undergoing surgery. Compared with other fluids, HES solutions significantly decreased blood platelet count and increased length of ICU stay. No significant differences were reported in mortality, serum creatinine, activated partial thromboplastin time, or blood loss. The authors of the meta-analysis recommended avoiding use of HES in pediatric patients and concluded that more high-quality clinical trials were needed to confirm its safety.

In addition to the lack of good evidence on the safety of HES solutions in children, the current labels for HES products have ambiguous and somewhat contradictory information on the use of the products in pediatric patients:

---

[121] Food and Drug Administration. Center for Biologics Evaluation and Research. September 23, 2014 Pediatric Advisory Committee Memorandum. Voluven® pediatric safety and utilization review for the Pediatric Advisory Committee (PAC) Meeting. http://www.fda.gov/downloads/AdvisoryCommittees/CommitteesMeetingMaterials/PediatricAdvisoryCommittee/UCM414088.pdf. Accessed January 9, 2017. PDF p. 6.

[122] Van der Linden P, De Villé A, Hofer A, et al. Six percent hydroxyethyl starch 130/0.4 (Voluven®) versus 5% human serum albumin for volume replacement therapy during elective open-heart surgery in pediatric patients. *Anesthesiology*. 2013;119(6):1296-1309.

[123] Li L, Li Y, Xu X, et al. Safety evaluation on low-molecular-weight hydroxyethyl starch for volume expansion therapy in pediatric patients: a meta-analysis of randomized controlled trials. *Crit Care*. 2015;19:79.

- The Voluven label has detailed dosage and administration instructions for pediatric patients of all ages, but elsewhere, the label acknowledges the following: "Studies conducted in children have not been of sufficient size or follow-up duration to assess the risks of renal injury and mortality in this patient population."[124] This statement is buried deep within the label, on page 11 of a 23-page document, with no mention of this fact along with the approved dosage instructions for children on the first page.

- The Hespan label has no dosage information for pediatric patients and the following statement in the Pediatric Use section: "The safety and effectiveness of hetastarch in pediatric patients have not been established. Adequate, well-controlled clinical trials to establish the safety and effectiveness of HESPAN® in pediatric patients have not been conducted."[125] However, there is no explicit contraindication to the use of Hespan in pediatric patients, nor is the approved indication limited to adults.

- In the Hextend label, both the Pediatric Use and Dosage and Administration sections contain a statement that there is insufficient evidence to establish the safety and effectiveness of the product in pediatric patients.[126] However, as with the other HES products, there is no contraindication on the use of Hextend in pediatric patients, nor is the product's approved indication explicitly limited to adult patients.

5. Discussion

a. Safety: Numerous studies confirm risk of HES solutions in surgical and trauma patients, including with low-molecular-weight versions, while other studies are inadequate for evaluation of relevant outcomes

As explained in detail above, increasing and robust evidence of deleterious effects of HES solution use in surgical and trauma patients confirm that these patients are not immune to the previously well-established dangers of HES solutions in critically ill patients, including those with sepsis, for whom the use of HES solutions is no longer indicated. Furthermore, the risks of HES solutions have been seen with newer, low-molecular-weight versions, as in the CHEST trial, and at low volumes of infusion, as seen in the 2013 Mutter et al. meta-analysis. Given this evidence of increased kidney injury and the need for renal replacement therapy in surgical and trauma patients, it is not possible that HES products could safely be used in any patient population.

In their 2014 open letter to the EMA's Executive Director, 76 researchers (Appendix 2) in intensive care medicine and other disciplines asked what we believe to be the central question at

---

[124] Hospira Inc. Label: VOLUVEN (hydroxyethyl starch). February 2015. https://dailymed.nlm.nih.gov/dailymed/getFile.cfm?setid=c2c45e02-5d9a-4a54-f291-e5b54671b08b&type=pdf&name=c2c45e02-5d9a-4a54-f291-e5b54671b08b. Accessed January 9, 2017.

[125] B. Braun Medical Inc. Label: HESPAN (hetastarch). December 2013. http://www.bbraunusa.com/products.html?prid=L6511. Accessed October 10, 2016.

[126] Hospira Inc. Label: HEXTEND (hetastarch). November 2014. https://dailymed.nlm.nih.gov/dailymed/getFile.cfm?setid=fc79700c-6c3b-4998-8307-4dc8c81c5041&type=pdf&name=fc79700c-6c3b-4998-8307-4dc8c81c5041. Accessed October 10, 2016.

issue in this controversy: "[W]hat assumptions or clinical data would indicate that the same pathological mechanisms do not apply in [surgical and trauma, among other] patients with hypovolaemia from blood loss?"[127]

Regarding the necessary "assumptions" for such a conclusion, the 14 experts who dissented from the recommendation of the EMA's PRAC to reverse its ban on HES solutions pointed out that "[t]he mechanism underlying the risks of renal injury and mortality observed with HES is not well understood, and therefore these should be considered to be potential risks in all patient groups."[128] Regarding the required "clinical data" for such a conclusion, the dissenters further noted that a previous EMA advisory group had concluded that "the evidence to demonstrate a lower risk of renal injury and mortality in any settings other than sepsis and the critically ill is weak."[129]

The PRAC dissenters also alluded to the dubious distinction between the critically ill/septic and surgical/trauma patient populations in the first place: "There is an overlap between those patient groups where the benefit-risk balance of HES is judged to be negative (septic and critically ill patients), and the patients who will receive HES under the revised indication allowing use in patients with hypovolaemia due to acute bleeding (e.g. including the trauma and perioperative settings)."[130] We agree that the distinction offered by the EMA and FDA is quite arbitrary and, in many cases, nonexistent.

As discussed extensively above, studies evaluating the safety of HES products in elective surgical patients have been found to be inadequate because of small sample size, short periods of observation, and the use of other colloids as control fluids. Therefore, in addition to the fact that the category of surgical and trauma patients is arbitrary and, in some cases, nonexistent, these characteristics of the studies in such patients preclude definitive conclusions about the safety and benefits of HES solutions.

b. Efficacy: administered fluid volume of uncertain significance for clinical outcomes

This petition presented a number of studies that demonstrated that the fluid-saving effects of HES solutions are very modest and do not correspond to any benefits on either short-term surrogate measures of euvolemia or, more importantly, on clinically relevant outcomes. Indeed, as evidenced by the studies listed above, HES solutions only increase risks compared with crystalloids and certain other colloidal solutions. We believe it is worth recalling the opinion of the PRAC dissenters on this score, namely that "the data evaluating benefit in the perioperative

---

[127] Bellomo R, Bion J, Finfer S, et al. Open letter to the Executive Director of the European Medicines Agency concerning the licensing of hydroxyethyl starch solutions for fluid resuscitation. *Br J Anaesth*. 2014;112(3):595-600.
[128] European Medicines Agency. Assessment report for solutions for infusion containing hydroxyethyl starch. November 11, 2013.
http://www.ema.europa.eu/docs/en_GB/document_library/Referrals_document/Hydroxyethyl_starch-containing_medicines_107/Recommendation_provided_by_Pharmacovigilance_Risk_Assessment_Committee/WC500154254.pdf. Accessed January 9, 2017. PDF pp. 36, 40.
[129] *Ibid*.
[130] *Ibid*.

setting and trauma setting do not adequately support a clinically relevant beneficial effect for HES."[131]

e. Risks of HES a class effect

The risks associated with HES formulations seem to be a class effect and are not exclusive of high-molecular-weight variants. This was confirmed by large randomized controlled trials (6S and CHEST) where low-molecular-weight products (HES 130/0.42 and 130/0.40) were associated with increased mortality and/or renal injury in critically ill patients, including those with sepsis.[132,133] Similar risks were found in meta-analyses of trials of low-molecular-weight HES products,[134] including in surgical patients.[135]

Eight of the 9 panelists from the FDA public workshop held in September 2012 stated in their closing statements that "different HES formulations shared the same safety profile regardless of differences in chemical structure, molecular weight, degree of substitution, branching configuration of the starch, or starch source (maize or potato)." [136] In addition, the same 8 panelists stated that "alternative resuscitation fluids (e.g., crystalloids) were safer and no less effective than HES in certain populations."[137]

Indeed, the FDA acknowledged the validity of this reasoning in its November 25, 2013, Safety Communication, when it noted:

> "In its analysis, FDA extrapolated safety data from the lower molecular weight HES product to higher molecular weight HES products. This extrapolation was justified because of similarities in chemical structure and mechanism of action between the higher and lower molecular weight HES products. In addition, both higher and lower molecular weight formulations are metabolized by α-amylase into similar smaller fragments until the renal threshold of excretion (45-60 kDa) is reached. This fact implies that exposure to smaller molecular weight fragments occurs when higher molecular weight HES products are administered, so that renal toxicity may be anticipated."[138]

---

[131] *Ibid*.
[132] Perner A, Haase N, Guttormsen AB, et al. Hydroxyethyl starch 130/0.42 versus Ringer's acetate in severe sepsis. *N Engl J Med*. 2012;367(2):124-134.
[133] Myburgh JA, Finfer S, Bellomo R, et al. Hydroxyethyl starch or saline for fluid resuscitation in intensive care. *N Engl J Med*. 2012;367(20):1901-1911.
[134] Mutter TC, Ruth CA, Dart AB. Hydroxyethyl starch (HES) versus other fluid therapies: effects on kidney function. *Cochrane Database Syst Rev*. 2013;7:CD007594.
[135] Wilkes MM, Navickis RJ. Postoperative renal replacement therapy after hydroxyethyl starch infusion: a meta-analysis of randomised trials. *Neth J Crit Care*. 2014;18(4): 4-9.
[136] Food and Drug Administration. Center for Biologics Evaluation and Research. September 23, 2014 Pediatric Advisory Committee memorandum. Voluven® pediatric safety and utilization review for the Pediatric Advisory Committee (PAC) Meeting, at PDF p. 20. http://www.fda.gov/downloads/AdvisoryCommittees/CommitteesMeetingMaterials/PediatricAdvisoryCommittee/UCM414088.pdf. Accessed January 9, 2017.
[137] *Ibid.*
[138] Food and Drug Administration. FDA safety communication: boxed warning on increased mortality and severe renal injury, and additional warning on risk of bleeding, for use of hydroxyethyl starch solutions in some settings. November 25, 2013. http://www.fda.gov/BiologicsBloodVaccines/SafetyAvailability/ucm358271.htm. Accessed January 5, 2017.

We agree with the FDA's reasoning that led to its conclusion that the risks of HES solutions constitute a class effect in critically ill patients, including those with sepsis. We believe that the same default assumption that the dangers of HES solutions apply to all formulations should be extended to surgical and trauma patients.

f. Potential publication bias — and outright fraud — characterize some HES literature

Some studies of HES solutions have evidenced significant publication bias, such as selective reporting of positive outcomes, suppression of unfavorable data, and reporting inconclusive results in a way that favors HES solution use.[139,140] A large proportion of these favorable publications have been funded by industry.[141] In addition, several meta-analyses have been criticized for selective inclusion of favorable studies and analyses skewed in favor of HES.[142,143] In the case of Joachim Boldt, a formerly prolific author on HES products, this bias resulted from outright research fraud. After Boldt was found guilty of research misconduct, at least 88 studies he had published were withdrawn from the medical literature.[144]

5. Conclusion

The harms of HES products to patients outweigh their very modest fluid-saving effects, and these solutions should not be used in any patient population, including in surgical, trauma, and pediatric patients. HES products should be immediately removed from the market to prevent further harm to thousands of patients.

Therefore, for the reasons stated above, we hereby petition the FDA, pursuant to the Federal Food, Drug, and Cosmetic Act; 21 U.S.C. § 355(e); and 21 C.F.R. § 10.30, to immediately ban all formulations of HES IV solutions.

C. ENVIRONMENTAL IMPACT STATEMENT

We claim categorical exclusion under 21 C.F.R. § 25.31(a) from the environmental assessment requirement. An assessment is not required because the requested action would not increase the use of the active moiety that is the subject of this petition.

D. ECONOMIC IMPACT

Will be submitted upon request.

---

[139] Wiedermann CJ. Reporting bias in trials of volume resuscitation with hydroxyethyl starch. *Wien Klin Wochenschr*. 2014;126(7-8):189-194.
[140] Hartog CS, Natanson C, Sun J, et al. Concerns over use of hydroxyethyl starch solutions. *BMJ*. 2014;349:g5981.
[141]*Ibid*.
[142] Navickis RJ, Haynes GR, Wilkes MM. Tetrastarch in cardiac surgery: error, confounding and bias in a meta-analysis of randomized trials. *Crit Care*. 2015;19:187.
[143] Takala J, Hartog C, Reinhart K. Safety of modern starches used during surgery: Misleading conclusions. *Anesth Analg*. 2013;117(2):527-528.
[144] Wise J. Boldt: the great pretender. BMJ. 2013;346:f1738.

E. CERTIFICATION

We certify that, to the best of the knowledge and belief of the undersigned, this petition includes all information and views on which this petition relies, and that it includes representative data and information known to the petitioners which are unfavorable to the petition.

Sincerely,

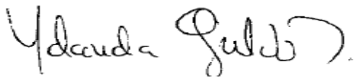

Yolanda Giraldo, M.D., M.P.H.
Contribution to petition while a:
Resident, General Preventive Medicine
Johns Hopkins School of Public Health

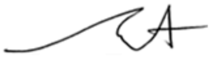

Sammy Almashat, M.D., M.P.H.
Researcher
Public Citizen's Health Research Group

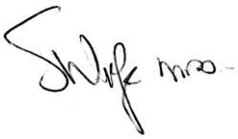

Sidney Wolfe, M.D.
Founder and Senior Adviser
Public Citizen's Health Research Group

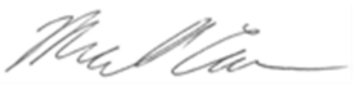

Michael Carome, M.D.
Director
Public Citizen's Health Research Group
1600 20th Street, N.W.
Washington, DC 20009
202-588-1000

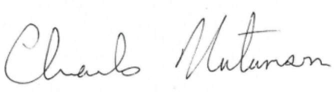

Charles Natanson, M.D.
Critical Care Physician

Ian Roberts, M.B., B.Ch., F.R.C.P., F.P.H.
Coordinating Editor
Cochrane Injuries Group
Co-Director
Clinical Trials Unit, London School of Hygiene & Tropical Medicine

Appendix 1. Divergent statement (dissenting opinion) submitted by 14 members of the EMA's PRAC in response to the EMA's decision to reverse the ban on HES products.[145]

Article 107i of Directive 2001/83/EC

Procedure No: EMEA/H/A-107i/1376

Solutions for infusion containing hydroxyethyl starch

Divergent statement

The following members of PRAC did not agree with the PRAC's Recommendation on the Article 107i referral resulting from pharmacovigilance data for solutions for infusion containing hydroxyethyl starch (HES) based on the following reasons:

1. Harm from use of HES compared with crystalloids in terms of increased mortality and increased renal injury, as well as other serious adverse reactions, has been shown in large well-designed randomised controlled trials in septic and critically ill patients. The available studies in elective surgery and trauma cannot provide reassurance of a lower risk than in septic and critically ill patients, or indeed exclude such a risk. The ad hoc Expert Advice Group held in September 2013 agreed that the evidence to demonstrate a lower risk of renal injury and mortality in any settings other than sepsis and the critically ill is weak.

2. There is very limited evidence on the benefits and risks of hydrox[y]ethyl starch solutions for use in elective surgery and trauma. The magnitude of the volume sparing effect of HES relative to crystalloid solutions has often been cited as 3-4 fold, however there is some evidence that this ratio is lower in surgical settings, around 1.8 fold in some types of surgery (Hartog 2011). It is unclear how the surrogate endpoints from these studies translate to clinically relevant endpoints. Both ad hoc Expert Advice Groups (meetings 19 April 2013 and 13 September) 2013) commented that the data evaluating benefit in the perioperative setting and trauma setting do not adequately support a clinically relevant beneficial effect for HES.

3. There is an overlap between those patient groups where the benefit-risk balance of HES is judged to be negative (septic and critically ill patients), and the patients who will receive HES under the revised indication allowing use in patients with hypovolaemia due to acute bleeding (e.g. including the trauma and perioperative settings). In traumatic injury the patients most likely to receive HES are also those likely to have the most severe injury, and therefore have a greater degree of systemic inflammatory processes and increased risk from exposure to HES. It should also be noted that elective surgery and trauma patients can develop sepsis or complications requiring critical care and these patients cannot be identified in advance. Approximately 20% of the critically ill patients in the CHEST study entered the ICU following elective surgery (Myburgh et al, 2012).

[145] European Medicines Agency. Assessment report for solutions for infusion containing hydroxyethyl starch. November 11, 2013. PDF pp. 36-37, 40-41. http://www.ema.europa.eu/docs/en_GB/document_library/Referrals_document/Hydroxyethyl_starch-containing_medicines_107/Recommendation_provided_by_Pharmacovigilance_Risk_Assessment_Committee/WC500154254.pdf. Accessed October 10, 2016.

4. The mechanism underlying the risks of renal injury and mortality observed with HES is not well understood, and therefore these should be considered to be potential risks in all patient groups. Systemic inflammatory processes may contribute to the observed increased risk in sepsis and burn injury. There is a continuum in the extent of systemic inflammation between healthy individuals and patients with sepsis or burn injury. Trauma and surgery patients are located on an intermediate position on this continuum. There is also evidence that tissue deposition of hydroxyethyl starch occurs in healthy patients without inflammatory processes (Sirtl et al, 1999).

5. Alternative treatments are available in the form of crystalloids, and high quality care is possible without the use of HES: a survey of 391 ICUs worldwide conducted in 2010 (Finfer et al, 2010) showed no use of HES in the United States or Australia.

6. Without evidence to provide reassurance that patients will not be exposed to increased risk of mortality and renal injury by use of HES, and given the lack of data supporting a clinically relevant benefit, suspension of marketing authorisations for HES products in all patient populations remains appropriate to protect public health. This would avoid the situation where patients are unnecessarily exposed to risk from treatment with HES with no convincing evidence that they are receiving any additional benefit.

7. The ability of the proposed risk minimisation measures to sufficiently minimise the risks of HES is a concern. Data are lacking to identify an appropriate maximum dose, and expert advice is that there is no absolute ‘safe’ lower dose below which there is no risk associated with HES administration. The recommendation to monitor renal function in patients for at least 90 days may not be an effective measure to minimize the risk of renal injury in all patients as detection of worsening of renal function by monitoring may not be practical in patients who are discharged shortly after receiving HES. Furthermore, in emergency settings, it may be particularly difficult to evaluate patients for contraindications.

Due to the above mentioned arguments the below mentioned PRAC delegate considers the benefit-risk balance of hydroxyethyl starch (HES) to be negative in all patient populations, justifying the suspension of the marketing authorisations of all HES-containing medicinal products.

PRAC members expressing a divergent position:

Kamila Czajkowska (PL)

Marie Louise De Bruin

Jacqueline Genoux-Hames (LU)

Martin Huber (DE)

Brigitte Keller-Stanislawski

Maria Popova-Kiradjieva (BG)

Carmela Macchiarulo (IT)

Almath Spooner (IE)

Doris Stenver (DK)

Amy Tanti (MT)

Kirsti Villikka (FI)

Julie Williams (UK)

Stephen Evans

Ingebjørg Buajordet (NO) [note that Dr. Buajordet submitted a separate, but identical, divergent statement]

Appendix 2. Signatories to the 2014 open letter to the EMA urging the agency to rescind the reversal of its original decision to ban HES solutions in Europe.[146]

Author	Country	Affiliation
D. Angus	USA	Distinguished Professor and Mitchell P. Fink Endowed Chair Department of Critical Care Medicine, University of Pittsburgh and UPMC Health System, Pittsburgh, PA, USA
M. Antonelli	Italy	President of the Italian Society of Anesthesiology and Intensive Care Medicine (SIAARTI); Director of the General ICU, Institute of Anesthesiology and Intensive Care and Director of the School of Specialty in Anesthesiology and Intensive Care Medicine, University Hospital; Rome, Italy
A. Artigas	Spain	Director Critical Care Center Sabadell Hospital, Barcelona, Spain; Past President Catalan Intensive Care Society
M. Bauer	Germany	Professor of Anesthesiology and Critical Care Medicine; Chief-Executive Director Center for Sepsis Control & Care; Jena University Hospital, Jena, Germany
R. Bellomo	Australia	MBBS, MD (Hons), FRACP, FCICM, PG Dip Echo; Professorial Fellow, Faculty of Medicine, University of Melbourne, Melbourne, Australia; Honorary Professor, Faculty of Medicine, Monash University, Melbourne, Australia; Honorary Professorial Fellow, Faculty of Medicine, The University of Sydney, Sydney, Australia; Concurrent Professor, Faculty of Medicine, University of Nanjing, Nanjing, China; Honorary Principal Research Fellow, Howard Florey Institute, University of Melbourne; Co-director, Australian and New Zealand Intensive Care Research Center; Editor, *Critical Care and Resuscitation*; Director of Intensive Care Research, Staff Specialist in Intensive Care, Department of Intensive Care, Austin Hospital, Heidelberg, Victoria, Australia
G. Bernard	USA	Professor of Medicine; Chair, NIH NHLBI ARDS Clinical Trials Network; Melinda Owen Bass Professor of Medicine; Program Director, Vanderbilt Institute for Clinical and Translational Research; Associate Vice Chancellor for Research; Senior Associate Dean for Clinical Science Vanderbilt University Medical Center; Nashville, TN, USA
J. Bion	UK	FRCP, FRCA, FFICM, MD, Professor of Intensive Care Medicine, University of Birmingham; Past President of the European Society of Intensive Care Medicine (ESICM); Past Foundation Chair of the European Board of Intensive Care Medicine; Past Foundation Dean of the UK Faculty of Intensive

[146] Bellomo R, Bion J, Finfer S, Myburgh J, Perner A, Reinhart K; all co-signatories. Open letter to the Executive Director of the European Medicines Agency concerning the licensing of hydroxyethyl starch solutions for fluid resuscitation. *Br J Anaesth*. 2014;112(3):595-600.

Author	Country	Affiliation
		Care Medicine
L. Brochard	France	Previous (until October 2013) Head of the Medico Surgical ICU in Geneva University Hospital, Geneva, Switzerland; presently, Interdepartmental Division Director for Critical Care in the Faculty of Medicine, University of Toronto, Ontario, Canada; Full Professor, Clinician scientist in the Division of Critical Care Saint Michael's Hospital, Toronto, ON, Canada. Head of the European Clinical Research Network REVA
C. Brun Buisson	France	MD, Director, Medical ICU, CHU Henri Mondor; Professor of Critical Care Medicine, Université Paris-Est Créteil; Paris, France
F. M. Brunkhorst	Germany	Secretary General of the German Sepsis Society, Director of the Center of Clinical Studies, Department of Anesthesiology and Intensive Care Medicine, Jena University Hospital, Jena, Germany
V. Bumbasirevic	Serbia	President, Serbian Society of Intensive Care Medicine; Head, Department of Intensive Care Medicine; Emergency Center, Clinic of Anesthesiology, Clinical Center of Serbia; Belgrade, Serbia
H. Burchardi	Germany	FRCA, Professor of Anesthesiology and Critical Care Medicine; Bovenden, Germany; Past President of the European Society of Intensive Care Medicine (ESICM)
P. Caironi	Italy	Assistant Professor; Department of Pathophysiology and Transplantation; Policlinico Hospital of Milan; Italy
J. Carlet	France	Past President of the European Society of Intensive Care Medicine (ESICM)
J. Chalmers	Australia	Emeritus Professor of Medicine, University of Sydney and George Institute for Global Health
J. Chastre	France	Professor of Medicine at Paris, University School of Medicine; Director of the Medical Intensive Care Unit and of the Intermediate Care Unit at the Institute of Cardiology, Groupe Hospitalier Pitié–Salpêtrière, Paris, France
G. Citerio	Italy	MD; Professor; Department of Perioperative Medicine and Intensive Care, Hospital San Gerardo, Monza, Italy
D. Cook	Canada	Canada Research Chair in Critical Care, McMaster University, Ontario, Canada
J. Cooper	Australia	BMBS, MD, FRACP, FCICM; Professor of Intensive Care Medicine; Director, ANZICS Research Center, Monash University; Director, Center of Research Excellence for Patient Blood Management in Critical Illness and Trauma, Monash University; Director, Critical Care, School of Public Health and Preventive Medicine, Monash University; Head, The Alfred ICU

Author	Country	Affiliation
		Research; Melbourne, Australia
P. Dellinger	USA	Professor and Chair, Department of Medicine, Cooper Medical School of Rowan University and Chief, Medicine Service at Cooper University Hospital, Camden, NJ. Past president of the Society of Critical Care Medicine
T. Evans	UK	Professor of Intensive Care Medicine, Imperial College, London, UK
S. Finfer	Australia	Professor of Critical Care Medicine, The George Institute of Global Health, The University of Sydney, Sydney, Australia
H. Flaatten	Norway	Department of Anesthesia, Haukeland University Hospital (HUS); University of Bergen, Norway
R. Freebairn	New Zealand	Consultant, Intensive Care Services & Clinical Director, Acute Services Hawke's Bay Hospital; Adjunct Associate Professor, Department of Anesthesia & Intensive Care, Chinese University of Hong Kong; President, College of Intensive Care Medicine, Melbourne, Australia
C. French	Australia	FCICM, FANZCA; Clinical Associate Professor, The University of Melbourne; Adjunct Associate Professor, The University of Notre Dame; Director of Intensive Care Western Health; Vice Chair Australian and New Zealand Intensive Care Society Clinical Trials Group (ANZICS); Melbourne, Australia
D. Gattas	Australia	Clinical Associate Professor, Sydney Medical School, University of Sydney; Executive Committee, Australian and New Zealand Intensive Care Society Clinical Trials Group (ANZICS); Honorary Fellow, Critical Care & Trauma Division, The George Institute for Global Health; Senior Staff Intensivist, Royal Prince Alfred Hospital, Sydney, Australia
L. Gattinoni	Italy	MD, FRCP; Professor in Anesthesia and Intensive Care, University of Milan; Chairman of the Department of Pathophysiology and Transplantation, University of Milan; Chief of the Department of Emergency and Intensive Care, Policlinico Hospital of Milan, Italy; Past President of the World Federation Societies of Intensive and Critical Care Medicine (WFSICCM)
H. Gerlach	Germany	Director, Department of Anesthesiology, Operative Intensive Care and Pain Management, Vivantes-Klinikum Neukoelln, Berlin, Germany; Past Secretary ESICM; Past Treasurer ESICM; Past Chair of the Section Systemic Inflammation and Sepsis; President of the German Sepsis Society
E. J. Giamarellos-Bourboulis	Greece	4th Department of Internal Medicine, University of Athens, Medical School, Athens, Greece; Co-ordinator Hellenic Sepsis Study Group (www.sepsis.gr)
C. Hartog	Germany	MD, Senior lecturer; Department of Anesthesiology and Intensive Care Medicine, Jena University Hospital, Jena,

Author	Country	Affiliation
		Germany
C. Hinds	UK	Professor of Intensive Care Medicine; Barts and The London School of Medicine & Dentistry, Queen Mary University of London, UK
U. Kaisers	Germany	Professor of Anesthesia and Intensive Care Medicine; Director of the Department for Anesthesiology and Intensive Care, University of Leipzig, Medical Faculty, Leipzig, Germany
M. Levy	USA	Professor of Medicine, Brown University, School of Medicine, Providence RI, USA; Past President of the Society of Critical Care Medicine (SCCM)
J. Lipman	Australia	MBBCH, DA, FFA (Crit Care), FCICM, MD Professor and Head; Anesthesiology and Critical Care, The University of Queensland, Queensland, Australia
S. MacMahon	Australia/UK	Principal Director, The George Institute for Global Health; Executive Director, The George Institute for Global Health UK at the Oxford University, UK; Professor of Medicine, University of Sydney, Australia; Professor of Medicine and James Martin Professorial Fellow, Oxford University, UK
D. McAuley	UK	Professor of Intensive Care Medicine Center for Infection and Immunity, Queen's University of Belfast and Royal Victoria Hospital, Belfast; Co-Director of Research, UK Intensive Care Society; Chair, Irish Critical Care Trials Group; Director Northern Ireland Clinical Trials Unit; Belfast, UK
S. McGuinness	New Zealand	FRCA, FANZCA, FFICM; Specialist, Cardiothoracic & Vascular ICU, Auckland City Hospital; Auckland, New Zealand
L. McIntyre	Canada	MD, MSc, FRCPC, Assistant Professor, Department of Medicine (Critical Care), Ottawa Hospital; Scientist, Ottawa Hospital Research Institute; Center for Transfusion and Critical Care Research; Adjunct Scientist, Canadian Blood Services; Ottawa, Ontario, Canada
M. Maggorini	Switzerland	Professor; Medical Intensive Care Unit, University Hospital Zurich; Zurich, Switzerland; Chair of the Division of Professional Development ESICM 2010–3
J. Mancebo	Spain	MD, Director Intensive Care Medicine at University Hospital Sant Pau, Barcelona, Spain
J. Marshall	Canada	MD, FRCSC, Professor of Surgery, University of Toronto, Senior lnvestigator, Li Ka Shing Knowledge Institute, St Michael's Hospital; Chairman International Forum for Acute Care Trialists and past-chair, Canadian Critical Care Trials Group; Toronto, Ontario, Canada
R. Moreno	Portugal	MD, PhD, Director, UCINC Hospital de São José Centro Hospitalar de Lisboa Central Lisbon, Portugal; Past President

Author	Country	Affiliation
		Portuguese Society of Intensive Care Medicine; Past President of the European Society of Intensive Care Medicine (ESICM); Past President European Board of Intensive Care Medicine
J. Morgan	Australia	Fellow of the College of Intensive Care Medicine of Australia and New Zealand; Intensive Care Specialist, Mater Health Services; Senior Lecturer, University of Queensland; Brisbane, Australia
J. Myburgh	Australia	MBBCh, PhD, FCICM; Professor of Critical Care Medicine, University of New South Wales; Director, George Institute for Global Health; Honorary Professor, University of Sydney; Honorary Professor, Monash University, Melbourne; Senior Physician, Department of Intensive Care Medicine, St George Hospital, Sydney, Australia; Past President, College of Intensive Care Medicine; Past Chairman, Australian and New Zealand Intensive Care Society Clinical Trials Group (ANZICS); Lead investigator, Crystalloid vs. Hydroxyethyl Starch Trial (CHEST)
C. Natanson	USA	Senior investigator and chief of the Anesthesia Section, Clinical Center's Critical Care Medicine Department at the National Institutes of Health (NIH); Professor of Anesthesia at University of Maryland and at Johns Hopkins University, Baltimore, MD; Clinical Professor of Medicine at George Washington University; Washington, DC, USA
R. Norton	UK/Australia	MA, MPH, PhD; Professor of Global Health & James Martin Professorial Fellow, University of Oxford; Professor of Public Health, The University of Sydney; Sydney, Australia; Co-Principal Director of The George Institute for Global Health; Sydney, Australia
D. Payen	France	Professor of Medicine; Director, Department of Anesthesiology and Intensive Care, Lariboisiere University Hospital; University Paris 7 Denis Diderot, Paris, France
A. Perner	Denmark	Professor in Intensive Care Medicine, University of Copenhagen; Chair Scandinavian Critical Care Trials Group; Senior staff specialist, Rigshospitalet, Copenhagen, Denmark; Principal investigator, The TRISS trial
V. Perkovic	Australia	MBBS, PhD, FRACP FASN Executive Director, The George Institute Australia; Professor of Medicine, University of Sydney, Sydney, Australia
A. Pesenti	Italy	Professor of Anesthesia and Intensive Care, University of Milano Bicocca, Chief Anesthesia and Critical Care; Director, Department of Emergency Medicine, Ospedale San Gerardo, Monza, Italy; Coordinator of the Emergency Medical Services of Milan Metropolitan Area, Regional Emergency Systems Agency (AREU), Regione Lombardia, Italy

Author	Country	Affiliation
V. Pettila	Finland	MD, Professor, Helsinki University Central Hospital, Helsinki, Finland
C. Putensen	Germany	Professor of Intensive Care Medicine, University Hospital of Bonn; Past Chairman of the ESICM Acute Respiratory Failure Section, Bonn, Germany
M. Quintel	Germany	Director, Center for Anesthesiology, Emergency and Intensive Care Medicine, University of Göttingen, Göttingen, Germany; Past President Interdisciplinary Society of Intensive Care Medicine (DIVI)
M. Ranieri	Italy	Professor of Anesthesia and Critical Care Medicine; Department of Anesthesia and Intensive Care Medicine, University of Turin, Italy; Past President of the European Society of Intensive Care Medicine (ESICM)
K. Reinhart	Germany	Professor of Anesthesia and Critical Care Medicine, Jena University Hospital, Jena, Germany; Chairman Global Sepsis Alliance; Member of the German National Academy of Science Leopoldina
A. Rhodes	UK	FRCP, FRCA, FFICM, MD(Res); Hon. Reader in Anesthesia and Intensive Care Medicine St George's Healthcare NHS; Trust and St George's University of London, London, UK; Past President of the European Society of Intensive Care Medicine (ESICM)
C. Richard	France	Professor of Critical Care Medicine; Head of Medical Intensive Care Unit; Bicetre Hospital; Risk and Quality Coordinator; Assistance Publique—Hôpitaux de Paris, Paris, France; Past President of the Société de Réanimation de Langue Française (SRLF)
N.C. Riedemann	Germany	Vice Director Intensive Care Medicine, Jena University Hospital, Jena, Germany
I. Roberts	UK	Editor, Cochrane Injuries Group; Director, Clinical Trials Unit, London School of Hygiene & Tropical Medicine, Keppel Street, London, UK
G. Rubenfeld	Canada	MD, MSc, FRCPC; Chief, Trauma, Emergency and Critical Care Program, Sunnybrook Health Sciences Center; Professor of Medicine, Interdepartmental Division of Critical Care Medicine, University of Toronto; Senior Scientist, Sunnybrook Research Institute; Toronto, Ontario, Canada
F. Schortgen	France	MD, PhD; Department of Medical Intensive Care, Henri Mondor University Hospital, University Paris-Est, Créteil, France
G. Sigurdsson	Iceland	Chairman of University Hospital Research Council; Professor & Faculty Chairman; Department of Anaesthesia & Intensive Care Medicine, Landspitali University Hospital, Reykjavik, Iceland; Director, Scandinavian Postgraduate Program in Intensive Care

Author	Country	Affiliation
		Medicine; Editor, *Acta Anaesthesiologica Scandinavica*
C. Sprung	Israel	Director, General Intensive Care Unit; Department of Anesthesiology and Critical Care Medicine, Hadassah Hebrew University Medical Center, Jerusalem, Israel; Past Treasurer of European Society of Intensive Care Medicine (ESICM)
N. Stochetti	Italy	Professor of Anesthesia and Intensive Care, Milan University, Department of Physiopathology and Transplants, Milan, Italy
P. Suter	Switzerland	Honorary Professor, University of Geneva; Genva, Switzerland; Past President of Swiss Academie of Science and European Society of Intensive Care Medicine (ESICM)
J. Takala	Switzerland	MD, PhD, Professor of Intensive Care Medicine; Director and Chief Physician, Department of Intensive Care Medicine; University Hospital Bern (Inselspital); Bern, Switzerland; Past President of the European Society of Intensive Care Medicine (ESICM)
T. Thompson	USA	MD, Director of Translational Research; Pulmonary Critical Care Unit; Massachusetts General Hospital; Professor of Medicine; Harvard Medical School; Boston, MA, USA
A. Turner	Australia	President Australian and New Zealand Intensive Care Society
T. Walsh	UK	Professor of Critical Care, Edinburgh University; Chair UK National Institute of Healthcare Research Critical Care Network, Edinburgh, UK
S. Webb	Australia	Senior Staff Specialist, Intensive Care Unit, Royal Perth Hospital; Clinical Professor, School of Medicine and Pharmacology and School of Population Health, University of Western Australia; Perth, WA, Australia; Immediate Past Chair, Australian and New Zealand Intensive Care Society Clinical Trials Group (ANZICS)
N. Webster	UK	Professor of Anesthesia and Intensive Care, University of Aberdeen; Aberdeen, UK
T. Welte	Germany	Director, Department of Respiratory Medicine, Hannover Medical School, Hannover, Germany; President of the German Society of Pneumology (DGP); Past President of the German Sepsis Society; Member of the Executive Board of the German Interdisciplinary Society of Intensive Care Medicine (DIVI); Spokesman of the Fachkolleg I (Inflammation) of the German Research Council (DFG)
M. White	Australia	MB BCh, BAO, FFARCSI; Associate Professor, Discipline of Acute Care Medicine, University of Adelaide; Adelaide, Australia; Immediate Past President, Australian and New Zealand Intensive Care Society Clinical Trials Group (ANZICS)
C. Wiedermann	Italy	Associate Professor of Internal Medicine, Medical University of

Author	Country	Affiliation
		Innbruck; Past President of the Austrian Society of General and Medical Intensive Care Medicine; Co-Editor of 'Pharmainformation—Unabhängige Information für Ärzte/innen' by The Austrian Chamber of Physicians (Member of the International Society of Drug Bulletins, ISDB)
D. Young	UK	Senior Clinical Lecturer in Intensive Care, University of Oxford; Oxford, UK; Chair, CHEST study DMEC
R. Zarychanski	Canada	MD, MSc, University of Manitoba, Department of Internal Medicine, Sections of Critical Care and of Hematology and Medical Oncology, University of Manitoba, Winnipeg, Canada

Appendix 3. Summary of the strongest evidence for the adverse effects of HES in critically ill patients, including those with sepsis.

Randomized Controlled Trials

- A 2008 German two-by-two factorial trial (Brunkhorst et al.) randomly assigned 537 severely septic subjects to either intensive or conventional insulin therapy and either HES 200/0.5 solution or modified Ringer's lactate.[147] The co-primary endpoints were all-cause mortality at day 28 and the mean score on the Sequential Organ Failure Assessment scale. The planned interim analysis found that HES solution was associated with a significantly higher incidence of acute renal failure (34.9% vs. 22.8%; p = 0.002) and renal replacement therapy (31.0% vs. 18.8%; p = 0.001) and a trend toward increased 90-day all-cause mortality (41.0% vs. 33.9%; p = 0.09) compared with Ringer's lactate. Subjects receiving HES solution also had a lower median platelet count and required more units of red blood cells than those receiving Ringer's lactate. The study was therefore terminated early.

- The 2012 CRYSTMAS study randomly assigned 196 subjects with severe sepsis to fluid resuscitation treatment with either low-molecular-weight HES (130/0.4) or normal saline.[148] The primary endpoint for which the trial was powered was the difference in the volume of fluid necessary for subjects to become hemodynamically stable. Significantly less HES than normal saline was required to reach hemodynamic stability (1379 ± 886 mL vs. 1,709 ± 1,164 mL, respectively; p = 0.0185). However, there was no significant difference in the total amount of fluid administered over four days, or in the mean fluid balance, between the groups. There also were no significant differences between the groups in any vital signs, hemodynamic parameters, or ICU or hospital lengths of stay.

  The group receiving HES had a slightly numerically higher mortality rate than the group receiving normal saline at both 28 days (31% vs. 25%, respectively) and 90 days (40% vs. 34%, respectively). Neither mortality difference was statistically significant, but this small study was not powered to detect significant differences between the groups on mortality or any other major clinical outcome.

- The 2012 Crystalloid versus Hydroxyethyl Starch Trial (CHEST) study randomly assigned 7,000 patients admitted to an intensive care unit and requiring fluid resuscitation to either 6% HES 130/0.4 solution or 0.9% sodium chloride, in indistinguishable bags, for all fluid resuscitation needs. A total of 43% of the subjects were surgical cases, and 71% were non-septic.

  The primary endpoint was all-cause mortality within 90 days. Secondary endpoints were acute kidney injury, the need for renal replacement therapy, new failure of other organ

[147] Brunkhorst FM, Engel C, Bloos F, et al. Intensive insulin therapy and pentastarch resuscitation in severe sepsis. *N Engl J Med*. 2008;358(2):125-139.

[148] Guidet B, Martinet O, Boulain T, et al. Assessment of hemodynamic efficacy and safety of 6% hydroxyethylstarch 130/0.4 vs. 0.9% NaCl fluid replacement in patients with severe sepsis: The CRYSTMAS study. *Crit Care*. 2012;16(3):R94.

systems, duration of mechanical ventilation and renal replacement therapy, and cause-specific mortality. Acute kidney injury was measured using the five-category RIFLE criteria.

There was no statistically significant difference between the two groups in all-cause mortality at 90 days (18% HES solution vs. 17% saline; RR 1.06; 95% CI 0.96 – 1.18; p = 0.26). HES solution put significantly fewer subjects at risk for kidney dysfunction (RIFLE-R: 54.0% vs. 57.3%; RR 0.94; 95% CI 0.90 – 0.98; p = 0.007) and led to significantly fewer rates of kidney injury (RIFLE-I: 34.6% vs. 38.0%; RR 0.91; 95% CI 0.85 – 0.97; p = 0.005) than saline. However, HES solution led to numerically higher rates of the more severe outcome of kidney failure (RIFLE-Failure: 10.4% vs. 9.2%; RR 1.12; 95% CI 0.97 – 1.30; p = 0.12) and, most importantly, more subjects receiving HES solution needed renal-replacement therapy than did subjects receiving saline (7.0% vs. 5.8%; RR 1.21; 95% CI 1.00 – 1.45; p = 0.04).

The authors explained these seemingly contradictory results by pointing out that HES solution had different effects on the two components of the RIFLE score, serum creatinine levels and urine output. An increased urine output with HES solution, likely through intravascular volume expansion or a direct diuretic effect, accounted entirely for the significantly better RIFLE-R and RIFLE-I scores in the HES group.[149] However, HES solution consistently increased serum creatinine levels across all RIFLE levels, “suggesting a progressive reduction in creatinine clearance and more severe acute kidney injury.”[150]

There were conflicting results on new organ failure. Significantly fewer subjects receiving HES solution experienced cardiovascular failure (36.5% vs. 39.9%; RR 0.91; 95% CI 0.84 – 0.99; p = 0.03), but HES solution led to significantly higher rates of hepatic failure (1.9% vs. 1.2%; RR 1.56; 95% CI 1.03 – 2.36; p = 0.03) and numerically higher rates of coagulation failure (4.8% vs. 4.0%; RR 1.20; 95% CI 0.95 – 1.53; p = 0.13). The authors noted that the reduction in cardiovascular failure was defined as a reduction in the use of vasopressors, which was likely due to the greater intravascular volume expansion seen in HES subjects compared with those receiving saline. However, the reduction in cardiovascular failure “was not associated with differences in other resuscitation end points, such as mean arterial pressure or serum lactate levels.”[151]

HES solution was associated with more treatment-related adverse events overall (5.3% vs. 2.8%, respectively; p < 0.001), mainly pruritis and skin rash, than normal saline.

The authors concluded that their “study does not provide evidence that resuscitation with 6% HES (130/0.4), as compared with saline, in the ICU [intensive care unit] provides any clinical benefit to the patient. Indeed, the use of HES resulted in an increased rate of renal-replacement therapy.”[152]

[149] *Ibid*. Supplementary Appendix.
[150] *Ibid*.
[151] *Ibid.*
[152] *Ibid.*

- The 2012 Scandinavian Starch for Severe Sepsis/Septic Shock (6S) trial compared the effects of fluid resuscitation with either 6% HES 130/0.42 solution or Ringer's acetate among 798 subjects with severe sepsis who required fluid resuscitation in the ICU.[153] The composite primary outcome was all-cause mortality or dependence on dialysis at 90 days. Significantly more HES subjects than those receiving Ringer's acetate experienced the primary outcome (51% vs. 43%, respectively; RR 1.17; 95% CI 1.01 – 1.36; p = 0.03). This increased risk was driven almost entirely by increased all-cause mortality with HES solution (51% vs. 43%; RR 1.17; 95% CI 1.01 – 1.36; p = 0.03), as only one subject in each group was dependent on dialysis by day 90. The need for renal replacement therapy (22% vs. 16%; RR 1.35; 95% CI 1.01 – 1.80; p = 0.04) and severe bleeding (10% vs. 6%; RR 1.52; 95% CI 0.94 – 2.48; p = 0.09) also were higher for subjects in the HES group.

  The authors concluded that "patients with severe sepsis who received fluid resuscitation with HES 130/0.42, as compared with those who received Ringer's acetate, had a higher risk of death at 90 days, were more likely to receive renal-replacement therapy, and had fewer days alive without renal-replacement therapy and fewer days alive out of the hospital."[154] In a subsequent post-hoc analysis, some of the authors concluded that acute kidney impairment may have been partly responsible for the increased rate of death in HES subjects.[155]

- The 2013 Colloids Versus Crystalloids for the Resuscitation of the Critically Ill (CRISTAL) trial randomized 2,857 critically ill subjects (sepsis, trauma, or hypovolemic shock without sepsis or trauma) requiring fluid resuscitation for acute hypovolemia to one of several colloids (n = 1,414; gelatins, dextran, albumin, or HES solutions) or crystalloids (n = 1,443; isotonic or hypertonic saline or Ringer's lactate).[156] The primary outcome was mortality within 28 days, while secondary outcomes included mortality at day 90 and days without the need for renal replacement therapy. There was no significant difference in mortality at 28 days between subjects receiving colloids and those receiving crystalloids (25.4% vs. 27.0%, respectively; RR 0.96; 95% CI 0.88 – 1.04; p = 0.26); colloids did result in a significant reduction in mortality at 90 days (30.7% vs. 34.2%; RR 0.92; 95% CI 0.86 – 0.99; p = 0.03). However, among subjects who received only one type of fluid and in subjects with sepsis, there was no significant difference between HES solutions and crystalloids in either 28-day or 90-day mortality, with the sole exception of a significant reduction in 90-day mortality in subjects receiving only HES solutions compared with subjects receiving only isotonic saline (HR 0.79; 95% CI 0.66 – 0.95). The authors concluded that the finding of reduced mortality "should be considered exploratory and requires further study before reaching conclusions about efficacy."[157]

[153] Perner A, Haase N, Guttormsen AB, et al. Hydroxyethyl starch 130/0.42 versus Ringer's acetate in severe sepsis. *N Engl J Med*. 2012;367(2):124-134.
[154] *Ibid.*
[155] Müller RB, Haase N, Lange T, et al. Acute kidney injury with hydroxyethyl starch 130/0.42 in severe sepsis. *Acta Anaesthesiol Scand*. 2015;59(3):329-336.
[156] Annane D, Siami S, Jaber S, et al. Effects of fluid resuscitation with colloids vs crystalloids on mortality in critically ill patients presenting with hypovolemic shock: the CRISTAL randomized trial. *JAMA*. 2013;310(17): 1809-1817.
[157] *Ibid.*

Meta-analyses

In 2013, to our knowledge, five meta-analyses (in addition to Mutter et al.,[158] described in detail in the main body of the petition) were published assessing randomized controlled trials comparing HES solutions with crystalloids and other colloids.

- A Cochrane meta-analysis (Perel et al.) assessed mortality rates in 70 randomized controlled trials that compared colloids (HES, dextran, gelatin, albumin, or plasma protein fraction) with crystalloids in critically ill subjects, including surgical and trauma patients.[159] In the 25 trials (N = 9,147) comparing HES solutions to crystalloids, the pooled RR for mortality in the HES group was 1.10 (95% CI 1.02 – 1.19; removing the trials authored by Joachim Boldt did not change these results). There was no significant risk of increased mortality associated with any of the other assessed colloids.

  The authors concluded that "[t]here is no evidence from randomised controlled trials that resuscitation using colloids compared with crystalloids reduces the risk of death in patients with trauma, burns or following surgery. The use of hydroxyethyl starch might even increase mortality. Since colloid use is not associated with improved survival and colloids are considerably more expensive than crystalloids, it is hard to see how their continued use in clinical practice can be justified."[160]

- Gattas et al. performed a meta-analysis of 35 randomized controlled trials that assessed the risk of mortality and the need for renal replacement therapy in 10,391 hospitalized adults who were acutely ill or undergoing major surgery and who received either low-molecular-weight 6% HES (130/0.4 or 130/0.42) solutions or other crystalloid or colloid fluid.[161] It reported a numerically increased risk of mortality (RR 1.08; 95% CI 1.00 – 1.17) and a significant increase in the risk of treatment with renal replacement therapy (RR 1.25; 95% CI 1.08 – 1.44) in the HES-treated groups. The three largest trials, published or completed in 2012, contributed 77% of the studied subjects.

- Zarychanski et al. published a meta-analysis of 38 randomized controlled trials comparing HES solutions to crystalloids, albumin, or gelatin, assessing the outcomes of mortality and acute renal failure in critically ill subjects receiving acute volume resuscitation.[162] For the 35 trials (N = 10,880) with mortality data, HES solutions were numerically, but not statistically significantly, associated with a higher rate of mortality (RR 1.07; 95% CI 1.00 – 1.14). However, after excluding seven trials (N = 590) authored

[158] Mutter TC, Ruth CA, Dart AB. Hydroxyethyl starch (HES) versus other fluid therapies: effects on kidney function. *Cochrane Database Syst Rev*. 2013;7:CD007594.
[159] Perel P, Roberts I, Ker K. Colloids versus crystalloids for fluid resuscitation in critically ill patients. *Cochrane Database Syst Rev*. 2013;2:CD000567.
[160] *Ibid.*
[161] Gattas DJ, Dan A, Myburgh J, Billot L, Lo S, Finfer Set al; CHEST Management Committee. Fluid resuscitation with 6% hydroxyethyl starch (130/0.4 and 130/0.42) in acutely ill patients: systematic review of effects on mortality and treatment with renal replacement therapy. *Intensive Care Med*. 2013;39(4):558-568.
[162] Zarychanski R, Abou-Setta AM, Turgeon AF, et al. Association of hydroxyethyl starch administration with mortality and acute kidney injury in critically ill patients requiring volume resuscitation: A systematic review and meta-analysis. *JAMA*. 2013;309(7):678-688.

by Joachim Boldt, the association of HES solutions with higher rates of mortality reached statistical significance (RR 1.09; 95% CI 1.02 – 1.17). HES solutions also led to increased rates of renal replacement therapy (RR 1.32; 95% CI 1.15 – 1.50; 10 trials, N = 9,258) and acute renal failure (RR 1.27; 95% CI 1.09 – 1.47; 5 trials, N = 8,725).

- Haase et al. published a meta-analysis of nine randomized controlled trials of 3,456 subjects with sepsis that compared HES 130/0.38 or HES 130/0.45 solutions with either crystalloids or albumin.[163] HES solutions were not associated with an increased risk of death over all of the trials (RR1.04; 95% CI 0.89 – 1.22). When analyzing only the four trials (N = 3,016 subjects) with "low risk of bias," increased mortality was numerically higher with HES (RR 1.11; 95% CI 1.00 – 1.23), although the difference in mortality between the subgroups of trials with low- and high-risk of bias was not significant.

  HES solutions were associated with significantly increased risks of renal replacement therapy (RR 1.36; 95% CI 1.08 – 1.72), red blood cell transfusions (RR 1.29; 95% CI 1.13 – 1.48; although the transfused volume of red blood cells did not differ significantly between the groups), and serious adverse events (RR 1.30; 95% CI 1.02 – 1.67). Sensitivity analyses accounting for sparse data across the analyzed trials confirmed the finding of increased risk of renal replacement therapy and red blood cell transfusion.

- Patel et al. published a meta-analysis of six randomized trials of low-molecular-weight HES (130/0.4 or 130/0.42) solutions in 3,033 subjects with severe sepsis.[164] HES solutions were associated with a higher rate of 90-day mortality (RR 1.13; 95% CI: 1.02 – 1.25) and renal replacement therapy (RR 1.42; 95% CI: 1.09 – 1.85).

[163] Haase N, Perner A, Hennings LI, et al. Hydroxyethyl starch 130/0.38-0.45 versus crystalloid or albumin in patients with sepsis: systematic review with meta-analysis and trial sequential analysis. *BMJ*. 2013;346:f839.
[164] Patel A, Waheed U, Brett SJ. Randomised trials of 6% tetrastarch (hydroxyethyl starch 130/0.4 or 0.42) for severe sepsis reporting mortality: systematic review and meta-analysis. *Intensive Care Med*. 2013;39(5):811-822.

Feb. 8, 2017

Dangerous IV Solutions Must Be Banned Immediately, Public Citizen Tells FDA

Hydroxyethyl Starch Solutions Cause Life-Threatening Side Effects; Safer, Equally Effective and Cheaper Alternatives Are Available

WASHINGTON, D.C. – A type of intravenous (IV) solution used mainly in hospitals should be taken off the market because it causes life-threatening side effects and safer, equally effective IV solutions are readily available, Public Citizen said today in petition (https://www.citizen.org/our-work/health-and-safety/petition-fda-ban-hydroxethyl-starch-solutions) to the U.S. Food and Drug Administration (FDA). Joining Public Citizen as co-petitioners are two internationally recognized experts in critical care medicine.

Hydroxyethyl starch (HES) solutions have been marketed since 1972 as an option for treating patients who need IV solutions to maintain or increase their fluid volume. Three branded versions are available in the U.S.: Hespan (made by B. Braun Medical, Inc.), Hextend (made by BioTime, Inc.) and Voluven (made by Fresenius Kabi Norge A.S.).

Soon after HES solutions came on the market, evidence emerged that they could impede the blood's ability to clot properly and that they remained stored in body tissues long after being administered. Later evidence showed that HES solutions can cause kidney failure and death. That such evidence emerged only after the solutions were approved was not surprising, give that their approval was based on small, brief and poorly designed clinical trials.

In 2013, the FDA issued a safety alert (http://www.fda.gov/BiologicsBloodVaccines/SafetyAvailability/ucm358271.htm) warning doctors and patients of these side effects and warned against using HES solutions in critically ill patients, including those with sepsis because of the increased risk of death and kidney damage. However, the agency, with no justification, allowed the solutions continue to be used in other patients, including those undergoing major surgery and treatment for trauma, despite evidence showing that HES solutions had the same dangers in all patients.

Also in 2013, a key committee of the European Medicines Agency (EMA) voted to ban HES solutions in Europe, only to reverse the decision a few months later in a split decision, with 14 of the 33 members of the committee voting against the reversal and issuing a dissenting opinion (http://www.ema.europa.eu/docs/en_GB/document_library/Referrals_document/Hydroxyethyl starch-containing_medicines_107/Recommendation_provided_by_Pharmacovigilance_Risk_Assessment_Committee/WC500154254.pdf) that detailed the reasons the risks of HES solutions outweighed their (nonunique) benefits in all patients.

In its petition (https://www.citizen.org/our-work/health-and-safety/petition-fda-ban-hydroxethyl-starch-solutions), Public Citizen offers a point-by-point rebuttal – supported by a comprehensive review of the scientific literature – of the arguments offered by the producers of HES solutions and others for keeping the products on the market. The petition also explains that HES solutions offer no unique benefit over several other types of available intravenous solutions and that there is therefore no justifiable reason to continue to expose patients to the unique risks of HES products.

“The evidence on the dangers of HES solutions is overwhelming,” said Dr. Sammy Almashat, researcher with Public Citizen’[illegible] Health Research Group and a co-author of the petition. “There is no good justification, other than corporate profits, to subjec[illegible] any patient to these dangers when there are so many equally effective but much safer alternatives available.”

In addition to Public Citizen, two other co-petitioners, signing on their own behalf, are leading experts on critical care and flui[illegible] management. Both joined 74 other experts around the world in co-signing a 2014 open letter (https://academic.oup.com/bja/article/112/3/595/248371/Open-letter-to-the-Executive-Director[illegible] of-the#2932328) to the EMA that urged the agency to ban HES solutions in Europe.

One co-petitioner is Dr. Charles Natanson, who has published extensively on the risks of HES products, including three papers as part of his work as a senior investigator and chief of the Anesthesia Section in the National Institutes of Health’s (NIH’s) Clinical Center's Critical Care Medicine Department.

“HES fulfills no unmet medical need, and there are excellent alternatives available without the unacceptable toxicities of HES[illegible] said Natanson. “The FDA and EMA should fulfill their mission to protect the public health and, without delay, ban HES solutions in the U.S. and Europe.”

The other co-petitioner, Dr. Ian Roberts, coordinating editor of the Cochrane Injuries Group and co-director of the Clinical Trials Unit at the London School of Hygiene & Tropical Medicine, added, “Medicines should be licensed for use only if they a[illegible] safe and effective. HES products are clearly not and should be banned worldwide.” Roberts also has published several articles on the risks of HES products.

In addition to filing a petition (https://www.citizen.org/our-work/health-and-safety/petition-fda-ban-hydroxethyl-starch-solutions) with the FDA, Public Citizen and its two co-petitioners sent a copy of the petition to th[illegible] EMA’s executive director, Dr. Guido Rasi, along with a letter (https://www.citizen.org/our-work/health-and-safety/letter-ema-urging-ban-hydroxyethyl-starch-solutions-europe) urging the EMA to reconsider its 2013 decision and ban HES products in Europe.

###
