# Supplementary material for: Starch Wars—New Episodes of the Saga. Changes in Regulations on Hydroxyethyl Starch in the European Union
Source: Front Vet Sci. 2019 Jan 18;5:336. doi: 10.3389/fvets.2018.00336 (PMC6345713; doi:10.3389/fvets.2018.00336)
Supplement: Supplementary file 5 [file Data_Sheet_5.PDF]

The European Union electronic Register of Post-Authorisation Studies (EU PAS Register)

Status: Finalised **Last updated on:** 08/06/2018

1. Study identification

Official title **EU PAS Register Number** EUPAS10897

Retrospective Drug Utilisation Study to investigate the routine use of Hydroxyethyl Starch (HES)- containing Infusion Solutions in Hospital

Study title acronym

NA

Study type

Observational study

Brief description of the study

The objective of the Drug Utilisation Study (DUS) is to assess the adherence of hospital physicians to the revised European Product Information (PI) [Summary of Product Characteristics (SmPC);
Package Leaflet) for Hydroxyethyl Starch (HES) - containing medicinal products concerning indication, posology (dosage) and contraindications.

Was this study requested by a regulator?

Yes

Germany

Is the study required by a Risk Management Plan (RMP)?
EU RMP category 1 (imposed as condition of marketing authorisation)

Other study registration identification numbers and URLs as applicable

2. Research centres and Investigator details

Coordinating study entity

**Centre name**	Kantar Health GmbH Germany
**Centre location**	Munich, Germany

Details of (Primary) lead investigator

**Title**	Miss
**Last name**	Apecechea
**First name**	Mercedes

© European Medicines Agency, 2018. Reproduction is authorised provided the source is acknowledged.

Is this study being carried out with the collaboration of a research network?

No

Other centres where this study is being conducted

Multiple centres

In total how many centres are involved in this Study?

45

TBD, TBD

Countries in which this study is being conducted

International study

Austria
Belgium
Czech Republic
France
Germany
Hungary
Netherlands
Poland
Spain

3 Study timelines: initial administrative steps, progress reports and final report

	Planned	Actual
Date when funding contract was signed	01/07/2015	09/12/2015
Start date of data collection	01/10/2015	18/05/2016
Start date of data analysis	01/10/2016	05/12/2016
Date of interim report, if expected		
Date of final study report	31/03/2017	06/07/2017

4. Sources of funding

Please provide estimates of the percentage of funding by source for this study

	Name(s)	Approximate % funding
Pharmaceutical companies	Fresenius Kabi Deutschland GmbH	100
Charities		
Government body		
Research councils		
EU funding scheme		
Other		

5. Contact details for enquiries

Scientific Enquiries

**Title**	Mr
**Last name**	Contact
**First name**	Scientific
**Address line 1**	Else-Kröner-Straße 1
**Address line 2**	
**Address line 3**	
**City**	Bad Homburg
**Postcode**	61352
**Country**	Germany
**Phone number (incl. country code)**	004961726864970
**Alternative phone number**	
**Fax number (incl. country code)**	
**Email address**	HES-DUS_ENCePP-enquiry@fresenius-kabi.com

Public Enquiries

**Title**	Mr
**Last name**	Contact
**First name**	Public
**Address line 1**	Else-Kröner-Straße 1
**Address line 2**	
**Address line 3**	
**City**	Bad Homburg
**Postcode**	61352
**Country**	Germany
**Phone number (incl. country code)**	004961726864970
**Alternative phone number**	
**Fax number (incl. country code)**	
**Email address**	HES-DUS_ENCePP-enquiry@fresenius-kabi.com

6. Study drug(s) information

Substance class (ATC Code)	B05AA07 (hydroxyethylstarch)

7. Medical conditions to be studied

Medical condition(s) No

8. Population under study

Age

Preterm newborns
Term newborns (0-27 days)
Infants and toddlers (28 days - 23 months)
Children (2 - 11 years)
Adolescents (12 - 17 years)
Adults (18 - 44 years)
Adults (45 - 64 years)
Adults (65 - 74 years)
Adults (75 years and over)

Sex

Male
Female

9. Number of patients

Estimated total number of subjects 3000

Additional information

approx. 334 patients per country

10. Source of data

Is this study being carried out with an established data source? No

Sources of data

Retrospective non-interventional Patient Chart Study (based on medical records)

11. Scope of the study

What is the scope of the study?

Drug utilisation study

Primary scope: Drug utilisation study

12. Main objectives(s)

What is the main objective of the study?

The objective of the DUS is to assess the adherence of hospital physicians to the revised European PI for HES-containing medicina! products concerning indication, posology (dosage), and contraindications.

Are there primary outcomes? Yes

Outcome measure is the adherence to the concerned sections of the
revised European PI for HES-containing medicinal products. Usage of these medicinal products according to indications, posolagy (dosage), and contraindications as specified in the revised PI of
the HES solutions will be assessed.

Are there secondary outcomes? No

13. Study design

What is the design of the study?

Drug utilisation study

14. Follow-up of patients

Will patients be followed up?

Not applicable/no follow-up

15. Data analysis plan

Please provide a brief summary of the analysis method

Because of the exploratory character of this study only descriptive statistics will be performed. All parameters will be presented as mean +/- standard deviation for continuous normally distributed variables and median (25th; 75th percentile) for ordinal and continuous non-normal
(skewed) variables. Categorical variables will be presented as percentage (and 95 % confidence interval). All analyses will be performed for the overall population as well as for each country separately. Ail data will be examined for the overall population and subgroups (each site/country separately) specified by indication and
contraindications according to the revised PI.

16. ENCePP seal

Are you requesting the ENCePP seal for this study? No

17. Full protocol

Document
HE06-022PW-CNI-HESDUS_CSP
blackened_all.pdf

18. Study Results

Document
HE06-022-CNI-HESDUSCSR-Abstract-ges
ch.pdf

Please list the 5 most relevant publications using data from your study

None

19. Other relevant documents

Conflict(s) of interest of investigator(s)

Not Submitted

Composition of Steering Group and Observers

Not Submitted

Other documents

Not Submitted

Signed Code of Conduct Checklist

Not Submitted

Signed Code of Conduct Declaration

Not Submitted

Methodological Checklist

Not Submitted

**PDF documents can be downloaded and printed from the tab "Documents" in the study entry*

The European Union electronic Register of Post-Authorisation Studies (EU PAS Register)

Status: Finalised **Last updated on:** 09/02/2018

1. Study identification

Official title **EU PAS Register Number** EUPAS12540

Retrospective Drug Utilisation Study to investigate the routine use of Hydroxyethyl Starch (HES)-containing Infusion Solutions of B. Braun Melsungen AG in Hospitals

Study title acronym

None

Study type

Observational study

Brief description of the study

The objective of the Drug Utilisation Study (DUS) is to assess the adherence of hospital physicians to the revised European Product Information (PI) [Summary of Product Characteristics (SmPC); Package Leaflet] for Hydroxyethyl Starch (HES) - containing medicinal products of B. Braun Melsungen AG concerning indication, posology (dosage) and contraindications.

Was this study requested by a regulator?

Yes

Sweden

Is the study required by a Risk Management Plan (RMP)?

EU RMP category 1 (imposed as condition of marketing authorisation)

Other study registration identification numbers and URLs as applicable

2. Research centres and Investigator details

Coordinating study entity

**Centre name**	B. Braun Melsungen AG
**Centre location**	Carl-Braun-Str. 1, 34212 Melsungen, Germany

Details of (Primary) lead investigator

**Title**	Mr
**Last name**	Greger
**First name**	Jens

© European Medicines Agency, 2018. Reproduction is authorised provided the source is acknowledged.

Is this study being carried out with the collaboration of a research network?

No

Other centres where this study is being conducted

Multiple centres

In total how many centres are involved in this Study?

40

Countries in which this study is being conducted

International study

Belgium
Czech Republic
France
Germany
Italy
Netherlands
Poland
Spain
Sweden

3 Study timelines: initial administrative steps, progress reports and final report

	**Planned**	**Actual**
Date when funding contract was signed	14/09/2015	14/09/2015
Start date of data collection	01/03/2016	01/03/2016
Start date of data analysis	28/02/2017	28/02/2017
Date of interim report, if expected		
Date of final study report	06/11/2017	22/09/2017

4. Sources of funding

Please provide estimates of the percentage of funding by source for this study

	**Name(s)**	**Approximate % funding**
Pharmaceutical companies	B.Braun Melsungen AG	100
Charities		
Government body		
Research councils		
EU funding scheme		
Other		

5. Contact details for enquiries

Scientific Enquiries

**Title**	Mr
**Last name**	Contact

**First name**	Scientific
**Address line 1**	Carl-Braun-Str. 1
**Address line 2**	
**Address line 3**	
**City**	34212
**Postcode**	
**Country**	Germany
**Phone number (incl. country code)**	00495661714498
**Alternative phone number**	
**Fax number (incl. country code)**	
**Email address**	dus-hes130-encepp@bbraun.com

Public Enquiries

**Title**	Mr
**Last name**	Contact
**First name**	Public
**Address line 1**	Carl-Braun-Str. 1
**Address line 2**	
**Address line 3**	
**City**	34212
**Postcode**	
**Country**	Germany
**Phone number (incl. country code)**	495661714498
**Alternative phone number**	
**Fax number (incl. country code)**	
**Email address**	dus-hes130-encepp@bbraun.com

6. Study drug(s) information

Substance class (ATC Code)	B05AA07 (hydroxyethylstarch)

7. Medical conditions to be studied

Medical condition(s) No

8. Population under study

Age

Preterm newborns
Term newborns (0-27 days)
Infants and toddlers (28 days - 23 months)
Children (2 - 11 years)
Adolescents (12 - 17 years)
Adults (18 - 44 years)
Adults (45 - 64 years)
Adults (65 - 74 years)
Adults (75 years and over)

Sex

Male
Female

9. Number of patients

Estimated total number of subjects 3000

10. Source of data

Is this study being carried out with an established data source? No

Sources of data

Retrospective non-interventional patient chart study

11. Scope of the study

What is the scope of the study?

Drug utilisation study

Primary scope: Drug utilisation study

12. Main objectives(s)

What is the main objective of the study?

The objective of the DUS is to assess the adherence of hospital physicians to the revised European PI for HES-containing medicinal products of B. Braun Melsungen AG concerning indication, posology (dosage) and contraindications.

Are there primary outcomes? Yes

Outcome measure is the adherence to the concerned sections of the revised European PI. Usage of these medicinal products according to indications, posology (dosage), and contraindications as specified in the revised PI of the HES solutions will be assessed.

Are there secondary outcomes? No

13. Study design

What is the design of the study?

Drug utilisation study

14. Follow-up of patients

Will patients be followed up?

Not applicable/no follow-up

15. Data analysis plan

Please provide a brief summary of the analysis method

Because of the exploratory character of this study only descriptive statistics will be performed. All parameters will be presented as mean +/- standard deviation for continuous normally distributed variables and median (25th; 75th percentile) for ordinal and continuous non-normal (skewed) variables. Categorical variables will be presented as percentage (and 95 % confidence interval). All analyses will be performed for the overall population as well as for each country separately. All data will be examined for the overall population and subgroups (each site/country separately) specified by indication and contraindications according to the revised PI.

16. ENCePP seal

Are you requesting the ENCePP seal for this study? No

17. Full protocol

Not Submitted

18. Study Results

Not Submitted

Please list the 5 most relevant publications using data from your study

None

19. Other relevant documents

Conflict(s) of interest of investigator(s)

Not Submitted

Composition of Steering Group and Observers

Not Submitted

Other documents

Not Submitted

Signed Code of Conduct Checklist

Not Submitted

Signed Code of Conduct Declaration

Not Submitted

Methodological Checklist

Not Submitted

**PDF documents can be downloaded and printed from the tab "Documents" in the study entry*
