# Supplementary material for: Starch Wars—New Episodes of the Saga. Changes in Regulations on Hydroxyethyl Starch in the European Union
Source: Front Vet Sci. 2019 Jan 18;5:336. doi: 10.3389/fvets.2018.00336 (PMC6345713; doi:10.3389/fvets.2018.00336)
Supplement: Supplementary file 6 [file Data_Sheet_6.PDF]

1. BEZEICHNUNG DES ARZNEIMITTELS

Gelafundin 4 % Infusionslösung

2. QUALITATIVE UND QUANTITATIVE ZUSAMMENSETZUNG

1000 ml Infusionslösung enthalten:

Gelatinepolysuccinat 40,00 g
($\overline{M}_w$ 30 000, $\overline{M}_n$ 23 200)

Elektrolyte:
Natrium 154 mmol/l (3535 mg/l)
Chlorid 120 mmol/l

Sonstige Bestandteile

1000 ml Infusionslösung enthalten 154 mmol Natrium als Natriumchlorid

3. DARREICHUNGSFORM

Infusionslösung
klare schwach gelbliche Lösung

pH-Wert:	7,1 – 7,7
Theoretische Osmolarität:	274 mOsm/l
Gelierpunkt:	< 3 °C

4. KLINISCHE ANGABEN

4.1 Anwendungsgebiete

Als kolloidales Volumenersatzmittel zur
- Prophylaxe und Therapie von Volumenmangel und Schock
- akuten normovolämischen Hämodilution

4.2 Dosierung, Art und Dauer der Anwendung

Die tägliche Dosis und die Infusionsgeschwindigkeit richten sich nach der Höhe des vorangegangenen Blutverlusts, dem individuellen Bedarf zur Aufrechterhaltung bzw. Wiederherstellung der Normovolämie und hämodynamischen Stabilität sowie nach dem Ausmaß der Hämodilution. Als Entscheidungskriterien kommen u. a. in Betracht: Zentralvenöser Druck (ZVD), Blutdruck, Herzfrequenz, Diureserate, Hämoglobinkonzentration bzw. Hämatokrit.

Maximale Tagesmenge:

Die maximale tägliche Dosis wird durch den Grad der Hämodilution bestimmt. Der als kritisch anzusehende Hämoglobin- oder Hämatokritwert ist eine patientenindividuelle Größe, die sich u. a. nach der Sauerstoffausschöpfung der Kapillaren, dem Alter, den Kreislaufreserven und dem klinischen Zustand richtet. Bei Patienten ohne erhöhten Sauerstoffbedarf und mit uneingeschränkten Kompensationsmechanismen gilt eine Hämodilution bis zu einer Hb-Konzentration von 8 g/dl als tolerabel, bei Intensivpatienten jedoch nur bis zu einer Hb-Konzentration von 10 g/dl.

Außer auf die Verdünnung des Hämoglobins ist auch auf die Verdünnung von Plasmaproteinen, insbesondere von Gerinnungsfaktoren, und deren bedarfsgerechte Substitution zu achten.

Infusionsgeschwindigkeit:

Die Infusionsgeschwindigkeit richtet sich nach der klinischen Situation und den Kreislaufverhältnissen des Patienten. Bei Patienten im Schock können bis zu 20 ml Gelafundin 4 % pro kg Körpergewicht und Stunde infundiert werden (entsprechend 0,33 ml pro kg Körpergewicht in der Minute).

In akuten Notfällen kann eine Druckinfusion erfolgen. In diesem Fall ist darauf zu achten, dass Behältnis und Infusionssystem vor dem Anlegen der Infusion vollständig entlüftet werden.

Bei Verwendung als Druckinfusion (Druckmanschette, Infusionspumpe) sollte Gelafundin 4 % zuvor auf Körpertemperatur erwärmt werden.

Bei zu hoher Infusionsgeschwindigkeit besteht die Gefahr der Kreislaufüberlastung.

Art und Dauer der Anwendung

Zur intravenösen Anwendung.

Um eventuelle anaphylaktoide Reaktionen (s. Abschnitt „Nebenwirkungen") frühzeitig erkennen zu können, sind die ersten 20 – 30 ml langsam und unter sorgfältiger Beobachtung des Patienten zu infundieren.

Die Anwendungsdauer richtet sich danach, wie lange die Hypovolämie bzw. die Kreislaufinstabilität besteht, und nach dem therapiebedingten Ausmaß der Hämodilution.

4.3 Gegenanzeigen

Gelafundin 4 % darf nicht angewendet werden bei
- einer bekannten Überempfindlichkeit gegen Gelatine
- Hypervolämie
- Überwässerung (Hyperhydratation)
- schwerer Herzinsuffizienz
- schweren Blutgerinnungsstörungen
- Hypernatriämie
- Hyperchlorämie

4.4 Besondere Warnhinweise und Vorsichtsmaßnahmen für die Anwendung

Gelafundin 4 % soll nur mit Vorsicht angewendet werden bei
- Dehydratationszuständen, da in diesem Fall primär der Wasserhaushalt korrigiert werden sollte
- Blutgerinnungsstörungen, da die Anwendung zu einer Verdünnung der Gerinnungsfaktoren führt
- Niereninsuffizienz, da der übliche Ausscheidungsweg beeinträchtigt sein kann
- chronischen Lebererkrankungen, da hier die Synthese von Albumin und Gerinnungsfaktoren gestört sein kann und die Anwendung eine weitere Verdünnung bewirkt.
- Lungenödem
- intrakraniellen Blutungen

Bei Fibrinogenmangel soll das Präparat nur in lebensbedrohlichen Notfällen verabreicht werden.

Elektrolyte sind nach Bedarf zu substituieren.

Anwendung bei Kindern
Über die Anwendung bei Kindern liegen keine ausreichenden Erfahrungen vor.

Notwendige Überwachungsmaßnahmen:
- Kontrollen des Serumionogramms und der Wasserbilanz sind erforderlich. Dies gilt im besonderen bei Hypernatriämie, Dehydratationszuständen und Niereninsuffizienz.
- Bei Blutgerinnungsstörungen und chronischen Lebererkrankungen sollten Gerinnungsparameter und Serumalbumin überwacht werden.
- Abhängig vom infundierten Volumen müssen Gerinnungsparameter und Albumin auch außerhalb chronischer Lebererkrankungen und Blutgerinnungsstörungen überwacht und ggf. substituiert werden.

Druckinfusion:
Falls in einer Notsituation die Anwendung über eine Druckinfusion erfolgen soll, so ist darauf zu achten, dass Behältnis und Infusionssystem vor Anlegen der Infusion vollständig entlüftet werden.

Beeinflussung von Laborwerten
Eine Beeinflussung klinisch-chemischer Parameter ist möglich. So können die Werte der folgenden Laborbestimmungen erhöht sein: Blutsenkungsgeschwindigkeit, spezifisches Gewicht des Urins sowie die unspezifische Proteinbestimmung (z. B. nach der Biuret-Methode). Diese Einflüsse klingen innerhalb von 24 Stunden nach Infusionsende vollständig ab.

Hinweis:
Die Anwendung von Gelafundin 4 % kann bei Dopingkontrollen zu positiven Ergebnissen führen.

1000 ml Infusionslösung enthalten 154 mmol (= 3535 mg) Natrium. Dies ist zu berücksichtigen bei Personen unter Natrium-kontrollierter (natriumarmer/kochsalzarmer) Diät.

4.5 Wechselwirkungen mit anderen Arzneimitteln und sonstige Wechselwirkungen

Nicht bekannt

4.6 Schwangerschaft und Stillzeit

Zu den reproduktionstoxischen Eigenschaften von Gelafundin 4 % liegen keine ausreichenden Erkenntnisse vor. Die Anwendung in der Schwangerschaft sollte wegen des nicht auszuschließenden Risikos einer anaphylaktischen/anaphylaktoiden Reaktion nur nach strenger Abwägung von Nutzen und Risiko erfolgen.

Angaben zum Übertritt von Gelafundin 4 % in die Muttermilch liegen nicht vor.

4.7 Auswirkungen auf die Verkehrstüchtigkeit und das Bedienen von Maschinen

Nicht zutreffend

4.8 Nebenwirkungen

Als potentiell schwerwiegende Nebenwirkungen sind die anaphylaktischen/anaphylaktoiden Reaktionen zu nennen, wie sie bei allen kolloidalen Volumenersatzmitteln auftreten können.

Bei der Bewertung von Nebenwirkungen werden folgende Häufigkeiten zugrunde gelegt:

Sehr häufig	(≥ 1/10)
Häufig	(≥ 1/100 bis < 1/10)
Gelegentlich	(≥ 1/1.000 bis < 1/100)
Selten	(≥ 1/10.000 bis < 1/1.000)

Sehr selten (< 1/10.000)
nicht bekannt: auf Grundlage der verfügbaren Daten nicht abschätzbar

Erkrankungen des Immunsystems
Selten: anaphylaktische/anaphylaktoide Reaktionen aller Schweregrade
Sehr selten: anaphylaktische/anaphylaktoide Reaktionen der Schweregrade III und IV
Weitere Angaben siehe Abschnitt „Anaphylaktische/anaphylaktoide Reaktionen“ unten.

Erkrankungen des Gastrointestinaltrakts
Gelegentlich: Übelkeit, abdominale Krämpfe.

Allgemeine Erkrankungen
Gelegentlich: Fieber

Anaphylaktische/anaphylaktoide Reaktionen

Nach Gelafundin 4 %-Infusionen können wie nach allen kolloidalen Volumenersatzmitteln anaphylaktische/anaphylaktoide Reaktionen verschiedener Schweregrade auftreten. Diese Reaktionen manifestieren sich als Hauterscheinungen (Urtikaria) oder führen über eine Rötung von Hals und Gesicht (Flush) in sehr seltenen Fällen zu Blutdruckabfall, Bronchospasmus, Schock, Herz- und Atemstillstand.
Patienten, die Gelafundin 4 % erhalten, müssen wegen möglicher anaphylaktischer/anaphylaktoider Reaktionen entsprechend überwacht werden.

Allgemeine Richtlinien zur Prophylaxe und Therapie von anaphylaktischen/anaphylaktoiden Reaktionen

- Ausreichende Information der Ärzte und des Pflegepersonals über Art und Schwere der möglichen Reaktionen bei Verabreichung eines kolloidalen Volumenersatzmittels.
- Strenge Beobachtung des Patienten unter der Infusion, besonders während die ersten 20 – 30 ml der Infusionslösung einlaufen.
- Bereitstellung aller zur Reanimation erforderlichen allgemeinen und medikamentösen Maßnahmen.
- Sofortiger Infusionsstop, falls sich anaphylaktische/anaphylaktoide Reaktionen andeuten.

Es ist durch kein Testverfahren voraussagbar, welche Patienten mit einer anaphylaktischen/anaphylaktoiden Reaktion reagieren. Der Verlauf einer Unverträglichkeit ist nicht vorhersehbar.

Anaphylaktische/anaphylaktoide Reaktionen auf Gelatinelösungen können sowohl histaminvermittelt als auch histaminunabhängig sein. Die Histaminfreisetzung kann durch eine Prophylaxe mit H_1- und H_2-Blockern gehemmt werden. Eine prophylaktische Kortikosteroidgabe hat sich nicht bewährt.

Anaphylaktische/anaphylaktoide Reaktionen können sowohl beim wachen als auch beim narkotisierten Patienten auftreten. In der Akutphase des Volumenmangelschocks sind anaphylaktische/anaphylaktoide Reaktionen bisher nicht bekannt geworden.

Die **Notfallbehandlung anaphylaktoider Reaktionen** wird entsprechend der Art und Schwere der Symptome gemäß etablierten Behandlungsschemata durchgeführt (z. B. Adams HA et al.: Empfehlungen zur Diagnostik und Therapie der Schockformen der IAG Schock der DIVI, Teil 4: Anaphylaktischer Schock. Intensivmed.42 (2005): 299 – 304).

Meldung des Verdachts auf Nebenwirkungen
Die Meldung des Verdachts auf Nebenwirkungen nach der Zulassung ist von großer Wichtigkeit. Sie ermöglicht eine kontinuierliche Überwachung des Nutzen-Risiko-Verhältnisses des Arzneimittels. Angehörige von Gesundheitsberufen sind aufgefordert, jeden Verdachtsfall einer Nebenwirkung dem Bundesinstitut für Arzneimittel und Medizinprodukte, Abt. Pharmakovigilanz, Kurt-Georg-Kiesinger-Allee 3, D-53175 Bonn, Website: www.bfarm.de anzuzeigen.

4.9 Überdosierung

Bei einer Überdosierung von Plasmaersatzpräparaten kann es zu einer unerwünschten Erhöhung des Kreislaufvolumens kommen. In der Folge können die Funktionen des Herzens und der Lunge beeinträchtigt werden.

Bei beginnender Kreislaufüberlastung durch Überdosierung (Dyspnoe, Halsvenenstauung) ist die Infusion sofort zu unterbrechen.

5. PHARMAKOLOGISCHE EIGENSCHAFTEN

5.1 Pharmakodynamische Eigenschaften

Pharmakotherapeutische Gruppe: Blutersatzmittel und Plasmaproteinfraktionen
ATC-Code: B05A A06

Gelafundin 4 % ist eine 4%ige Lösung von Gelatinepolysuccinat (auch als succinylierte oder modifizierte flüssige Gelatine bezeichnet) mit einem mittleren Molekulargewicht $\overline{M}_w$ (Gewichtsmittel) von 30 000 Dalton. Die durch die Succinylierung in das Molekül eingeführten negativen Ladungen führen zu einer Spreizung des Moleküls, das damit erheblich raumfüllender ist als nichtsuccinylierte Proteinketten gleichen Molekulargewichts.

Steigende Volumina von Gelafundin 4 % bewirken eine im gleichen Maß zunehmende Hämodilution und Verdünnung von Gerinnungsfaktoren. Dadurch kann es bei Verabreichung von hohen Dosen zur Verlängerung der aPTT oder Blutungszeit kommen.

5.2 Pharmakokinetische Eigenschaften

Die spezifischen Eigenschaften von Gelafundin 4 % gewährleisten eine ausreichende Volumenwirksamkeit für ca. 3 – 4 Stunden.

Die Ausscheidung erfolgt überwiegend renal. Nur eine geringe Menge wird über den Darm ausgeschieden und nur ca. 1 % der infundierten Dosis wird verstoffwechselt.

In 24 Stunden werden ca. 60 % der infundierten Gelatine im Urin ausgeschieden.

Verhalten von Gelatine bei Dialyse:
Bei Patienten mit dialysepflichtiger Niereninsuffizienz kommt es nicht zu einer relevanten Kumulation der Gelatine im Serum.

5.3 Präklinische Daten zur Sicherheit

In Tierversuchen konnten bei einmaliger und wiederholter Gabe keine Anzeichen für eine toxische Wirkung ermittelt werden. Die maximale Dosis wird nicht durch die applizierte Menge an Arzneistoff, sondern durch die Volumenzufuhr und die daraus resultierende Hypervolämie bzw. Hämodilution bestimmt.

6. PHARMAZEUTISCHE ANGABEN

6.1 Liste der sonstigen Bestandteile

Natriumchlorid,
Natriumhydroxid,
Salzsäure,
Wasser für Injektionszwecke

6.2 Inkompatibilitäten

Beim Mischen mit anderen Arzneimitteln können Inkompatibilitäten auftreten.

Gelafundin 4 % darf ohne vorausgehende Kompatibilitätsprüfung durch den Hersteller nicht mit anderen Arzneimitteln gemischt werden.

6.3 Dauer der Haltbarkeit

- *im ungeöffneten Originalbehältnis*

Polyethylenflasche (Ecoflac plus):	3 Jahre
Kunststoffbeutel (Ecobag):	2 Jahre

- *nach Anbruch des Behältnisses*

Nicht zutreffend. Siehe auch Abschnitt 6.6.

- *nach Zumischung von Additiven*

Aus mikrobiologischen Gründen sollten Mischungen sofort verwendet werden. Falls sie nicht sofort verwendet werden, liegen Lagerungszeiten und -bedingungen in der Verantwortung des Anwenders. Normalerweise sollten Mischungen mit anderen Komponenten nicht länger als 24 Stunden bei 2 °C – 8 °C gelagert werden, falls sie nicht unter kontrollierten und validierten aseptischen Bedingungen hergestellt wurden.

6.4 Besondere Vorsichtsmaßnahmen für die Aufbewahrung

Nicht unter 10 °C und nicht über 25 °C lagern.
Nicht einfrieren.

6.5 Art und Inhalt des Behältnisses

- Flaschen aus farblosem Polyethylen (Ecoflac plus), Inhalt: 500 ml, 1000 ml lieferbar in Packungen zu:
  1 × 500 ml, 10 × 500 ml
  1 × 1000 ml, 10 × 1000 ml
- Kunststoffbeutel (Ecobag), Inhalt: 500 ml, 1000 ml lieferbar in Packungen zu:
  1 × 500 ml, 20 × 500 ml
  1 × 1000 ml, 10 × 1000 ml

Es werden möglicherweise nicht alle Packungsgrößen in den Verkehr gebracht.

6.6 Besondere Vorsichtsmaßnahmen für die Beseitigung uns sonstige Hinweise zur Handhabung

Vorsichtsmaßnahmen für die Beseitigung

Nicht verwendetes Arzneimittel oder Abfallmaterial ist entsprechend den nationalen Anforderungen zu entsorgen.

Hinweise zur Handhabung

Nur zu verwenden, wenn das Behältnis unversehrt und die Lösung klar ist.

Die Behältnisse sind zur einmaligen Anwendung bestimmt. Nach einem Anwendungsgang nicht verbrauchte Infusionslösung ist zu verwerfen.

7. INHABER DER ZULASSUNG

B. Braun Melsungen AG
Carl-Braun-Straße 1
34212 Melsungen

Postanschrift:
34209 Melsungen

Tel.-Nr.: 05661-71-0
Fax-Nr.: 05661-71-4567

8. ZULASSUNGSNUMMER

36326.00.00

9. DATUM DER ERTEILUNG DER ZULASSUNG/VERLÄNGERUNG DER ZULASSUNG

17.06.1998/17.01.2006

10. STAND DER INFORMATION

März 2015

11. VERKAUFSABGRENZUNG

Apothekenpflichtig

Zentrale Anforderung an:

Rote Liste Service GmbH

Fachinfo-Service

Mainzer Landstraße 55

60329 Frankfurt

We use cookies to personalize your ads and to analyze traffic on our site. Learn more OK

Gelaspan Solution For Infusion

Projekt18_Layout 1 12.01.15 15:53 Seite 1

Projekt18_Layout 1 12.01.15 15:53 Seite 1

Gelaspan solution for infusion

SUMMARY OF PRODUCT CHARACTERISTICS

B. Braun Melsungen AG • 34209 Melsungen, Germany

1. NAME OF THE MEDICINAL PRODUCT

Gelaspan solution for infusion

2. QUALITATIVE AND QUANTITATIVE COMPOSITION 1000 ml solution contain:

Succinylated gelatine (= modified fluid gelatine) 40.0 g

(Molecular weight, weight average: 26 500 Dalton)

Sodium chloride 5.55 g

Sodium acetate trihydrate 3.27 g

Potassium chloride 0.30 g

Calcium chloride dihydrate 0.15 g

Magnesium chloride hexahydrate 0.20 g

Electrolyte concentrations

Sodium 151 mmol/l

Chloride 103 mmol/l

Potassium 4 mmol/l

Calcium 1 mmol/l

Magnesium 1 mmol/l

Acetate 24 mmol/l

Excipients:

For a full list of excipients see section 6.1.

3. PHARMACEUTICAL FORM

Solution for infusion

Clear, colourless or slightly yellowish solution

Theoretical osmolarity: 284 mosmol/l

pH: 7.4 ± 0.3

4. CLINICAL PARTICULARS

4.1 Therapeutic indications

Gelaspan is a colloidal plasma volume substitute in an isotonic, fully balanced electrolyte solution for:

- Prophylaxis and treatment of imminent or manifest relative or absolute hypo-volaemia and shock

4.2 Posology and method of administration

Posology

Dosage and infusion rate are adjusted according to the amount of blood loss and to individual needs for restoration and maintenance of a stable haemodynamic situation, respectively. The dose administered is initially 500 to 1000 ml on average, in case of severe blood loss higher doses have to be applied.

Adults

In adults, 500 ml is administered at an appropriate rate depending on the haemodynamic status of the patient. In the case of more than 20 per cent blood loss usually blood or blood components should be given in addition to Gelaspan.

Paediatric population

The safety and efficacy of Gelaspan in children have not yet been completely established. Therefore, no recommendation on a posology can be made. Gelaspan should only be administered to these patients if the expected benefits clearly outweigh potential risks. In those cases the patient's prevailing clinical condition should be taken into account and the therapy should be monitored especially carefully. (See also section 4.4.)

Maximum dose:

The maximum daily dose is determined by the degree of haemodilution. Care must be taken to avoid a decrease of the haematocrit below critical values, see section **4.4.**

If necessary, blood or packed red cells must be transfused additionally.

Attention must also be paid to the dilution of plasma proteins (e.g. albumin and coagulation factors), which must be adequately substituted if necessary.

Infusion rate:

The infusion rate depends on the actual haemodynamic situation.

The first 20 - 30 ml of solution should be infused slowly in order to detect rare anaphylactoid reactions as early as possible. See also sections **4.4** and **4.8.**

In shock situations, Gelaspan may be infused rapidly by pressure infusion, 500 ml within 5 - 10 min.

Method of administration Intravenous use

When given rapidly Gelaspan should be warmed to not more than 37°C if possible. In case of pressure infusion, which might be necessary in vital emergencies, all air must be removed from the container and the infusion set before the solution is administered.

4.3 Contraindications

Gelaspan must not be used in the following situations:

- hypersensitivity to gelatine solutions or to any of the other ingredients of Gelaspan
- hypervolaemia
- hyperhydration
- hyperkalaemia

343/NP34332/0115

4.4 Special warnings and precautions for use

Gelaspan should be administered with caution to patients with a history of allergic diseases, e.g. asthma.

Gelatine preparations for volume replacement may rarely cause allergic (anaphylac-tic/anaphylactoid) reactions of varying degrees of severity. In order to detect the occurrence of an allergic reaction as early as possible, the first 20 - 30 ml should be infused slowly and the patient should be under careful observation especially at the beginning of the infusion. For symptoms of anaphylactoid reactions, see section **4.8.** In case of an allergic reaction, the infusion must be stopped immediately and appropriate treatment given.

Gelaspan should be administered only with caution to patients

- at risk due to circulatory overload e.g. patients with congestive heart failure, right or left ventricular insufficiency, hypertension, pulmonary oedema or renal insufficiency with oligo- or anuria.
- with severely impaired renal function
- having oedema with water/salt retention
- with major blood coagulation disorders

Gelaspan must not be infused through the same infusion line together with blood or blood products (packed cells, plasma and plasma fractions).

Checks of serum electrolyte concentrations and water balance are necessary, in particular in patients with hypernatraemia, hyperkalaemia or impairment of renal function.

The haemodynamic, haematological and coagulation system should be monitored. During compensation of severe blood losses by infusions of large amounts of Gelaspan, haematocrit and electrolytes must be monitored. The haematocrit should not decrease below 25 %. In elderly or critically ill patients it should not fall below 30%. Likewise in those situations the dilution effect on coagulation factors should be observed, especially in patients with existing disorders of haemostasis.

Because the product does not substitute lost plasma protein, it is advisable to check the plasma protein concentrations, see also section **4.2**, "Maximum dose". *Paediatric population*

There is no sufficient experience with the use of Gelaspan in children. Therefore Gelaspan should only be administered to these patients if the expected benefits clearly outweigh potential risks. (See also section 4.2)

Influence on laboratory tests

Laboratory blood tests (blood group or irregular antigens) are possible after Gelaspan infusions. Nevertheless it is recommended to draw blood samples before the infusion of Gelaspan in order to avoid hampered interpretation of results.

Gelaspan may have an influence on the following clinical-chemical tests, leading to falsely high values:

- erythrocyte sedimentation rate,
- specific gravity of urine,
- unspecific protein assays, e.g. the biuret method.

4.5 Interactions with other medicinal products and other forms of interaction

Caution should be exercised in patients concurrently taking or receiving medicinal products that can cause potassium (e.g. potassium sparing diuretics, ACE inhibitors) or sodium retention.

4.6 Fertility, pregnancy and lactation *Women of childbearing potential*

No data available

Contraception in males and females

No data available

Pregnancy

There are no or limited amount of data from the use of Gelaspan in pregnant women. Animal studies are insufficient with respect to reproductive toxicity (see section 5.3).

Due to possible anaphylactoid reactions with consecutive foetal and neonatal distress due to maternal hypotension, the use of Gelaspan should be avoided during pregnancy unless the clinical condition of the woman requires treatment with the medicinal product.

Breastfeeding

There is insufficient information on the excretion of Gelaspan in human or animal milk. A risk to the suckling child cannot be excluded. A decision must be made whether to discontinue breast-feeding or to discontinue/abstain from Gelespan therapy taking into account the benefit of breast feeding for the child and the benefit of therapy for the woman.

Fertility

There are no data on the effect of Gelaspan on human or animal fertility.

4.7 Effects on ability to drive and use machines Not relevant.

4.8 Undesirable effects

The only potentially serious adverse reactions are anaphylactoid reactions described below. However, severe reactions are very rare.

**Approval for Printing BIBRAUN** Melsungen AG	
Approved for Printing	□
Approved for Printing when corrected	□
New draft required	□
Date	Signature
Name in capital letters	

BIBRAUN

#-----------#-----------#-----------#

PACKAGE LEAFLET: INFORMATION FOR THE USER

B. Braun Melsungen AG • 34209 Melsungen, Germany

Gelaspan solution for infusion

Read all of this leaflet carefully before you start using this medicine.

- Keep this leaflet. You may need to read it again.
- If you have any further questions, ask your doctor or pharmacist.
- This medicine has been prescribed for you. Do not pass it on to others. It may harm them, even if their symptoms are the same as yours.
- If any of the side effects gets serious, or if you notice any side effects not listed in this leaflet, please tell your doctor or pharmacist.

In this leaflet:

1. What Gelaspan is and what it is used for
2. Before you use Gelaspan
3. How to use Gelaspan
4. Possible side effects
5. How to store Gelaspan
6. Further information **1. WHAT GELASPAN IS AND WHAT IT IS USED FOR**

Gelaspan is a so-called plasma volume substitute. This means that it replaces fluid lost from the circulation.

Gelaspan is used to replace blood and body fluid, which have been lost as a result of, for example, an operation, an accident or a burn.

2. BEFORE YOU USE GELASPAN

Do not use Gelaspan

- if you are allergic (hypersensitive) to gelatine or any of the other ingredients of Gelaspan
- if your blood volume is too high
- if you have too much water in your body
- if you have an abnormally high blood potassium level.

Special care will be taken with Gelaspan

if you suffer from:

- heart problems
- high blood pressure
- water on your lungs
- severe kidney problems

Giving large amounts of liquids through an intravenous drip may worsen your condition.

Your doctor will also exercise caution

- if your blood clotting is severely impaired
- if you retain water and salt, which may be associated with tissue swelling.

<u>Children:</u>

There is only little experience regarding the use of Gelaspan in children. So the doctor will only administer this medicine to your child when he/she thinks that it is absolutely necessary.

All plasma substitutes carry a slight risk of allergic reactions that are mostly mild or moderate but can in very few cases also become severe. Such reactions are assumed to be more frequent in patients with known allergic conditions such as asthma. For that reason you will be under close observation by a health professional, especially at the beginning of the infusion. While receiving Gelaspan, your blood composition will be monitored.

Taking or using other medicines

Please tell your doctor or pharmacist if you are taking or using or have recently taken or used any other medicines, including medicines obtained without a prescription.

In particular your doctor should know if you are taking or receiving medicines that make you retain sodium or potassium (e.g. spironolactone, triamterene, amiloride; ACE-inhibitors like captopril or enalapril), such as certain water tablets or cortisones.

Pregnancy and breast-feeding

Ask your doctor or pharmacist for advice before taking any medicine.

Your doctor will give you Gelaspan only if he thinks it is essential for you.

Driving and using machines

Gelaspan is normally given to immobile patients in a controlled setting (e.g.

emergency treatment, acute treatment in a hospital or a day therapy unit). This will exclude driving and using machines.

3. HOW TO USE GELASPAN

Gelaspan is given intravenously, i.e. by a drip.

Adults

How much you are given and for how long will depend on how much blood or fluid you have lost and on your condition.

Children:

There is only little experience of the use of Gelaspan in children. Your doctor will only administer this medicine to your child if he/she considers it essential for your child's recovery. In those cases the clinical condition of your child will be taken into account and his/her therapy will be monitored especially carefully The doctor will carry out tests (on blood and blood pressure, for example) during treatment, and the dose of Gelaspan will be adjusted according to the patients needs.

In case of pressure infusion, all air must be removed from the container and the infusion set before the solution is administered.

If you received more Gelaspan than you should

An overdose of Gelaspan may cause too high blood volume (hypervolaemia), circulatory overload and imbalances of your blood composition.

You may notice the following symptoms:

- impairment of heart-and lung function
- headache, difficulties to breathe, congestion of blood in the jugular vein If an overdose occurred your doctor will give you any necessary treatment. If you have any further questions on the use of this product, ask your doctor or pharmacist.

4. POSSIBLE SIDE EFFECTS

Like all medicines, Gelaspan can cause side effects, although not everybody gets them.

In this section, side effects are ranked according to their frequency using the following terms:

very common: affecting more than 1 treated patient of 10 common: affecting 1 to 10 treated patients of 100

uncommon: affecting 1 to 10 treated patients of 1,000

rare: affecting 1 to 10 treated patients of 10,000

very rare: affecting less than 1 treated patient of 10,000

not known: cannot be estimated from the available data

The following side effects may become serious and require immediate medical treatment:

Rare:

- allergic skin reactions such as hives or nettle rash
- other allergic (anaphylactoid) reactions, including e.g. difficulty breathing, wheeze, nausea, vomiting, dizziness, sweating, chest or throat tightness, stomach ache, swelling of neck and face

If an allergic reaction, especially an anaphylactoid reaction occurs your infusion will be stopped immediately and you will be given any necessary treatment.

Very rare:

- quickening of heartbeat
- severe allergic (anaphylactoid) reactions such as drop of blood pressure, confusion, involuntary excretion of urine, blue coloration of the skin and mucous membranes (so-called cyanosis) and extremely rare cases of loss of consciousness and collapse.

BIBRAUN

Schwarz 210x700 mm 343/NP34332/0115 Latus: 7385 GroBbritannien/Irland Font size 8 SPC; 9 PIL

343/NP34332/0115

	Uncommon (> 1/1,000 to < 1/100)	Rare<br>(> 1/10,000 to < 1/1,000)	Very rare (< 1/10,000)
**Immune system disorders**		Anaphylactoid reactions, all grades*	Severe anaphylactoid reactions*
**Cardiac disorders**			Tachycardia
**Vascular disorders**			Hypotension
**Respiratory, thoracic and mediastinal disorders**			Respiratory

			difficulties
**Skin and subcutaneous tissue disorders**		Allergic skin reactions*	
**General disorders and administration site conditions**	Mild transient increase of body temperature		Fever, chills

Mild anaphylactoid reactions include:

Generalised oedema, urticaria, periorbital oedema, or angiooedema.

Moderate anaphylactoid reactions include:

Dyspnoea, stridor, wheeze, urticaria, nausea, vomiting, dizziness (presyncope), diaphoresis, chest or throat tightness, or abdominal pain.

Severe anaphylactoid reactions include:

Cyanosis or SaO_2 < 92% at any stage, hypotension

(systolic blood pressure < 90 mmHg in adults), confusion, collapse, loss of consciousness or incontinence.

In the event of an anaphylactoid reaction, the infusion must be discontinued immediately and the usual acute treatment given.

Paediatric patients:

No special features

Reporting of suspected adverse reactions

Reporting suspected adverse reactions after authorisation of the medicinal product is important. It allows continued monitoring of the benefit/risk balance of the medicinal product. Healthcare professionals are asked to report any suspected adverse reactions via the following:

United Kingdom - Yellow card scheme: www.mhra.gov.uk/yellowcard Ireland

HPRA Pharmacovigilance, Earlsfort Terrace, IRL - Dublin 2;

Tel: +353 1 6764971; Fax: +353 1 6762517.

Website: www.hpra.ie; e-mail: medsafety@hpra.ie

4.9 Overdose

Symptoms

Overdose of Gelaspan may cause hypervolaemia and circulatory overload with a significant fall in haematocrit and plasma proteins. This may be associated with consecutive impairment of heart and lung function (pulmonary oedema). Symptoms of circulatory overload are e.g. headache, dyspnoea, and jugular vein congestion. *Treatment*

In case of circulatory overload the infusion must be stopped and a rapid-acting diuretic should be given. If an overdose occurs, the patient should be treated symptomatically and electrolytes should be monitored.

5. PHARMACOLOGICAL PROPERTIES

5.1 Pharmacodynamic properties *Pharmaco-therapeutic group* Blood substitutes and plasma protein fractions ATC code: B05A A06, gelatine agents.

Gelaspan is a 4 % w/v solution of succinylated gelatine (also known as modified fluid gelatine) with an average molecular weight of 26 500 Dalton (weight average) in a plasma-adapted, balanced isotonic electrolyte solution. The negative charges introduced into the molecule by succinylation cause an expansion of the molecule. The molecular volume is therefore higher than that of unsuccinylated gelatine of the same molecular weight.

The measured initial volume effect of Gelaspan is about 100% of the infused volume with a sufficient volume effect over 4 - 5 hours.

Gelaspan does not interfere with the determination of blood groups and it is neutral regarding clotting mechanisms.

Therapeutic effect

Gelaspan substitutes intra- and extravascular volume deficits caused by losses of blood, plasma and interstitial fluid. Thus the mean arterial pressure, the left-ventricular end-diastolic pressure, the cardiac stroke volume, the cardiac index, the oxygen supply, the microcirculation and the diuresis are increased without dehydrating the extravascular space.

Mechanisms of action

The colloid-osmotic pressure of the solution determines its initial volume effect. The duration of the effect depends on the clearance of the colloid mainly by renal excretion. Since the volume effect of Gelaspan is equivalent to the administered amount of solution, Gelaspan is a plasma substitute, not a plasma expander. The solution also restores the extravascular compartment, does not disturb the electrolyte balance of the extracellular space. Gelaspan is isotonic, it therefore does not cause fluid shifts into the intracellular space as caused by hypotonic solutions. Gelaspan contributes in the restoration of electrolyte balance and the correction of acidosis. Gelaspan is lactate free and can be used in patients with liver diseases. As a precursor of bicarbonate the solution contains acetate which is metabolisable in all organs and muscles.

5.2 Pharmacokinetic properties *Distribution*

After infusion, Gelaspan is rapidly distributed in the intravascular compartment. There is no evidence that Gelaspan is stored in the reticulo-endothelial system or elsewhere in the organism.

Metabolism/elimination

Most of the infused Gelaspan is excreted via the kidneys. Only a minor amount is excreted in faeces and not more than about 1 % is metabolised. The smaller molecules are excreted directly by glomerular filtration while the larger molecules first are degraded proteolytically and then excreted via kidneys. The proteolytic metabolism is so adaptable that even under the condition of renal insufficiency no accumulation of Gelaspan is observed.

Pharmacokinetics in special clinical situations

The plasma half-life of Gelaspan may be prolonged in patients on haemodialysis (GFR < 0.5 ml/min). Gelaspan minimizes the risks of dilutional acidosis and rebound alkalosis as observed with lactate containing solutions infused to patients with liver diseases. Gelaspan contains acetate and is lactate free. It therefore can also be indicated in hypovolaemic patients with liver disease.

5.3 Preclinical safety data

Non-clinical data for the individual components of Gelaspan reveal no special hazard for humans based on conventional studies of single and repeated dose toxicity. There is no or limited non-clinical data available for reproductive toxicity.

The maximum dose of the product is limited by its volume and dilution effects, not by any intrinsic toxicological properties.

6. PHARMACEUTICAL PARTICULARS

6.1 List of excipients

Sodium hydroxide (for pH adjustment),

Hydrochloric acid, diluted (for pH-adjustment),

Water for injections

6.2 Incompatibilities

In the absence of compatibility studies, this medicinal product must not be mixed with other medicinal products.

6.3 Shelf life

- *Unopened*

Polyethylene containers "Ecoflac plus": 2 years Plastic bags "Ecobag" (non-PVC): 2 years

- *After first opening the container*

The infusion should commence immediately after connecting the container to the giving set.

- *After admixture of an additive* Not applicable (see section 6.2)

6.4 Special precautions for storage Do not store above 25 °C. Do not freeze.

6.5 Nature and contents of container Gelaspan is supplied in:

- Bottles of low-density polyethylene "Ecoflac plus", contents: 500 ml available in packs of 10 x 500 ml
- Plastic bags "Ecobag" (non-PVC), sealed with halogenbutyl rubber stoppers contents: 500 ml

available in packs of 20 x 500 ml Not all pack sizes may be marketed

6.6 Special precautions for disposal and other handling

No special requirements for disposal

The product is supplied in containers for single use only. Unused contents of an opened container must be discarded.

Only to be used if solution is clear and free of precipitate and the container undamaged.

Use immediately after connecting container to the giving set.

7. MARKETING AUTHORISATION HOLDER

B. Braun Melsungen AG Carl-Braun-StraBe 1 34212 Melsungen, Germany *Postal address* 34209 Melsungen

8. MARKETING AUTHORISATION NUMBER(S)

PA 736/34/1 (Ireland)

PL 03551/0120 (United Kingdom)

9. DATE OF FIRST AUTHORISATION / RENEWAL OF THE AUTHORISATION

Date of first authorisation:

9th September 2011 (Ireland)

14th June 2011 (United Kingdom)

10. DATE OF REVISION OF THE TEXT

September 2014

BIBRAUN

B. Braun Melsungen AG

34209 Melsungen, Germany

#-----------#-----------#-----------#

Such reactions are assumed to be more frequent in patients with known allergic conditions such as asthma.

Unfortunately, there is no test which can show in advance who is likely to experience such reactions, nor can their course be predicted.

Other side effects include:

Uncommon:

- mild short lasting increase of body temperature

Very rare:

- fever, chills

If any of the side effects gets serious, or if you notice any side effects not listed in this leaflet, please tell your doctor or pharmacist.

Reporting of side effects

If you get any side effects, talk to your doctor or pharmacist. This includes any possible side effects not listed in this leaflet. You can also report side effects directly (see details below). By reporting side effects you can help provide more information on the safety of this medicine.

United Kingdom:

Yellow Card Scheme

Website: www.mhra.gov.uk/yellowcard

Ireland:

HPRA Pharmacovigilance, Earlsfort Terrace, IRL - Dublin 2; Tel: +353 1 6764971; Fax: +353 1 6762517. Website: www.hpra.ie; e-mail: medsafe-ty@hpra.ie

5. HOW TO STORE GELASPAN

Keep out of the reach and sight of children.

Do not use Gelaspan after the expiry date which is stated on the label and the outer carton. The expiry date refers to the last day of that month.

Do not store above 25 °C. Do not freeze.

Do not use Gelaspan if you notice:

- cloudiness or discolouration of the solution
- leaking of the container.

Previously opened or partly used Gelaspan should be thrown away. Partially used bottles or bags should not be reconnected.

6. FURTHER INFORMATION

What Gelaspan contains

Active substances:

1000 ml of the solution contain:

Succinylated (modified fluid) gelatine	40.0 g
Sodium chloride	5.55 g
Sodium acetate trihydrate	3.27 g
Potassium chloride	0.30 g
Calcium chloride dihydrate	0.15 g
Magnesium chloride hexahydrate	0.20 g

Electrolyte concentrations	
Sodium	151 mmol/l
Chloride	103 mmol/l
Potassium	4 mmol/l
Calcium	1 mmol/l
Magnesium	1 mmol/l
Acetate	24 mmol/l

The other ingredients are:

Water for injections, hydrochloric acid, diluted (for pH-adjustment) and sodium hydroxide (for pH adjustment).

What Gelaspan looks like and contents of the pack

Gelaspan is a solution for infusion administered through an intravenous drip (a drip into a vein).

It is a clear colourless or slightly yellowish sterile solution.

Gelaspan is supplied in:

- Bottles of low-density polyethylene "Ecoflac plus", contents: 500 ml available in packs of 10 x 500 ml
- Plastic bags "Ecobag" (non-PVC), sealed with rubber stoppers, contents: 500 ml

available in packs of 20 x 500 ml Not all pack sizes may be marketed.

Marketing Authorisation Holder

B. Braun Melsungen AG Carl-Braun-StraBe 1 34212 Melsungen, Germany *Postal address* 34209 Melsungen

Manufacturer

B. Braun Melsungen AG Carl-Braun-StraBe 1 34212 Melsungen, Germany *Postal address* 34209 Melsungen Phone: +49-5661-71-0 Fay: +49-5661-4567

This medicinal product is authorised in the Member States of the EEA under the following names:

Austria Gelofusin Iso 40mg/ml Infusionsldsung

Belgium Isogelo oplossing voor infusie, solution pour perfusion,

Infusionsldsung

Bulgaria Gelofusine Balance 4% solution for Infusion

Czech Republic Gelaspan 4%

Germany Gelafundin ISO 40mg/ml Infusionsldsung

Denmark Gelaspan

Estonia Gelaspan infusioonilahus 4%

Greece Gelaspan solution for Infusion 4%

Spain Gelaspan 40mg/ml solucion para perfusion

Finland Gelaspan

France Gelaspan, solution pour perfusion

Hungary Gelaspan 4% oldatos infuzio

Ireland Gelaspan Solution for Infusion

Italy Gelaspan

Lithuania Gelaspan 4% infuzinis tirpalas

Luxemburg Gelafundin ISO 40mg/ml Infusionsldsung

Latvia Gelaspan 4% Solution for Infusion

Malta   Gelaspan 4% Solution for Infusion

Norway   Gelaspan

Netherlands Gelaspan, oplossing voor infusie

Portugal   Gelaspan

Poland   Gelaspan

Romania   Gelaspan 40 mg/ml solutie perfuzabila

Sweden   Gelaspan

Slovenia   Gelaspan 40 mg/ml raztopina za infundiranje

Slovakia   Gelaspan 4%

United Kingdom Gelaspan solution for infusion

This leaflet was last approved in January 2015

The following information is intended for health-care professionals only:

Precautions for use

Gelaspan must not be infused through the same infusion line together with blood or blood products (packed cells, plasma and plasma fractions). During compensation of severe blood losses by infusions of large amounts of Gelaspan, haematocrit and electrolytes must be monitored. The haema-tocrit should not decrease below 25 %. In elderly or critically ill patients it should not fall below 30%.

Likewise in those situations the dilution effect on coagulation factors should be observed, especially in patients with existing disorders of haemostasis.

Because the product does not substitute lost plasma protein, it is advisable to check the plasma protein concentrations.

Influence on laboratory tests

Laboratory blood tests (blood group or irregular antigens) are possible after Gelaspan infusions. Nevertheless it is recommended to draw blood samples before the infusion of Gelaspan in order to avoid hampered interpretation of results.

Gelaspan may have an influence on the following clinical-chemical tests, leading to falsely high values:

- erythrocyte sedimentation rate,
- specific gravity of urine,
- unspecific protein assays, e.g. the Biuret method.

Incompatibilities

In the absence of compatibility studies, this medicinal product must not be mixed with other medicinal products.

BIBRAUN

B. Braun Melsungen AG

34209 Melsungen Germany

Schwarz 210x700 mm 343/NP34332/0115 Latus: 7385 GroBbritannien/Irland Font size 8 SPC; 9 PIL

© 2018 • Terms of use • Privacy policy • Contact
